# Protocol for a randomized controlled trial of a combined motivational interviewing and behavioral couples therapy intervention to reduce intimate partner violence and alcohol use in south India

**DOI:** 10.1371/journal.pone.0335332

**Published:** 2025-12-01

**Authors:** Meghna Achar, Megan Ramaiya, Krishnamachari Srinivasan, Maria L. Ekstrand, Elsa Heylen, Johnson Pradeep R, Miriam Hartmann, Nisha C. K, Matilda Pereira, Bibhav Acharya

**Affiliations:** 1 Division of Mental Health and Neurosciences, St. John’s Research Institute, St. John’s Medical College, Bengaluru, Karnataka, India; 2 Department of Psychiatry and Behavioral Sciences, University of California, San Francisco, San Francisco, California, United States of America; 3 Department of Psychiatry, St. John’s Medical College Hospital, St. John’s Medical College, Bengaluru, Karnataka, India; 4 Department of Medicine, Division of Prevention Science, University of California, San Francisco, California, United States of America; 5 Women’s Global Health Imperative, RTI, Berkeley, California, United States of America; 6 Department of Global Public Health, Karolinska Institute, Stockholm, Sweden; PLOS: Public Library of Science, UNITED KINGDOM OF GREAT BRITAIN AND NORTHERN IRELAND

## Abstract

There is a strong association between alcohol use disorder (AUD) and intimate partner violence (IPV), both widely prevalent global health issues. However, few interventions target both IPV and AUD, include both partners in the intervention, and are delivered by non-specialist providers in low- and middle-income country (LMIC) settings with scarce mental health resources. This paper describes the protocol for a randomized controlled trial of a combined motivational interviewing (MI) and behavioral couples therapy (BCT) intervention delivered in urban primary care settings in India by nurses with no behavioral health training prior to joining the study. A total of 400 couples will be enrolled and randomized to one of two arms: an intervention arm comprised of 10, hour-long sessions of the MI + BCT intervention, and a control arm receiving enhanced usual care and medical-legal referrals. Data collection will take place at five timepoints: baseline (pre-intervention), three-, six-, nine-, and 12-month follow-ups. Primary quantitative outcomes include the frequency of intimate partner violence over the last 6 months and self-reported quantity and frequency of alcohol use, drinking behaviors, and alcohol-related problems as assessed on the Alcohol Use Disorders Identification Test (AUDIT). Secondary outcomes include number of days with a negative breathalyzer test over a one-week period, communication patterns, and the quality of marital relationship. Qualitative interviews with a sub-sample (n = 40 couples) from the intervention arm will take place immediately post-intervention and at 12 months to explore underlying mechanisms of change. If successful, study results can inform future efforts to develop scalable interventions for IPV and AUD that can be sustained in the Indian public health system through existing PHC staff and infrastructure and be adapted to similar sociocultural settings.

## Introduction

Alcohol use disorder (AUD) and intimate partner violence (IPV) are interconnected issues with significant adverse global public health implications [1]. Globally, an estimated 30% of women report physical or sexual violence by an intimate partner in their lifetime [2]. An increasingly large body of evidence globally supports a causal relationship between AUD and IPV, defined as psychological, physical, and sexual violence, as well as abusive control, by a close partner [1,3,4]. AUD is associated with lowered inhibitions and distorted perception of cues resulting in increased aggression [5–7]. There is growing consensus that AUD in one or both partners is associated with severity and frequency of violence globally [1–3,8,9], including in India [10–16].

In a 2019–2021 national survey among ever-married women in India, 29% reported experiencing physical spousal violence [17], while multi-site studies estimate that up to 52% of Indian women have experienced some form of spousal abuse in their lifetime [18]. Studies in India have found that women with spouses who drink alcohol are more likely to experience violent events [10–16]. Women in India who report IPV victimization can experience poor short- and long-term health outcomes including increased risk for sexually transmitted infections and HIV [19,20], poor maternal health outcomes, [21] and increased suicide risk [20].

Despite strong links between IPV and AUD, there is a dearth of effective and scalable interventions that simultaneously target both AUD and IPV. A 2019 global meta-analysis found few interventions that targeted both AUD and IPV in the United States, Europe, Africa and India, [22] and among those, none were successful in changing both target behaviors, [22–27] highlighting a large scientific gap. Furthermore, interventions that have been successful at reducing either alcohol use or IPV are delivered by mental health professionals, limiting scalability [22]. Interventions in the US [23–25] have employed cognitive behavioral therapy (CBT) techniques using group and individual sessions delivered by trained masters-level therapists [23,25] to strengthen problem-solving and communication skills among incarcerated men. These studies found a significant reduction in self-reported days of alcohol use, [23] and participants were less likely to report engaging in aggressive behaviors after drinking episodes [25] but found no significant difference in reported frequency of physical IPV. Other studies in the US added substance use treatment in addition to IPV treatment as usual but did not find statistically significant changes in outcomes [28,29].

Evidence from global systematic reviews of AUD and IPV intervention studies suggest two major gaps. First, most often, only the male partner was engaged in the intervention [22–27,30]. Despite ongoing IPV, many women, particularly from LMIC settings, cannot or may not want to leave their partners [31]. In such cases, working with couples on communication and conflict negotiation has been effective at reducing violence [32–38], suggesting that couples therapy may be more effective than individual treatment. The second gap is that most studies failed to integrate treatment for both AUD and IPV [30]. Behavioral couples therapy (BCT), which is based on principles of CBT and improvement of interpersonal relationships, can be effective at reducing IPV [27,39]. When BCT is offered when the husband has AUD, it is important to first deliver a program to reduce his alcohol use [27,39,40]. Given the pervasiveness of IPV and its association with AUD, there is a strong need for evidence-based interventions that overcome these gaps, are culturally appropriate, and are tailored to the population of interest.

Our study team conducted a pilot intervention in 2017 in Bengaluru, India [36] in which BCT was combined with contingency management (an empirically supported, behavioral substance use treatment) to reduce AUD and IPV among 60 couples, including both partners and combining treatments for both AUD and IPV. Results showed that the intervention significantly reduced men’s alcohol use (measured by daily breathalyzer testing) and IPV (based on the Indian Family Violence and Control Scale (IFVCS) [41] at one- and four-month follow-up periods. Participant couples reported the intervention did not lead to an increase in violence, an important concern when enrolling couples in IPV interventions. The BCT component was described as a strategy to improve communication and relationships, which improved acceptability, recruitment, and retention of vulnerable couples.

The current RCT is based on the results of this pilot study, replacing the contingency management component with motivational interviewing (MI), a robust intervention with substantial evidence in reducing AUD in multiple populations globally [42], including India [43]. This increases the scalability of the intervention by avoiding concerns of ongoing monetary incentives as part of contingency management for alcohol use. To ensure the intervention has the highest likelihood of success and eventual scale-up, we trained nurses to deliver MI and BCT. Several studies have shown that nurse-led MI can successfully reduce alcohol use [43–49] and improve multiple other health outcomes [50–55]. In India, MI has been used widely [56,57], including by non-mental-health professionals to reduce alcohol use [49,58]. These findings demonstrate that in primary care settings, where there is a shortage of mental health professionals, training nurses in MI is feasible and effective at reducing AUD. Our study will thus address gaps in the current literature by using trained nurse-counselors to deliver MI and BCT to reduce AUD and IPV.

### Study objective

**Fig 1:**
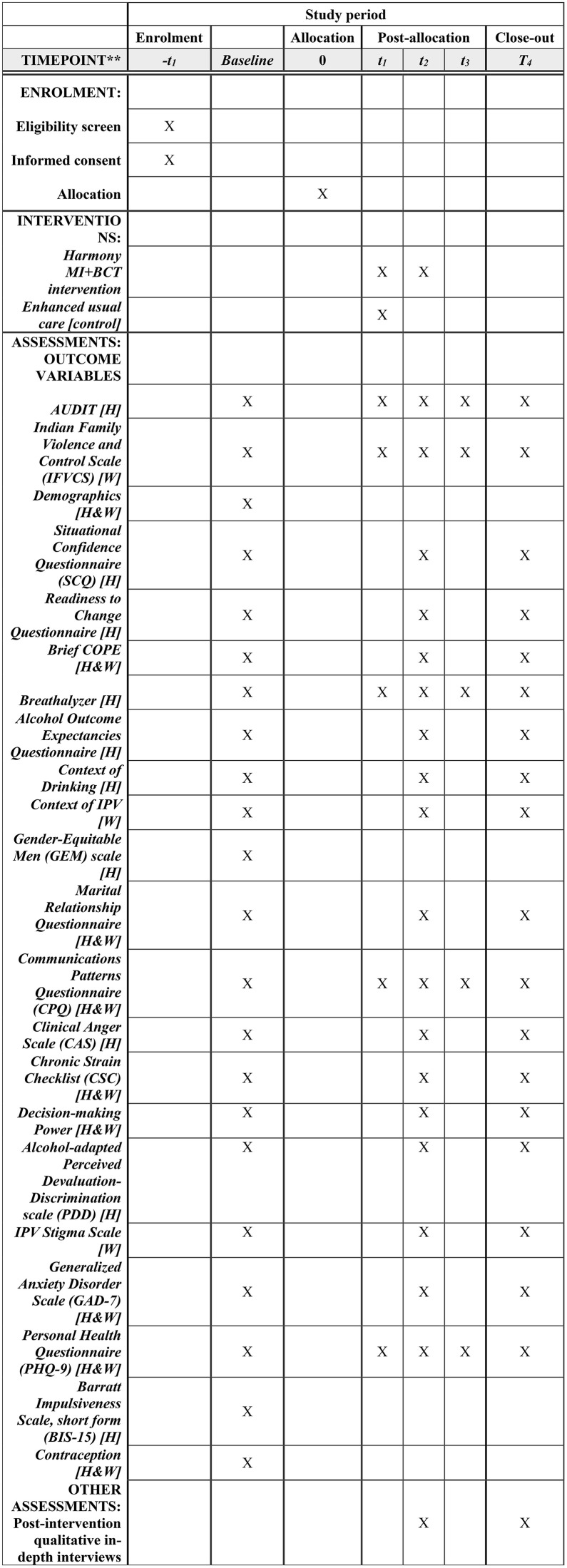
SPIRIT schedule of enrollment, intervention, and assessment.

This study builds on the prior evidence base and existing infrastructure at primary health clinics in south India by conducting a randomized controlled trial (N= 400 couples) to study the efficacy of MI and BCT in reducing IPV behaviors and AUD among the men. The intervention is delivered by study nurses in communities that are part of the catchment areas of local government-run primary health centers (PHCs) providing services for underserved populations. We will also assess secondary outcomes. Other key measures will be assessed utilizing mixed methods to explore theorized mechanisms of change influencing intervention efficacy. If successful, our study will provide evidence for an intervention for IPV and AUD that can be adapted and delivered by non-specialists in primary care settings in South Asia and other resource-constrained settings with similar cultural norms (see Fig 1).

### Theoretical framework

**Fig 2:**
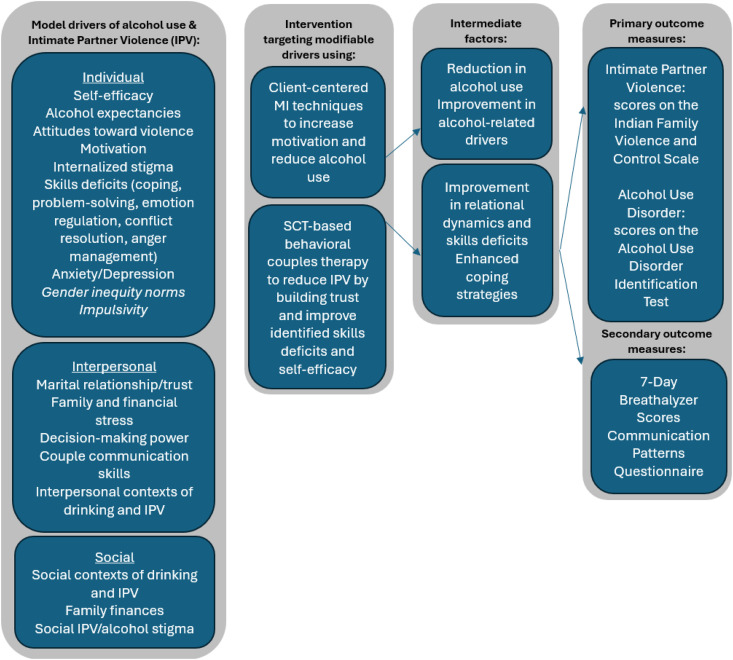
Conceptual model to reduce incidence of intimate partner violence via reduction of alcohol use and improvement of relational dynamics based on Social Cognitive Theory, Motivational Interviewing, and empirical data from pilot studies. Hypothesized moderators are listed in italics.

The intervention, named “Harmony”, is guided by an integration of Social Cognitive Theory (SCT) [59], MI, and findings from our pilot study [36] (see Fig 2). MI is a “client–centered, directive method for enhancing intrinsic motivation to change by exploring and resolving ambivalence” [60] and is a well-established behavioral intervention strategy to reduce AUD in multiple global settings, including India [42,41,47,48,58]. Because of its tailored nature, MI is an effective strategy to address behavior change to reduce alcohol use while encouraging development of self-efficacy and behavioral skills and is compatible with the SCT framework [60].

SCT is a strong fit for this intervention because of its emphasis on interpersonal interactions and environmental influences on behaviors, as well as on specific strategies that promote behavior change, all important to reduce barriers and promote facilitators of change. Women often have few options to leave their marriage [35], are subject to the broader norms of their family and society, and often express commitment to staying with their husbands despite ongoing violence [61]. SCT allows us to incorporate these factors at the individual, couple, and environmental levels.

SCT is especially relevant to alcohol reduction efforts as self-regulatory capacity and self-efficacy – central tenets of both MI and SCT – are salient to reducing alcohol use among participants with AUD [59,62]. Self-efficacy, which refers to a person’s confidence in their ability to succeed at a specific task, is a key element in motivation to change. Self-efficacy changes because of learning skills, having formative experiences, and receiving feedback. BCT is rooted in social learning theory and focuses on improving communication, trust, and conflict negotiation skills to enhance the relationship between partners [40]. The key drivers of alcohol use and perpetration of IPV that we have included in the model are antecedents or precipitants that are proximally associated with IPV perpetration and are malleable to a brief psychosocial intervention. While we do not expect that the intervention will impact gender norms or impulsivity, these factors may moderate the intervention effects and will be assessed at baseline. Proximal antecedents of alcohol use and IPV perpetration include contextual factors linked to alcohol use, situational factors linked to violence, cognitive variables (motivation, self-efficacy), affect (anxiety), stress, and behavioral measures such as relationship, communication, coping strategies, and anger control [63–66]. See Fig 2 for a detailed conceptual model.

The two-step intervention strategy proposed is in alignment with the recommendations by O’Farrell and colleagues [40] that BCT be coupled with an intervention directed at reducing harmful drinking. This strategy may create short-term behavior change and increase the likelihood of success of BCT in enhancing the relationship via skills training. MI is intended to provide the requisite reduction in alcohol use to enable the couple to participate in and benefit from the BCT sessions. MI’s client-centered approach trains counselors to express empathy and to adjust to, rather than confront, the discord expressed by people with AUD. MI strategies also help clients discover discrepancies between their goals and their behaviors while enhancing self-efficacy. This approach is consistent with skill-building strategies which are cornerstones of SCT-based interventions, including BCT.

Our pilot study showed that the reduction in violence was likely attributable to reductions in the husband’s use of controlling behaviors towards their female partner, which in turn may have resulted from improved communication skills combined with greater self- and emotion regulation due to reductions in drinking [36]. These results support the use of our integrated SCT-MI conceptual model for improving motivation to reduce harmful drinking, which can lead to improved self-regulatory capacity, followed by skills training and self-efficacy, ultimately promoting healthy couples’ interactional behavior and reducing IPV.

## Materials & methods

The trial has been registered with clinicaltrials.gov (*Trial registration number: NCT05893277)* and with the Clinical Trials Registry – India *(CTRI registration number: CTRI/2022/09/045513)*.

### Study design

**Fig 3:**
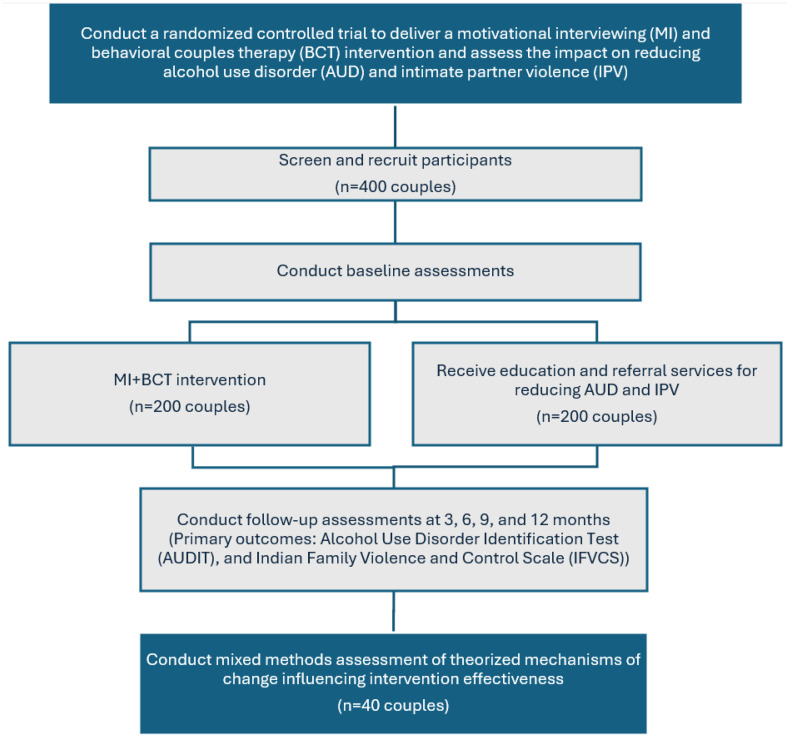
Study design and flow.

The study design (Fig 3) is a mixed methods randomized controlled trial (RCT) of the combined MI and BCT Harmony intervention. Couples will be randomized 1:1 to the control group (n = 200 couples) and the intervention group (n = 200 couples). The dyadic intervention condition consists of 10, hour-long sessions and involves spouses in 3 individual and 7 joint sessions over a five-month period. The control condition consists of enhanced usual care, which includes safety assessments and referrals for IPV resources for the women and brief psychoeducation for AUD for the men. Assessments will be conducted at baseline, 3-, 6-, 9-, and 12-month follow-up. A subset of couples in the intervention arm (n = 40) will be randomly selected to partake in the post-intervention qualitative in-depth interviews (IDIs).

### Study setting

The study is conducted in regions served by the government-run urban primary health centers (PHCs) in Bengaluru, India. The Bengaluru metropolis includes several PHCs, and each center serves as a nodal point for delivery of health services to primarily low-income populations in low-resource settings, ranging from 50,000 to 75,000 people per catchment area. The PHCs provide comprehensive primary care, basic emergency care, referrals, basic laboratory testing, pharmacy services, antenatal care, family planning, and immunization services. They also serve as the focal point for outreach work and other public health interventions in the catchment area. Each PHC includes several healthcare workers, including community health workers called Accredited Social Health Activists (ASHAs). Our study will engage eight to nine PHCs and several community-based organizations that operate within the catchment areas of these PHCs for recruitment.

### Eligibility criteria

#### Inclusion criteria.

For all participants, eligibility criteria include: i) married with both spouses aged ≥18 years; ii) living within the catchment area of the PHC; iii) Kannada-speaking; iv) woman reporting physical or sexual IPV in the past 12 months (per adapted version of the International Violence against Women Survey [67] – IVAWS); and v) the husband with AUD (AUDIT-C ≥ 4). [68]

#### Exclusion criteria.

Exclusion criteria include: i) husband has severe alcohol dependence (Severity of Alcohol Dependence Questionnaire, [69] SADQ ≥ 31) or is at risk of severe withdrawal symptoms based on Clinical Institute Withdrawal Assessment for Alcohol Scale–Revised [70] (CIWA-AR); ii) significant medical problems that will make the couple unable to participate in the intervention sessions; iii) cognitive problems (Short-Blessed Cognitive Test [71] score ≤7); iv) past year history of IPV severe enough to result in hospitalization; or v) wife is positive for AUD (AUDIT-C ≥ 4) at screening [68].

### Sample size determination

Sample size was determined based on the power necessary to detect a meaningful intervention effect at 12 months in the continuous primary outcomes. Based on our pilot results, [36] we expect the intervention group to have a 4-5 point lower mean IFVCS score compared to the control group at the end of the study (SD = 14). With alpha = 0.05, power = 80%, this requires an effective sample size of n = 320. Allowing for 20% attrition, the initial sample size necessary is 400 couples. This anticipated effect translates into a standardized minimum detectable effect size of d = 0.31, a small effect according to Cohen. [71] Since we will treat the AUDIT as continuous as well, we are similarly powered for a significant minimum detectable effect size of 0.31 standard deviations difference between the two groups on this second primary outcome. This is within our expectations based on a meta-analysis [72] that found a standardized mean difference of 0.29–0.38 with brief one-session interventions or usual care, respectively.

### Recruitment & retention

Our recruitment plan is guided by the successful processes we have implemented in prior community-based studies [36]. ASHAs are women residents of the community who are selected to be trained and deployed to work alongside the local PHC to improve the health of all community residents by ensuring they have access to medical treatment; their trifold role includes that of a health activist, link-worker, and a community health worker, all of which make them an ideal fit for their study role in identifying and referring potentially eligible couples within their community. The ASHA will identify couples they know are experiencing IPV in which the husband is also using alcohol. Following consultation with the research team, the ASHA will bring the couple to the PHC, the Namma Clinic (an urban healthcare and wellness centers affiliated with the PHC), or community spaces owned by local private and religious organizations for screening.

The intervention will be presented to participants as one that is aimed at enhancing communication and improving relationship quality between spouses (and, critically, not an IPV intervention). The wife will be screened using the SBT [73] to rule out cognitive deficits impeding optimal study participation, AUDIT-C to rule out AUD, [68] and IVAWS [67] to identify physical and/or sexual violence in the past 12 months and to rule out violence severe enough to have resulted in the woman being admitted at a hospital or lodging police complaints. The husband will be screened using the SBT to rule out cognitive deficits impeding optimal study participation, [70] AUDIT-C to identify AUD (score>4), [67] SADQ to rule out severe alcohol dependence (score≥31), [69] and CIWA-AR to screen out participants who are at high risk of developing withdrawal symptoms (score≥8) [70]. Based on our prior experiences, we conservatively estimate needing to screen 2,350 couples to enroll 400. All participants who are ineligible for the study but screen positive for AUD and/or IPV will receive a brief educational session [74] and a referral to the National Institute of Mental Health and Neurosciences (NIMHANS) in Bangalore, a premier tertiary care institute which offers counseling and psychiatry services as well as a dedicated de-addiction treatment for severe alcohol dependence and alcohol withdrawal.

Research staff will screen and obtain informed consent with the spouses separately. Due to varying literacy levels, the informed consent form will be read aloud to the participant. The participant will be asked to narrate back to the staff what they understand and is offered opportunities to ask questions. The participant will then insert their signature or their thumbprint on the form in the presence of an adult witness, followed by the obtaining of the signature or thumbprint of the adult witness. Following informed consent to participate in the study, the staff will enroll eligible couples in the study, conduct baseline assessments and randomly assign them to the intervention or enhanced usual care arm, as shown in Fig 3.

### Training

All nurses delivering the intervention will receive intensive training in MI and BCT from the study intervention coordinator who is a trained clinical psychologist (author MA), under the guidance of co-authors (MH and KS) with expertise in BCT and co-authors (JPR and NCK and ME) with expertise in MI. Staff will also be trained by a study consultant with expertise in gender-based health, as well as safety procedures for responding to participant distress. Training will takes place over one month and includes a combination of didactics, video, and interactive role-plays. Trained nurses-counselors will be tested on general counseling skills, MI-specific skills, and session-by-session MI + BCT manual components and participant handouts, followed by repeated mock sessions with supervisor and peer feedback. At least two independent raters will use intervention fidelity checklists to observe and evaluate each nurse’s delivery of all sessions and certify them to be nurse-counselors before they begin delivering the intervention to enrolled couples. Nurses who do not meet the threshold on the fidelity checklist will be re-trained until they meet certification criteria.

### Procedures

#### Intervention arm.

The Harmony intervention consists of 10, hour-long sessions delivered once weekly (Table 1) during a three- to five-month period. Initially, the husband receives three individual sessions of MI as well as one session jointly with his wife to address alcohol use, which is the standard primary care-based intervention to reduce AUD in community populations. The MI sessions focus on specific individual, interpersonal, and social factors driving disordered alcohol use by utilizing the four-step process of engaging, focusing, evoking and planning. [62] The intervention is presented to participants as a means of improving the couple’s communication and marital relationship (rather than explicitly as an AUD/IPV intervention) to reduce the potential stigma of attending sessions focused on AUD/IPV. The husband receives training in drink refusal, emotion regulation, problem-solving, and urge management skills. The nurse-counselor co-constructs a weekly change-and-action plan to help the husband use these skills between sessions. The nurse-counselor invites the wife to the fourth MI session, which orients the couple to lapse and relapse prevention and management. This joint session marks the transition to BCT. Following this, both spouses receive six sessions of BCT together [40] to improve interpersonal communication, caring, and trust.

**Table 1:** Summary of MI* + *BCT Intervention.

Session Number	Session Content	Session Participants
1-4	• Engaging, focusing, evoking and planning • Drink refusal, emotion regulation, problem solving, and urge management skills • Relapse prevention and management	Husband in 1–3 Husband and Wife in 4
5	• Trust: Daily Trust Contract	Husband and Wife
6	• Coping: Catching yourself, time-out, diaphragmatic breathing, relaxation, positive self-talk	Husband and Wife
7	• Effective communication • Joint problem-solving	Husband and Wife
8	• Caring: Catching your partner being nice and telling them	Husband and Wife
9	• Active listening • Expressing feelings directly using I-statements	Husband and Wife
10	• Review and wrap-up	Husband and Wife

The first BCT session focuses on the development of a daily trust contract in which the husband verbally commits to reducing his drinking while his wife acknowledges her support and appreciation. The second BCT session discusses specific strategies to cope with stress and reduced alcohol use (including time-outs, a diaphragmatic breathing exercise, and engaging in positive self-talk). Effective communication and joint problem-solving are core components of session three, followed by mutually caring activities in BCT session 4. The fifth BCT session expands on effective communication by helping couples learn and practice active listening and using “I” statements to reciprocally express feelings in a direct manner. The last BCT session includes review and wrap-up, with couples revisiting useful topics and skills and planning for long-term improvement in alcohol use and relationship quality. All sessions include role plays and end with simple homework practice assignments. In addition, nurse-counselors use in-session charts with visuals and limited text, due to variability in participant literacy levels, to enhance participant comprehension and reinforce lessons and skills taught.

The intervention window is five months to allow for missed visits due to family holidays, illness, and travel. During their first visit, the couple is informed that session scheduling attempts will be discontinued after five months, regardless of the number of sessions attended at that point.

#### Intervention fidelity.

Motivational Interviewing Treatment Integrity (MITI) [75] will be used to ensure MI fidelity, rating all nurse-counselors on content coverage, MI-specific skills, and general counseling skills. For BCT, a standard checklist updated from the pilot [36] will be used to evaluate nurse-counselors on content coverage and overall session delivery quality. These checklists will be used to certify the nurse-counselors before they begin delivering the intervention in the trial and to assess ongoing sessions during the study. A clinical psychologist will directly observe and review 10% of all sessions per nurse-counselor, including 20 and 30 randomly selected MI and BCT sessions, respectively. Daily check-ins with each nurse-counselor will be carried out by the clinical psychologist to track couples and discuss challenges, if any, with regards to scheduling and conducting the sessions. Weekly group supervision sessions will include presentation of each intervention “case” or couple by the respective nurse-counselor and a discussion of session content, processes, and strategies for addressing challenges.

#### Control arm.

Our prior work in India has shown a lack of availability of care for AUD and/or IPV in PHCs, as well as lack of adherence to existing protocols. [76,77] To meet ethical guidelines, the control arm participants receive a single session of enhanced usual care, consisting of (1) validation and safety planning for the wife; (2) referrals to provide medical-legal and shelter support; and (3) a brief educational intervention to the husband on alcohol use, based on the World Health Organization’s manual for managing AUD in primary care settings, [74] and a referral to tertiary care mental health (NIMHANS) and addictions treatment center. These will be carried out within two months of the baseline assessment by a trained nurse practitioner who is not involved in delivering the intervention.

### Outcomes

#### Primary.

Primary outcome measures include: 1) mean scores on the Indian Family Violence and Control Scale (IFVCS) [41] and 2) mean scores on the Alcohol Use Disorders Identification Test (AUDIT) [77,78].

#### Secondary.

Key secondary outcome measures include number of days (0–7) with a negative breathalyzer test over a one-week period prior to each follow-up interview and scores on the Communications Patterns Questionnaire (CPQ) [79] to assess couples’ communication and related behaviors during and after conflict interactions. A complete list of measures is included in Fig 1 and Table 2. All scales have been validated for use in India.

**Table 2:** Outcome variables, measures and intervention strategies.

Key Model Variables	Husband or Wife	Description of Scales	Intervention Strategies
**Demographics** *BL*	H, W	Age, years married, arranged marriage, children, living situation, education, religion, and income	
**Self-Efficacy** *BL, 6mo, 12mo*	H	Situational Confidence Questionnaire (SCQ) [80]: eight items assessing self-efficacy in resisting urge to drink heavily in alcohol-related situations	• Elicit participant-specific motivations, fears, and ambivalence and evoke change talk to facilitate behavior change (MI) • Collaboratively develop plan for change and problem-solve barriers (MI) • Provide coping skills to resist drinking urges (BCT)
**Motivation to Change** *BL, 6mo,12mo*	H	12-item Readiness to Change questionnaire for use in brief, opportunistic interventions among excessive drinkers [81]	
**Coping Skills** *BL, 6mo, 12mo*	H, W	Brief COPE [82]: a 28-item scale that assesses the use of different coping strategies for challenging situations	
**Alcohol Consumption and Dependence** *Primary and secondary outcome: quarterly*	H	Alcohol Use Disorders Identification Test (AUDIT) [78,83]:10 items measuring alcohol consumption, drinking behaviors and alcohol-related problems Number of negative breathalyzer tests in 7 consecutive days prior to quantitative interview	• Facilitate increased awareness of drinking patterns (MI) • Elicit negative consequences of alcohol use (MI) • Support reduced alcohol use (MI) • Develop daily trust contract for accountability to maintain sobriety (BCT) • Review positive reasons for sobriety, distracting activities and positive self-talk (BCT) • Identify interpersonal triggers and ways to avoid alcohol use (BCT)
**Alcohol Expectancies** *BL, 6mo, 12mo*	H	Alcohol Outcome Expectancies questionnaire: 38 items assessing positive and negative alcohol use expectancies [84,85]	
**Context of Drinking** *BL, 6mo, 12mo*	H	Five items measuring social and interpersonal contexts of alcohol consumption [86,87]	
**Context of IPV** *BL, 6mo, 12mo*	H, W	14 items measuring social and interpersonal contexts of IPV [86–88]	• Promote positive relationship dynamics, provide safe discussion space to explore conflict, violence, and relationship impact (BCT)
**Attitudes Towards Violence** *BL*	H	Gender-Equitable Men (GEM) scale, IPV subscale: 4 items assessing attitudes towards gender norms in intimate relationships [10,89]	
**Intimate Partner Violence** *Primary Outcome: BL, 6mo, 12mo*	W	Indian Family Violence and Control Scale (IFVCS): 63 items to measuring control, psychological, physical, and sexual violence/coercion [40]	• Link violence to stressors and drinking patterns (BCT)
**Marital Relationship** *BL, 6mo, 12mo*	H, W	17 items assessing the frequency of positive interactions between spouses in the past month [36,90,91]	• Build skills to improve relationship dynamics (BCT)
**Communication Skills** *Secondary outcome:* *quarterly*	H, W	Communications Patterns Questionnaire (CPQ): 35 items assessing couple’s behavior patterns during and after conflict interactions [79]	• Teach active listening skills (BCT) • Promote healthy communication (BCT)
**Anger Management** *BL, 6mo, 12mo*	H	Clinical Anger Scale (CAS): 21 items assessing clinical anger and treatment progress [92]	• Promote emotion regulation through improved communication (BCT)
**Family and Financial Stress** *BL, 6mo, 12mo*	H, W	Chronic Strain Checklist (CSC): 23 items assessing family, marital, financial and work-related stressors [93,94]	• Identify life stressors and their relation to relationship problems and drinking patterns (MI, BCT)
**Decision Making Power** *BL, 6mo, 12mo*	H, W	Sexual Relationship Power Scale (SRPS): 9 items assessing decision-making power in the relationship [95] and three additional items on financial decisions	• Facilitate joint financial and general decision-making (BCT)
**Stigma** *BL, 6mo, 12mo*	H, W	• Alcohol-adapted Perceived Devaluation-Discrimination scale (PDD), adapted: 13 items assessing perceived alcohol stigma [96] • IPV Stigma Scale: 20 items assessing or internalized, anticipated, and perpetrator stigma, and isolation [97]	• Increase knowledge and identification of consequences of stigma related to alcohol use and interpersonal violence (BCT)
**Anxiety** *BL, 6mo, 12mo* **Depression** *quarterly*	H, W	• Generalized Anxiety Disorder Scale (GAD-7): 7 items assessing DSM-specific anxiety symptoms [98] • Personal Health Questionnaire (PHQ-9): 9 items assessing DSM-specific depressive symptoms [99]	• Improve coping to reduce stress and reduce drinking (MI, BCT)
**Gender Norms** *BL*	H	Gender-Equitable Men (GEM): 23 items assessing attitudes toward gender-equitable and inequitable norms [89,100]	Theoretical Moderators
**Impulsivity** *BL*	H	Barratt Impulsiveness Scale, short form (BIS-15): 15 items assessing personality and behavioral constructs of impulsivity [101]	
**Contraception** *BL*	H, W	8 items assessing contraceptive use with spouse (for women) and with spouse and other sexual partners (for men).	

H = Husband; W = Wife; BL = Baseline; 6mo = 6 months; 12mo = 12 months.

### Randomization & study blinding

The study statistician will a priori randomize 400 study ID numbers to one of two arms in a 1:1 ratio. The list will be shared with the intervention coordinator. When a couple is enrolled, the intervention coordinator will consult this list and informs the nurse-counselors of the assignment using a password-protected online log. During the baseline visit, the husband will be shown how to use the breathalyzer by a male assessment team member and asked to blow into it every day for seven days. The following week, the assigned nurse-counselor will contact the couple to inform them of their assigned group and schedule the first session. Assessment team members will be blinded to group assignment to minimize performance bias.

Following the final 12-month follow-up assessment, control arm couples will be offered four sessions of the intervention, if interested. After intervention completion, the study statistician will randomly select couples stratified by complete vs. incomplete intervention attendance to participate in qualitative IDIs. Every two months, the study statistician will select approximately 20 percent of the number of couples available that round. This process balances random selection with ensuring couples can accurately recall their intervention experience. 

### Data collection

Data collection will take place in person at five timepoints: baseline (pre-intervention) and three-, six-, nine-, and 12-month follow-up. Study staff will solicit support from ASHAs, PHC medical officers, and community-based non-governmental organizations who work with women and are well-known to PHC staff. After informed consent, research staff will administer baseline questionnaires using REDCap on study tablets. The husband will receive a reminder via text message to use the breathalyzer every day and to send a photo using it the subsequent week. At all remaining quarterly follow-up outcome assessments, the seven-day breathalyzer assessment will precede questionnaire administration. Breathalyzers will be dropped off and collected by members of the assessment team.

To assess the hypothesized mediators and mechanisms of change, IDIs will be conducted with the qualitative subsample at two time points: within three months after the end of the intervention and upon completion of the 12-month follow-up. Spouses will be interviewed separately by trained qualitative interviewers, with interviews audio-recorded, transcribed in the local language (Kannada), and translated to English for analyses.

### Participant safety

One potential risk in IPV intervention research is an increase in IPV. No increases in IPV were noted in our pilot study in India. [37] The intervention approach entails presenting the intervention to participants as a communication- and relationship-enhancing intervention and not an IPV intervention directly. [102] This framing is used to increase intervention acceptability and reduce stigma related to IPV and participation in substance use programs and mental health services. We will not include those with severe alcohol dependence or severe IPV because the intervention is not designed for those cases. All staff will be routinely trained in issues related to IPV and alcohol use and associated stigma as well as in safety procedures to enable them to respond to circumstances of distress. To address potential risks, we will refer participants who experience exacerbation in violence to local, integrated services with whom we have close contact. At any point in the study, if an enrolled wife expresses a desire for further support for the violence, we will refer her to legal and/or integrated support organizations, a few of which are headed by members of Harmony’s Community Advisory Board. Participants will be assessed at each follow-up for clinically significant depressive symptoms and suicide risk and offered referrals to the psychiatrist or the nearest hospital for consultation and care in case of moderate or severe risk of suicide or harm to self (as assessed by the study clinical psychologist). The Data Safety & Monitoring Board will closely review safety data. Our Data and Safety Monitoring Plan (DSMP) includes a detailed plan to monitor and mitigate risk. A consultant with expertise in gender-related health issues conducts regular gender sensitization and safety workshops with the study staff and will be available to the team for ad-hoc consultations and support.

### Data analysis

#### Quantitative.

We will perform intention-to-treat (ITT) analyses to compare the treatment and control groups on the two primary outcomes: 1) mean overall AUDIT score as the measure of alcohol use; and 2) mean overall IFVCS score as the measure of IPV. We hypothesize that the treatment group will show a lower mean AUDIT score and a lower mean IFVCS score than the control group participants at 12-month follow-up. We will analyze this via a linear regression model for each outcome, in which we will control for the baseline outcome score and any relevant covariates for which the treatment groups might differ at baseline, despite randomization. A multilevel version of the models will be used for the longitudinal analyses, with repeated measures nested within participants and a random intercept for participants. The treatment arm*wave interaction will serve as the test of the intervention effect. Since each of these outcomes will only be collected from one of the spouses (alcohol use from the male and IPV from the female),combining/controlling for multiple reports within the couple is not necessary. If despite our extensive, proven-effective retention efforts, there is a substantial amount of missing data, we will use multiple imputations, assuming data are missing at random, including socio-demographic and other variables as auxiliary variables as appropriate, to perform sensitivity analyses of the original results [103].

Secondary analyses will consist of similar ITT linear regressions as for IFVCS above for the secondary outcomes of mean CPQ and breathalyzer scores. We hypothesize that post-intervention, the treatment group will show more positive and less negative communication patterns than the control group. We also expect that intervention participants will have lower post-intervention breathalyzer scores, on average, than the control group, reflecting reductions in frequency and quantity of alcohol use. Like the primary outcomes, the breathalyzer data is only available from one spouse, but CPQ data will be collected from both spouses. CPQ subscale scores will be constructed based on each spouse’s report separately, and agreement assessed via intraclass correlations. The treatment effect will be explored using husband and wife reports as the outcome variable in separate models to allow a differential effect of the treatment on mean CPQ subscale scores as reported by each partner in the couple [104].

Mediation analyses will assess if the effect of the intervention on the primary outcomes (AUD and IPV) was mediated by change in the theorized drivers and intermediate variables (e.g. self-efficacy, anger management, coping skills), by assessing the effect of the intervention on the mediators and of both the mediators and the intervention on the outcome. This will be tested via structural equation models (SEM) with bootstrapped confidence intervals for the indirect effects [105]. SEM can also accommodate dyadic analyses in which variables reported by both spouses are included in the same model and estimate both ‘actor’ and ‘partner’ effects (e.g. effect of change in wife’s report of marital relationship on change in wife’s CPQ score and on change in husband’s CPQ score, respectively) [106]. We will also examine whether personality factors, such as impulsivity and gender norms, moderate the intervention effect. Moderation will be assessed by including an interaction between the potential moderators and the intervention variable in the regression model. Based on the global literature, including studies from India, [107–112] on impulsivity, gender equity attitudes, alcohol and IPV, we hypothesize that the intervention impact will be less among men who score highly on impulsivity and have lower score on gender equitable norms. Other secondary, preliminary analyses will rerun the ITT models above, but with intervention dose (number of sessions attended) added to the models.

#### Qualitative.

Analyses of the 160 transcripts (interviews with each of the 40 couples at two time points: immediately post-intervention and at 12 months) will use a thematic approach to coding and summarizing using Dedoose qualitative analysis software [113]. A preliminary codebook will be developed from interview guides and rapid analysis content, then refined after all analysts code a set of transcripts independently and resolve discrepancies through consensus. The remaining transcripts will be single-coded, maintaining intercoder reliability of at least 80% throughout the coding process using kappa calculations. Coding memos will be used to summarize and explore the relationship between the constructs of the theory of change framework (e.g., the relationship between intervention processes and hypothesized mechanisms of change, or between mechanisms of change and outcomes). Analysts will also review and analyze coded serial IDI content within each participant’s dataset to look specifically for patterns over time to further understand sustained results.

#### Triangulation of quantitative and qualitative data.

Differences and similarities in the qualitative and quantitative data sets will be triangulated to build a more comprehensive understanding of how the hypothesized changes in mechanisms of action resulted in the intended outcomes and their relative weight in creating change among intervention participants. Both will be examined and presented to understand the relationship between the constructs in the conceptual model and to highlight any factors that may be uniquely identified. These data will inform interpretation of the efficacy findings and next steps for any refinements that may be necessary as the intervention is scaled up.

### Ethics approval

This study was approved by the Institutional Ethics Committee at St. John’s Medical College (IEC/1/829/2021) and the Institutional Review Board at the University of California San Francisco (Protocol 21–34788). All study materials and protocols have been reviewed by corresponding ethics boards in both English and Kannada. The study was approved by the Health Ministry Screening Committee (HMSC), the Indian Council of Medical Research, and the Ministry of Health, Government of India.

### Trial status

The study is currently active and enrolling. Recruitment was initiated in July 2023 and expected to conclude by September 2026. All assessments are anticipated to be completed by May 2027. After final data analysis and the writing of the results, we plan to share the main findings in manuscripts submitted beginning December 2027.

## Discussion

This manuscript outlines the protocol for a randomized controlled trial (RCT) of a couple’s intervention targeting co-occurring spousal alcohol use disorder (AUD) and intimate partner violence (IPV) for 400 couples, delivered by nurse-counselors in Bengaluru, India. The active condition consists of a 10-session, motivational interviewing (MI) and behavioral couples therapy (BCT) intervention delivered to 200 couples, while the control condition consists of a single session of enhanced usual care (n = 200 couples). A secondary aim consists of examining hypothesized mediators and moderators of intervention efficacy using quantitative and qualitative data.

The current Harmony intervention RCT has several strengths. First, it is among few programs that incorporate both partners in an IPV intervention. Many IPV interventions have focused on only women while AUD interventions have only targeted men. This separation places the entirety of burden to effect change in IPV on women who are already experiencing violence. Women-only interventions risk an IPV increase if the husband finds out that his wife has been accessing services for IPV. It is also important to include both partners when they have few options to leave and desire to stay together. Second, the intervention approach (i.e., working with both members of the couple and integrating the intervention into the public health care system) is novel and offers promising sustainability and scalability. Third, integration of BCT and MI will increase the evidence base of interventions for co-occurring AUD and IPV. Furthermore, intervention delivery by trained nurses increases scalability and addresses the ongoing treatment gap between the need for services and provider availability in South Asia and other resource-constrained settings worldwide. Additional strengths include use of an appropriately powered RCT to maximize internal validity, [114] use of culturally validated measures, and a study foundation from prior formative work [36] that found a similar intervention to be safe and acceptable.

Potential limitations and design considerations also accompany the study, including difficulties enrolling and retaining both partners in the intervention. To address this barrier, we have developed a plan to utilize ASHA community health workers who engage in a series of steps to re-engage families. The study team also has substantial experience with engaging vulnerable and marginalized participants in our previous trials in South India as well as in our pilot work [36,115,116]. In addition, the study may benefit from the positive focus on improving relationships and communication.

Like all intervention studies, ours includes potential risks, including the potential for the intervention to inadvertently increase IPV. However, our formative work with a similar population showed no safety concerns or increases in violence following participation [36]. A final risk is the time and cost burden on participants. We estimate the total time for the assessments in each wave to take between 60–90 minutes. Participants will be compensated for their time, transportation, and, if applicable, for lost wages when they miss work to attend sessions. To reduce the impact on the PHC staff, all assessments will be conducted by study interviewers and all intervention components are delivered by a study staff nurse-counselor with the same educational qualifications as the PHC nurses to ensure that the findings are generalizable.

## Conclusion

The Harmony study has potential to develop an evidence base for a novel, scalable, and sustainable intervention that combines MI and BCT to address concurrent AUD and IPV in primary care settings in India. Given that it is community based, it has the potential to be acceptable to couples who are otherwise unlikely to access services for IPV in situations in which few women have a real choice to leave an abusive relationship. If successful, this novel and combined intervention can be scaled up in community-based primary care settings as well as adapted to similar resource-constrained, global settings.

## Supporting information

S1 File Harmony study protocol Minus Logo. (DOCX)

## References

[1] Foran HM, O’Leary KD. Alcohol and intimate partner violence: a meta-analytic review. Clin Psychol Rev. 2008;28(7):1222–34. doi: 10.1016/j.cpr.2008.05.001 18550239

[2] World Health Organization. Violence against women: intimate partner and sexual violence against women: evidence brief. World Health Organization; 2019.

[3] Leonard KE, Quigley BM. Thirty years of research show alcohol to be a cause of intimate partner violence: Future research needs to identify who to treat and how to treat them. Drug Alcohol Rev. 2017;36(1):7–9. doi: 10.1111/dar.12434 27305859

[4] Cafferky BM, Mendez M, Anderson JR, Stith SM. Substance use and intimate partner violence: A meta-analytic review. Psychology of Violence. 2018;8(1):110–31. doi: 10.1037/vio0000074

[5] Leonard KE. Alcohol consumption and escalatory aggression in intoxicated and sober dyads. J Stud Alcohol. 1984;45(1):75–80. doi: 10.15288/jsa.1984.45.75 6700223

[6] Steele CM, Josephs RA. Alcohol myopia. Its prized and dangerous effects. Am Psychol. 1990;45(8):921–33. doi: 10.1037//0003-066x.45.8.921 2221564

[7] Barnett OW, Fagan RW. Alcohol use in male spouse abusers and their female partners. J Fam Viol. 1993;8(1):1–25. doi: 10.1007/bf00986990

[8] Tenkorang EY, Asamoah-Boaheng M, Owusu AY. Intimate Partner Violence (IPV) Against HIV-Positive Women in Sub-Saharan Africa: A Mixed-Method Systematic Review and Meta-Analysis. Trauma Violence Abuse. 2021;22(5):1104–28. doi: 10.1177/1524838020906560 32067599

[9] Thompson MP, Kingree JB. The roles of victim and perpetrator alcohol use in intimate partner violence outcomes. J Interpers Violence. 2006;21(2):163–77. doi: 10.1177/0886260505282283 16368759

[10] Heylen E, Shamban E, Steward WT, Krishnan G, Solomon R, Srikrishnan AK, et al. Alcohol Use and Experiences of Partner Violence Among Female Sex Workers in Coastal Andhra Pradesh, India. Violence Against Women. 2019;25(3):251–73. doi: 10.1177/1077801218778384 29953335 PMC6274613

[11] Wagman JA, Donta B, Ritter J, Naik DD, Nair S, Saggurti N, et al. Husband’s Alcohol Use, Intimate Partner Violence, and Family Maltreatment of Low-Income Postpartum Women in Mumbai, India. J Interpers Violence. 2018;33(14):2241–67. doi: 10.1177/0886260515624235 26802047 PMC6886467

[12] Mukherjee R, Joshi RK. Controlling Behavior and Intimate Partner Violence: A Cross-Sectional Study in an Urban Area of Delhi, India. J Interpers Violence. 2021;36(19–20):NP10831–42. doi: 10.1177/0886260519876720 31561731

[13] Ramadugu S, Jayaram PV, Srivastava K, Chatterjee K, Madhusudan T. Understanding intimate partner violence and its correlates. Ind Psychiatry J. 2015;24(2):172–8. doi: 10.4103/0972-6748.181714 27212823 PMC4866346

[14] Dasgupta A, Silverman J, Saggurti N, Ghule M, Donta B, Battala M, et al. Understanding Men’s Elevated Alcohol Use, Gender Equity Ideologies, and Intimate Partner Violence Among Married Couples in Rural India. Am J Mens Health. 2018;12(4):1084–93. doi: 10.1177/1557988318775844 29779428 PMC6131423

[15] Ler P, Sivakami M, Monárrez-Espino J. Prevalence and Factors Associated With Intimate Partner Violence Among Young Women Aged 15 to 24 Years in India: A Social-Ecological Approach. J Interpers Violence. 2020;35(19–20):4083–116. doi: 10.1177/0886260517710484 29294780

[16] Mathew SS, Goud RB, Pradeep J. Intimate Partner Violence among Ever-married Women Treated for Depression at a Rural Health Center in Bengaluru Urban District. Indian J Community Med. 2019;44(Suppl 1):S70–3. doi: 10.4103/ijcm.IJCM_72_19 31728096 PMC6824182

[17] International Institute for Population Sciences IIPS, ICF. National Family Health Survey (NFHS-4) 2019-21. Mumbai, India: IIPS; 2021.

[18] Nanda P, Gautam A, Verma R. Masculinity, intimate partner violence and son preference in India. New Delhi, India: International Center for Research on Women (ICRW); 2014.

[19] Silverman JG, Decker MR, Saggurti N, Balaiah D, Raj A. Intimate partner violence and HIV infection among married Indian women. JAMA. 2008;300(6):703–10. doi: 10.1001/jama.300.6.703 18698068

[20] Chowdhary N, Patel V. The effect of spousal violence on women’s health: findings from the Stree Arogya Shodh in Goa, India. J Postgrad Med. 2008;54(4):306–12. doi: 10.4103/0022-3859.43514 18953151

[21] Das S, Bapat U, Shah More N, Alcock G, Joshi W, Pantvaidya S, et al. Intimate partner violence against women during and after pregnancy: a cross-sectional study in Mumbai slums. BMC Public Health. 2013;13:817. doi: 10.1186/1471-2458-13-817 24015762 PMC3846679

[22] Stephens-Lewis D, Johnson A, Huntley A, Gilchrist E, McMurran M, Henderson J, et al. Interventions to Reduce Intimate Partner Violence Perpetration by Men Who Use Substances: A Systematic Review and Meta-Analysis of Efficacy. Trauma Violence Abuse. 2021;22(5):1262–78. doi: 10.1177/1524838019882357 31711372 PMC8649458

[23] Easton CJ, Mandel D, Babuscio T, Rounsaville BJ, Carroll KM. Differences in treatment outcome between male alcohol dependent offenders of domestic violence with and without positive drug screens. Addict Behav. 2007;32(10):2151–63. doi: 10.1016/j.addbeh.2007.01.031 17367953

[24] Mbilinyi LF, Neighbors C, Walker DD, Roffman RA, Zegree J, Edleson J, et al. A Telephone Intervention for Substance-Using Adult Male Perpetrators of Intimate Partner Violence. Res Soc Work Pract. 2011;21(1):43–56. doi: 10.1177/1049731509359008 22754270 PMC3384695

[25] Easton CJ, Crane CA, Mandel D. A Randomized Controlled Trial Assessing the Efficacy of Cognitive Behavioral Therapy for Substance-Dependent Domestic Violence Offenders: An Integrated Substance Abuse-Domestic Violence Treatment Approach (SADV). J Marital Fam Ther. 2018;44(3):483–98. doi: 10.1111/jmft.12260 29108096

[26] Kraanen FL, Vedel E, Scholing A, Emmelkamp PMG. The comparative effectiveness of Integrated treatment for Substance abuse and Partner violence (I-StoP) and substance abuse treatment alone: a randomized controlled trial. BMC Psychiatry. 2013;13:189. doi: 10.1186/1471-244X-13-189 24059784 PMC3716952

[27] Satyanarayana VA, Nattala P, Selvam S, Pradeep J, Hebbani S, Hegde S, et al. Integrated Cognitive Behavioral Intervention Reduces Intimate Partner Violence Among Alcohol Dependent Men, and Improves Mental Health Outcomes in their Spouses: A Clinic Based Randomized Controlled Trial from South India. J Subst Abuse Treat. 2016;64:29–34. doi: 10.1016/j.jsat.2016.02.005 26965174

[28] Murphy CM, Ting LA, Jordan LC, Musser PH, Winters JJ, Poole GM, et al. A randomized clinical trial of motivational enhancement therapy for alcohol problems in partner violent men. J Subst Abuse Treat. 2018;89:11–9. doi: 10.1016/j.jsat.2018.03.004 29706170 PMC5930382

[29] Stuart GL, Shorey RC, Moore TM, Ramsey SE, Kahler CW, O’Farrell TJ, et al. Randomized clinical trial examining the incremental efficacy of a 90-minute motivational alcohol intervention as an adjunct to standard batterer intervention for men. Addiction. 2013;108(8):1376–84. doi: 10.1111/add.12142 23414253 PMC3681834

[30] Giusto A, Puffer E. A systematic review of interventions targeting men’s alcohol use and family relationships in low- and middle-income countries. Glob Ment Health (Camb). 2018;5:e10. doi: 10.1017/gmh.2017.32 29632682 PMC5885490

[31] Heise L. Working with couples: promise or peril?. Rio de Janeiro: Sexual Violence Research Initiative (SVRI); 2017.

[32] Jones D, Weiss SM, Arheart K, Cook R, Chitalu N. Implementation of HIV prevention interventions in resource limited settings: the partner project. J Community Health. 2014;39(1):151–8. doi: 10.1007/s10900-013-9753-2 23963855 PMC3880645

[33] Minnis AM, Doherty IA, Kline TL, Zule WA, Myers B, Carney T, et al. Relationship power, communication, and violence among couples: results of a cluster-randomized HIV prevention study in a South African township. Int J Womens Health. 2015;7:517–25. doi: 10.2147/IJWH.S77398 25999767 PMC4435250

[34] Raj A, Ghule M, Ritter J, Battala M, Gajanan V, Nair S, et al. Cluster Randomized Controlled Trial Evaluation of a Gender Equity and Family Planning Intervention for Married Men and Couples in Rural India. PLoS One. 2016;11(5):e0153190. doi: 10.1371/journal.pone.0153190 27167981 PMC4864357

[35] Stern E, Nyiratunga R. A Process Review of the Indashyikirwa Couples Curriculum to Prevent Intimate Partner Violence and Support Healthy, Equitable Relationships in Rwanda. Social Sciences. 2017;6(2):63. doi: 10.3390/socsci6020063

[36] Hartmann M, Datta S, Browne EN, Appiah P, Banay R, Caetano V, et al. A Combined Behavioral Economics and Cognitive Behavioral Therapy Intervention to Reduce Alcohol Use and Intimate Partner Violence Among Couples in Bengaluru, India: Results of a Pilot Study. J Interpers Violence. 2021;36(23–24):NP12456–80. doi: 10.1177/0886260519898431 31959030

[37] Fals-Stewart W, Clinton-Sherrod M. Treating intimate partner violence among substance-abusing dyads: The effect of couples therapy. Professional Psychology: Research and Practice. 2009;40(3):257–63. doi: 10.1037/a0012708

[38] Fulu E, Kerr-Wilson A, Lang J, Gibbs A, Jacobson J, Jewkes R. What works to prevent violence against women and girls. Evidence Review of interventions to prevent violence against women and girls Pretoria: Medical Research Council; 2014 Jun;1:580–1589.

[39] Ruff S, McComb JL, Coker CJ, Sprenkle DH. Behavioral couples therapy for the treatment of substance abuse: a substantive and methodological review of O’Farrell, Fals-Stewart, and colleagues’ program of research. Fam Process. 2010;49(4):439–56. doi: 10.1111/j.1545-5300.2010.01333.x 21083548

[40] O’Farrell TJ, Fals-Stewart W. Behavioral couples therapy for alcoholism and drug abuse. J Subst Abuse Treat. 2000;18(1):51–4. doi: 10.1016/s0740-5472(99)00026-4 10636606 PMC3215582

[41] Kalokhe AS, Stephenson R, Kelley ME, Dunkle KL, Paranjape A, Solas V, et al. The Development and Validation of the Indian Family Violence and Control Scale. PLoS One. 2016;11(1):e0148120. doi: 10.1371/journal.pone.0148120 26824611 PMC4732749

[42] Frost H, Campbell P, Maxwell M, O’Carroll RE, Dombrowski SU, Williams B, et al. Effectiveness of Motivational Interviewing on adult behaviour change in health and social care settings: A systematic review of reviews. PLoS One. 2018;13(10):e0204890. doi: 10.1371/journal.pone.0204890 30335780 PMC6193639

[43] Joseph J, Basu D. Efficacy of Brief Interventions in Reducing Hazardous or Harmful Alcohol Use in Middle-Income Countries: Systematic Review of Randomized Controlled Trials. Alcohol Alcohol. 2017;52(1):56–64. doi: 10.1093/alcalc/agw054 27567270

[44] Joseph J, Basu D, Dandapani M, Krishnan N. Are nurse-conducted brief interventions (NCBIs) efficacious for hazardous or harmful alcohol use? A systematic review. Int Nurs Rev. 2014;61(2):203–10. doi: 10.1111/inr.12096 24645911

[45] Tsai M-C, Tsai Y-F, Hwang F-M, Liu C-Y. Effectiveness of a brief intervention for managing hazardous drinking problems of inpatients in Taiwan. J Adv Nurs. 2011;67(9):2038–46. doi: 10.1111/j.1365-2648.2011.05623.x 21827531

[46] Cherpitel CJ, Moskalewicz J, Swiatkiewicz G, Ye Y, Bond J. Screening, brief intervention, and referral to treatment (SBIRT) in a Polish emergency department: three-month outcomes of a randomized, controlled clinical trial. J Stud Alcohol Drugs. 2009;70(6):982–90. doi: 10.15288/jsad.2009.70.982 19895777 PMC2776128

[47] Noknoy S, Rangsin R, Saengcharnchai P, Tantibhaedhyangkul U, McCambridge J. RCT of effectiveness of motivational enhancement therapy delivered by nurses for hazardous drinkers in primary care units in Thailand. Alcohol & Alcoholism. 2010;45(3):263–70. 20236990 10.1093/alcalc/agq013 PMC4394355

[48] Mertens JR, Ward CL, Bresick GF, Broder T, Weisner CM. Effectiveness of nurse-practitioner-delivered brief motivational intervention for young adult alcohol and drug use in primary care in South Africa: a randomized clinical trial. Alcohol Alcohol. 2014;49(4):430–8. doi: 10.1093/alcalc/agu030 24899076 PMC4060738

[49] Kamal K, Sunita S, Karobi D, Abhishek G. Nurse-Delivered Screening and Brief Intervention Among College Students with Hazardous Alcohol Use: A Double-Blind Randomized Clinical Trial from India. Alcohol Alcohol. 2020;55(3):284–90. doi: 10.1093/alcalc/agaa014 32103254

[50] Halcomb EJ, Furler JS, Hermiz OS, Blackberry ID, Smith JP, Richmond RL, et al. Process evaluation of a practice nurse-led smoking cessation trial in Australian general practice: views of general practitioners and practice nurses. Fam Pract. 2015;32(4):468–73. doi: 10.1093/fampra/cmv041 26024924

[51] Steffenssen MCW, Klemp I, Nielsen M, Bakholdt V, Thomsen J-B, Sørensen JA. Nurse-led counselling and replacement therapy is effective for smoking cessation in oral cancer patients. Eur J Plast Surg. 2017;40(6):593–6. doi: 10.1007/s00238-017-1314-y

[52] Channon S, Bekkers M-J, Sanders J, Cannings-John R, Robertson L, Bennert K, et al. Motivational interviewing competencies among UK family nurse partnership nurses: a process evaluation component of the building blocks trial. BMC Nurs. 2016;15:55. doi: 10.1186/s12912-016-0176-0 27660554 PMC5029038

[53] van den Wijngaart LS, Sieben A, van der Vlugt M, de Leeuw FE, Bredie SJH. A nurse-led multidisciplinary intervention to improve cardiovascular disease profile of patients. West J Nurs Res. 2015;37(6):705–23. doi: 10.1177/0193945914533427 24823969

[54] McKenzie K, Chang Y-P. The effect of nurse-led motivational interviewing on medication adherence in patients with bipolar disorder. Perspect Psychiatr Care. 2015;51(1):36–44. doi: 10.1111/ppc.12060 24433493

[55] DiIorio C, McCarty F, Resnicow K, McDonnell Holstad M, Soet J, Yeager K, et al. Using motivational interviewing to promote adherence to antiretroviral medications: a randomized controlled study. AIDS Care. 2008;20(3):273–83. doi: 10.1080/09540120701593489 18351473 PMC3103182

[56] Lakshmana G. Efficacy of Combination of Motivational Interviewing and Cognitive Behavior Intervention with Substance Abuse Street Adolescents in India: A Randomized Control Study. Journal of Social Work Practice in the Addictions. 2016;16(4):337–57. doi: 10.1080/1533256x.2016.1235414

[57] Mehrotra K, Chand P, Bandawar M, Rao Sagi M, Kaur S, G A, et al. Effectiveness of NIMHANS ECHO blended tele-mentoring model on Integrated Mental Health and Addiction for counsellors in rural and underserved districts of Chhattisgarh, India. Asian J Psychiatr. 2018;36:123–7. doi: 10.1016/j.ajp.2018.07.010 30086513

[58] Nadkarni A, Weobong B, Weiss HA, McCambridge J, Bhat B, Katti B, et al. Counselling for Alcohol Problems (CAP), a lay counsellor-delivered brief psychological treatment for harmful drinking in men, in primary care in India: a randomised controlled trial. The Lancet. 2017;389(10065):186–95. 10.1016/S0140-6736(16)31590-2 PMC5236065 27988144

[59] Bandura A. Health promotion by social cognitive means. Health Educ Behav. 2004;31(2):143–64. doi: 10.1177/1090198104263660 15090118

[60] Britt E, Hudson SM, Blampied NM. Motivational interviewing in health settings: a review. Patient Education and Counseling. 2004;53(2):147–55. 15140454 10.1016/S0738-3991(03)00141-1

[61] Karakurt G, Whiting K, van Esch C, Bolen SD, Calabrese JR. Couples Therapy for Intimate Partner Violence: A Systematic Review and Meta-Analysis. J Marital Fam Ther. 2016;42(4):567–83. doi: 10.1111/jmft.12178 27377617 PMC5050084

[62] Miller WR, Rollnick S. Motivational interviewing: Helping people change. Guilford press; 2012.

[63] Bell KM, Naugle AE. Intimate partner violence theoretical considerations: moving towards a contextual framework. Clin Psychol Rev. 2008;28(7):1096–107. doi: 10.1016/j.cpr.2008.03.003 18430501

[64] O’Leary SG, Slep AMS. Precipitants of partner aggression. J Fam Psychol. 2006;20(2):344–7. doi: 10.1037/0893-3200.20.2.344 16756412

[65] Mihalic SW, Elliott D. A Social Learning Theory Model of Marital Violence. Domestic Violence. Routledge; 2017. p. 303–29. doi: 10.4324/9781315264905-22

[66] Riggs DS, O’Leary KD. Aggression between heterosexual dating partners: An examination of a causal model of courtship aggression. Journal of Interpersonal Violence. 1996;11(4):519–40.

[67] Johnson H, Ollus N, Nevala S. Violence Against Women: An International Perspective. New York: Springer; 2008.

[68] Bush K, Kivlahan DR, McDonell MB, Fihn SD, Bradley KA. The AUDIT alcohol consumption questions (AUDIT-C): an effective brief screening test for problem drinking. Ambulatory Care Quality Improvement Project (ACQUIP). Alcohol Use Disorders Identification Test. Arch Intern Med. 1998;158(16):1789–95. doi: 10.1001/archinte.158.16.1789 9738608

[69] Stockwell T, Murphy D, Hodgson R. The severity of alcohol dependence questionnaire: its use, reliability and validity. Br J Addict. 1983;78(2):145–55. doi: 10.1111/j.1360-0443.1983.tb05502.x 6135435

[70] Sullivan JT, Sykora K, Schneiderman J, Naranjo CA, Sellers EM. Assessment of alcohol withdrawal: the revised clinical institute withdrawal assessment for alcohol scale (CIWA-Ar). Br J Addict. 1989;84(11):1353–7. doi: 10.1111/j.1360-0443.1989.tb00737.x 2597811

[71] Cohen J. Statistical power analysis for the behavioral sciences. Routledge; 2013.

[72] Tan CJ, Shufelt T, Behan E, Chantara J, Koomsri C, Gordon AJ, et al. Comparative effectiveness of psychosocial interventions in adults with harmful use of alcohol: a systematic review and network meta-analysis. Addiction. 2023;118(8):1414–29. doi: 10.1111/add.16187 36905310

[73] Katzman R, Brown T, Fuld P, Peck A, Schechter R, Schimmel H. Validation of a short Orientation-Memory-Concentration Test of cognitive impairment. Am J Psychiatry. 1983;140(6):734–9. doi: 10.1176/ajp.140.6.734 6846631

[74] World Health Organization. WHO alcohol brief intervention training manual for primary care. World Health Organization; 2017.

[75] Moyers TB, Martin T, Manuel JK, Miller WR, Ernst D. Revised global scales: Motivational interviewing treatment integrity 3.1. 1 (MITI 3.1. 1). Albuquerque, NM: University of New Mexico; 2010.

[76] Rege S, Bhate-Deosthali P, Ravindran TKS. Violence against women and girls: Understanding responses and approached in the Indian health sector. Routledge. 2021.

[77] Chibber KS, Krishnan S, Minkler M. Physician practices in response to intimate partner violence in southern India: insights from a qualitative study. Women Health. 2011;51(2):168–85. doi: 10.1080/03630242.2010.550993 21476176 PMC3098281

[78] Saunders JB, Aasland OG, Babor TF, De la Fuente JR, Grant M. Development of the alcohol use disorders identification test (AUDIT): WHO collaborative project on early detection of persons with harmful alcohol consumption‐II. Addiction. 1993;88(6):791–804. 8329970 10.1111/j.1360-0443.1993.tb02093.x

[79] Heavey CL, Larson BM, Zumtobel DC, Christensen A. The Communication Patterns Questionnaire: The Reliability and Validity of a Constructive Communication Subscale. Journal of Marriage and the Family. 1996;58(3):796. doi: 10.2307/353737

[80] Annis H, Graham JM. Situational confidence questionnaire (SCQ): User’s guide. Addiction Research Foundation. 1988.

[81] Rollnick S, Heather N, Gold R, Hall W. Development of a short “readiness to change” questionnaire for use in brief, opportunistic interventions among excessive drinkers. Br J Addict. 1992;87(5):743–54. doi: 10.1111/j.1360-0443.1992.tb02720.x 1591525

[82] Gillespie-Smith K, Goodall K, McConachie D, Van Herwegen J, Crawford H, Ballantyne C, et al. A longitudinal study looking at the impact of COVID-19 restrictions and transitions on psychological distress in caregivers of children with intellectual disabilities in the UK. JCPP Advances. 2024;:e12261. doi: 10.1111/jcpp.12261 PMC12446709 40979737

[83] Endsley P, Weobong B, Nadkarni A. Psychometric properties of the AUDIT among men in Goa, India. Asian J Psychiatr. 2017;29:54–8. doi: 10.1016/j.ajp.2017.03.006 29061428 PMC5650630

[84] Leigh BC, Stacy AW. Alcohol outcome expectancies: Scale construction and predictive utility in higher order confirmatory models. Psychological Assessment. 1993;5(2):216–29. doi: 10.1037/1040-3590.5.2.216

[85] Steward WT, Satyanarayana VA, Heylen E, Srikrishnan AK, Vasudevan CK, Krishnan G, et al. Alcohol use, expectancies and HIV-related sexual risk: a cross-sectional survey of male migrant workers in South India. AIDS Care. 2018;30(5):656–62. doi: 10.1080/09540121.2017.1394964 29084445 PMC5860933

[86] Heravian A, Solomon R, Krishnan G, Vasudevan CK, Krishnan AK, Osmand T, et al. Alcohol consumption patterns and sexual risk behavior among female sex workers in two South Indian communities. Int J Drug Policy. 2012;23(6):498–504. doi: 10.1016/j.drugpo.2012.03.005 22608567 PMC3454864

[87] Rodríguez DC, Krishnan AK, Kumarasamy N, Krishnan G, Solomon D, Johnson S, et al. Two sides of the same story: alcohol use and HIV risk taking in South India. AIDS Behav. 2010;14 Suppl 1(1):S136-46. doi: 10.1007/s10461-010-9722-z 20544382 PMC2900584

[88] Heylen E, Shamban E, Steward WT, Krishnan G, Solomon R, Srikrishnan AK, et al. Alcohol Use and Experiences of Partner Violence Among Female Sex Workers in Coastal Andhra Pradesh, India. Violence Against Women. 2019;25(3):251–73. doi: 10.1177/1077801218778384 29953335 PMC6274613

[89] Pulerwitz J, Barker G. Measuring attitudes toward gender norms among young men in Brazil: development and psychometric evaluation of the GEM scale. Men and masculinities. 2008;10(3):322–38.

[90] Shah A. Clinical validity of Marital Quality Scale. Nimhans Journal. 1995.

[91] Johnson PR, Britto C, Sudevan KJ, Bosco A, Sreedaran P, Ashok MV. Resilience in Wives of persons with Alcoholism: An Indian exploration. Indian J Psychiatry. 2018;60(1):84–9. doi: 10.4103/psychiatry.IndianJPsychiatry_271_14 29736068 PMC5914269

[92] Snell WE Jr, Gum S, Shuck RL, Mosley JA, Hite TL. The Clinical Anger Scale: preliminary reliability and validity. J Clin Psychol. 1995;51(2):215–26. doi: 10.1002/1097-4679(199503)51:2<215::aid-jclp2270510211>3.0.co;2-z 7797645

[93] Rao KG. Personality and coping behaviour in relation to stressful life events. Bangalore (India): Bangalore University; 1986.

[94] Rao K, Bhaskaran SA, Subbakrishna DK. Support utilization in the context of chronic strains. Family Therapy. 2001;28(3):143.

[95] Pulerwitz J, Gortmaker SL, DeJong W. Measuring Sexual Relationship Power in HIV/STD Research. Sex Roles. 2000;42(7–8):637–60. doi: 10.1023/a:1007051506972

[96] Glass JE, Kristjansson SD, Bucholz KK. Perceived alcohol stigma: factor structure and construct validation. Alcohol Clin Exp Res. 2013;37 Suppl 1(0 1):E237-46. doi: 10.1111/j.1530-0277.2012.01887.x 22758603 PMC3893041

[97] Crowe A, Overstreet NM, Murray CE. The Intimate Partner Violence Stigma Scale: Initial Development and Validation. J Interpers Violence. 2021;36(15–16):7456–79. doi: 10.1177/0886260519834095 30866696

[98] Spitzer RL, Kroenke K, Williams JB, Löwe B. A brief measure for assessing generalized anxiety disorder: the GAD-7. Archives of Internal Medicine. 2006;166(10):1092–7. 16717171 10.1001/archinte.166.10.1092

[99] Kroenke K, Spitzer RL, Williams JB. The PHQ-9: validity of a brief depression severity measure. J Gen Intern Med. 2001;16(9):606–13. doi: 10.1046/j.1525-1497.2001.016009606.x 11556941 PMC1495268

[100] Singh K, Verma R, Barker G. Making women count: an annual publication on gender and evaluation. New Delhi: UN Women; 2013.

[101] Spinella M. Normative data and a short form of the Barratt Impulsiveness Scale. Int J Neurosci. 2007;117(3):359–68. doi: 10.1080/00207450600588881 17365120

[102] World Health Organization. Ethical and safety recommendations for intervention research on violence against women: building on lessons from the WHO publication putting women first: ethical and safety recommendations for research on domestic violence against women. World Health Organization; 2016.

[103] Cro S, Morris TP, Kenward MG, Carpenter JR. Sensitivity analysis for clinical trials with missing continuous outcome data using controlled multiple imputation: a practical guide. Statistics in Medicine. 2020;39(21):2815–42. 32419182 10.1002/sim.8569

[104] Schumm JA, O’Farrell TJ, Kahler CW, Murphy MM, Muchowski P. A randomized clinical trial of behavioral couples therapy versus individually based treatment for women with alcohol dependence. J Consult Clin Psychol. 2014;82(6):993–1004. doi: 10.1037/a0037497 25045910 PMC4244232

[105] Preacher KJ, Hayes AF. Asymptotic and resampling strategies for assessing and comparing indirect effects in multiple mediator models. Behav Res Methods. 2008;40(3):879–91. doi: 10.3758/brm.40.3.879 18697684

[106] Ledermann T, Kenny DA. Analyzing dyadic data with multilevel modeling versus structural equation modeling: A tale of two methods. J Fam Psychol. 2017;31(4):442–52. doi: 10.1037/fam0000290 28165269

[107] Poulose B, Srinivasan K. High risk behaviours following alcohol use in alcohol dependent men. Indian J Med Res. 2009;129(4):376–81. 19535831

[108] Leone RM, Crane CA, Parrott DJ, Eckhardt CI. Problematic drinking, impulsivity, and physical IPV perpetration: A dyadic analysis. Psychol Addict Behav. 2016;30(3):356–66. doi: 10.1037/adb0000159 26828640 PMC4877202

[109] Mulawa MI, Reyes HLM, Foshee VA, Halpern CT, Martin SL, Kajula LJ, et al. Associations Between Peer Network Gender Norms and the Perpetration of Intimate Partner Violence Among Urban Tanzanian Men: a Multilevel Analysis. Prev Sci. 2018;19(4):427–36. doi: 10.1007/s11121-017-0835-8 28849338 PMC5832502

[110] Reed E, Donta B, Dasgupta A, Ghule M, Battala M, Nair S, et al. Household Debt and Relation to Intimate Partner Violence and Husbands’ Attitudes Toward Gender Norms: A Study Among Young Married Couples in Rural Maharashtra, India. Public Health Rep. 2015;130(6):664–71. doi: 10.1177/003335491513000616 26556938 PMC4612175

[111] Satyanarayana VA, Hebbani S, Hegde S, Krishnan S, Srinivasan K. Two sides of a coin: Perpetrators and survivors perspectives on the triad of alcohol, intimate partner violence and mental health in South India. Asian J Psychiatr. 2015;15:38–43. doi: 10.1016/j.ajp.2015.04.014 26001901

[112] Sikweyiya Y, Addo-Lartey AA, Alangea DO, Dako-Gyeke P, Chirwa ED, Coker-Appiah D, et al. Patriarchy and gender-inequitable attitudes as drivers of intimate partner violence against women in the central region of Ghana. BMC Public Health. 2020;20:1–1. 32404153 10.1186/s12889-020-08825-z PMC7222313

[113] Dedoose. Dedoose Version 8.0. 35. Web application for managing, analyzing, and presenting qualitative and mixed method research data. 2018.

[114] Vittinghoff E, Sen S, McCulloch CE. Sample size calculations for evaluating mediation. Stat Med. 2009;28(4):541–57. doi: 10.1002/sim.3491 19065627

[115] Johnson Pradeep R, Ekstrand ML, Selvam S, Heylen E, Mony PK, Srinivasan K. Risk factors for severity of depression in participants with chronic medical conditions in rural primary health care settings in India. J Affect Disord Rep. 2021;3:100071. doi: 10.1016/j.jadr.2020.100071 33681860 PMC7929528

[116] Thomas S, Srinivasan K, Heylen E, Ekstrand ML. Correlates of social support in individuals with a diagnosis of common mental disorders and non communicable medical diseases in rural South India. Social Psychiatry and Psychiatric Epidemiology. 2021:1–9. 10.1007/s00127-020-01997-4 PMC8245575 33386410

