# Supplementary material for: Protocol for a randomized controlled trial of a combined motivational interviewing and behavioral couples therapy intervention to reduce intimate partner violence and alcohol use in south India
Source: PLoS One. 2025 Dec 1;20(12):e0335332. doi: 10.1371/journal.pone.0335332 (PMC12668616; doi:10.1371/journal.pone.0335332)
Supplement: S1 File — (DOCX) [file pone.0335332.s001.docx]

**PROJECT HARMONY**

**STUDY PROTOCOL**

**Table of Contents**

**Section A: Study Overview and Organizational Chart**

**Section B: Setting**

**Section C: Assessment Protocol**

C.1. Recruitment and Follow-Up Timeline

C.2. Assessment Schedule

C.3. Enrollment Procedures

C.3.1. Enrollment

C.3.2. Eligibility criteria

C.3.3. Participant identification and referral – ASHAs

C.3.4. Bringing the potential female participant to the PHC/Namma Clinic

C.3.5. Eligibility screening of potential female participants – procedure

C.3.6. Assigning dummy numbers

C.3.7. Procedures when the potential male participant comes to the PHC/Namma Clinic

C.3.8. Eligibility screening of potential male participants - procedure

C.3.9. Obtaining informed consent – husband and wife

C.4. Baseline Assessment Procedures

C.4.1. Assignment of individual permanent study ID numbers

C.4.2. Tracking form

C.4.3. Administration of baseline questionnaire

C.4.4. Communicating completion of baseline to the intervention coordinator

C.4.5 . Breathalyzer

C.5. Assignment to the intervention and control arm

C.6. Blinding

C.7. Follow-up Assessments

C.7.1. Identifying and scheduling participants for follow-up

C.7.2. Timeline for making follow-up calls

C.7.3. Procedures for making follow-up calls

C.7.4. Tracking for non-responsive participants

C.7.5. Interview procedures for 3M, 6M, 9M, 12M F/U

C.8. Common Procedures Applicable for All Waves

C.8.1. Pre-interview procedures

C.8.2. Post-interview procedures

C.8.3. Incentives

C.9. Communications

C.10. Assessment – Quality Control

C.11. REDCap & Data Safety Procedures

C.12. Participant Withdrawal, Loss to Follow-up or Death

C.13. Office Procedures

**Section D: Intervention and Control**

D.1. Intervention

D.1.1. Scheduling the first session following randomization

D.1.2. Intervention delivery

a. Preparation

b. Conducting individual MI sessions – MI session 1 To MI session 3

c. Conducting joint MI session with wife – MI session 4

d. Conducting couple sessions – BCT session 1 to BCT session 6

D.1.3. Tracking and debriefing

D.1.4. Incentives

D.1.5. Handling difficult/challenging situations

D.1.6. Suicidal ideation during intervention

D.1.7. Quality control

D.2. Control Arm

D2.1 Scheduling the Session Following Allotment to Control Arm

D2.2. Delayed Intervention for Couples in the Control Arm

**Section E: Safety Protocol**

E.1. Informed Consent and Confidentiality

E.2. Protection Against Risks

E.2.1. Violence

E.2.2. Alcohol

E.2.3. Suicidality

**Section F: Aim 3 - Mechanisms**

**Section G: Appendices**

1. Research Strategy
2. Scripts for ASHAs to orient potential female and male participants.
3. Script for the assessment team member to orient the female participant.
4. Script for assessment team member to orient the couple before they are split up for screening
5. Project Harmony – Visit Schedule
6. Screening Form
7. Script for women who have AUD at screening and want to seek help.
8. Script for women who report IPV at screening and are not eligible.
9. Basic Contact Sheet
10. Script for the assessment team member while contacting husband.
11. Referral letters to NIMHANS Centre for Addiction Medicine and/or to the nearest general hospital
12. Script for men who have AUD at screening, want to seek help and are not eligible.
13. Study Information Sheet and Informed Consent Forms – Husband and Wife
14. Breathalyzer Usage Script
15. Assessment team tracking form
16. Intervention Master Sheet
17. Control Arm Tracking Sheet
18. Assessment team certification form
19. Death Report Form – IEC
20. Death Report Form – UCSF
21. NC script for women in the intervention arm – MI #4
22. Script for women who report IPV – Control Arm
23. MI Certification Form
24. BCT Certification Form
25. MI Fidelity Proforma
26. BCT Fidelity Proforma

AA. Control Arm: Brief Alcohol Educational Module

AB NIMHANS Legal Aid/Sakhi/APSA Referral Letter

AC. Handling Difficult Situations During the Intervention

AD. REDCap User Manual

AE. Checklist: Health fairs

AF. Leave and break policy

AG. Invoicing procedures

AH. Qualitative IDI guides

**Section A: Study Overview and Organizational Chart**

Study Overview: A combined behavioral couples therapy and motivational interviewing intervention to reduce intimate partner violence and alcohol use in South India.

Globally**,** an estimated 30% of women have reported physical or sexual violence by an intimate partner in their lifetime. Women who report intimate partner violence (IPV) have worse short- and long-term health outcomes, including increased risk for sexually transmitted infections and HIV, poor maternal health outcomes, and increased risk for suicide attempts. Perpetrator Alcohol Use Disorder (AUD) increases risk taking behaviors, and impairs problem-solving and cognitive processes, which may drive IPV. The current scientific understanding of these urgent issues has following limitations: a) most interventions improve either IPV or AUD but not both outcomes; b) interventions that successfully improve both are delivered by highly trained mental health professionals, limiting access and scalability; and c) most interventions focus on either just the husband or the wife but not both. These limitations have led to a strong scientific and implementation gap of interventions that are feasible, effective, and scalable in low-resource settings to target both IPV and AUD. Our Indo-US collaborative team pilot tested an intervention to deliver behavioral couple’s therapy (BCT), based on principles derived from Social Cognitive Theory (SCT) to enhance couple’s communication, combined with contingency management to reduce alcohol use. This intervention was acceptable, feasible and showed preliminary efficacy of IPV and alcohol use in couples when the husband had AUD. We now propose to build on and extend this intervention to combine BCT with motivational interviewing (MI), delivered by primary care nurses, to reduce alcohol use and IPV among couples in India and to test this in a randomized controlled trial.

Our research team has a long history of collaborative research in South Asia. Dr. Ekstrand has a 25-year history of research in India, supported by 11 NIH-funded studies where she was the PI or MPI, six of which were at the proposed site. Drs. Acharya and Ekstrand currently oversee two NIH-funded R34 and R21 studies in South Asia successfully using MI. Dr. Srinivasan has led several studies that examined the relationship between AUD and high-risk behavior, including IPV, and was the senior PI of our pilot intervention on which this proposal is based. Dr Srinivasan has also been MPI on three NIH-funded R01 studies with Dr. Ekstrand.

We propose to build on this evidence base and robust research infrastructure at primary health clinics at our South India site. We will conduct a randomized controlled trial (n= 400 couples) and study the impact of BCT and MI in reducing IPV and AUD. The intervention will be delivered by nurses in primary health centers who will be supervised by a clinical psychologist. We will perform intention to treat analyses to compare treatment and control groups on the two primary outcomes at 12-months follow-up: 1) mean scores on the Indian Family Violence and Control Scale and 2) number of days with a negative breathalyzer test over a 1-week period. We will assess secondary outcomes and other measures to conduct mixed-methods analyses to assess the theorized mechanisms of change influencing intervention effectiveness. If successful, our study will provide evidence for a low-cost couples’ intervention for IPV and AUD that can be delivered in primary care settings.

**Figure 1**

*Organizational Chart*

**
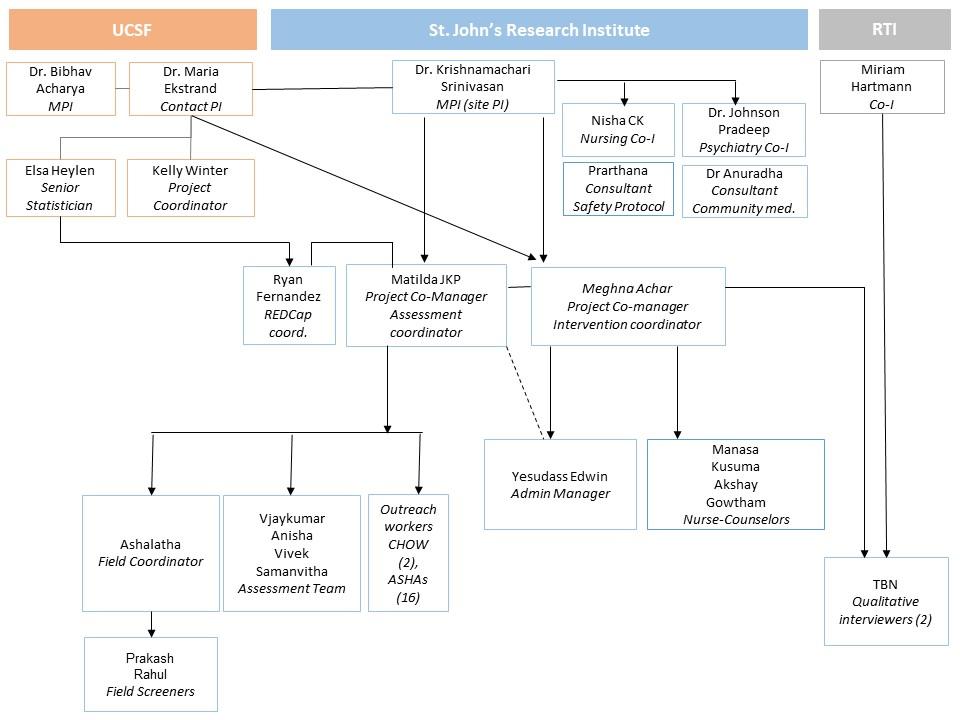
**

Community Advisory Board

Our Community Advisory Board (CAB) members represent diverse populations who help in facilitating community input by providing feedback and advice on all aspects of the research process and provide a voice to the concerns and interests of their respective communities.

The Community Advisory Board consists of the following members:

| Name and Designation | Organization |
| --- | --- |
| Mrs. Sheila Devaraj  Director | Association for Promoting Social Action (APSA), Bangalore  APSA works with underprivileged and marginalized peoples in urban slums to bring about change in the social and economic quality of their lives. |
| Mrs. Chitra D | AgniRaksha  AgniRaksha focuses on successful holistic rehabilitation of burn survivors. They also have programs focused on the vulnerable sections of society like children, women, MSMs, promoting their rights and wellbeing. |
| Fr. Shinto Mathew  Managing Director | TREDA  TREDA is a non-profit organization involved in supporting and improving the quality of life of uneducated and financially vulnerable people afflicted with substance addiction in rural and  urban areas. |
| Ms. Thangam  Assistant Director, Program Operations | Concern India Foundation  CIF works with grassroot level NGOs in the areas of education, health and community development. |
| Ms. Sumitra | Advocate |

**Section B: Setting**

This study will be conducted at government-run urban primary health centers (PHCs) located in Bengaluru, India. Permission has been granted by BBMP to involve 6 PHCs from 3 zones of Bengaluru.

Each PHC typically includes a full-time nurse, one physician, a female health volunteer (focusing on nutrition and other public health campaigns), laboratory technician, pharmacist, public health manager, support staff, and community health workers (ASHAs).

ASHAs and staff at the PHCs suggested that Namma Clinics/Anganwadis – introduced by the Karnataka State Government in 2023 to make primary healthcare available to urban poor in remote parts of the communities - may be used for recruitment, assessment, and intervention purposes, as they are relatively new and spacious buildings and closer to the community residential areas.

The list of sites for Project Harmony intervention is as follows.

**Table 1:** *Study Sites*

| **Site no.** | **Site name** | **Distance from project office at SJRI (approx.)** | **BBMP Zone, District, State** | **PHC physician point of contact** |
| --- | --- | --- | --- | --- |
| PHC1 | Kodihalli UPHC | 7.7 kilometers | West Zone, Bengaluru Urban, Karnataka | Dr. Manjunatha; Ph:9535129684 |
| PHC2 | KG Halli UPHC | 13 kilometers | West Zone | Dr. Divyavathi;  Ph: 9741010458 |
| PHC3 | J.P. Nagar UPHC | 9.3 kilometers | South Zone, Bengaluru Urban, Karnataka | Dr. Geetha;  Ph: 9008193333 |
| PHC4 | Siddapura Urban Primary Health Centre (UPHC) | 5.1 kilometers | South Zone, Bengaluru Urban, Karnataka | Dr. Ashwini; Ph: 9620495419 |
| PHC5 | Kamakshi Palya UPHC | 16 kilometers | East Zone | Dr. Shobha;  Ph: 9480683795 |
| PHC6 | Srirampura UPHC | 13 kilometers | East Zone | Dr. Sathyavathi;  Ph: 9886205341 |

Map of the locations of the PHCs


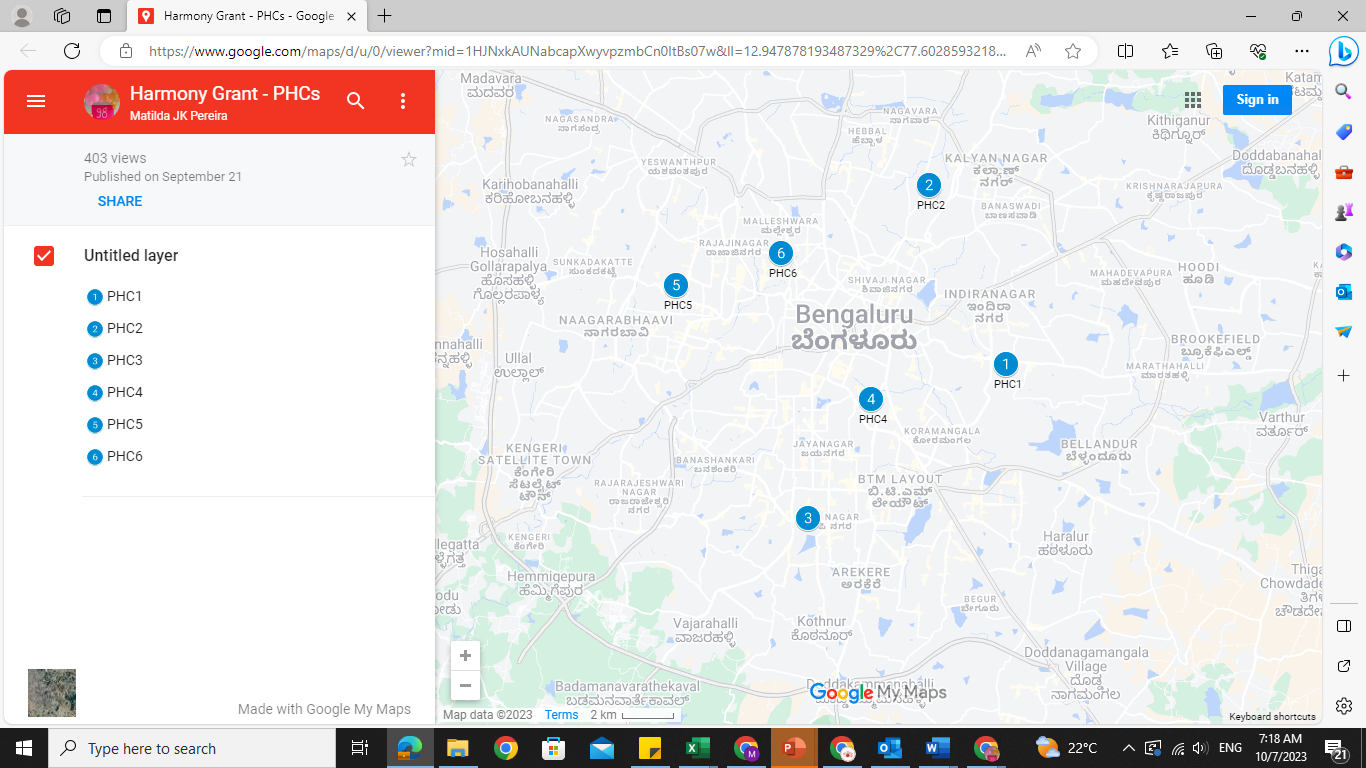


Table 2: Collaborating NGOs under the PHCs

| Site no. | Collaborating NGO | Contact persons | Contact number |
| --- | --- | --- | --- |
| PHC2 | Association for Promoting Social Action (APSA) | Mrs. Sheila Devraj, Director  Ms. Manjula,  Field Coordinator | 98861 99770  96633 67115 |
| PHC3 | Agniraksha | Ms. Chitra Dhananjay | 98860 66360 |

Section B.a: Timeline


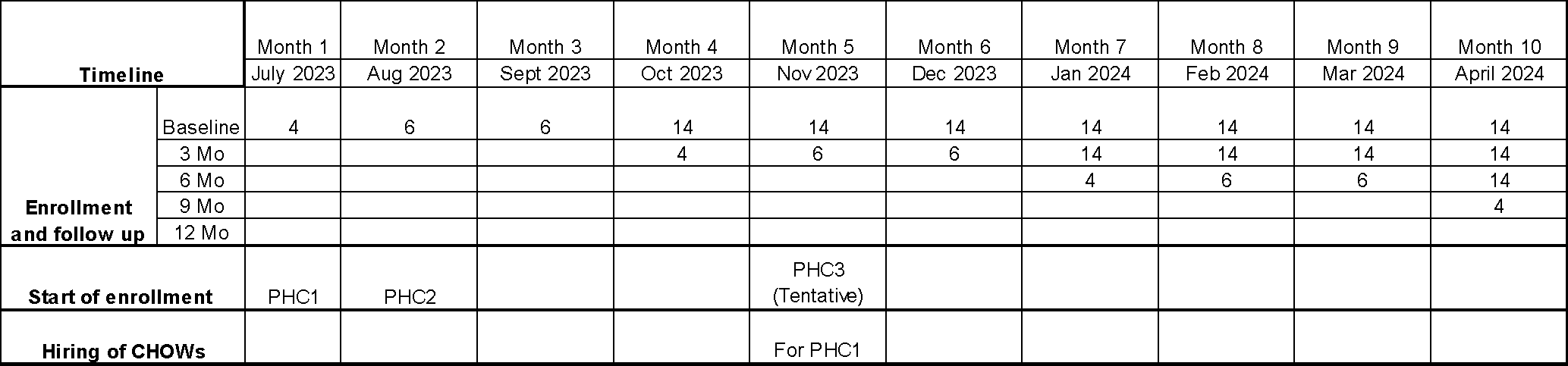


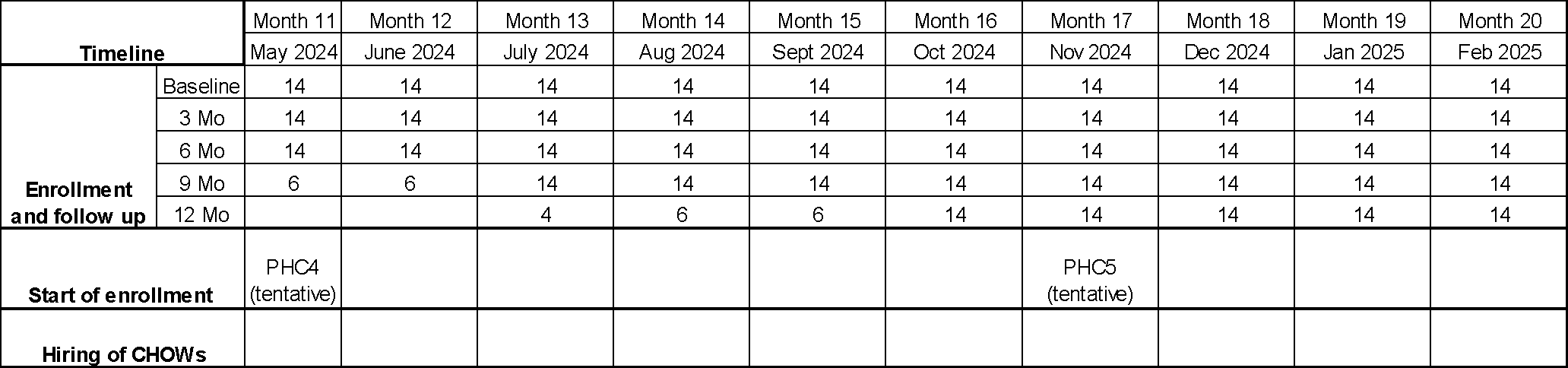


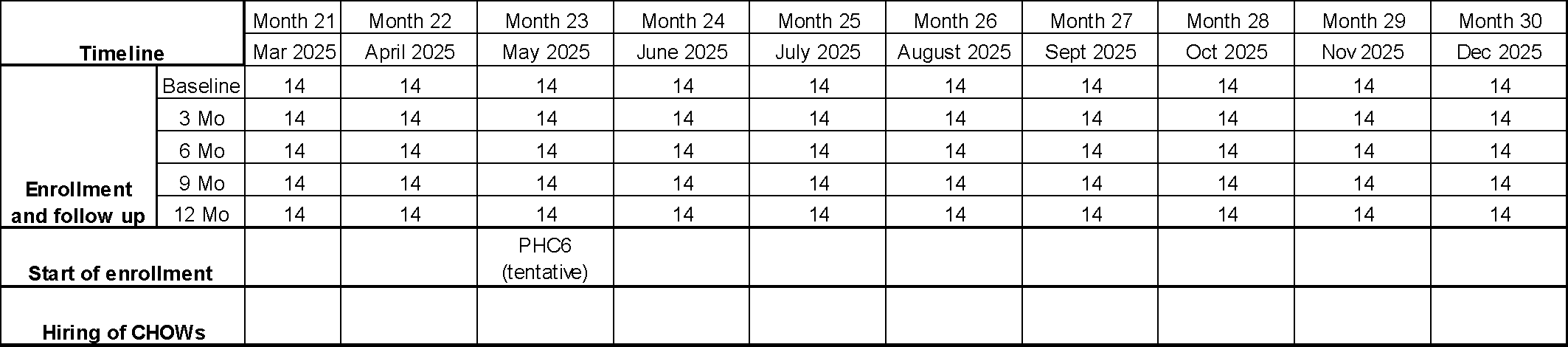


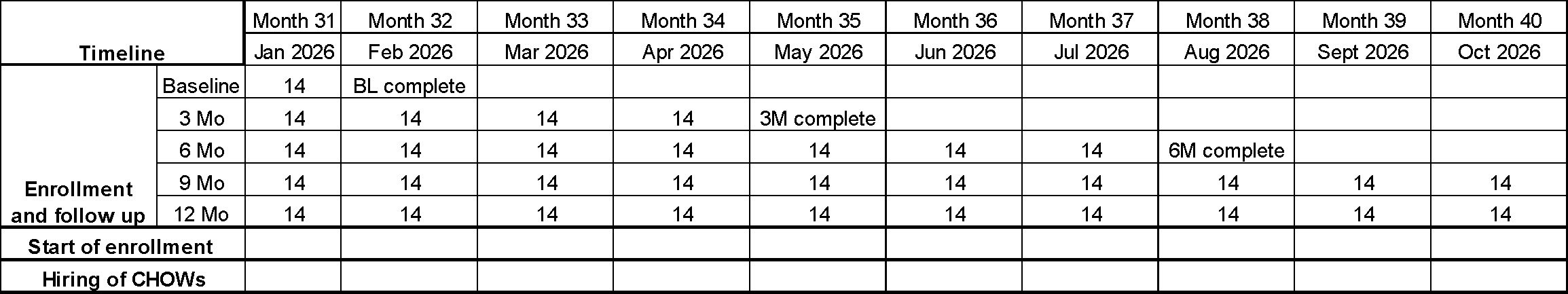


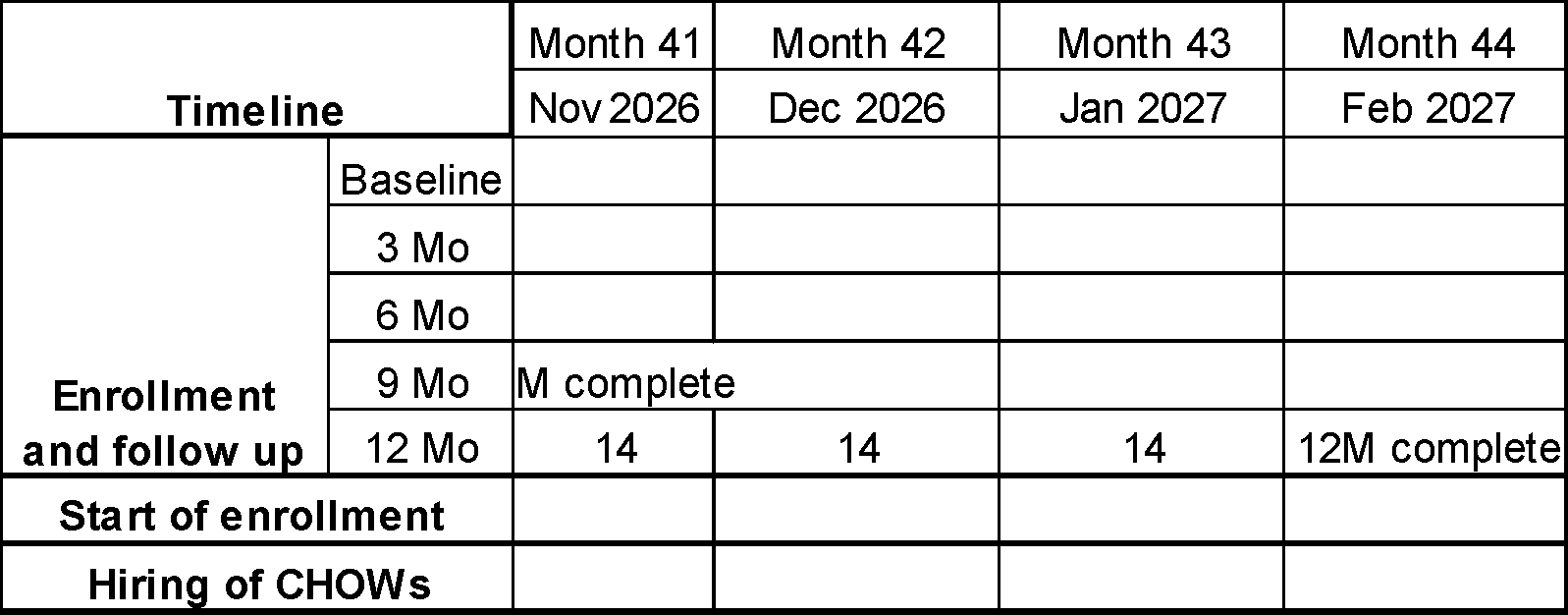


**Section C: Assessment Protocol**

The assessments will be conducted by trained assessment team members in the assessment team. Following are the different data collection components:

- Screening and eligibility assessment (Section 2.2c)
- Face-to-face interviews using tablets to enter data: Baseline, 3-month, 6-month, 9-month and 12-month follow-up.
- Breathalyzer - used for 7 days: one week **after** the baseline assessment and for one week **before** each of the 3-month, 6-month, 9-month and 12-month follow-up interviews.

**C.1. Recruitment and Follow-Up Timeline**

The table below outlines the timeline for recruitment and follow-up across all PHCs. Recruitment will begin in July 2023 and will conclude in February 2026, the 3-month follow-up is scheduled to conclude in May 2026, 6-month follow-up in August 2026, the 9-month follow-up in November 2026 and the last 12-month follow-up is scheduled to be concluded in February 2027.

## **C.2. Assessment Schedule**

## The table below outlines the assessment schedule for baseline and follow-up assessments.

**Table 3**

*Assessment Schedule*

| Assessment | Breathalyzer | Questionnaire |
| --- | --- | --- |
| Baseline | **X** | **X** |
| 3M Follow-up | **X** | **X** |
| 6M Follow-up | **X** | **X** |
| 9M Follow-up | **X** | **X** |
| 12M Follow-up | **X** | **X** |

**C.3. Enrollment Procedures**

***C.3.1. Enrollment***

We will enroll 400 married couples i.e., 800 participants total – 400 husbands and 400 wives. 200 couples will be randomly assigned to the intervention arm and 200 couples to the control (enhanced usual care) arm.

***C.3.2. Eligibility Criteria***

*Inclusion criteria for both spouses:*

a. Married and above the age of 18 years.

b, Currently living with their spouse.

c. Understands Kannada fluently.

d. Able and willing to participate in the study, provide informed consent, contact information, and express a willingness to return for follow-up visits as per the study schedule for the next 12 months.

e. Live in the catchment area of the PHC/Namma Clinic where recruitment is carried out.

*Additional inclusions for female participants:*

- - - Report that her husband drinks alcohol
    - Short blessed cognitive score of <7
    - IVAWS – Presence of IPV in the past year
    - Audit score of <4

*Additional inclusion criteria for male participants:*

- - - Audit score of >4
    - Short blessed cognitive score of <7
    - SADQ score (Severe Alcohol Dependence Questionnaire) of <31
    - CIWA – AR score of <8

*Exclusion criteria for both spouses:*

a. Impaired cognitive functioning indicating potentially diminished capacity to participate in the study.

b. Does not understand the consent form.

c. Family members of the same household are enrolled in Harmony.

d. Was a participant of the “Beautiful Home” study.

e. Presence of any significant medical problems that makes it impossible to participate in study

*Additional exclusion criteria for female participants:*

- - - Hospitalization due to IPV in past 1 year

***C.3.3. Participant identification and referral – Strategies***

a. Participant identification and referral by the ASHAs in collaboration with the Harmony Grant screening team

b. Approaching women / men / couples who visit the PHCs

c. Conducting community health fairs in collaboration with local NGOs

d. Conducting community meetings in collaboration with our NGO partners

e. Contacting PHC patients via the medical officer at the PHC/Namma Clinic

C 3.3. a Participant identification and referral by the ASHAs in collaboration with the Harmony Grant screening team

ASHAs will identify potential couples based on their knowledge of community members and ongoing interactions during their routine health work. They consider eligibility indicators, such as: Language proficiency (to ensure participants can understand and engage in Kannada), Cohabitation status (confirming the couple currently lives together), Potential relevance to study criteria, such as alcohol use or experiences of violence, based on informal assessments and observations during home visits or conversations. ASHAs refer potentially eligible couples to the screening team by providing background information. Importantly, ASHAs are trained by the Field Coordinator to emphasize the inclusion of both the wife and the husband ASHAs will also accompany the screening team to introduce them to potential participants and help build trust. The screening team coordinates with the ASHAs after conducting an initial assessment of couples based on basic criteria—such as language, cohabitation status, and the likelihood of meeting AUD/IVAWS eligibility (based on input from the referring ASHA)—to schedule the couples for full screening.The screening team will also assist in conducting health fairs, community meetings that will promote the study and improve visibility of the study to help reach out to interested women / couples in the community.

*C.3.3.b: Recruitment strategy 2: Approaching men, women and couples who visit the PHCs*

- On days when no follow-ups or outreach activities are scheduled, assessment team members will be stationed at their assigned Primary Health Centers (PHCs) from 10:00 AM to 4:00 PM, Monday through Friday, excluding holidays. Stationing team members at active PHCs ensures they are available in case follow-up visits are scheduled on short notice, and allows them to respond promptly to any referrals made by the Medical Officer (e.g., walk-in couples) or ASHA workers who may wish to refer couples while conducting their field visits.
- The female team members / screeners will approach the women, the male team members / screeners will approach the men and either of them will approach couples who visit the PHC for medical consultations, seated in the waiting area.
- The team member will introduce the study as a program to help improve the relationship between couples and will discuss with the wife ways to bring the husband / discuss with husband about meeting with the wife in the PHC so that he/she can be oriented to the study as well. The team member will hand over a pamphlet to the woman / man / couple. The assessment team member will then set an appointment and collect contact information to follow up.
- If the team member meet a couple, then the team will try to schedule a screening followed by a baseline assessment and will carry out screening procedures described in C3.3.3.e. below and, if eligible, continue with enrollment.

*C. 3.3.c: Recruitment strategy 3: Conducting community health fairs in collaboration with local NGOs*

- We will collaborate with NGOs in the catchment areas of the PHCs to conduct health fairs, i.e., an event where we will check height, weight, BMI, blood sugar, blood pressure. These will be evaluated by Harmony study’s community health physician.
- Two to three days prior to the health fair, the harmony study team, along with the NGO volunteers will conduct an outreach in the community to raise awareness of the upcoming health fair.
- The health fair will be conducted in an easily accessible venue in the village.
- The project administrator along with the NCs will ensure that the list of items (refer Appendix AD) required for the health fair are kept ready two days prior to the health fair.
- On the day of the health fair, the project NCs will help facilitate the medical checkup and the results will be recorded on a note pad and handed over to the couple. The couple will then take the record to the community medicine physician.
- The community medicine physician will go over the results with the couple, make referrals as appropriate, and let them know that there is a study for couples that might benefit them and send the couple to the assessment team member table for screening.
- Once the assessment team meets the couple, the team will carry out procedures described in C3.3.e. below.

*C 3.3.d: Recruitment strategy 4: Conducting community meetings in collaboration with our NGO partners*

- We will collaborate with NGOs in the catchment areas of the PHCs to conduct community meetings for couples.
- Our team will visit the community one day before the community meeting to meet with the coordinators from the NGO and advertise about the community meeting in the area.
- The community meeting will have talks, presented by resource persons on topics relevant to the couples such as financial literacy, government schemes for families, child nutrition, etc.
- At the end of the meeting, we will present the study and issue pamphlets to the couples present.
- As couples leave, we will ask them to give contact information for further follow up to gauge whether they are interested in participating.
- If interested, the couple will be asked to visit us at the PHC/ community hall depending on the convenience of the couple for screening.

C.3.3.e. *Contacting PHC patients via the PHC medical officer/NGO staff*

The medical officer may offer to provide the team with a list of contact details based on their patient records. In this circumstance, the assessment team member can only call or visit a potential participant after someone who is on staff at the PHC or NGO (Doctor, nurse, ASHA, NGO staff) has made the initial contact and only shares patient contact info with Harmony staff once they receive a verbal or text agreement from the husband or wife. The PHC/NGO staff can either 1) text the individual/couple and give us the team their contact information after receiving their permission, which can be a simple "Yes" via text, or 2) call the individual/couple and hand over the phone to the research team after getting verbal permission to do so.

- Once the assessment team meets the couple, the team will carry out additional screening and enrollment procedures described in C3.3.e below.

*C.3.3.f. Introducing the study to the couple under strategies 2-5:*

The assessment team member will provide a brief introduction to the study using a standard script called “*Script for the assessment team member to orient the couple*” [see Appendix] that covers an introduction to the study, process of screening and consenting, and the number of visits the participant is expected to makeover the period of one year if they are eligible and consent to participate. The assessment team members will use a document called “*Project Harmony – Visit Schedule*” (see Appendix) that shows visually the components of the study they are expected to be a part of once they consent to participate. To ensure that the couple has understood the study and its components, the assessment team member will request the couple to repeat what they have understood and clarify their queries and concerns, if any.

The assessment team member will check with the couple if they are willing to go ahead with the administration of the screening form that determines their eligibility. The screening of the husband and wife will be conducted simultaneously. A female assessment team member will conduct screening of the woman (see section C.3.4) and a male assessment team member will conduct screening of the man (see section C.3.5).

***C.3.4. Eligibility Screening of Potential Female Participants – Procedure***

Once the woman has agreed to partake in the screening process, the assessment team will administer the *Screening Form* [see Appendix] to determine eligibility. The assessment team member will administer the following:

- Short-Blessed Cognitive Test (if score is 7 or less, she is eligible).
- International Violence Against Women Survey (IVAWS) (if there is no history of IPV serious enough to require hospitalization, and there are no ongoing legal issues stemming from IPV, she is eligible).
- Alcohol Use Disorders Identification Test (AUDIT-C) (if score is less than 4, participant is eligible).
- Inquire if her husband consumes alcohol (only if he consumes alcohol, she is eligible).
- Rule out significant medical problems that may make it impossible for her to participate in the study (if there are no significant medical problems, she is eligible).

*C.3.4.a If the woman is not eligible for study enrollment after screening.* Woman will be thanked for coming to the PHC/Namma Clinic and the following procedures will be carried out by the assessment team member.

- *Referrals for IPV:* In case a woman is deemed ineligible for study enrolment and has reported that she has or is currently experiencing violence at home, the assessment team member will refer to the “*Script for women who report IPV at screening but are not eligible*” (See Appendix) to be able to further refer women to the referral services available. The assessment team member will encourage the woman to decide how she would like to keep the contact details with her (mentioned below – “Handling referrals to women”). If she wishes to seek help and is hesitant to go alone, the assessment team member will offer to accompany the woman on a mutually ascertained date or will link the woman up with an ASHA she is familiar with - to solicit the ASHA's help to accompany the woman to the referral body she opts for. The ASHA will be trained for facilitating this.
- *Referrals for AUD*: In case a woman who is deemed ineligible for study enrolment has a score of 4 or more on AUDIT-C, she will be given a referral to NIMHANS Centre for Addiction Medicine or the nearest general hospital to avail treatment for AUD, if interested. The assessment team member will inform the woman of her options by using the “*Script for women who have AUD at screening and want to seek help*” [see Appendix].

**Handling referrals to women.* If the woman REPORTS IPV but IS NOT ELIGIBLE to for study enrollment and IS INTERESTED IN SEEKING HELP, the following options will be provided to the woman for accessing the contact details of the referral facilities:

- The assessment team member will offer to write the contact details of the referral facilities on a piece of paper – a physical copy that she can keep with herself.

- The assessment team member will ask if she would like to take a picture of the document/a particular referral facility and save it on her phone if she has one. The assessment team member will also teach her how to store the image in a locked folder on her phone.

- The assessment team member will ask if she wants to save the phone number and the contact person’s name from the referral facility on her phone, if she has one.

IF THE WOMAN DOES NOT WANT TO KEEP ANY MATERIALS, the assessment team member will give a copy of the referral details to an ASHA with whom the woman is familiar and let the woman know that she may contact the ASHA in case she needs to access the referral contact details.

***C.3.5. Eligibility Screening of Potential Male Participants – Procedure***

The assessment team will carry out screening using the *Screening Form* [see Appendix]. The assessment team member will administer the following:

1. AUDIT-C: score of 4 or more is eligible.
2. Short-Blessed Cognitive Test: if score is 7 or less, he is eligible.
3. Severe Alcohol Dependence Questionnaire: if score less than 31, he is eligible.
4. Clinical Institute Withdrawal Assessment for Alcohol – Revised (CIWA-AR): if score is less than 8, he is eligible.
5. Rule out significant medical problems that may make it impossible for him to participate in the study: if there are no significant medical problems, he is eligible.

*C.3.5a. If the husband is found not eligible for study enrollment after screening.* The assessment team member will thank the couple for their time and for coming to the PHC/Namma Clinic. If the husband has a score of 4 or more on AUDIT-C, it implies he has AUD. The assessment team member will then provide the husband with *referral letters to NIMHANS Centre for Addiction Medicine and/or to the nearest general hospital* [see Appendix] and orient him to how he may seek help from these referral facilities using the “*Script for men who have AUD/problem drinking at screening*” [see Appendix].

If both the wife and husband are eligible, they move on to the Informed consent procedure (see below).

***C.3.7. Obtaining Informed Consent – Husband and Wife***

The informed consent will be obtained only once the couple has committed to attending the baseline interview.

The wife will be seen by a female assessment team member while the husband will be seen by a male assessment team member, in separate rooms.

The assessment team member will read out the Study Information Sheet [see Appendix] containing information about the study protocol, potential risks, potential benefits, and amount of time involved in study participation followed by the Informed Consent Form [see Appendix]. The form will be read section by section and after each section the participant will be asked what he or she understood. The assessment team member will ensure that the participant has understood what was said before moving on to the next section. If the participant has any questions or concerns, they will be addressed by the assessment team member at this time. If the assessment team member does not have the answer to a question, they are to contact the project manager for clarification and further direction.

The assessment team member will write down the date and the place of consent on the consent form and get the signature of a witness on the form.

If the participant provides verbal consent, the assessment team member will request them to sign or obtain their thumbprint on the informed consent form and the signature of a witness in whose presence the consent was read to the participant.

If the **husband does not consent**, then he will be thanked for coming to the PHC/Namma Clinic and the couple will not be enrolled. If interested to seek help for problem drinking, the assessment team member will provide the husband with *referral letters to NIMHANS Centre for Addiction Medicine and/or to the nearest general hospital* [see Appendix] and orient him to how he may seek help from these referral facilities using the “*Script for men who have AUD/problem drinking at screening*” [see Appendix], if interested.

If **the wife does not consent**, she will be thanked for coming to the PHC/Namma Clinic and the couple will not be enrolled. If interested, she will be provided with a referral list of resources for medical, psychological, and legal support or for treatment of AUD if interested.

**If the couple consents** to participate in the study, the assessment team members go ahead with administering the baseline interview.

Contact attempts will be recorded on the *Tracking attempts sheet* [see Appendix].

**C.4. Baseline Assessment Procedures**

***C.4.1. Assignment of Individual Permanent Study ID Numbers***

Before beginning the baseline interview, the couple will be assigned a Permanent ID number that will be used throughout the whole study. Each Permanent ID will be a 5-digit number, with the first digit representing the PHC (1-6), the next 3 digits representing the couple (001, 002, …) and the last digit representing husband (0) or wife (1).

Examples: The first couple enrolled from PHC 1 will be 10010 and 10011. The fifteenth couple from PHC5 will be 50150 and 50151.

In addition, in each assessment wave, each couple will have a separate record ID in REDCap, the program in which the surveys are administered (see below). These record IDs start at 1 and reflect the chronological order of the interviews for that wave.

***C.4.2. Tracking Form***

- The Tracking form is a document which is completed for every participant who consents to participate in the study. The assessment team member will record:
- Name, age, PHC
- Record the permanent ID assigned. (See C.4.1 above)
- Make sure that at least three means of contacting the participant for follow-up are obtained and recorded on the tracking form– including the correct address, contact numbers, persons to contact, and places to contact.
- The tracking form must include a secondary contact to be considered complete.
- The primary number and the secondary contact numbers will be verified on the spot using the study mobile phone number.
- Restricting details to a single mobile number on the tracking form to contact participants will be considered incomplete. Once the tracking form is complete, the assessment team member will administer the baseline questionnaire, per study protocol.

***C.4.3. Administration of Baseline Questionnaire***

Following the completion of the tracking form, the assessment staff will administer the questionnaire on REDCap using the study tablets. Each question in the questionnaire will be available in Kannada and English. Assessment team members will ask all applicable questions verbatim and mark an answer for each question. If a respondent declines to answering a question, or does not know what to answer, the assessment team members will first try to reassure the participant that all responses are confidential, and that there are no right or wrong answers, and if the participant is not sure of the response, they can give their best guess.

As a precaution against technical errors, the assessment team members will always carry paper copies of the questionnaire with them as a back-up. If after several attempts and troubleshooting, questionnaire administration via tablet is not possible with the help of the SJRI REDCap Team, the questionnaire will be administered via paper and pencil, and the data entered on the tablet afterwards (see C.11 below).

***C.4.4. Communication of completion of Baseline interview to the Intervention coordinator:***

Once the baseline interview has been completed, on the same day, the assessment coordinator will inform via email to the intervention coordinator about the enrollment. The Intervention coordinator will access the tracking forms filed in the office on the next day, to contact the participants to help the intervention team schedule the intervention / control sessions of the enrolled couple.

***C.4.5. Breathalyzer***

After the baseline interview has been completed, the male assessment team member will hand over the breathalyzer and the charger to the participant. Along with the breathalyzer, the male assessment team member will hand over a zip lock bag to the participant in which the participant will need to put the used mouthpiece of the breathalyzer and the straw used for recording. The straw will be cut in half before being handed over to the participant for ease of use.

The male assessment team member will demonstrate and explain the usage of the breathalyzer by administering a script named “Script to demonstrate the use of Breathalyzer” (See Appendix) and will also demonstrate the use of the breathalyzer by conducting a test blow (test1) and following the demonstration will request the participant to do another test blow (test2). The test1 and test2 photographs will be shared by the male assessment team members in a Whatsapp group with the following information like device sl. no. participant ID, if the breathalyzer bag was checked before issuing if the a. charger, b.machine, c. straws and mouth piece, d. ziplock bag were all available. Any notes about if the participant is using a keypad phone / or has whatsapp, best time to call will also be recorded. This will help the breathalyzer data coordinator with the next steps of data management.

The day on which the baseline was conducted will be considered as day 1 of the breathalyzer test and the participant will continue to use it for the next seven days. Participants will be requested to capture the breathalyzer test being done using their phone camera and send the photograph to the assessment team via WhatsApp. The assessment team member who conducts the male participant’s baseline interview will be responsible for adding the office WhatsApp number (managed by the breathalyzer data coordinator) to the participant’s phone and sending a test message to confirm that the number has been saved. This ensures smoother communication when the participant sends the breathalyzer image. The breathalyser data coordinator will acknowledge each WhatsApp message and send daily reminders to the participant to submit their breathalyser image. Breathalyzer photographs: The images shared by the participant from Day 1 to 7 along with a “meta data” file generated by the assessment team member that has the information on when the photo was taken (as backup in case the image sent by the participant is not clear) will be uploaded to the box daily by the breathalyzer data coordinator.

On day 8, ASHA / FC / assessment team member / screener / breathalyzer data coordinator will visit the participant’s house to collect the device and the zip lock bag which will have the used mouthpiece and the straw.

Once the breathalyzer and the zip locks bag have been collected (by the FC /assessment team members / screener), it will be brought to SJRI and will be handed over to the breathalyzer data coordinator.

Once the breathalyzer data coordinator receives the device and the zip-lock bags the data will be downloaded from the device and the mouthpieces will be sterilized.

**Sterilization of the mouthpiece**:

The sterilization of the mouthpiece will be carried out by the lab in Nadathur Breast Cancer Lab at SJRI on an informal agreement with on-site PI of the study.

The breathalyzer data coordinator will store all the mouth pieces in an auto-clave bag that will be used for sterilization in the lab. This bag will be handed over to the lab assistant on every second Saturday and fourth Saturday and will be collected back on the following Monday.

The mouth pieces will be transferred to individual zip lock bags using a glove for further re-issue to the next participants.

Discarding the straws: The zip lock bags that have the straws will be collected in a plastic bag and discarded once a month to the bio-waste department.

**Transfer of data from the breathalyzer:** When the device is returned by a participant, the data from the device is transferred to a secure password protected laptop by the breathalzyer data coordinator. The device will be connected to the laptop via USB cable and copy the data to an excel file.

Once the data is copied, the data is cross checked on the device with the data that was downloaded to ensure the correctness of the data. If the data shows multiple breathalyzer readings for the *same day* and shows *identical levels of alcohol*, only the first of these readings needs to be kept and all other readings with identical data and alcohol levels may be deleted. *Any readings from the same day that show a different level of alcohol must all be kept.*

The breathalyzer data coordinator will update the breathalyzer readings and breathalyzer tracker files on the SJRI’s one drive and the folder on the UCSF box that contains the breathalyzer use photos on a daily basis

The breathalyzer data coordinator will update the data downloaded from the device to the “breathalyzer readings data file” on SJRI’s one drive and verifies with the tracker and photographs received to ensure that the data received, and the photograph received match each other.

Once the data has been pasted and a QC is done, the breathalyzer data coordinator will notify the assessment coordinator via email to cross check the updated data. Once the assessment coordinator has confirmed about the accuracy of the data via an email, the data from the device is erased.

Users of the document on the UCSF box will have their own ID and no one can use another person’s login information.

Every Friday, the readings and tracker entries for the past week, Thursday to Thursday are copied from the local version on the SJRI’s onedrive to the documents on the UCSF box. The assessment coordinator conducts one final QC and notifies the UCSF statistician about the IDs which are updated and QCd.

Once the UCSF statistician has completed QC and has confirmed accuracy of the data, the photographs are erased from the UCSF box and the study phone handled by the breathalyzer data coordinator.

**C.5. Assignment to the Intervention and Control Arm**

After both spouses have completed the baseline interview and their 7-day breathalyzer assessment, the couple will be informed of their allotted arm by a nurse-counsellor over the phone at the time of scheduling the control arm/first intervention session. The intervention coordinator assigns the couple to either study arm, referring to an a priori randomization list shared by the study statistician. The intervention coordinator then communicates this to a nurse-counsellor using the *Intervention Mastersheet,* who then, at the time of scheduling, informs the couple of their assigned arm.

**C.6. Blinding**

The assessment team will be blinded to participants’ assignment to control or intervention group. We have taken the following steps to ensure that the integrity of the blinding is maintained throughout the study:

- The intervention and assessment teams are composed of different staff members.
- The NCs will emphasize to the intervention arm participants that they are not to reveal that they have received the intervention when they are contacted by the assessment team members for scheduling the subsequent follow-up assessments.
- The NCs and ASHAs will not discuss intervention-related issues with assessment team members.
- Intervention team members will not make phone calls to study participants in the presence of the assessment team members.
- All study documents related to assignment of the intervention will be securely stored in a place that is clearly marked and inaccessible to assessment team members.

**C.7. Follow-Up Assessments**

The following are the procedures to be carried out for follow-up.

***C.7.1. Identifying Participants for Follow-Up***

The assessment coordinator / will maintain a follow-up tracker which will automatically calculate the date and month when a participant is due for follow-up.

1. Based on this follow-up tracker, the assessment coordinator, will list participants due for 3M, 6M, 9M and 12M follow-up on every Monday in preparation for the meeting with the field coordinator and the screening team–
2. The date on which the couple completes their baseline interview will be considered as a base for calculating the due date for the upcoming interviews.
3. If possible, an assessment team member will be responsible for one participant until their follow-up is completed but in cases when this is not possible, the participants will be reassigned to another assessment team member.

***C.7.2. Timeline for Making Follow-Up Calls***

For the 3M, 6M, 9M and 12M follow ups, the follow-up window is divided into three parts. Minus15 days, 0 to 2 weeks, and 2 to 4 weeks.

*Minus 15 days.* During this window, the assessment team members will start making follow-up calls 14 days before the participant is due for a follow-up visit.

*0-2 weeks.* The window within which the interviews should be.

*2-4 weeks.* In cases where the participant is not available for the interview in the 0-2 weeks after the due date, it can take up to 4 weeks after the due date before the participant is considered lost to follow up for that assessment visit.

For the 12M window, to increase the flexibility in completing the interviews, the follow up window will be extended by one more month as an exception, especially when a couple is away in their native for an extended cultural celebration.

***C.7.3. Procedures for Making Follow-Up Calls***

1. All calls will be made using the study mobile phone numbers at times specified by the participant in the tracking form.

2. The assessment coordinator will create a WhatsApp group called “assessment team follow-up communication” to handle all communication between the team members with reference to follow-up assessments.

3. If possible, participants will be contacted on their mobile phones. If mobile numbers are not available, then the assessment team member will follow the instructions provided by the participant on the tracking form with respect to how to introduce themselves when contacting the participant or the secondary contact mentioned in the contact sheet. (e.g.: call from St John’s hospital, PHC, friend, insurance agent…)

4. The male assessment team member and the female assessment team member will contact the husband and the wife respectively to let them know that they are due for a follow-up interview and that we will be contacting them again on the next day so that they can let us know if they will be coming for the follow-up assessment together or if they would prefer coming separately.

5. The next day the male assessment team member and the female assessment team member will contact the husband and the wife respectively and check with them the date and time at which they will be coming for the follow-up assessment.

6. During a call, if the assessment team member was able to reach the participant and was able to schedule a participant for a follow assessment, the online “Assessment follow-up tracking sheet” will be updated with the date and time at which the participant has agreed to come for the follow-up.

7. If a participant is scheduled for an assessment, two more reminder calls will be placed prior to the scheduled assessment i.e., one call three days prior and one call a day prior to the scheduled assessment date.

8. The assessment team member will send a reminder message via WhatsApp to those

participants who use the app, who are willing to be contacted over WhatsApp, and who are unavailable by phone (not answering calls, number switched off/not reachable).

9. The assessment team members will enter details of the call made i.e., if the calls were successful or unsuccessful, in the online “Assessment follow-up tracking sheet” (see C.7.1 above) daily.

10. After updating the document, the assessment team members will share the document with the assessment coordinator and field coordinator. The assessment coordinator will review the follow-up tracker by the end of the day and make any comments if the attempts made can be accelerated in any way.

The assessment team members can use the tablet while in the field or the computers in the office to update the “Assessment follow-up tracking sheet” based on where they are stationed on a day-to-day basis.

11. When participants indicate that they are not available during the next assessment interview date, the assessment team member will note this detail in the assessment tracking sheet. The assessment team member will start contacting the participant three weeks before the interview date to check if there have been any changes to the participant’s plans and ask if they are available for the interview.

***C.7.4. Tracking for Non-Responsive Participants***

Here are the steps to be followed in case a participant is non-responsive to contact attempts.

1. Number of tracking attempts: The tracking attempts will continue daily until the participant has been seen for their follow-up visit. A minimum of 10 attempted contacts must be logged in before the participant can be declared as "lost to follow-up" *regarding that visit* (not in terms of the entire study). These will be recorded in the tracking attempt sheet.

2. If a participant has not been scheduled for a visit with the assessment team and does not answer the calls from the study team by 2 weeks after the due date of the visit, assessment coordinator will notify the field coordinator who in-turn will inform the ASHA in charge of the area to which the participant belongs. The ASHA worker in-charge will conduct a home visit within four working days after being notified by the field coordinator . The purpose of the visit is to get in touch with the participant and check with them when they can meet the Assessment team at the PHC for the follow up visit (in the remaining time left in the follow-up window) and inform the field coordinator. The ASHA will be encouraged to use this visit as an opportunity to collect new contact details (if they have changed) and inform the field coordinator. If the contact details are changed, the assessment team member updates the tracking form. The field coordinator will record all the follow-up attempts made by the ASHA in the assessment follow-up tracking sheet.

3. During the weekly team meeting, the assessment team members / screening team will share their tracking and interview challenges with the team and the assessment coordinator /, so that they can learn from each other and help problem solve difficult situations.

** Handling calls to the study phone number from participants.* When a participant calls the study phone number, the assessment team member answering the phone will respond to the request. If the caller needs to be referred to a specific member of the team, then the person answering the phone should obtain the caller's contact information and tell the participant that the assessment team member will return the call as soon as possible. If a specific assessment team member needs to be reached quickly, the person answering the study mobile phone should attempt to contact that assessment team member to relay a message or inform the assessment coordinator immediately.

** Please note:* Participants should only be given the study mobile phone numbers if they need to contact the study.

***C.7.5. Interview Procedures for 3M, 6M, 9M AND 12M Follow-Up***

*Breathalyzer:*

- The ASHA / Assessment team member / screeners will be assigned to couples, based on the areas that they work in. Each Monday, the assessment coordinator / will provide the breathalyzer data coordinator and field coordinator with a list of names with participant follow-up dates. They will go over the list, noting which participants are due for their breathalyzer assessment in the coming week. One week prior to each follow-up assessment for the husband, the breathalyzer data coordinator in consultation with the field coordinator will work with ASHAs / assessment team member / or themselves will bring the breathalyzer to the participant’s house and repeat the instructions on how to use it (see C.4.4. above and appendix). On day 8, i.e., the day after the participant’s last breathalyzer test, the ASHAs / FC / assessment team member / breathalyzer data coordinator will return to the participant’s home to obtain the breathalyzer, and escort them to the PHC to complete their follow-up assessment visit.
- If a participant is unable to attend the follow up interview on day 8 and reschedules the interview to another day, the assessment team member in contact with the participant will request the participant to continue using the breathalyzer and continue sending us the photograph via WhatsApp / mms until the day when the participant meets us for the follow up interview.
  The breathalyzer data coordinator who manages the inflow of the photograph received from the participant and the breathalyzer readings from the device will continue recording all the data received between Day 1 of the issue of the breathalyzer until the day the follow up interview was conducted, both in the breathalyzer tracker and the breathalyzer readings document.

Incentive for breathalyzer use – 12M follow up

To increase participant compliance with breathalyzer usage requirements a monetary incentive of Rs. 200 will be provided for those who complete and submit valid breathalyzer readings over four consecutive days during the week prior to their scheduled 12-month interview, along with appropriate photo verification.

To qualify for the Rs. 200 incentive, participants must:

1. Use the breathalyzer for four consecutive days.
2. Submit a clear photograph of each reading showing:
   - The breathalyzer display with the result visible.
   - A timestamp

Phone interviews:

Phone Interviews at 3-, 6-, and 9-Month Follow-Ups

To enhance retention and maintain engagement with study participants, phone interviews will be offered at the 3-, 6-, and 9-month follow-up points for couples who continue to remain in contact with the team but are unable to attend in-person interviews. This strategy is particularly applicable in scenarios where in-person follow-up is unlikely due to logistical challenges, relocation, time constraints, or other personal reasons.

Process:

1. Scheduling:
   - The assigned assessment team member will contact the participant in advance to schedule a convenient time for the phone interview.
   - Multiple reminders (via call or WhatsApp) may be sent to ensure the participant is available and prepared.
2. Interview Format:
   - Phone interviews will follow the same structured questionnaire used in in-person follow-ups, ensuring consistency in data collection.
   - All responses will be recorded in real-time on the digital data collection platform (e.g., REDCap).
3. Privacy Assurance:
   - The interviewer will confirm whether the participant is in a private setting before asking any sensitive questions.
   - If privacy cannot be ensured, the call will be rescheduled.
4. Documentation:
   - The mode of interview (phone) will be marked clearly in the “comments section of redcap” for future reference.
5. Follow-Up Support:
   - Participants will be reminded about the next scheduled follow-up (if applicable) and provided with contact details in case they have further questions.

*Tracking forms:*

Before administering the follow-up questionnaire, the assessment team member will review the tracking information with the participant to check if there are any changes in address and/or contact numbers since the last interview.

If there are any changes in the tracking information, a new tracking form will be completed. If there is no new information, the assessment team member will make a note on the tracking form that it was reviewed and there are no changes.

After verifying and updating the tracking form, at the start of the interview, the assessment team member will check the breathalyzer results for any alcohol use in the past 7 days and enter a yes/no response for this in the REDCap survey. The assessment team member will then administer the follow-up interview.

*Data forms:*

Screening, tracking and consent forms: The assessment team members will hand over the screening form, tracking form and consent forms to the project administrator at the end of each day for filing at SJRI.

All the above confidential documents will be kept under lock and key and all the digital documents will be stored securely in UCSF Box.

***C.8.3. Incentives***

Participants will receive reimbursement of Rs. 350 at the time of baseline, Rs. 400 at 3 months, Rs 450 at 6 months, Rs. 500 at 9 months and Rs. 550 at 12 months for their time. In addition, couples will receive a travel reimbursement of Rs.200 per visit. The assessment team member will obtain a signed receipt (available in the office) from the participant

Signed receipts will be cross-checked with the number of participants who have been interviewed on a given day to ensure the appropriate amount of reimbursement has been given to participants based on recruitment and follow-up numbers. A participant can refuse reimbursement. Whenever this happens, it needs to be communicated immediately by the assessment team member to the assessment coordinator / and project administrator via an email for documentation purposes.

**C.9. Communications**

The assessment team will have two study mobile numbers. One mobile number will be shared with husbands to contact the male assessment team members, and one mobile number will be shared with the wife to contact the female assessment team members.

These phone numbers will be used to contact the participants for scheduling assessments as well for any other purpose of the study. Participants or their family members can contact the assessment team members on these mobile numbers in case of emergencies. The assessment team will also have a designated tablet computer with a WhatsApp number through which messages will be sent to the participants to handle communications.

The participant can contact and talk to the study staff anytime on this number. Assessment team members will take turns being on call and carry the study mobile phone with them after office hours. The assessment coordinator will make sure that a male and female assessment team member will be reachable 24 hours a day through one of these numbers.

**C.10. Assessment Quality Control**

The following are quality control measures for the assessment team.

***Human subjects certification***

Every team member is required to complete an online Human Subjects Protection Course by CITI (Collaborative Institutional Training Initiative). The assessment team members are expected to complete the social-behavioral-educational course under the Human Subjects Protection training on the website. The team members can register for the training by accessing this link <https://www.citiprogram.org/index.cfm?pageID=154&icat=0&clear=1&_ga=2.57750953.2019871031.1542216516-2128224034.1542216516> and follow the below procedure:

1. Select UCSF as your organization.

2. Fill in name and email address

3. Complete username and personal profile sections, do not select to receive continuing education credits.

4. Select human subjects protection training social-behavioral-educational course

The training usually takes two to four hours. There will be self-assessments in each module that assesses the knowledge of the person taking the course on the content they have just read. Once the course is completed, the assessment team member will receive a completion report which will be shared with the assessment coordinator and project administrator via an email. A hard copy of the completion report will be filed in the office and a soft copy will be uploaded in the UCSF Box by the project administrator.

***Assessment Protocol – Certification***

Once the assessment team members complete the Human Subjects Protection course, the assessment team members are taken through the protocol and trained on administering screening, consent, tracking and questionnaire administration.

The training will consist of mock interviews, Q and A sessions that will be conducted based on a training plan put together by the assessment coordinator. The assessment team members will work on the individual feedback given during training and will try to display improvements in the next mock interviews and Q & A sessions. Assessment team members will be trained in administering the questionnaire via tablet using RedCap software.

At the end of the training, each assessment team member will conduct mock interviews that will be rated by the assessment coordinator based on a *scoring document* [see Appendix] on a two-point scale (1- Completed, 0 - Needs additional training). If the assessment team member completes every section of the mock, she or he is considered “certified”, and if the assessment team member does not quality in the certification process, he/she will go through booster training; re-certification in components they failed to score will be carried out. This process will be repeated until the assessment team member has been declared certified.

***Regular and Ongoing Supervision***

The assessment coordinator will supervise the staff on a daily basis and report directly to Drs. Srinivasan and Ekstrand communicating via phone, meetings, weekly reports, and e-mail.

***Quality Checks***

The assessment coordinator and the field coordinator will observe one interview per assessment team member each month to ensure that the assessment interview is being conducted according to protocol. After observing, the assessment coordinator will provide feedback to the assessment team member one-on-one and in the group format during team meetings. All observations made and feedback given will be recorded in a QC document maintained by the assessment coordinator which will be shared with the PIs on a regular basis as and when the QC has been completed.

The assessment coordinator will conduct random quality checks of study procedures in the PHC/Namma Clinic once a week. All observations made will be recorded in a QC document maintained by the assessment coordinator / which will be recorded in the weekly report to be communicated with the MPIs and the feedback will be shared with the assessment coordinator one-on-one.

***Team Meetings***

The assessment coordinator will hold weekly supervision meetings with the assessment team to discuss issues/challenges during the week including areas of the protocol that need more clarity, errors found during reviews and other issues observed by the assessment coordinator / and field coordinator during their regular QCs.

Apart from the weekly meetings, the assessment coordinator / can conduct meetings and discuss local issues with the teams on an ad hoc basis.

Any suggestions that impact data collection should be brought up with the UCSF statistician, who will consult with PIs, before implementation.

***Refresher Training***

The assessment team will go through refresher trainings on the assessment protocol at least once a every three months or on a need basis to ensure the assessment team is always kept up to date with reference to the current protocol that will help the team deliver quality work in all the aspects of the assessment protocol.

**C.11. REDCap Data & Data Safety Procedures**

**Conducting the interview:**

Assessment data will be gathered through tablet-administered questionnaires, through the web-based version of REDCap. The tablets are equipped with sim cards that were tested and provide reliable internet access at all but one of the field sites (Jio). Back-ups in case of problems with internet access consist of using a project cell phone as a hotspot with service from a provider that has reliable coverage at the recruitment sites (Vodafone or Airtel). REDCap will save data directly to the SJRI server in real time as and when the interviewers click on “Save and Stay” on REDCap after completion of each section. All tablets used to collect data will be password protected. Quality of this data will be assured by programming the data collections tools such that non-plausible values or missing responses bring up an immediate warning for the interviewer’s review, and skips based on previous answers are automatically implemented. Extensive pre-testing via mock data collection sessions will be performed to ensure warnings and skips work as intended.

***Pre-Interview Procedures***

. Assessment team members carry the tabs with them and will make sure the tablets are fully charged, are working well and have no technical issues by making a dummy entry. Assessment team members will take hard copies of questionnaires (as back up, in case of any technical difficulties with the tablet), tracking forms, and other required forms/documents to the sites from the inventory which is managed by the project administrator.

***Post-Interview Procedures***

Post-interview procedures include the following.

1. After conducting an interview, the interviewers are required to click on “save and stay” and conduct a review of the data collected in the presence of the participant, before they exit the questionnaire on REDCap. In case there are discrepancies with references to the responses which the interviewer might be able to clarify with the participant on the spot, the interviewer can use the “edit” option to edit the responses.

2. Once the assessment team member has gone through the questionnaire on REDCap he/she will lock the form and it will be considered “temporarily locked”.

3. All the completed forms will be remotely reviewed by the assessment coordinator /by end of each day and will be marked as “locked” on REDCap. Once the form is locked, the interviewers cannot edit the data.

4. During the review, if the assessment coordinator /finds errors or missing responses, these items will be noted down in the “errors log” maintained by the assessment coordinator.

5. The assessment coordinator along with the assessment team members will meet and discuss any errors found during the data review. This is to help decrease the risk that the errors will be repeated in future. The coordinator will also discuss helpful interviewing techniques and strategies that may help improve data collection. This may be done either on an individual basis or in group

**Procedure for Reassigning Data to the Correct REDCap record ID**

**Identifying the Error :** Data entered under the wrong record ID is identified either by an assessment team member or during data entry review.

**Documentation:** The assessment coordinator will record the participants’ permanent study IDs involved, the record ID under which this data was mistakenly entered, and the correct record ID that needs to be used for these participant study IDs. The date, time, and any circumstances surrounding the error will be noted.

**2. Verifying the Error**

- The assessment coordinator will confirm that the error has affected the data by verifying whether data entry under the wrong record ID corresponds to the participants in question.
- The assessment coordinator will communicate with the team members who entered the data under the wrong record ID to confirm that this is a case of data entry error, rather than intentional input.

**3. Notifying SJRI REDCap Team and UCSF team:** The assessment coordinator will inform the SJRI REDCap team and the UCSF team via a email immediately, including the details of the error: participant study IDs involved, wrong record ID used, and correct record ID to be used.

**4. Reassigning the Data :**

- **Data Review:** The SJRI REDCap team will conduct a thorough review of the dataset under the wrong record ID to ensure all relevant data (e.g., participant records, responses etc.) are identified.
- **Create a Backup:** Before making any changes, the team wil create a backup of the data under the incorrect record ID to preserve an audit trail in case there are issues with the reassignment.
- **Reassign Data:**
  - **Transfer Data:** The SJRI REDCap team will use the built-in data reassignment tools or, if necessary, export and reimport data to ensure that all fields are associated with the correct record ID.
  - **Double-Check:** Once reassigned, the team will carefully review the data under the correct record ID to confirm that the reassignment was successful and all fields are properly updated.

Conducting assessment using a paper questionnaire:

As a further back-up in case of technical problems, the assessment team members will always carry paper copies of the questionnaire.  If after several attempts and troubleshooting, questionnaire administration via tablet is not possible, the questionnaire will be administered via paper and pencil.

The assessment team member who collected the data on paper will enter the data into REDCap on the tablet within two working days from the date of the interview.  The assessment coordinator will verify the data entry of these questionnaires within two days of the data entry. The REDCap form will have an option to enter a date for data entry in cases of interviews conducted on paper. Simultaneously the assessment coordinator will maintain a separate excel sheet to track interviews conducted on paper and the completion of data entries.

The technical challenges in these cases where questionnaire administration via tablet failed, will be escalated to the SJRI REDCap team and the tablets that have a technical error will be handed over to the SJRI REDCap team. The SJRI REDCap will address the issue within one working day and return the tab. If there are further delays in resolving the issue with the tab, the assessment team member whose tab is being rectified, will use a back-up tab to conduct the assessments.

**Database management and quality control**

The SJRI REDCap Team will manage the centralized database. The assessment team will enter the date of interview, participant ID and the name of the interviewer who conducted the interview in a document called the “interview log”, stored in the SJRI one drive where it is accessible to the SJRI REDCap team as well. The log will also note if an interview was conducted on paper, and if so, the date it was entered in REDCap, to account for the delays in the interviews showing up the REDCap database. On a daily basis, the SJRI REDCap team will compare the new entries in the database with the interview log updated by the assessment team, to ensure that all data from all interviews that were conducted that day, are indeed present in the database.

The SJRI REDCap team will give the assessment coordinator and the UCSF statistician access to the main database, and provide a weekly progress report on number of interviews of a given wave, REDCap visit status and basic data quality information such as missing fields or errors.

Throughout the data collection period, the UCSF statistician will conduct a review of the data once a month (or once every 2 weeks in the initial stages of data collection) and run basic descriptive analyses to further inspect data quality on an ongoing basis.  If inconsistencies/errors are detected in the data, they will be communicated via email to the India based assessment and IT teams for clarification.  Errors related to participant ID or otherwise having implications for further data collection will be corrected in the local (India) version of the database, all other errors will be corrected in the UCSF statistician’s version of the database only.  All changes will be approved by the scientific team and tracked in a log.

**Data safety and confidentiality**

To protect confidentiality, information that can directly identify participants will not be collected in REDCap. The SJRI REDCap team will not have user rights that allow it to edit or delete any data in the REDCap database.

The following table explains user rights in REDCap of different staff:

| **Functions / Role** | **Create Record = start interview with a new participant** | **Enter & Edit Data  = administer interview, enter data, change data after entry** | **Lock/ Unlock records or interviews so they are no longer accessible for entry or editing** | **Export Data** |
| --- | --- | --- | --- | --- |
| Interviewer | Yes | Yes | No | No |
| Assessment supervisor, | Yes | Yes | Yes | Yes |
| Statistician | No | No | No | Yes |
| Admin (SJRI REDCap team) | No (read only access) | No (read only access) | No (read only access) | Yes |

**C.12. Participant Withdrawal, Loss to Follow-Up or Death**

***Participant Withdrawal***

When a participant withdraws from the study, the team should follow the following procedures:

- When a participant expresses that he/she wants to withdraw from the study to the field coordinator or assessment team member, the information is immediately passed on to the assessment coordinator with a copy to the field coordinator via an email on the same day for further perusal.
- The assessment coordinator or the field coordinator will call the participant and probe and note down the reasons for the withdrawal and the date of the withdrawal.
- All the information gathered will be updated to the withdrawal list on the UCSF box.
- If the wife withdraws from the study and not the husband, the husband will continue to attend the assessments and vice versa.
- The withdrawal will be reported to the Principal Investigator (brief report) on the withdrawn status of the participant by the assessment coordinator via an email and will also be updated in the weekly report.
- The assessment coordinator will write a note on the Tracking form stating, ‘withdrawn from the study’

***Death of a Participant***

When a participant’s death is reported by the ASHA, spouse, family member or friend to the field coordinator or assessment team member, they will –

- Not disclose anything to the family member or friend who reports the death about the participant’s participation in the Harmony study. They will empathize with the person who reported the death.
- If the family of the participant knows about participant’s status and participation in the study, the team member will empathize with the family of the participant and thank them for allowing the participant to participate in the study and acknowledge participant’s help in the study.
- The team member who got to know about the death will immediately update the information to the assessment coordinator.
- The assessment coordinator will fill up the death report with information on participant’s date of death, cause of death, person who passed on the information and the relationship with the participant. In case of unnatural death, collect information about how the person died (suicide, murder, accident, etc.), when the death occurred, and any additional information available and communicate to the PIs on the same day.
- Using the death report put together by the assessment coordinator, the Death Report Form for IEC [see Appendix] will be completed by the project administrator and emailed to the IEC on the same day.
- The assessment coordinator will mark the tracking forms as “participant deceased” and write notes on the time when the participant’s death was reported, date of the actual event and relationship of the person with the participant, who reported the death the participant.

**C.13. Office Procedures**

***Reimbursement of Expenses***

The assessment staff will be given a cash advance by the project administrator on a bi-weekly basis, as per the interviews planned. The assessment staff will pay the participants cash & obtain the signature of the participant on the ‘participant voucher’ after completing the information (participant id, date of interview etc.).

The assessment team members will settle the cash vouchers with the project administrator on a bi-weekly basis. If not, this will take place as soon as they get back to the study office.

***Photocopying of study documents***

The assessment coordinator, on a weekly basis, will oversee the required forms to be used on the field which includes, screening forms, tracking forms, consent forms etc., with the help of a designated assessment team member and will email and request the project administrator when a refill is required. The project administrator will keep track of the number of forms required and will ensure photocopy with the help of assessment team members in the SJRI study office and notify the assessment coordinator via replying to the email.

**Section D: Intervention and Control**

**D.1. Intervention Arm**

As part of the intervention participants will receive MI to reduce their alcohol use and BCT to improve their interpersonal interactions to improve communication and reduce the incidence of IPV. Participants will engage in 10 hour-long sessions once a week, as summarized in the table below. The husband will receive MI over 4 sessions, which is the standard primary care- based intervention to reduce AUD in community populations. His wife will be invited to join the final session. Subsequently, both partners will receive BCT over the remaining 6 sessions covering the content found to be effective in the pilot study.

| **Table: Summary of the MI and BCT sessions (10 sessions)** | |
| --- | --- |
|  | **Session Content** |
| 1 | Motivational Interviewing: standard 4 sessions for AUD following the four processes: engaging, focusing, evoking, and planning. |
| 2 |
| 3 |
| 4 |
| 5 | BCT session 1: Establish the “daily trust contract”, specific strategies to cope with reduced alcohol use. |
| 6 | BCT session 2: Revisit effectiveness of coping skills and build new strategies, if necessary |
| 7 | BCT session 3: Understanding and addressing communication challenges between partners |
| 8 | BCT session 4: Activity to encourage caring behaviors between partners. |
| 9 | BCT session 5: Active listening skills, reviewing strategies to improve communication; addressing ongoing challenges, if necessary. |
| 10 | BCT session 6: Reviewing caring activity; reviewing all lessons and preparing for long-term chance. |

***D1.1 Scheduling the First Session Following Allotment to Intervention Arm***

Following randomization, the intervention coordinator will allot the couple to one of the 4 Nurse Counselors (NC) by updating the Harmony Intervention Master Sheet. The Intervention Master Sheet has 4 tabs – one per NC. The intervention coordinator will enlist a couple in one of the 4 tabs, based on each NC’s caseload. The couple’s study IDs along with their contact number will be mentioned in the spreadsheet. After allotment, the intervention coordinator will notify the respective NC that they have been allotted a couple via a WhatsApp group for NC case allotment and session scheduling.

The respective NC will contact the male participant and schedule the first session (MI #1) within 7 days of completion of the baseline assessment. The NC who is allotted the couple will brief each partner over the phone about what to expect during the first and subsequent visits. This briefing must cover the following points.

- There will be a total of 10 sessions, each a week apart. The first three sessions will involve only the husband, and the rest of the sessions will require the couple to be present together.
- The NC will notify the date, time and venue for the first session with the male partner.
- Participants will receive two reminder calls from the NC - the first call will be three days before the scheduled date and the second reminder call one day prior to the date of the visit to confirm their availability and presence.
- The NC will ask the participants to save the phone number they contact the couple with and call them on that specific number if they wish to reschedule a session or clarify anything related to the counseling sessions.

The NC will then call the participant 3 days and 1 day before the scheduled first session or at a frequency and time preferred by the participant to confirm the participant’s attendance and update the Harmony Intervention Master Sheet after every reminder call with details of the reminder calls i.e., the date and time of the call, whether the call was successful or not, what was the participant’s response (confirmed/request to reschedule), and reason for unsuccessful call (no response, unreachable, number does not exist, switched off, etc.).

***1.2 Intervention Delivery***

The following is the detailed procedure for delivery of the intervention.

**a. Preparation.** The assigned NC will handle all sessions (MI and BCT) with the couple after allotment. Each NC will have a folder with 2 divisions. One division will contain freshly printed participant handouts (1. drinking diary, 2. grounding exercise steps, 3. the daily trust calendar, and 4. the caring activity sheet) as per the session number and number of sessions they will be conducting on the given day. For example, if MI session 2 and MI session 3 were scheduled that day, the NC will have copies of all handouts for sessions 2 and 3.

The other folder division is to be used to collect filled-out or used handouts. Filled handouts will be collected from the participant at two timepoints – one, at the start of BCT session 1 (drinking diary) and two, at the end of BCT session 6 (caring activity sheet). When the NC returns to the office from the field, they will deposit the contents of the second folder division (used handouts) in the designated storage space at the office.

Each NC will also have with them the Harmony MI+BCT Manual, NC handouts booklet, a notebook, and a pen.

As soon as an NC is allotted a couple, the NC will contact the intervention coordinator and/or the project manager to access the baseline AUDIT score of the male participant (which s/he will use in MI session 1).

**b. Conducting individual MI sessions – MI session 1 to MI session 3.** When the male participant reports to the PHC/Namma Clinic, he will be received by the respective NC and then directed to the room in which the session will be conducted. The NC will introduce themselves and start off by assessing signs of intoxication and withdrawal, if any. The NC will then proceed to conduct MI session 1, using the session guide in the Harmony MI+BCT Manual.

After the NC concludes the session, they will fill out the *Harmony Intervention Master Sheet* [see Appendix] to specify when the next session has been scheduled. The NC will confirm the date with the husband by referring to the intervention team Google Calendar, thank him for participating in the session, convey that they look forward to seeing him in the next session, and inform him that he will be called 3 days before and 1 day before the next scheduled session as a reminder and to confirm his attendance. The husband will then be directed to the exit. The NC will then fill out the *Debriefing Form* for MI session 1 and update the *Harmony Intervention Master Sheet*.

At the end of MI session 3, the NC will inform the husband that his wife is to attend the next session with him. After filling out the *Harmony Intervention Master Sheet,* the NC will ask the husband what date he prefers for the next session with his wife. The NC will then make a phone call to the wife in the presence of the husband to fix the date for the next session while referring to the intervention team Google Calendar. The NC will mark the date on the *Harmony Intervention Master Sheet.* The NC will confirm the date with the husband, thank him for participating in the session, convey that they look forward to seeing him in the next session, and inform him that he and his wife will be called 3 days before and 1 day before the next scheduled session as a reminder and to confirm the couple’s attendance*.* The NC will then fill out the *Debriefing Form* for MI session 3.

**c. Conducting joint MI session with the wife – MI session 4.** The respective NC will contact each partner 3 days before the scheduled session date and confirm their attendance on the scheduled date and time. The respective NC will then contact the couple (both partners) 1 day before the scheduled date to reconfirm and as a reminder. The NC will fill out the reminder call dates and participants’ response in the *Harmony Intervention Master Sheet.*

When the couple reports to the PHC/Namma Clinic, they will be received by the respective NC. The NC will initially speak to the wife separately – s/he will speak with the wife briefly (7-10 minutes) while referring to the “*NC script for women in the intervention arm – MI #4*” [see Appendix] to ensure her safety during the intervention and make her aware of how she may reach out to the team to seek help *in case of occurrence or exacerbation of violence* during the intervention.

After this, the NC will direct the couple to the room in which the session will be conducted. After the NC concludes the session, they will fill out the *Harmony Intervention Master Sheet* - to specify when the next session has been scheduled. The NC will confirm the date with the couple, thank them for participating in the session, convey that they look forward to seeing them in the next session, and inform them that they will be called 3 days before and 1 day before the next scheduled session as a reminder and to confirm their attendance. The couple will then be directed to the exit. The NC will then fill out the *Debriefing Form* for MI session 4.

**d. Conducting couple sessions – BCT session 1 to BCT session 6**. When the couple reports to the PHC/Namma Clinic, they will be received by the respective NC and then directed to the room in which the session will be conducted.

The NC will then commence BCT session 1. After the NC concludes the session, they will fill out the *Harmony Intervention Master Sheet* - to specify when the next session has been scheduled. The NC will confirm the date with the couple, thank them for participating in the session, convey that they look forward to seeing them in the next session, and inform them that they will be called three days before and 1 day before the next scheduled session as a reminder and to confirm their attendance. The couple will then be directed to the exit. The NC will then fill out the *Debriefing Form* for BCT session 1.

The above protocol will be followed for the remaining 5 BCT sessions. At the end of the final BCT session, the NC will notify the couple that they may be contacted in a couple of weeks or that they may already have been contacted by the research staff (baseline assessment → commencement of intervention within 1 week of baseline → 10 sessions over 10 weeks (2.5 months) → 3-month assessment after 2 weeks of the last BCT session) and that it is essential that the couple do not disclose to the caller that they have received or are receiving the intervention.

*Note:* if the participant refuses to attend MI sessions or has stopped drinking, the NC can skip MI sessions 2-4 and initiate BCT sessions with the couple.

*Note:* the scheduling window for intervention arm couples is five months, within which the NC will attempt to conduct as many sessions of the intervention as possible. When the scheduling window closes, the NC will attempt to contact both partners to inform them of the closing of the scheduling window and that the assessment team will be in touch with them.

*Note:* under exceptional circumstances where either partner in the couple has to travel outside the city for work, and given both partners are willing and interested, sessions will be conducted online.

** In the case of phone calls made to the study phone mobile numbers****.*** All calls and messages from the primary NC to the participants will be made and sent using the study phone number. In case there is a phone call from a participant to the study phone number asking for a specific NC, the responding NC will note down the contact details and reason for contact and then notify the asked-for NC on the WhatsApp group for intervention team correspondence. In case there is a phone call from a participant due to an emergency or a need to contact the referral resources, the NC will notify the intervention coordinator and/or the project manager, following which the safety protocol will be carried out [refer to the Safety Protocol section].

* *In the case of need to reschedule due to participants’ unavailability****.*** When the husband or wife informs the NC in the 3-day-prior phone call or 1-day-prior reminder phone call about either or both being unable to attend the subsequent session, the NC can offer to reschedule the session to a day within 3 days before or after the initially scheduled date. The NC will then note this down in the *Harmony Intervention Master Sheet.*

** In case of reports of heightened violence*, the NC will immediately notify the intervention coordinator and/or the project manager, following which the safety protocol will be carried out [refer to Safety Protocol section].

* *In case of no response to contact attempts and/or non-attendance,* within 24 hours, the NC will contact the other partner. If the other partner does not respond, the NC will notify the intervention coordinator or the project manager and access the primary and secondary contact numbers. The NC will introduce themselves as suggested by the participant in the initial tracking sheet. If call attempts to the primary and secondary contact numbers are unsuccessful, the NC will notify the intervention coordinator, the field coordinator, and the ASHA in-charge of the ward that the couple belongs to (after 48 hours of unsuccessful contact attempts with primary and secondary contacts). The field coordinator and ASHA will then conduct a home visit to check in with the couple about their non-attendance and non-response. The NC will follow-up and coordinate with the field coordinator and ASHA to get the couple to attend the sessions, and if they refuse to/wish to withdraw, the NC will notify the intervention coordinator and/or project manager and record this in the Intervention Master Sheet.

*Provision of sessions online under exceptional circumstances.* Under certain exceptional circumstances listed below, couples may receive some sessions online. This will only apply to: Couples who report that they are 1) unable to attend remaining sessions in person for a period of time that would extend their absence beyond the 5 months allocated to the intervention, 2) who express an interest in receiving the intervention despite their physical unavailability, and 3) who have completed the MI sessions and attended at least one BCT session to enable them both to establish a relationship with the nurse counsellor. Note that this will not be offered up front to all participants, but only to couples who meet the above 3 criteria and who have access to a private space, a smart phone, and a reliable internet connection.

***1.3. Tracking and Debriefing***

The NC will fill out the online *Harmony Intervention Master Sheet* and the respective session’s online Debriefing Form immediately after the completion of each counseling session.

***1.4. Incentives***

When a participant or couple completes a session and exits the counseling room, an NC (any NC other than the NC who is the primary counselor for the couple) will hand over Rs. 200 to the participant or couple, and state that it is a token of appreciation for them having made time and putting in efforts to travel to the PHC/Namma Clinic to attend the sessions. This will be done at the end of every session. In case either or both spouses cite the inability to miss work due to loss of wage as the reason for missed sessions, they will be offered Rs. 450 as compensation for lost wages.

***1.5. In case of emergency absence of the NC***

In case an NC applied for emergency leave and a session has been scheduled and confirmed for that day, one of the other NCs will be notified; this NC will conduct the session for that couple as a substitute for the absent NC. The substitute NC will contact the couple and inform them that their primary NC is on emergency leave and that they will instead conduct the session for that day. The substitute NC will be updated by the absent NC about the couple ID, and the session number. They will be provided with the debriefing form from the previous session as well.

***1.6. Handling Difficult/Challenging Situations***

Please refer to the *Handling Difficult Situations During Intervention* document.

***1.7 Suicidal Ideation During Intervention***

NCs will be trained to identify severe depressive symptomatology in participants. If the NC identifies this, or if the participant expresses death wishes or suicidal ideas, the NC will refer to the *Handling Difficult Situations During Intervention* document for the script to respond to the participant in the session, and then immediately notify the intervention coordinator and/or the project manager, who will then carry out the safety protocol [refer to the Safety Protocol section]. The intervention coordinator and/or the project manager will plan for the participant to consult a psychiatrist and offer an NC to accompany them to the referral facility. A *referral letter signed by the study investigators will be filled out* [see Appendix] and handed over to the consulting psychiatrist.

***1.8. Quality Control – Intervention***

The following quality control measures will be carried out.

**Human subjects certification.** Every team member is required to complete an online Human Subjects Protection Course by CITI (Collaborative Institutional Training Initiative).

The NCs are expected to complete the social-behavioral-educational course under the Human Subjects Protection training on the website. The team members can register for the training by accessing this link: <https://www.citiprogram.org/index.cfm?pageID=154&icat=0&clear=1&_ga=2.57750953.2019871031.1542216516-2128224034.1542216516> and follow the procedure delineated below:

1. Select UCSF as your organization.

2. Fill in your name and email address.

3. Complete the username and personal profile sections. Deselect to receive continuing education credits.

4. Select “human subjects protection training social-behavioral-educational course”.

The ethics training usually takes two to four hours. There will be self-assessments in each module that assess the knowledge of the person taking the course on the content they have just read. Once the course is completed, the NC will receive a completion report which will be shared with the intervention coordinator, project administrator and project manager via an email. A hard copy of the completion report will be filed in the office and a soft copy will be uploaded to the UCSF Box by the project administrator.

**Training.** Once the NC completes the Human Subjects Protection course, they will be familiarized with the study protocol. There will be rigorous theoretical and practical training thereafter on basic counseling skills, MI-specific skills, the basics of behavior therapy, understanding alcohol use disorders and intimate partner violence. Following these training sessions, the NCs will undergo extensive training in the delivery of the MI+BCT intervention, which will include didactic conceptual comprehension sessions and mock counseling sessions. Each NC will be provided with detailed individualized feedback during and after each mock session is delivered.

**Certification.** The NCs will undergo certification procedures starting from the last week of June 2023. They will administer essential sections of the MI and BCT session and be rated on specific counseling skills relevant to the intervention. The observers will use the *MI Certification Form* and *BCT Certification Form* [see Appendix] to rate the NC on each section as “Completed” meaning that they have demonstrated sufficient proficiency, or “Needs additional training” meaning they are not yet sufficiently proficient in the delivery of that skill/section and needs to recalibrate and be re-certified.

**Supervision**. The intervention coordinator will conduct daily check-ins with each NC either over the phone or in-person, to address pressing concerns if any. There will be weekly in-person group supervision sessions supervised by the intervention coordinator and the nurse co-investigator, which will help address concerns and queries of NCs across the week and encourage peer supervision.

**Fidelity checks.** The intervention coordinator will sample 10% of all sessions conducted by each NC. If an NC is assigned 50 couples (out of a total of 200 couples in intervention), that will be 50 random observations per NC, i.e., 1 session per couple. The intervention coordinator will use an online random number generator to select which session to sit in on and observe the sessions and score the quality of the intervention being delivered. Based on the NCs performance, there will be feedback and attempts to address any areas in need of approvement. Once the *MI Fidelity Checklist* and the *BCT Fidelity Form* [see Appendix] are filled out, they will be uploaded onto Box.

**Refresher training.** The NCs will go through refresher training sessions on the intervention protocol at least once every three months or on a needs basis to ensure optimal delivery of the intervention sessions.

**D2. Control Arm (Enhanced Usual Care)**

It has been found in previous research that usual care for AUD and IPV in primary health centers (PHC) is very limited and even when protocols exist, they are not routinely followed. In order to meet ethical parameters, the control arm will have enhanced usual care that will include: a) PHC clinicians (who will be trained by the research team) will conduct initial safety assessment for all participants; b) for IPV, PHC staff will refer them to a legal cell at NIMHANS, a one-stop IPV center, and inform participants of their options (including support for leaving the relationship if they wish) and; c) for AUD, they will provide a brief educational intervention based on the World Health Organization’s manual for managing AUD in PHCs and will provide referral to NIMHANS, a tertiary care mental health and addictions treatment center that has a dedicated referral system arrangement with the PHCs.

***D2.1 Scheduling the Session Following Allotment to Control Arm***

Following randomization, the NC will schedule the control arm procedure session on a date within 7 days of completion of baseline assessment, which is mutually convenient for the control arm practitioner as well as the couple to be present.

The NC will update the couple ID and date of session on the *Intervention Master Sheet.* The couple will be briefed about the following by the NC.

- There will be one session with the male participant with the practitioner that will last 30 to 40 minutes.
- There will be one session with the female participant with the practitioner that will last 15 to 20 minutes.
- Participants will receive two reminder calls from the coordinating nurse counsellor - the first call will be three days before the scheduled date and the second reminder call one day prior to the date of visit.

When the couple arrives on the scheduled date to the PHC/Namma Clinic/Anganwadi/community hall, they will be received by the NC and the control arm practitioner. The NC will direct the couple to the room where the control arm practitioner is seated.

After the practitioner and couple introduce themselves, the practitioner will request the wife to sit outside the room for a short while. The practitioner conducts the Brief Alcohol Educational Module with the husband [refer to *Control Arm: Brief Alcohol Educational Module].* After the module is completed, the practitioner will provide the husband with signed *letters to NIMHANS Centre for Addiction Medicine and/or to the nearest general hospital* [see Appendix].

The husband will be asked to step out for a short while and the wife will be invited to step in and sit down in the practitioner’s room. The practitioner will inform her of confidentiality of what she shares in the session, provides space to ventilate about her difficulties and distress, and refer to *Script for women who report IPV – Control Arm* [see Appendix] to make her aware of how she may seek help from the team in case of occurrence or exacerbation of violence during the course of her participation in the study.

Following this, the couple will be addressed together once again by the practitioner to answer questions and provide brief clarification. The couple will be given Rs. 200 for taking time to attend the session, and the NC will obtain the couple’s signatures on the voucher. The practitioner will thank the couple for coming to the PHC and direct them towards the exit of the room. The NC will then update the *Intervention Master Sheet.* The NC or the control arm practitioner will inform the couple that they will be contacted 3 months from then to visit the PHC/Namma Clinic again for another round of assessments. The control arm practitioner will remind the couple that they will be offered four sessions of the delayed intervention after their participation in the study comes to an end, that is, after the 12-month follow-up assessment.

*Note:* the scheduling window for control arm couples is two months. When the scheduling window closes, the NC will attempt to contact both partners to inform them of the closing of the scheduling window and that the assessment team will be in touch with them.

***D2.2. Delayed Intervention for Couples in the Control Arm***

When the 12-month assessment is completed, the assessment coordinator updates the follow-up tracker which can be accessed by the intervention coordinator. The intervention coordinator will update the intervention mastersheet with details of control arm couples who have completed the 12 month assessment, and will assign couples to the NCs in alphabetical order. The respective NC will contact the couple within a week of the 12 month assessment completion and will inform the couple that, if they so desire, they can receive four sessions of intervention to help the husband reduce his drinking for the husband and wife to improve communication and support in their relationship. The NC will enquire if the couple is interested in receiving the delayed intervention and update the Intervention Mastersheet of their response. If the couple is interested, the NC will discuss with the male spouse and schedule the first (MI) session and initiate the delayed intervention. If the both or either spouse inform that they are not interested, the NC will update the Mastersheet to indicate that the couple declined.

**Section E: Safety Protocol Procedures**

**E.1. Informed Consent and Confidentiality**

- As IPV and alcohol are stigmatized, when contacting participants for screening we will frame the study in terms of a family well-being intervention aimed to reduce alcohol use, family conflict and violence, and strengthening caring and supportive relationships.
- ASHAs and study staff will be trained to do their utmost to ensure that all potential participants are safe and have a confidential and comfortable space in which to make decisions regarding study participation, and if they choose to do so, complete the study assessments and interviews.
- Screening, consent, and enrollment will always be done in person.
- The study information sheet containing information about the study protocol, potential risks, potential benefits, and amount of time involved in study participation will be read to interested participants before they make a decision about enrollment and sign informed consent. The study information sheet will also emphasize that participation is voluntary, that there are no negative consequences for refusing, and that if someone chooses to participate, they can decide to drop out of the study at any time with no negative consequences.
- Following provision of the study information sheet to both couples and the opportunity to ask questions, eligible and interested participants will be asked to give written consent or a thumb print. Both will be witnessed by a person who is not part of the study.
- Participants will be offered a copy of the informed consent form in English and Kannada which will include a description of all study procedures. Participants will have the option to not keep the informed consent form, as some participants lack privacy in their homes, including access to personal documents by extended family and visiting neighbors, leading many participants to choose not to keep any study materials.
- No patient identifiers and no screening data will be transmitted back to the PHC.
- Contact tracking forms linking study ID numbers to contact information provided by participants, including names and phone numbers, will be kept under lock and key with the consent forms in the study office, and all the digital documents will be stored securely in the UCSF Box.
- Informed consent forms will be stored under lock and key, separate from the study data, and will be available only to trained staff. We will also inform participants that none of their responses will be shared with their clinic physicians or other clinic staff not involved in the study.
- Assessment data collected via tablet will occur through the mobile app version of REDCap, which is HIPAA-compliant, and data will be stored on secure, password-protected servers. When data are collected offline in the field, data will be stored on the password-protected mobile REDCap app until they are able to be uploaded to the secure database. The tablets will be password-protected to protect data as it is being collected in the field, and data stored on tablets will be erased after being uploaded and backed up. Transfer of data from the mobile apps to the server will occur through encrypted channels.
- Paper copies of the tracking information and consent forms will also be stored under lock and key, separate from the data, and will be restricted to project staff on as-needed basis.
- Participant contact information (e.g., names and telephone numbers) will be used to remind participants of their scheduled assessments, and, with permission, to conduct home visits. When contacting participants over the phone, research staff members will identify themselves in a manner agreed upon by the participants. For participants who give us permission, we will conduct home visits, ensuring that staff members identify themselves in a manner agreed to by the participants, in order to preserve confidentiality. Study staff may also track participants with the help of the ASHAs.
- During the MI+BCT intervention sessions, participants will be asked to keep all personal information shared by their partner confidential to help reduce any fears participants may have of revealing personal situations. They will be cautioned that we cannot guarantee each partner will keep session content confidential, and they may want to take this risk into consideration when sharing personal situations.

**E.2. Protection Against Risks**

All incidents or instances of emergency contact with the study team will be documented in detail in the aforementioned study log by the project manager and/or the intervention coordinator i.e.,   <https://ucsf.box.com/s/mok5xves6o0avxa0gnq99bdfz47bcgy3:> this log will include the narration/description for why additional support was sought for research reporting purposes.

Instances of emergency contact include a. exacerbation of violence during the course of participation in the study, b. severe alcohol withdrawal symptoms during the course of participation in the study, and c. emotional and psychological distress, including suicidality.

***E.2.1. Violence***

**Female participants*.*** All female participants (control *and*intervention arms) will be provided the study phone number, and *not* the resources referral contact list (containing contact numbers of Sakhi, NIMHANS Legal Aid lawyers, etc.) *unless they specifically ask*. They will be asked to get in touch with the team using the study phone number (which will be in the possession of assessment team members and NCs) in situations where their safety is compromised, if there is an exacerbation of violence, if they have sustained injuries, or if they feel unsafe and wish to seek help.

If the phone call is received by an assessment team member or nurse-counselor and the woman is in distress/in an emergency, the assessment team member or nurse-counselor will listen to the woman's concerns and notify the project manager or the intervention coordinator immediately.

The PM and IC will discuss with each other and contact the Safety Officer and in her unavailability, the Community Medical Consultant, to triage and determine the plan of action - such as connecting the woman to and setting up consultation appointments with the referral facilities listed (Sakhi One Stop Centre and the NIMHANS Legal Aid Clinic) and offer to accompany the woman in case she is hesitant to go on her own.

The MPIs will be notified within 24 hours regarding each such incident via email and/or phone.

**Women who report IPV at screening and are not eligible for study enrolment*.*** The woman will be offered a copy of the contact numbers of the referral bodies but *not* the study phone number. The assessment team member will encourage the woman to decide how she would like to keep the contact details with her i.e., take the hard copy with her, store a picture of the list or the contact numbers in a locked folder on her phone, or give the contact sheet to an ASHA she is familiar with. If she wishes to seek help and is hesitant to go alone, the assessment team member will offer to accompany the woman on a mutually ascertained date or will link the woman up with the ASHA she is familiar with - to solicit the ASHA's help to accompany the woman to the referral body she opts for.

***E.2.2. Alcohol***

**Male participants*.*** Male participants in the control arm will receive the brief alcohol education module and a referral letter to the nearest general hospital and to NIMHANS Centre for Addiction Medicine, if they wish to seek additional help. Male participants (control *and*intervention arms) who report alcohol-related complications will be referred to the nearest general hospital and NIMHANS Center for Addiction Medicine. Complications include the following: participants who are severely dependent on alcohol or are found to be experiencing withdrawal symptoms during study interviews or sessions*.*

**Men with AUD at screening but who do not meet study enrollment criteria**. The men will be offered a referral letter to the nearest general hospital and to NIMHANS Centre for Addiction Medicine, if they wish to seek help.

***E.2.3. Assessment of suicidal ideation and depression referrals***

*If the participant endorses question no. 9 (answers 1,2, or 3 on "Thoughts that you would be better off dead or of hurting yourself in some way in past 2 weeks") in the Patient Health Questionnaire (PHQ-9), the assessment team member will administer four items additional questions (SI 1-SI4) on intent, history, plan and means. The responses will be recorded on paper.*

**1) If the participant answers “yes” to all four questions (SI 1-4).** The assessment team member will tell the participant that the study psychologist will speak to them over video call to ask them a few additional questions. The intervention manager will then carry out a suicide risk assessment on video call – which will include suicidal ideation (frequency, intensity, duration), plan (timing, location, lethality, means, and preparatory acts), behavior (past attempts, aborted attempts, rehearsals and non-suicidal self-injurious behaviors), and intent (ambivalence, extent to which they expect to carry out the plan and believe the plan to be lethal). This information will be emailed by the intervention manager to the PIs within 24 hours of risk assessment completion. Following the risk assessment video call, the assessment team member will offer to arrange a referral for psychiatric consultation and (if desired) will make sure that an assessment team member accompanies the participant to the hospital.

**Conclusion: The person is considered at-risk (determined by the intervention manager).**

***If an at-risk participant does not agree to meet a psychiatrist in person.*** The assessment team member will arrange for a video consultation with the psychiatrist immediately.

***If an at-risk participant continues to refuse psychiatric consultation***. The intervention manager will explain to the participant that in order to protect him or her from serious harm:

The assessment team member will reach out to the closest family member and inform them about the participant’s plan of carrying out suicide.

- Once the referral process has been completed, the assessment team member will complete the suicide ideation form with all the details and email it to the intervention manager and the assessment coordinator. The questionnaire needs to be updated with this information as well. After verifying the information, the Suicide Ideation Form will be forwarded to the UCSF team within 24 hours.
- In case the participant refuses a referral, after two days the assessment team member will make a follow-up call. If the participant says that he or she is doing okay and is not feeling suicidal, the PHQ scale will be re-administered. If the participant scores less than 10 and does not endorse PHQ item no. 9, the process ends here. The administered scale will be attached to the original questionnaire and the Suicide Ideation form will be updated with the information by the assessment team member and will contact the intervention manager.

**2) If the participant endorses the “trigger question” (PHQ9), but states all the following:**

a) scores less than 10

b) does not have current suicidal ideation

c) does not have a history of suicidal intent or past attempt

d) does not have a lethal plan or access (access to a sari only counts as access if together with a plan and intent)

**Conclusion: The participant is considered to be low risk. These will be documented by the intervention manager on the UCSF Box Safety Log:** [**https://ucsf.box.com/s/mok5xves6o0avxa0gnq99bdfz47bcgy3**](https://ucsf.box.com/s/mok5xves6o0avxa0gnq99bdfz47bcgy3)

**These conclusions must be made by the intervention manager, who is a clinical psychologist, not by the assessment team member and not the assessment manager.**

***E.2.4. Emotional and Psychological Distress***

On PHQ-9, if the participant’s total score is *15 or higher*, the participant will be referred to the nearest district hospital psychiatrist identified by the PHC physician; the participant will be provided a referral letter (see Appendix).

For those participants who score *higher than 19* on PHQ-9, a study team member will accompany the participant to the district hospital psychiatrist and set up an appointment.

For those participants with a total score of 10-14 on PHQ-9, if the participant continues to score 10 or above at 3-months FU, they will be referred to the district hospital psychiatrist.

PROJECT MANAGEMENT

**Meetings**

*Monthly staff meeting:* A monthly staff meeting will be held on every 4^th^ Tuesday of the month to go over any topics / challenges with the entire team if required.

*Twice monthly meeting of the India-based Co-investigators and consultants* will be held to ensure everyone is updated about the progress and challenges of the project, and to brainstorm possible solutions and strategies to address these challenges.

*Community Advisory Board meetings:* The community advisory board will meet quarterly to discuss progress and challenges focusing on recruitment, retention and other field-based issues and brainstorm possible solutions and strategies to address these challenges.

**Communication**

*Team*: WhatsApp groups are used to facilitate communications between staff in the office and the field, and for those field staff coordinating study activities.

*Coordinators*: The intervention, assessment and field coordinators will have daily check-in with the teams, hold regular weekly meetings and will also be available on a per-needed basis.

The project manager and intervention coordinator will be in daily contact with the UCSF and the SJRI PIs. The project manager will put together a weekly report in collaboration with the intervention coordinator every Monday in a prescribed format reporting weekly updates on recruitment / enrollment, assessments, intervention, HR/admin and other project related topics.

The intervention coordinator will be in touch with the Co-Is Dr. Johnson and Ms. Nisha via emails to notify and update on the meetings of the Co-Is and consultants.

**Financial**

*Monthly accounting report:*

Monthly accounting reports will be submitted to the UCSF team within 15^th^ of each month by the project administrator prepared in collaboration with the project manager.

The project administrator keeps track of the funds in the study account and ensures that they are spent according to the budget under the supervision of the project manager.

*Invoices*

Quarterly invoices will be put together by the project administrator in collaboration with the project manager, signed by the SJNAHS Director, and shared with the UCSF financial analyst.

*ASHA payments*

ASHA workers who help us with referral and tracking of the participants are paid Rs.600 monthly by the assessment team member from the petty cash available in the study office. The ASHA worker signs a voucher confirming that she received payment, and the assessment or intervention staff gives the signed voucher to their coordinator. The coordinator submits the voucher, together with the claim sheet, to the project administrator for reimbursement.

## **HR**

*Leave policy*

In addition to the SJRI leave policies, the staff will adhere to the study’s leave and break policy (see appendix)

*Attendance report*

The Project administrator will calculate attendance of staff on a monthly basis and prepare a monthly attendance report on the 20th of each month. The report is cross verified and forwarded to the on-site PI by the project manager. The on-site PI will forward this report to the HR Manager.

## **Reports and submissions**

*Annual report:*

An annual report will be submitted summarizing activities for the year in collaboration with Dr. Ekstrand to be used as part of the required annual NIH progress report.

*IRB Renewal*

The renewal of IRB approval is done on an annual basis, in the month of July of each year.

The most up-to-date renewal of the St. John’s IRB approval expires on 16th August, 2025.

**Section F: Aim 3 - Mechanisms**

1. **Background**

Using purposive random sampling, a subset of up to 40 intervention couples will participate – male and female partners separately - in serial qualitative interviews. These will occur approximately 1- to 3 months after intervention completion ~at months 4 (wave 1) and at month 12 (wave 2) for a total of up to 160 interviews. Interviews will focus on individual’s experiences with Harmony study activities, particularly the intervention. Acceptance of participation in the interviews will not affect participation in the study.

Interviews will explore experiences with the Harmony study, relationship quality and experiences with spousal violence, alcohol use and potential Harmony study-related effects on these. Barriers and facilitators to intervention participation during the study, perceived quality of intervention activities, and perceptions of how participation affected hypothesized mechanisms of change underpinning the intervention will be explored. Level of sustained change will also be assessed in the wave 2 in-depth interview, along with barriers and facilitators to sustained change and any unanticipated positive or negative consequences.

1. **Purpose & Principle**

To define the qualitative data collection and management procedures for the Harmony study.

**3.0 Procedures**

**3.1 Responsibilities**

All site staff members delegated by the Multiple Principal Investigators (MPIs) to collect, record, review, and/or transmit Harmony qualitative study data are responsible for understanding and following this SOP.

**Qualitative Protocol Lead/Co-Investigator (CI):** The qualitative lead co-investigator is responsible for leading the design of the qualitative component and all related activities in collaboration with the study team. This includes oversight to the development of this standard operating procedure (SOP), development of in-depth interview (IDI) guides, and provision of templates for documentation, as well as leading training of study staff who will collect and manage qualitative study data in accordance with this SOP. They will also play a role in quality oversight and will communicate regularly with the study team regarding implementation and quality of the qualitative data collection process. The MPIs and Co-I may at any interval perform a Quality Assurance (QA) check on how the qualitative procedures are conducted, administrated and recorded, including through site monitoring.

**Qualitative Lead (QL):** The Qualitative Lead is responsible for overseeing the qualitative component, including co-development of this standard operating procedure (SOP) with the co-I, hiring qualitative interviewers, training, and generally ensuring that all day-to-day qualitative data management and collection processes are carried out in accordance with the SOP. The Qualitative Lead will ensure that overall qualitative implementation occurs according to study-specific procedures and is in line with study expectations. The Qualitative Lead will check for completeness, legibility, and consistency of data collected, or ensure that this is done by delegated staff. The Qualitative Lead is responsible for communicating regularly with the MPIs and Co-I about data collection activities, conduct of internal data QC procedures, and to provide valuable information about the context in which data collection happens. The Qualitative Lead may also assist with data analysis. The Qualitative Lead will oversee quality control and safety of incoming qualitative data.

**Multiple Principal Investigators (MPIs):** The MPIs have ultimate responsibility for the quality of data and for ensuring that all applicable Harmony study staff members follow study procedures, including this SOP.

**Qualitative Interviewers (QI):** The in-depth interview (IDI) is conducted by an interviewer. This role involves establishing a comfortable and safe environment for the participant(s), eliciting discussion on a pre-determined list of topics of interest and probing further when appropriate. The interviewer is responsible for ensuring completion of the interview audio recording and writing a debrief report within 24 hours of the interview/discussion. They are also responsible for transcribing recorded interviews into a Kannada language transcript and subsequently translating the Kannada transcript into an English language transcript. QIs may also be involved in qualitative data analysis. The Harmony study will have one male qualitative interviewer and one female qualitative interviewer on staff.

- 1. **Staff training, certification, and support**

Each staff member who will be responsible for conducting Harmony qualitative interviews must be certified before conducting IDIs with Harmony participants. For certification, staff will participate in an initial 5-day in person training co-led by the CI and Qualitative Lead and complete at least three mock interviews in Kannada deemed of sufficient quality. The two interviewers (one male and one female) must be certified before the site can begin implementation of the Harmony qualitative component.

- - 1. **Training**

The training will take place over 5 days and will cover the qualitative study design, procedures, techniques, and documentation associated with the qualitative component. Training on the overall study and relevant aspects of its conduct, as well as gender sensitivity will be included in this training, or otherwise covered during additional training time.

A refresher training will also occur prior to beginning the second wave of IDIs with participants to review procedures specific to wave 2 and relevant techniques for maintaining rapport and building on learnings from the wave 1 IDI. It will also serve as a refresher of qualitative interviewing techniques drawing from lessons learned from the first wave of IDIs conducted to date.

- - 1. **Mock interviews**

The initial training will be followed by a series of observed mock interviews. The mock interviews will be observed in-person and/or through Zoom or via retrospective observation of mock interview video recordings. These will be observed by the CI and Qualitative Lead. Certification will be awarded upon completion of at least five mock sessions (three in Kannada) that meet or exceed quality and fidelity criteria. Quality and fidelity criteria will include covering all primary topics included in the guide, adequate probing, appropriate linking of topics, quality of rapport building, and other qualitative skills covered via the in-person training. Ongoing review of fidelity and quality will occur through review of in-depth interview transcripts, as described in section 3.6. If at any time, the quality of interviewing is deemed to fall below standard, interviewers may be required to receive additional training. These will be documented on the IDI team certification form (see Appendix A).

- - 1. **Ongoing support and feedback**

In addition to training and certification, interviewers will receive ongoing opportunities to debrief and access psychological support, as needed. As conducting research on the issue of intimate partner violence can lead to burnout, compassion fatigue, and vicarious trauma, this support is critical for the wellbeing of the interviewers and the quality of the study.

The interviewers will have fortnightly online meetings with the CI and the QL and weekly one-on-one review and feedback sessions with the QL.

- 1. **Selection of IDI participants**
     1. **Number of qualitative participants**

Up to 40 couples from the intervention sites, approximately evenly distributed across participating clinic sites, will be selected for participation in the qualitative component of the Harmony study. The team will focus efforts on 2 clinics at a time, progressively increasing the sample as the study team moves to additional clinic sites.

- - 1. **How will participants be selected**

Potential participants will be selected at random by the UCSF statistician taking into account the clinic from which they were recruited and their intervention dose (i.e. level of attendance with 50% of the sample having completed all 10 sessions and the remaining having completed less than 10 sessions, but at least one session with both spouses) in order to capture diversity of intervention experiences and potential barriers and facilitators. Once the potential participants are identified, the Qualitative Lead will note their PTIDs on the qualitative tracking log (see template in Appendix B), along with their selection criteria, and will provide contact details of the participants to the qualitative interviewers via the Contact Tracking Attempt Sheet (See Appendix E). The Qualitative Lead will monitor the selection of participants for representation of individuals with experience with each Study Nurse-counsellor and will inform UCSF if random selection does not adequately represent each counsellor. All selected participants will be administered a separate informed consent for participation in the IDIs (see Appendices C-D), and this consent will be filed in a separate folder from study data. Visit scheduling, tracking, and handling of missed visits will follow standard study procedures (i.e., according to section ‘C.7. Follow-up Assessments’ in the study protocol).

- - 1. **Timeline**

Following the quarterly study follow-up visit, participants will be alerted to their selection for the qualitative component consisting of a series of two waves of IDIs around month 4 (wave 1) and month 12 (wave 2).

- - 1. **Contacting qualitative participants**

Qualitative participants will initially be alerted to their selection by the study Nurse-counselor, who has an existing rapport with the participants. The Nurse-counsellors will call the participants using agreed upon contact methods. They will assess interest in the qualitative component and will schedule them for their first interview if interested. Following the first interview, subsequent contacting of the qualitative participants will occur through the qualitative interviewers who will use the contact information provided by the Qualitative Lead. For some couples, scheduling may be done by the Qualitative Interviewers after an introduction by the Nurse-counsellor. In either case, interviewers will contact participants using the phone numbers provided. If the participants do not answer the call, the interviewers will continue to try until they get a response. In the case of no response after 10 calls (a maximum of 3 calls per day for 2-3 consecutive days), Nurse-counsellors or interviewers may choose to visit participants at their documented address themselves or with the help of the team’s NGO/ASHA collaborators. All contacts, by the Nurse-counsellors and the Qualitative Interviewers, will be documented on a tracking attempt sheet (see Appendix E). This sheet will be completed in password protected electronic copy form. Only the Qualitative Lead and staff contacting participants will have access to these forms.

Once the participant has been reached, the Nurse-counsellor will briefly explain why they are calling and will schedule a time and location to meet to conduct the wave 1 qualitative interview with the participant. See IDI Scheduling Script (Appendix F). Given that both members of the couple will be interviewed, one Nurse-counsellor may contact the couple to schedule both interviewers, however the interviews will always be conducted separately by an interviewer who is the same sex as the participant (i.e. a male interviewer interviews a male participant).

**3.3 Activities**

**3.3.1** **Preparing for the IDI**

Before each IDI visit, the following will be done by the Qualitative Interviewer:

- Confirm the participant was selected for the qualitative component. Participants in the qualitative component will be selected as described above The qualitative IDI tracking log will note the criteria for selection.
- Ensure the correct versions of the IDI guide, and informed consent (for wave 1 only) are printed and ready for use.
- Call to remind the participant of their visit.
- Confirm the availability of the interview venue.
- Confirm that the audio-recorder is charged and/or has batteries and is functioning correctly.

**3.3.2 Conducting the IDI**

IDIs will be conducted in a private location in the available research sites (e.g., at the public health clinic, Anganwadi school room, community hall, etc.) to maintain the confidentiality and safety of the participants. The provided IDI visit checklists (see Appendix G) should guide the order of procedures for each IDI that should be administered by the Interviewer.

Prior to the interview

- Interviewer will greet the participant, introduce themselves and explain their background and staff roles during the discussion.
- Interviewer will then conduct the written informed consent procedures (for wave 1 only).

Interview

- All interviewer-administered guides will be administered in Kannada.
- The IDI will follow a discussion guide but will allow for iteration, probing and digression on relevant themes.
- IDIs will be audio-recorded and later transcribed by the Interviewer in Kannada.
- Following the IDI, the participant(s) will be thanked for their time and reimbursed for travel and time.
- If at any point during the interview the participant becomes too distressed to continue, the interviewer will be trained to follow a distress protocol (see Appendix H), which will involve stopping the interview, offering a glass of water, offering tissues, inviting the participant to take a pause by walking around or resting in the room. If needed, interviews can be paused and re-continued at a later date.
- If a potential safety risk resulting from Harmony study participation is identified during an IDI, this will be brought to the attention of the Qualitative Lead who will note it in the Safety Log (per Section E.2. Protection Against Risks of the study protocol).

After the interview

- Immediately following each IDI, the Interviewer will complete their notes.
- A debrief report will be completed within 24 hours of the discussion and undergo a QC process prior to being circulated to the study team. See Appendix I for a template.
- The Qualitative Lead will track the status of the IDI and associated data in a qualitative IDI tracking log (See Appendix B).

**3.4 Participant Study Files**

Separate files will be created for participants selected for qualitative interviews to ensure confidentiality of their data. All documentation for participants participating in the qualitative component should be completed and filed Harmony Aim 3 study folder, in accordance with the main study procedures. Note that only files without identifying information will be maintained in this location. Harmony qualitative participants will maintain the same participant ID (PTID) as used in the primary study.

The following will be source documents for the qualitative component of Harmony:

- Qualitative informed consent
- Visit checklist
- Notes taken during qualitative data collection on interview guides and/or separate sheets of paper
- Final IDI debriefing reports
- Final IDI transcripts

**3.5 Form and Guide Supply**

All master Harmony IDI guides in English will be supplied by the Co-Investigator and will be translated into the Kannada and back-translated to confirm the translation quality. The study’s local community partner will also review all Kannada translations for understandability. These will be printed locally. The Qualitative Lead is responsible for maintaining an adequate supply of the current version of guides in the relevant language(s). One copy of previous versions of guides will be maintained in an archive, and all other copies should be destroyed.

**3.6 Site Data Management and Quality Control**

**Site Audio File Procedures and QC**

**Same day as interview, as soon as possible following the IDI:**

- The Interviewer will verify that the audio recorder properly recorded the session. The audio file will be copied onto the secure limited-access UCSF study-specific Box drive, labeled with the ID number of the participant according to the file naming convention (see below on p.8), and deleted from the recording device.
  - If the audio recording did not record the session, the interviewer will review the guide and expand the notes they have taken during the discussion to serve as an alternate transcript.

**NOTE on Audio File Destruction**: Audio files of IDIs are considered source documentation and will NOT be destroyed until directed by Harmony MPIs following study completion. Audio files will be retained for at least five years following the study completion. The destruction process will be the responsibility of the Site PI. Once complete, destruction will be documented in the study files via a certificate from the person appointed to supervise destruction of the study material and confirmed via email with the Contact PI and qualitative protocol lead / co-Investigator.

**Site DR Procedures and QC Timeline**

**Same day as interview:**

- The interviewer will document their notes electronically into the Debrief Report (DR) form (created in Microsoft Word) within 24 hours of the IDI session and will review their own work for completeness and clarity prior to sending to the Qualitative Lead.

**Within one week following the interview:**

- A second QC review will occur on site to correct data inconsistencies/errors, and to ensure accuracy and completion. This review will be done by the Qualitative Lead. This will include:
  - Review of appropriate visit checklist to ensure all required procedures were completed
    - Ensuring no participant identifiers other than the ID are present on the documents
    - Ensuring the ID is correct and consistent across all documents
    - Ensuring the Visit Date is correct, and is the same on all documents (unless in the case of split visit, in which case this will be documented in the comments section of the visit checklist)
    - Filename matches appropriate file naming convention (see below)
  - DRs:
    - All information in heading of DR is completed and accurate
    - All sections of the DR template are filled in
  - Nothing needs clarifying or correction, e.g.:
    - Typos that lead to ambiguous meaning: e.g. “sore the medication” vs. “store the medication” (track changes may be used for straightforward typo corrections)
    - Sentences or phrasing requiring clarification
    - Clarification of any local terms used that may not be understood
  - Queries will be noted in track change comment bubbles for interviewer response and correction
- If corrections are needed to the DR, the interviewers will correct or clarify any problems identified directly in the report text using track changes and confirm the status (e.g. ‘done’, ‘corrected’, ‘not needed’, etc.) of each query within the comment bubble.
- After internal QC of the DR, all comments and track changes will be removed by the Qualitative Lead so the document is clean when sending to the Co-Investigator (CI). The clean DR Word document will be uploaded to the CI via the UCSF study-specific Box drive. No data should be transferred to the CI via email.
- For the first five DRs per interviewer, the CI will conduct a third-level QC to ensure completeness and clarity. This review may require additional changes from the interviewer before finalization. These changes will follow procedures described above regarding the use of track changes.
- Following all QCs, a final DR will be shared with the protocol team in order to share initial learnings from the qualitative component that may inform the study implementation (quantitatively or qualitatively), and to inform future probing and theme generation, as well as tracking of information saturation. This will be done by the CI or Qualitative Lead.

File Naming Conventions: All data files should be named according to a standard naming format. The name should include the PTID, data type (audio file, debrief report, transcript), and the date the IDI was conducted. Each time a document is edited, the editor should add their initials to the filename without changing any other part of the filename. For the first iteration of the file that is uploaded on the UCSF study-specific Box drive for review and the Co-Investigator is alerted to its presence, there is no need to include the editor’s initials. It is only upon subsequent review (QCing) that this occurs. For example, when reviewed for the first time, “10010_ Transcript_18NOV25” would become “10010_ Transcript_18NOV25_MH” and “10010_Transcript_18NOV25_MH_MA” for the second revision. Once the document is finalized, all initials will be removed from the name by the CI and replaced with the word “FINAL.”

**Site Transcription Procedures and QC**

**Within one month following interview:**

- Following the IDI session, the audio-file will be used to transcribe the discussion. See Appendix J for transcription guidelines. All transcripts will be transcribed first into Kannada and then translated into English. Unique phrases or sayings in the local language will be preserved and accompanied by explanatory notes in brackets to explain their meanings. All expanded notes, if required due to a recording failure, will be written in English.

Quality checks of transcription/translation will be performed at the site as described below. This process will include the following:

- The transcriber will certify that the transcript is an authentic representation of the audio recording by adding their name and date to the top portion of the transcript.
- A second qualitative team member (i.e. an individual who did not translate the interview) who is fluent in the local language will listen to the entire audio file while reading the English transcript. The quality of **the first three** **transcripts** **per transcriber** will be checked in this manner to determine that the quality of translation is sufficient. If the quality is deemed insufficient, it is appropriate for the Qualitative Lead to do a more thorough checking of the subsequent three transcripts and provide feedback to the transcriber. These reviews will be continued in batches of three until the quality is acceptable for each transcriber.
- Following this determination, systematic quality checks will include listening to **at least three, 5-minute spots** per interview of the audio file as compared to the transcript for 10% of transcripts.
- The Qualitative Lead will **log the QC process** (including which transcripts were reviewed in their entirety and which were spot checked, and by whom). Prior to start of the study, the Qualitative Lead will send the template QC log to the CI.
- The text of each transcript will still be reviewed by a second staff member (who did not translate the interview) in its entirety, even if the entire audio file is not reviewed. This review will focus on completion, content clarity and typos. The transcript will also be reviewed for interviewing technique (e.g. adequacy of probing, appropriate linking of topics, fidelity to the guide, etc.). Feedback will serve to provide additional training to the interviewers and to improve the quality of data collected. **The name of the staff member who reviewed each transcript will be documented on the transcript heading.**

**Site Transcript QC Process**

Following the site level QC process, the English language transcript will be uploaded to UCSF study-specific Box drive for review by the CI). The CI will receive an English language transcript within **one month** (30 days) of the interview date. Transcripts will then undergo the following QC process:

- Each transcript will be reviewed by the CI and queries will be made on the transcript using Microsoft Word’s review feature. The QC may include the identification of the following:
  - Problems such as typos that lead to ambiguous meaning, confusing terms or missing/potentially incorrect data
  - Issues identified by the protocol team requiring follow up, additional probing, or discussion with the interviewers. This could include general findings related to discussion facilitation techniques or specific issues that will be teased apart further in future IDIs.
- CI-reviewed transcripts will be uploaded to the UCSF study-specific Box drive site **within** **approximately two weeks** of transcript receipt and an email will be sent to the Qualitative Lead to notify that it is available.
- Qualitative interviewers will respond to all comments **within two weeks** of receipt of the reviewed transcript. Responses will be made either through changes directly in the transcript using track changes and confirm the status (e.g. ‘done’, ‘corrected’, ‘not needed’, etc.) of each query within the comment bubble. When in-text changes are unable to be made, the site reviewer will respond through using the comment box in the reviewing mode of MS Word. The corrected transcript is then uploaded onto the UCSF study-specific Box drive.
- When the revised information is received, the CI will review the corrected issues and indicate whether the issue has been resolved or requires further follow-up. If further clarification is necessary, the CI will upload the transcript onto the UCSF study-specific Box drive again and email the site. This process continues with the site until all necessary changes are made on the transcript.
- Once the CI finds no additional issues, the CI will accept all changes, save a clean, final copy of the transcript on an encrypted drive, and will upload to the UCSF study-specific Box drive for the site’s records.
- The participant’s final English transcript, and the final local language transcript (if applicable) must all be stored in the participant’s file.

**3.6.1 Staff responsibility**

The table below designates the site staff member(s) responsible for the various tasks associated with site data quality control.

| **Responsibility** | **Job Title(s) of Staff Member(s) Responsible** |
| --- | --- |
| Training of site staff on audio file, debrief report & transcript QC process | Co-Investigator, Qualitative Lead |
| Audio file QC review | Qualitative Lead |
| Debrief report QC review | Qualitative Lead |
| Transcript QC review | Qualitative Lead |
| Periodic evaluation of QC processes | Co-Investigator, Project Manager, Qualitative Lead or designee |

**3.6.2 Timing of Data Transmission**

The table below designates the site staff member(s) responsible for the various tasks associated with quality control, data management, and data transmission to.

| **Responsibility** | **Job Title(s) of Staff Member(s) Responsible** |
| --- | --- |
| Ensure the proper QC reviews (i.e. audio file, internal DR QC, internal transcript QC) have occurred prior to uploading | Qualitative Lead |
| Ensure DRs and transcripts are uploaded within the time frames listed above | Qualitative Lead |
| Ensure uploaded files have been received at | Qualitative Lead |
| File documents into participant study files once finalized | Qualitative Lead |

**4.0 Ethical Issues**

Confidentiality is maintained through the use of IDs or pseudonyms on all data. All team members will take special precaution to ensure that no identifying information is including in any qualitative data uploaded to UCSF study-specific Box drive.

**5.0 Document Management, Filing and Archiving**

All study information must be stored in accordance with the study protocol, ethical and GCP requirements and any other relevant regulations or standards.

**6.0 Other Related Working Practices**

ICH Consolidated Guidance for Good Clinical Practice (ICH-E6)

Harmony Study Protocol, in particular sections:

C.3. Enrollment Procedures

C.7. Follow-up Assessments / retention

C.12. Participant Withdrawal, Loss to Follow-up or Death

Documentation

C.4. Baseline Assessment Procedures

C.7.5. Interview procedures for 3M, 6M, 9M, 12M F/U

C.8. Common Procedures Applicable for All Waves

E. Safety Protocol

**Section G: Appendices**

**Appendix A**

**Research Strategy**

**1. Significance**

1.1. Alcohol use disorder (AUD) and Intimate partner violence (IPV) are interconnected issues with significant adverse global public health implications.

Globally**,** an estimated 30% of women report physical or sexual violence by an intimate partner in their lifetime. An increasingly large body of evidence supports a causal relationship between alcohol use disorder (AUD) and intimate partner violence (IPV), defined as control, psychological, physical, and sexual violence by a close partner. AUD is associated with lowered inhibitions and distorted perception of cues resulting in increased aggression. There is growing consensus that AUD in one or both partners is associated with severity and frequency of violence globally, including in India.

In a 2017 national survey among ever-married women in India, the rate of women who experienced physical violence by a spouse was 31%, while multi-state estimates predict Indian women who have experienced some form of spousal abuse in their lifetime to be as high as 52%. Studies in India have found that women with spouses who drink alcohol are more likely to experience violent events. Women in India who report IPV victimization experience poor short- and long-term health outcomes including increased risk for sexually transmitted infections and HIV, poor maternal health outcomes, and increased attempted suicide risk. Given the pervasiveness of IPV and its strong association with AUD, evidence-based interventions are urgently needed that are culturally appropriate and tailored for different populations.

1.2. There is a dearth of effective and scalable interventions to target both AUD and IPV.

A 2019 global meta-analysis found very few interventions that integrate reduction in AUD and IPV, and among those, no study was successful in changing both target behaviors, highlighting a large scientific gap in this field. Even among those which have been successful at reducing either alcohol use or IPV, such interventions are delivered by mental health professionals, limiting their scalability. Interventions in the US have employed cognitive behavioral therapy (CBT) techniques using group and individual sessions delivered by trained masters level therapists to strengthen problem-solving and communication skills among convicted male offenders. These studies found a significant reduction in self-reported days of alcohol use and participants were less likely to report engaging in aggressive behaviors after drinking episodes but found no significant difference in reported frequency of physical IPV. Other studies in the US added substance use treatments in addition to IPV treatment as usual but did not find statistically significant changes in outcomes. In India, integrated interventions have also relied on using trained mental health professionals, limiting scalability. An Indian study among married males with alcohol dependence and a history of IPV found that using CBT to prevent aggressive behavior led to a statistically significant decrease in reducing IPV but not in alcohol use.

1.3. An effective intervention will need to include both partners, and target both AUD and IPV.

Evidence from global systematic reviews of AUD and IPV suggested two major gaps. First, most often, only the man was engaged in the intervention. Despite ongoing IPV, many women cannot or may not want to leave their partners. In such cases, working with couples on communication and conflict negotiation has been effective at reducing violence, suggesting that couples therapy may be more effective than individual treatment. The second gap is that most studies failed to integrate treatment for both AUD and IPV. Behavioral couple’s therapy (BCT), which is based on the principles of cognitive behavioral therapy (CBT) and improvement of interpersonal dynamics, is effective at reducing IPV. It is typically combined with a program to reduce alcohol use prior to beginning BCT counselling. Thus, an effective intervention needs to incorporate strategies to address both AUD and IPV, with a focus on couples.

1.4. Our pilot research found that behavioral couple’s therapy (BCT) can reduce both AUD and IPV.

In our pilot intervention conducted in Bengaluru, India, we combined BCT with contingency management to reduce AUD and IPV among 60 couples, thereby addressing the gap in the literature by including both partners and by combining treatments for both AUD and IPV. The results showed that the intervention was effective at reducing men’s alcohol use as measured by daily breathalyzer test and IPV based on the Indian Family Violence and Control Scale (IFVCS). Participant couples reported that the intervention was safe and highly acceptable. There was no increase in violence associated with the intervention and we were successful in recruiting and retaining vulnerable couples, which is a serious impediment in many studies on AUD and IPV.

As BCT often needs to be preceded by strategies to reduce perpetrator alcohol use, we will deliver BCT with Motivational Interviewing (MI), a robust intervention with substantial evidence in reducing AUD in multiple populations globally, including India. To ensure that this intervention has the highest likelihood of success and eventual scale-up, we propose to train nurses to deliver MI, as several studies have shown nurse-led MI to successfully reduce alcohol use and improve multiple other health outcomes. In India, MI has been used widely, including by paraprofessionals to reduce alcohol use, as assessed by Alcohol Use Disorders Identification Test (AUDIT). These findings demonstrate that in primary care settings where there is a shortage of mental health professionals, training nurses in MI is feasible and effective at reducing AUD. Our study will thus address the gaps in the current literature by combining MI and BCT to reduce AUD and IPV.

**2. Innovation**

The proposed study is innovative in four important ways:

2.1 Including both partners in the intervention: Most IPV interventions have focused on women while AUD interventions have only targeted men, but such strategies have multiple limitations. First, for IPV, this places all burden to improve the situation on women, who are already enduring violence. Second, women-only interventions risk further IPV increase after the husband finds out that his wife has been accessing services for IPV. Third, it is important to address both partners when they have few options to leave and desire to stay together. Our intervention focuses on both partners, which is novel and overcomes these limitations.

2.2. Presenting the intervention as a couples’ communication strategy is a novel approach to enhance acceptability of AUD and IPV services: Men who engage in IPV often normalize IPV behaviors and are therefore unlikely to participate in an IPV reduction intervention. Many women also avoid participating in IPV interventions because of the stigma inherent in admitting to IPV, fear that disclosure may result in worsening of violence, and a desire to maintain and protect the family unit. Our study presents the intervention as a way to promote a healthy relationship through communications counselling, rather than emphasizing the more stigmatizing topics of AUD and IPV. As shown in our pilot study, a husband who is unlikely to accept that he engages in violence or that his alcohol use is a problem, may be more likely to accept that the couple has problems with communication resulting in relationship challenges that hinder their family goals. This innovative approach can make it more acceptable to both men and women to participate in an intervention that ultimately reduces AUD and IPV.

2.3. Integration of MI and BCT: BCT has been shown to be effective in improving interpersonal dynamics while there is robust evidence that MI can effectively reduce alcohol use. However, to our knowledge, MI and BCT have not been integrated and tested together, further enhancing the innovative aspect of our proposed intervention. Such an integrated approach is essential to address the co-occurring deleterious consequences of AUD and IPV.

2.4. Delivering the intervention by nurses increases scalability: MI and BCT are traditionally delivered by mental health specialists, but our proposed intervention will be delivered by non-specialist, primary care nurses. There is a lack of mental health specialists around the world while the prevalence of the problems of AUD and IPV is high. Delivering the intervention via task-shifting, which uses existing non-specialist primary care clinic staff, is an innovative approach to scale up these services.

**3. Aim 1: Refine the MI + BCT intervention and finalize the study measures to prepare for a randomized controlled trial.**

The goal of this aim is to adapt 3 study measures and finalize the intervention protocol for the Aim 2 RCT.

**3.1 Finalize intervention protocol for MI+BCT.** We will finalize the intervention protocol for MI+BCT based on direct observation of study nurses delivering it to research staff in mock sessions. As described under Preliminary Studies above, our team has extensive experience training non-doctoral-level staff to successfully administer MI in this and similar populations. The original versions of all training manuals, guides, fidelity checklists and trainers from our previous studies using MI and BCT are already available. Note that this is not a pilot study, but that the main purpose of this part of Aim 1 will be to make any necessary refinements to ensure that the overall treatment fidelity remains intact when MI and BCT are delivered sequentially by the nurses in a combined intervention. For MI, we will use the Motivational Interviewing Treatment Integrity (MITI), a standard tool used widely for MI fidelity. For BCT, we will use a standard checklist that our team used previously in our pilot study. The study investigators will supervise a study counselor in using this fidelity monitoring checklist to directly observe 4 MI and 6 BCT sessions. For MI, we will document the MITI score and for BCT, we will document the proportion of relevant topics covered in each session and the quality of counseling (e.g., maintains focus, nonjudgmental delivery), interaction with participants to assess their engagement with the material, and any factors that may have affected implementation. Note that all staff will need to meet and maintain set performance criteria. If they fail to do so, they will be retrained, re-certified or, if they continue to fail to meet the criteria, they will be replaced.

**4. Aim 2: Conduct a randomized controlled trial to deliver motivational interviewing and behavioral couples therapy and assess impact on reducing AUD and IPV.**

**4.1 Study Procedure and Recruitment.**

a. Inclusion criteria: 1) married with both spouses age ≥18; 2) living within the catchment area of the PHC; 3) speaking Kannada; 4) wife reporting any physical or sexual IPV in the past 12 months (note exclusion for severe IPV below), and 5) the husband having AUD (AUDIT-C ≥4).

b. Exclusion criteria: 1) husband has severe alcohol dependence (per Severity of Alcohol Dependence Questionnaire, SADQ ≥ 31) or is at risk of severe withdrawal symptoms (Clinical Institute Withdrawal Assessment for Alcohol Scale–Revised - CIWA-AR); 2) significant medical problems that will make the couple unable to participate in the intervention sessions; 3) cognitive problems, (Short-Blessed Cognitive Test score ≤7; previously adapted for India and used successfully in our HOPE RCT); 4) past year history of IPV severe enough to result in hospitalization (per an adapted version of the International Violence Against Women Survey - IVAWS^101^), or 5) wife screens positive for AUD (AUDIT-C ≥4). Female AUD is very rare in India (0.5%, which is 1/20^th^ of AUD among men).^102^ The intervention addresses alcohol use in the perpetrator of violence and the pilot study was conducted among men who have AUD and engage in IPV. We will thus exclude and refer couples for treatment when the wife screens positive for AUD.

c. Recruitment: Our recruitment plan is guided by the successful process we used in the pilot study. Community health workers, called ASHA in India, will refer couples that they know are experiencing IPV and in which the husband uses alcohol. Following consultation with the research team, the ASHA will bring the woman to the clinic for a “women’s health” appointment, which is a routine part of ASHA’s work. Research staff will screen potential participants for the presence of IPV and AUD. If she meets eligibility criteria for both (see above for details), the wife will be asked if her husband drinks any alcohol. If the response is positive, she will be asked about the most appropriate way to engage the husband, either: a) the woman will bring him in the next visit; b) the research staff will call him directly and ask him to come to the clinic; or c) ask the ASHA to speak with the husband. In the next visit with the couple, the research team will screen the husband and administer the three-question AUDIT-C to screen for AUD (score ≥4), the SADQ to screen out participants (score ≥ 31) who are severely dependent and the CIWA-AR^99^ for patients who are at high risk of withdrawal, and therefore ineligible. Based on our prior experience, we conservatively estimate that we may need to screen 2,350 couples to enroll 400. We have experience successfully conducting large screenings, as evidenced by our HOPE trial also conducted in primary health clinics, in which we screened 24,950 individuals to enroll 2,500 diagnosed with both mental illness and either cardiovascular disease or diabetes. As detailed in the Human Ss section, study staff will provide a brief educational session and refer all participants who are ineligible for the study, but who screen positive for AUD and/or IPV to the National Institute of Mental Health and Neurosciences (NIMHANS) in Bangalore, a premier tertiary care system, which includes psychology and psychiatry services as well as a dedicated de-addiction treatment for severe alcohol dependence and alcohol withdrawal. Following informed consent to participate in the study, the research staff will enroll eligible couples in the study, conduct baseline assessments and randomly assign them to the intervention or enhanced usual care arm.

d. Interview Schedule and Procedures: Based on our prior experience, we estimate that interviews will last between 45-60 minutes. All interviews will be conducted in the local language (Kannada), and will be held in a private room, most likely at the clinic, but could also be at a different, mutually convenient location that provides privacy. All participants will be asked to provide contact information and complete an assessment team member-administered baseline questionnaire upon enrollment and will be reassessed quarterly via an assessment team member-administered questionnaire. Responses will be entered into a tablet-based version of the REDCap mobile app. The interviews will be conducted by experienced staff members who are fluent in Hindi and Kannada and who will undergo training and certification on interviewing techniques found successful in our previous research on sensitive topics with vulnerable Indian populations, including strategies to reduce social desirability bias, standard operating procedures, human subjects protections and the study protocol.

e. Randomization procedures: Couples will be randomly assigned to intervention or enhanced usual care, following baseline assessments, using a random number generator. The couple will come to the PHC for randomization and the first orientation session. During the orientation session, the randomization assignment will be revealed to the couple using a sealed envelope with their study identification number.

**4.2. Study Design**

The study will be a randomized controlled trial with couples randomized 1:1 to the control group (n=200 couples) and the intervention group (n=200 couples).

**Control arm (Enhanced Usual Care):** Baseline assessments from our previous studies have shown that usual care for AUD and IPV in primary health centers (PHC) is very limited and even when protocols exist, they are not followed. As such, to meet ethical parameters, the control arm will have enhanced usual care that will include: a) PHC clinicians (who will be trained by the research team) will conduct initial safety assessment for all participants; b) for IPV, PHC staff will refer them to a legal cell at NIMHANS, a one-stop IPV center, and inform participants of their options (including support for leaving the relationship if they wish) based on our study log describing local resources such as names and contact information for local NGOs that can provide legal advice, counseling, and shelters and; c) for AUD, a trained nurse will provide a brief educational intervention based on the World Health Organization’s manual for managing AUD in PHCs and will provide referral to NIMHANS, a tertiary care mental health and addictions treatment center that has a dedicated referral system arrangement with the PHCs.


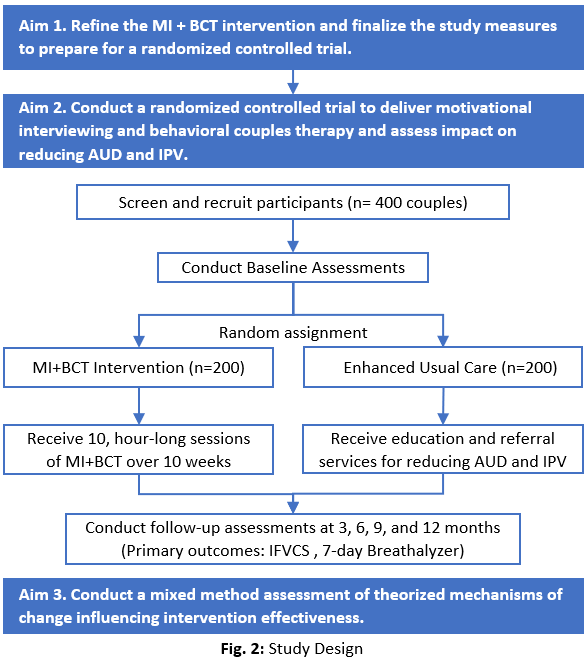


**Intervention arm:** Participants will receive MI to reduce their alcohol use and BCT to improve their interpersonal interactions to improve communication and reduce the incidence of IPV. Participants will engage in 10 hour-long sessions once a week, as summarized in Table 1. The husband will receive MI over 4 sessions, which is the standard primary care-based intervention to reduce AUD in community populations. Subsequently, both partners will receive BCT over the remaining 6 sessions covering the content found to be effective in the pilot study.

The MI sessions will evoke the specific individual, interpersonal, and social factors that are driving unhealthy behaviors with just the husband, with the end goal of establishing a “daily trust contract” during which he verbally commits to reduce his drinking and his wife acknowledges her support. The BCT sessions will include both partners.

**5. Aim 3: Conduct a mixed-methods assessment of theorized mechanisms of change influencing intervention effectiveness.**

To understand the processes through which the intervention reduces AUD and IPV, we will conduct a mixed-methods explanatory assessment of the intervention’s theorized mechanisms of change by combining data from the quantitative surveys (per Aim 2) and from serial in-depth interviews (IDIs) with 40 couples. Results will be triangulated to confirm the essential pathways of change and will be critical to explaining the overall effectiveness of the intervention and if deemed effective, contributing to future refinement of the intervention for scale-up.

**Appendix B**

**Scripts for ASHAs to orient potential female and male participants.**

ASHA Script for Women

Greetings! My name is ______ and I am an ASHA working along side the _______ PHC. I have come here on behalf of St. John’s Research Institute to tell you about their new research project called Harmony.

I have come here today to talk to you about the activities and goals of this study. The study has two main objectives: first, it attempts to provide supporting in reducing alcohol use among men. The second is to address the problems that arise between the husband and wife due to the husband’s alcohol use – problems such as disagreements, arguments, aggression and violence, etc. The study aims to improve the quality of relationship the couple, while also helping the husband cut down on his drinking.

Your benefits of partaking in the study are as follows:

1. by understanding the impact of alcohol on a person’s health, it might help in reducing alcohol consumption.

2. some participants will learn how to communicate better with their spouse, work on solving problems together and establish trust in their relationship with their spouse.

Your participation would be required for 1 year. The study requires that both you and your husband join together. I assure you that your safety and your privacy is of utmost importance, and it would be only natural if you are concerned about it because it is a matter related to alcohol and violence. We will ensure complete confidentiality of your identifying details from our side. There is no pressure to participate, and it is your choice if you wish to or not.

If you are willing to, I will ask you to come to _____ PHC on ______ for a women’s health appointment. When you get there, staff members of the study will tell you more about it in detail and ask if you would be willing to participate in the study. Only after that, we will include your husband in the study – we will get in touch with him in the manner that you prefer – such as you bringing him, or me contacting him, etc. which is for later.

Please feel free to think it over and then reach out to me or ask me any questions that you may have right now.

FAQ’s

1. Can you please ask my husband first, to check if he is agreeable to sign up. If he agrees, I shall consent.

We would like to contact your husband only if you are interested to participate. Therefore, right now we are trying to check if you are interested first. We will surely contact your husband to find out if he is interested, as this study requires only couples and individually you cannot take part. I wish to clarify that when we contact your husband we will not inform him about your consent, but will say that you are only interested to take part.

2. My husband may not agree to enrol, but a word from ____ might motivate him to agree. Can _____ please speak to my husband first?

Your decision or your husband’s decision to take part in this study is voluntary. Therefore, we do not wish for anybody to influence yours or his decision-making. If you are keen, we will tell your husband that you are interested and not tell him about you having given your consent.

3. What if my husband gets angry that I have expressed interest to be a part of the study?

We will tell your husband that you are only interested but not consented. You may tell him that participating in the study will depend on his approval first.

4. I am very keen to be a part of the study, but my husband may not agree. Can you tell me how I could convince my husband?

You may explain to him about benefits of the study, which is learning to reduce his alcohol consumption and to improve the relationship between the two of you. This can improve the overall environment at home for him as well as you. Other areas in which he may see improvement would be your financial situation, controlling anger, dealing with stress, health, relationship, and family wellbeing.

You may also tell him that we will be contacting him to give a detailed explanation of the study and he may take an independent decision about being enrolled or not after understanding the details.

5. How can I give my consent without talking to my husband first?

If you do give consent now, it does not mean that you will be enrolled in the study immediately. You may give your name now; however you are free to discuss it with your husband, and even get back to us if you decide that we should we not be contacting your husband in the next couple of days. We will inform your husband that you are interested but will not tell him that you are given your consent to participate. Please remember if there is any issue you may always feel free to contact me. I shall share my number with you.

6. How will you test the drinking of my husband?

We will give a machine to your husband and we will train him and you on how to use the machine at home itself, without having to go out. This would be only 3 times in a year and only for 1 week each time.

7. Traveling to the PHC will cost us a lot for the auto/bus…

The study will cover your expenses for traveling to and from the PHC.

8. I’m scared others will get to know about his drinking/that we have relationship problems…

As I have said earlier, your privacy and the confidentiality of your details and your participation is of utmost importance. The research staff and us, ASHAs and PHC staff as well, will never disclose or go around telling anyone about your participation in this study or what you tell us. This is a very strict guideline that follow, and we will do our best to uphold it.

9. How many times will we have to come to the PHC for this?

As I said earlier, once you come to the PHC for the appointment, the research staff will give you all the details about how often and when you will have to come. Nevertheless, I will tell you now as well – you will have to go to the PHC 4 times in one year for the research staff to ask you some questions. 3 times in the year, I or another ASHA will come to your home to measure your husband’s alcohol use. Some participants will have to come once a week for 10 weeks (2.5 months), and you will be told if you would be one of these participants. If you are not, then you will be coming to the PHC once in the beginning, then after 3 months, 6 months and at the end of the year.

Call for baseline assessment and randomization

Greetings! I am ______, an ASHA working with ______ PHC. Thank you for indicating your willingness to partake in the study and congratulations for starting this. As a first step, I request you to come to the PHC – the research staff will be asking you several questions for research purposes. It would go on for 1 hour. What would be a good day and time for you and your spouse?

Call for periodic outcome assessments

Greetings! I am ______, an ASHA working with ______ PHC. As you know, you have been participating in the Harmony study by SJRI. We would like you to come down to the PHC sometime this week for the research staff to conduct the regular assessments/tests. Like the first time, this will also go on for 1 hour. What would be a suitable day and time for you and your spouse? When you come here, please remember to NOT tell the research staff that your received counselling or did not.

**ಹಾರ್ಮನಿ ಪ್ರಾಜೆಕ್ಟ್**

**ASHA ರವರು ಮಹಿಳೆಯರಿಗೆ ಬಳಸುವಂತಹ ಸ್ಕ್ರಿಪ್ಟ್- Female**

ನಮಸ್ಕಾರ. ನನ್ನ ಹೆಸರು ______. ನಾನು _______ PHC ಯಲ್ಲಿ ASHA ಕಾರ್ಯಕರ್ತೆಯಾಗಿ ಕೆಲಸ ಮಾಡುತ್ತಿದ್ದೇನೆ. ನಾನು BBMP ಮತ್ತು St John 's ಆಸ್ಪತ್ರೆಯ ಹಾರ್ಮನಿ ಪ್ರೋಜೆಕ್ಟಿನ ಬಗ್ಗೆ ನಿಮಗೆ ತಿಳಿಸಲು ಬಂದಿದ್ದೇನೆ.

ಈಗ ನಿಮಗೆ ನಾನು ಈ ಪ್ರಾಜೆಕ್ಟ್ನ ಗುರಿಗಳ ಬಗ್ಗೆ ಹೇಳುತ್ತೇನೆ.

1: ಸಮಾಲೋಚನೆ ಸಭೆಗಳ ಮೂಲಕ ಗಂಡಸರ ಕುಡಿತದ ಅಭ್ಯಾಸವನ್ನು ಕಡಿಮೆ ಮಾಡುವುದು.

2: ಸಮಾಲೋಚನೆಯ ಸಭೆಗಳ ಮೂಲಕ ಗಂಡ ಹೆಂಡತಿಯ ಸಂಬಂಧವನ್ನು ಉತ್ತಮಗೊಳ್ಳಿಸುವುದು. ಗಂಡ-ಹೆಂಡತಿ ನಡುವೆ ಮಾತು-ಕಥೆ, ವಿಶ್ವಾಸ ಇತ್ಯಾದಿ ವಿಚಾರಗಳಲ್ಲಿ ಬದಲಾವಣೆ ತರುವ ವಿಧಾನಗಳನ್ನು ಚರ್ಚಿಸಿ ಮನೆಯ ವಾತಾವರಣವನ್ನು ಸುಖಕರ ಮಾಡುವುದು.

ಈ ಅಧ್ಯಯನದಲ್ಲಿ ಭಾಗವಹಿಸಿದರೆ ನಿಮಗೆ ಏನು ಲಾಭ ಇರಬಹುದೆಂದು ಹೇಳುತ್ತೇನೆ:

1. ಕುಡಿತದ ಕಾರಣ ಒಬ್ಬರ ಆರೋಗ್ಯದ ಮೇಲೆ ಏನೆಲ್ಲಾ ಕೆಟ್ಟ ಪ್ರಭಾವ ಬೀಳುತ್ತದೆ ಎಂದು ಅರ್ಥ ಮಾಡಿಕೊಂಡು, ನಿಮ್ಮ ಗಂಡನ ಕುಡಿತದ ಅಭ್ಯಾಸವನ್ನು ಕಡಿಮೆಮಾಡುವುದಕ್ಕೆ ಬೇರೆ-ಬೇರೆ ವಿಧಾನಗಳನ್ನು ತಿಳಿದುಕೊಳ್ಳುವುದು. ಈ ರೀತಿಯಲ್ಲಿ ನಿಮ್ಮ ಗಂಡ ಅವರ ಆರೋಗ್ಯವನ್ನು ಚನ್ನಾಗಿ ನೋಡಿಕೊಳ್ಳಬಹುದು.

2. ಕೆಲವು ಭಾಗವಹಿಸುವವರು ಅವರ ಗಂಡನೊಂದಿಗೆ ಒಳ್ಳೆಯ ಮಾತು-ಕಥೆ ಮಾಡುವುದು, ವಿಶ್ವಾಸದಿಂದ ಇರಲು, ಮತ್ತು ಸಮಸ್ಯೆಗಳನ್ನು ಪರಿಹರಿಸುವುದನ್ನು ಕಲಿತುಕೊಳ್ಳುತ್ತಾರೆ. ಒತ್ತಡದ ಪರಿಸ್ಥಿತಿಗಳನ್ನು ಹೇಗೆ ಒಟ್ಟಿಗೆ ಎಧುರಿಸುವುದು, ಕೋಪ ಮತ್ತು ಹಗೆತನದ ಭಾವನೆಗಳನ್ನು ಹೇಗೆ ಕಡಿಮೆಮಾಡುವುದು ಮತ್ತು ಮನಸ್ಸನ್ನು ಶಾಂತಿ ಮಾಡಿಕೊಳ್ಳುವುದು ಹೇಗೆ ಎಂದು ತಿಳಿಸಿ ಕೊಡಲಾಗುತ್ತದೆ.

ಈ ಅಧ್ಯಯನದಲ್ಲಿ ನಿಮ್ಮ ಭಾಗವಹಿಸುವಿಕೆ 1 ವರ್ಷದ ಕಾಲದಷ್ಟುಇರುತ್ತದೆ. ಇದರಲ್ಲಿ ನೀವು ಮತ್ತು ನಿಮ್ಮ ಗಂಡ ಇಬ್ಬರೂ ಒಟ್ಟಿಗೆ ಭಾಗವಹಿಸುವುದು ಬಹಳ ಮುಖ್ಯ. ಇದರಲ್ಲಿ ನೀವು ಒಬ್ಬರೇ ಅಥವಾ ಗಂಡ ಒಬ್ಬರೇ ಭಾಗವಹಿಸುವುದು ಸಾಧ್ಯವಿಲ್ಲ. ನೀವು ದಂಪತಿಗಳಾಗಿ ಭಾಗವಹಿಸ ಬೇಕು.

ಈ ಅಧ್ಯಯನದಲ್ಲಿ ನಿಮ್ಮ ಎಲ್ಲ ವಿವರಗಳನ್ನು ಗೌಪ್ಯವಾಗಿ ಇಡಲಾಗುತ್ತದೆ. ನಾವು ಮತ್ತು ಅಧ್ಯಯನದ ಸಿಬ್ಬಂದಿಗಳು ನಿಮ್ಮ ಭಾಗವಹಿಸುವಿಕೆಯ ಬಗ್ಗೆ ಮತ್ತು ನಿಮ್ಮ ವಿವರಗಳನ್ನು ಭಾಗವಹಿಸದ ಇತರರಿಗೆ ಹೇಳುವುದಿಲ್ಲ.

ಭಾಗವಹಿಸುವುದಕ್ಕೆ ಏನೂ ಒತ್ತಾಯಇಲ್ಲ. ನೀವು ಭಾಗವಹಿಸುತ್ತೀರೋ ಇಲ್ಲವೋ ಎಂಬುದು ನಿಮ್ಮ ಸ್ವಂತ ಇಚ್ಛೆ.

ನೀವು ಭಾಗವಹಿಸಲು ಬಯಸಿದ್ದರೆ ನಿಮ್ಮೊಂದಿಗೆ ಚರ್ಚಿಸಿದ ದಿನಾಂಕ ನಿಮ್ಮನ್ನು PHC ಗೆ ಬರಲು ಕರೆಯುತ್ತೇವೆ. ನೀವು PHC ಗೆ ಬಂದಾಗ ಅಧ್ಯಯನದ ಸಿಬ್ಬಂದಿಗಳು ನಿಮಗೆ ಇನ್ನೂ ಮಾಹಿತಿ ನೀಡುತ್ತಾರೆ.

ಅದಾದ ನಂತರ ನಿಮ್ಮ ಗಂಡನನ್ನು ನಾವು PHC ಗೆ ನಿಮ್ಮಇಚ್ಛೆಯಂತೆ ಅಥವಾ ನೀವು ಬಯಸುವ ವಿಧಾನದಲ್ಲಿ ಕರೆದುಕೊಂಡು ಬರುತ್ತೇವೆ.

ನೀವು ಈ ಅಧ್ಯಯನದಲ್ಲಿ ಭಾಗವಹಿಸುವ ಬಗ್ಗೆ ಸ್ವಲ್ಪ ಯೋಚಿಸಿದ ನಂತರ ನಿಮ್ಮ ನಿರ್ಧಾರವನ್ನು ನಮಗೆ ತಿಳಿಸಬಹುದು.

ನಿಮಗೆ ಏನಾದರೂ ಪ್ರಶ್ನೆ ಇದ್ದರೆ ನನ್ನ ಹತ್ತಿರ ಕೇಳಲು ಹಿಂಜರಿಯಬೇಡಿ.

**1. ನನ್ನ ಗಂಡ ಒಪ್ಪಿಕೊಂಡರೇನೇ ನಾನು ಒಪ್ಪುತ್ತೇನೆ.**

ನೀವು ಭಾಗವಹಿಸಲು ಬಯಸುತ್ತೀರಿ ಎಂದು ತಿಳಿಸಿದ ನಂತರವೇ ನೀವು ಹೇಳಿದ ರೀತಿಯಲ್ಲಿ ನಿಮ್ಮ ಗಂಡನನ್ನು ನಾವು ಸಂಪರ್ಕಿಸುತ್ತೇವೆ. ನಿಮ್ಮ ಗಂಡನ ಹತ್ತಿರ ನೀವು ಭಾಗವಹಿಸಲು ಒಪ್ಪಿದ್ದೀರಿ ಎಂದು ಹೇಳುವುದಿಲ್ಲ. ನಿಮಗೆ ಕೇವಲ ಭಾಗವಹಿಸಲು ಮನಸ್ಸಿದೆ ಎಂದು ತಿಳಿಸುತ್ತೇವೆ. ಅವರೂ ಭಾಗವಹಿಸಲು ಬಯಸ್ಸಿದ್ದರೇನೇ ನಾವು ನಿಮ್ಮಿಬ್ಬರಿಂದಲೂ ಒಪ್ಪಿಗೆಯನ್ನು ತೆಗೆದುಕೊಳ್ಳುತ್ತೇವೆ.

**2.ನನ್ನ ಗಂಡನ ಕುಡಿತದ ಅಳತೆ ನೀವು ಹೇಗೆ ಮಾಡುತ್ತೀರಿ?**

ನೀವು ನಿಮ್ಮ ಗಂಡನ ಜೊತೆ PHC ಗೆ ಬಂದಾಗ ಅಲ್ಲಿರುವ ಅಧ್ಯಯನದ ಸಿಬ್ಬಂದಿಗಳು ನಿಮ್ಮ ಗಂಡನ ಕುಡಿತದ ಅಭ್ಯಾಸದ ಬಗ್ಗೆ ಹಲವಾರು ಪ್ರಶ್ನೆಗಳನ್ನು ಕೇಳುತ್ತಾರೆ.ಈ ರೀತಿಯಲ್ಲಿ, ಅಧ್ಯಯನ ಸಿಬ್ಬಂದಿ ನಿಮ್ಮ ಗಂಡನ ಕುಡಿತವನ್ನು ಅರ್ಥಮಾಡಿಕೊಳ್ಳಲು ನಿಮ್ಮನ್ನು ಒಂದು ವರ್ಷದಲ್ಲಿ 4 ಸಲ PHC ಗೆ ಕರೆಯುತ್ತಾರೆ.

ಅದಾದ ಮೇಲೆ ಕುಡಿತವನ್ನುಅಳತೆ ಮಾಡುವ ಒಂದು ಯಂತ್ರವನ್ನು ನಾವು ನಿಮ್ಮ ಮನೆಗೆ ತರುತ್ತೇವೆ. ನಿಮ್ಮ ಗಂಡನಿಗೆ ಮತ್ತು ನಿಮಗೆ ಅದನ್ನು ಹೇಗೆ ಬಳಸಬೇಕೆಂದು ಹೇಳಿ ಕೊಡುತ್ತೇವೆ. ಆ ಯಂತ್ರವನ್ನು ಬಳಸಿ ನಮಗೆ ನಿಮ್ಮ ಗಂಡನ ಕುಡಿತದ ಪ್ರಮಾಣ ಎಷ್ಟು ಇದೆ ಎಂದುತಿಳಿದುಕೊಳ್ಳುತ್ತೇವೆ. ಒಂದು ವರ್ಷದಲ್ಲಿ 3 ಸಲ ಈ ಯಂತ್ರವನ್ನು ಬಳಸ ಬೇಕು. ಅದಕ್ಕಾಗಿ, ನಾವು ನಿಮ್ಮ ಮನೆಗೆ ಬಂದು ಈ ಯಂತ್ರವನ್ನು ಕೊಡುತ್ತೇವೆ.

**3. PHC ವರಗೆ ಹೋ**ಗಿ **ಬರುವುದಕ್ಕೆ ಬಹಳ ಖರ್ಚು ಆಗಿ ಬಿಡುವುತ್ತದೆ...**

PHC ಗೆಹೋಗಿ ಬರಲು ಪ್ರಯಾಣಕ್ಕೆ ಆಗುವ ಖರ್ಚನ್ನು ಪ್ರಾಜೆಕ್ಟ್ಕ ಕಡೆಯಿಂದ ಮರುಪಾವತಿ ಮಾಡಲಾಗುತ್ತದೆ.

**4. ನಾನು ಈ ತರದ ಅಧ್ಯಯನದಲ್ಲಿ ಭಾಗವಹಿಸುತ್ತಿದ್ದೇನೆ ಎಂದು/ನನ್ನಗಂಡನಿಗೆ ಕುಡಿಯುವ ಅಭ್ಯಾಸ ಇದೆ ಎಂದು/ನನ್ನ ಸಂಬಂಧಗಳಲ್ಲಿ ಸಮಸ್ಯೆಗಳಿವೆ ಎಂದು ಬೇರೆಯವರಿಗೆ (ಅಧ್ಯಯನದಲ್ಲಿ ಇಲ್ಲದವರಿಗೆ) ಗೊತ್ತಾದರೆ ಎಂದು ನನಗೆ ಭಯ ಆಗುತ್ತದೆ...**

ನಾನು ಹೇಳಿದ ಹಾಗೆ ನಿಮ್ಮ ಎಲ್ಲ ವಿವರಗಳನ್ನು ನಾವು ಖಡಾಖಂಡಿತವಾಗಿ ಗೌಪ್ಯವಾಗಿ ಇಡುತ್ತೇವೆ. ಎಲ್ಲಾ ಅಧ್ಯಯನದ ಸಿಬ್ಬಂದಿಗಳು ಮತ್ತು ನಾವು ASHA ಕಾರ್ಯಕರ್ತೆಯರು ನಿಮ್ಮ ಯಾವುದೇ ವಿವರಗಳನ್ನು ಯಾರಿಗೂ ಹೇಳುವುದಿಲ್ಲ.

**5.ಇದಕ್ಕೋಸ್ಕರ ನಾವು ಎಷ್ಟು ಸಲ PHC ಗೆ ಬರಬೇಕಾಗತ್ತೆ?**

ಅಧ್ಯಯನದಲ್ಲಿ ನಿಮಗೆ ಸಮಾಲೋಚನೆಗೆ ಅವಕಾಶ ಕೊಡಲಾಗಿದ್ದರೆ ನೀವು ಒಂದು ವರ್ಷದಲ್ಲಿ 15 ಸಲ PHC ಗೆ ಬರ ಬೇಕಾಗತ್ತೆ. ಇದರಲ್ಲಿ 10 ಸಲ ಸಭೆಗಳಿಗೆ ಬರುವುದು, ಮತ್ತು ಉಳಿದ 5 ಸಲ ನಿಮ್ಮ ಸಂಬಂಧ ಮತ್ತು ನಿಮ್ಮ ಗಂಡನಿಗೆ ಕುಡಿತವನ್ನುಅಳತೆ ಮಾಡುವುದರ ಬಗ್ಗೆ ಕೇಳುವುದಕ್ಕಾಗಿ ಬರಬೇಕಾಗುತ್ತದೆ.

ನಿಮಗೆ ಸಮಾಲೋಚನೆಗೆ ಅವಕಾಶ ಇಲ್ಲದಿದ್ದರೆ, ಒಂದು ವರ್ಷದಲ್ಲಿ 6 ಸಲ ಬರಬೇಕಾಗತ್ತೆ. ಇದರಲ್ಲಿ 1 ಸಲ ನಿಮ್ಮ ಕುಡಿತದ ಬಗ್ಗೆ ನಿಮಗೆ ಸ್ವಲ್ಪ ಮಾಹತಿ ಮತ್ತು ಸಹಾಯ ನೀಡಲು ಮತ್ತು ಉಳಿದ 5 ಸಲ ನಿಮ್ಮ ಸಂಬಂಧ ಮತ್ತು ಕುಡಿತವನ್ನು ಅಳತೆ ಮಾಡುವುದರ ಬಗ್ಗೆ ಪ್ರಶ್ನೆಗಳನ್ನು ಕೇಳುವುದಕ್ಕಾಗಿ ಬರಬೇಕಾಗುತ್ತದೆ. ಇದರ ಬಗ್ಗೆ ಇನ್ನೂ ಹೆಚ್ಚಿನ ಮಾಹಿತಿ ಅಧ್ಯಯನದ ಸಿಬ್ಬಂದಿಗಳು ನಿಮಗೆ PHC ನಲ್ಲಿ ನೀಡುತ್ತಾರೆ.

**6. ನನ್ನ ಗಂಡ ಅಧ್ಯಯನದಲ್ಲಿ ಭಾಗವಹಿಸುವುದು ಕಷ್ಟ. ಆದರೆ ನೀವು ಅಥವಾ ಬೇರೆಯವರು ಅವರೊಂದಿಗೆ ಮಾತನಾಡಿ ಮನವೊಲಿಸಿದರೆ ಅವರು ಒಪ್ಪಿಕೊಳ್ಳುತ್ತಾರೆ...**

ಈ ಅಧ್ಯಯನದಲ್ಲಿ ಭಾಗವಹಿಸುವುದು ನಿಮ್ಮ ಮತ್ತು ನಿಮ್ಮ ಗಂಡನ ಸ್ವಂತ ಇಚ್ಛೆ. ಅದಕ್ಕಾಗಿ ಅವರ ಮತ್ತು ನಿಮ್ಮ ನಿರ್ಧಾರದ ಮೇಲೆ ಬೇರೆ ಯಾರ ಪ್ರಭಾವ ಬೇಡ. ಬೇಕಿದ್ದರೆ ನಿಮ್ಮ ಗಂಡನ ಹತ್ತಿರ ನಿಮಗೆ ಭಾಗವಹಿಸಲು ಮನಸ್ಸಿದೆ ಆದರೆ ಒಪ್ಪಿಗೆ ಇನ್ನೂ ಕೊಟ್ಟಿಲ್ಲ ಎಂದು ಹೇಳುತ್ತೇವೆ.

**7. ಈ ಅಧ್ಯನದಲ್ಲಿ ನನಗೆ ಭಾಗವಿಸುದಕ್ಕೆ ಮನಸ್ಸಿದೆ ಎಂದು ಗಂಡನಿಗೆ ಹೇಳಿದಾಗ ಅವರು ಕೋಪಗೊಂಡರೆ ಏನು ಮಾಡುವುದು?**

ನಿಮಗೆ ಭಾಗವಹಿಸಲು ಮನಸ್ಸಿದೆ ಆದರೆ ಒಪ್ಪಿಗೆ ಇನ್ನೂ ಕೊಟ್ಟಿಲ್ಲ ಎಂದು ನಾವು ನಿಮ್ಮ ಗಂಡನಿಗೆ ತಿಳಿಸುತ್ತೇವೆ. ಅವರು ಭಾಗವಹಿಸಲು ಒಪ್ಪಿಗೆ ತಿಳಿಸಿದ ನಂತರವೇ ನೀವು ಒಪ್ಪುತ್ತೀರಿ ಎಂದು ನಿಮ್ಮ ಗಂಡನಿಗೆ ಹೇಳಬಹುದು.

**8. ಈ ಅಧ್ಯಯನದಲ್ಲಿ ಭಾಗವಹಿಸಲು ನನಗೆ ತುಂಬಾ ಮನಸ್ಸಿದೆ. ಆದರೆ ನನ್ನಗಂಡ ಒಪ್ಪುವುದು ಕಷ್ಟ ಎಂದು ನನಗೆ ಅನಿಸುತ್ತದೆ. ಅವರು ಒಪ್ಪಿಗೆ ಕೊಡಲು ನಾನು ಅವರನ್ನು ಹೇಗೆ ಮನವರಿಕೆ ಮಾಡಲಿ?**

ಅವರಿಗೆ ನೀವು ಈ ಯೋಜನೆಯ ಗುರಿಗಳನ್ನು ವಿವರಿಸ ಬಹುದು:

1. ಮದ್ಯದ ಬಳಕೆಯನ್ನು ಕಡಿಮೆ ಮಾಡುವುದು ಮತ್ತು

2. ನಿಮ್ಮ ಸಂಬಂಧವನ್ನು ಉತ್ತಮಗೊಳ್ಳಿಸುವುದು.

- ಇದರಿಂದ ಮನೆಯ ವಾತಾವರಣ ಸುಖಕರವಾಗ ಬಹುದು
- ಖರ್ಚು ಕಡಿಮೆ ಮಾಡಿ, ಉಳಿತಾಯ ಜಾಸ್ತಿ ಆಗಿ ನಿಮ್ಮ ಆರ್ಥಿಕ ಸ್ಥಿತಿ ಒಳ್ಳೆಯದಾಗಬಹುದು
- ಸಂಬಂಧಗಳು ಉತ್ತಮಗೊಳ್ಳಿಸ ಬಹುದು
- ಒತ್ತಡವನ್ನು ಹೇಗೆ ನಿಭಾಯಿಸುವುದು ಎಂದು ಕಲಿತು ಕೊಳ್ಳಬಹುದು
- ಕೋಪ ಮತ್ತು ಹಗೆತನದ ಭಾವನೆಗಳನ್ನು ನಿಯಂತ್ರಿಸಲು ಕಲಿತುಕೊಳ್ಳಬಹುದು.

ನಾವುಅಥವಾಅಧ್ಯಯನದಸಿಬ್ಬಂದಿಗಳುಅವರಿಗೆ ಈಅಧ್ಯಯನದಬಗ್ಗೆಮಾಹಿತಿಕೊಡುತ್ತೇವೆಎಂದುಅವರಿಗೆನೀವುತಿಳಿಸಬಹುದು.

ASHA Script for Men

**First contact**

Greetings! My name is ______ and I am an ASHA working alongside the _______ PHC. I have come here on behalf of St. John’s Research Institute to tell you about their new research project called Harmony.

I have come here today to talk to you about the activities and goals of this study. The study has two main objectives: first, it attempts to provide supporting in reducing alcohol use among men. When people drink, their relationships with their spouses can be affected. So, the second objective of the study to is to help couples to work on their relationship difficulties by improving communication, trust, and problem-solving. The study aims to improve the quality of relationship the couple, while also helping the husband cut down on his drinking.

Your benefits of partaking in the study are as follows:

1. by understanding the impact of alcohol on a person’s health, it might help in reducing alcohol consumption. You may be helped to find ways of cutting down on your drinking and thereby take better care of your health.

2. some participants will learn how to communicate better with their spouse, work on solving problems together and establish trust in their relationship with their spouse. Couples may be taught how to deal with stressful situations better together, how to control difficult emotions like anger, and skills to relax as well.

Your participation would be required for 1 year. The study requires that both you and your husband join together. Individuals cannot take part in this; you may partake only as a couple.

We will ensure complete confidentiality of your identifying details from our side, so that nobody outside the study knows about your participation, drinking or how your relationship with your wife is. I assure you, there is no pressure to participate, and it is your choice if you wish to or not.

I had contacted and spoken to your wife about this earlier and she expressed her interest to take part [but she has not formally consented]. You can take your time and think it over, and let me know if you would also be interested in participating.

Please feel free to ask any doubts or questions that you may have about this.

***FAQ’s***

***1. Just by cutting down drinking, how will I really benefit from it?***

*As I said, this study does focus on helping you cut down on your drinking. You may also learn to improve the relationship between you and your wife. This can improve the overall environment at home for you, your wife and the rest of your family. Other areas in which you may see improvement would be your financial situation, controlling anger, dealing with stress, health, relationship, and family wellbeing.*

*If you are willing and come down to our PHC, the research staff will give you a detailed explanation of the study and you may then take an independent decision about being enrolled or not after understanding the details.*

***6. How will you test my drinking?***

*We will give a machine to you, and we will train you and your wife on how to use the machine at home itself, without having to go out. This would be only 3 times in a year and only for 1 week each time.*

***7. Traveling to the PHC will cost us a lot for the auto/bus…***

*The study will cover your expenses for traveling to and from the PHC.*

***8. I’m scared others will get to know about my drinking/that I have relationship problems…***

*As I have said earlier, your privacy and the confidentiality of your details and your participation is of utmost importance. The research staff and us, ASHAs and PHC staff as well, will never disclose or go around telling anyone about your participation in this study or what you tell us. This is a very strict guideline that follow, and we will do our best to uphold it.*

***9. How many times will we have to come to the PHC for this?***

*As I said earlier, once you come to the PHC for the appointment, the research staff will give you all the details about how often and when you will have to come. Nevertheless, I will tell you now as well – you will have to go to the PHC 4 times in one year for the research staff to ask you some questions. 3 times in the year, I or another ASHA will come to your home to measure your husband’s alcohol use. Some participants will have to come once a week for 10 weeks (2.5 months), and you will be told if you would be one of these participants. If you are not, then you will be coming to the PHC once in the beginning, then after 3 months, 6 months and at the end of the year.*

**Call for baseline assessment and randomization**

Greetings! I am ______, an ASHA working with ______ PHC. Thank you for indicating your willingness to partake in the study and congratulations for starting this. As a first step, I request you to come to the PHC – the research staff will be asking you several questions for research purposes. It would go on for 1 hour. What would be a good day and time for you and your spouse?

**Call for periodic outcome assessments**

Greetings! I am ______, an ASHA working with ______ PHC. As you know, you have been participating in the Harmony study by SJRI. We would like you to come down to the PHC sometime this week for the research staff to conduct the regular assessments/tests. Like the first time, this will also go on for 1 hour. What would be a suitable day and time for you and your spouse? When you come here, please remember to NOT tell the research staff that your received counselling or did not.

**ಹಾರ್ಮನಿ ಪ್ರಾಜೆಕ್ಟ್**

**ASHA ರವರು ಪುರುಷರಿಗೆ ಬಳಸುವಂತಹ ಸ್ಕ್ರಿಪ್ಟ್ - Male**

ನಮಸ್ಕಾರ. ನನ್ನ ಹೆಸರು ______. ನಾನು _______ PHC ಯಲ್ಲಿ ASHA ಕಾರ್ಯಕರ್ತೆಯಾಗಿ ಕೆಲಸ ಮಾಡುತ್ತಿದ್ದೇನೆ. ನಾನು BBMP ಮತ್ತು St John 's ಆಸ್ಪತ್ರೆಯ ಹಾರ್ಮನಿ ಎಂಬ ಪ್ರೋಜೆಕ್ಟಿನ ಬಗ್ಗೆ ನಿಮಗೆ ತಿಳಿಸಲು ಬಂದಿದ್ದೇನೆ. ಈಗ ನಿಮಗೆ ನಾನು ಈ ಪ್ರಾಜೆಕ್ಟ್ ನ ಗುರಿಗಳು ಏನು ಎಂದು ಹೇಳುತ್ತೇನೆ.

1: ಸಮಾಲೋಚನೆಯ ಸಭೆಗಳ ಮೂಲಕ ಗಂಡಸರ ಕುಡಿತದ ಅಭ್ಯಾಸವನ್ನು ಕಡಿಮೆ ಮಾಡುವುದು.

2: ಸಮಾಲೋಚನೆಯ ಸಭೆಗಳ ಮೂಲಕ ಗಂಡ ಹೆಂಡತಿಯ ಸಂಬಂಧವನ್ನು ಉತ್ತಮಗೊಳ್ಳಿಸುವುದು. ಗಂಡ-ಹೆಂಡತಿ ನಡುವೆ ಮಾತು-ಕಥೆ, ವಿಶ್ವಾಸ ಇತ್ಯಾದಿ ವಿಚಾರಗಳಲ್ಲಿ ಬದಲಾವಣೆ ತರುವ ವಿಧಾನಗಳನ್ನು ಚರ್ಚಿಸಿ ಮನೆಯ ವಾತಾವರಣವನ್ನು ಸುಖಕರ ಮಾಡುವುದು.

ಈ ಅಧ್ಯಯನದಲ್ಲಿ ಭಾಗವಹಿಸಿದ್ದರೆ ನಿಮಗೆ ಏನು ಲಾಭ ಇರಬಹುದೆಂದು ಹೇಳುತ್ತೇನೆ:

1. ಕುಡಿತದ ಕಾರಣ ಒಬ್ಬರ ಆರೋಗ್ಯದ ಮೇಲೆ ಏನೆಲ್ಲಾ ಕೆಟ್ಟ ಪ್ರಭಾವ ಬೀಳುವುತ್ತದೆ ಎಂದು ಅರ್ಥಮಾಡಿಕೊಂಡು, ಕುಡಿತದ ಅಭ್ಯಾಸವನ್ನು ಕಡಿಮೆ ಮಾಡುವುದಕ್ಕೆ ಬೇರೆ-ಬೇರೆ ವಿಧಾನಗಳನ್ನು ತಿಳಿದುಕೊಳ್ಳಬಹುದು. ಈ ರೀತಿಯಲ್ಲಿ ನೀವು ನಿಮ್ಮ ಆರೋಗ್ಯವನ್ನು ಚನ್ನಾಗಿ ನೋಡಿಕೊಳ್ಳಬಹುದು.

2. ಕೆಲವು ಭಾಗವಹಿಸುವವರು ಅವರ ಹೆಂಡತಿಯೊಂದಿಗೆ ಒಳ್ಳೆಯ ಮಾತು-ಕಥೆ ಮಾಡುವುದು, ವಿಶ್ವಾಸದಿಂದ ಇರಲು, ಮತ್ತು ಸಮಸ್ಯೆಗಳನ್ನು ಪರಿಹರಿಸುವುದನ್ನು ಕಲಿತುಕೊಳ್ಳುತ್ತಾರೆ. ಒತ್ತಡದ ಪರಿಸ್ಥಿತಿಗಳನ್ನು ಹೇಗೆ ಒಟ್ಟಿಗೆ ಎಧುರಿಸುವುದು, ಕೋಪ ಮತ್ತು ಹಗೆತನದ ಭಾವನೆಗಳನ್ನು ಹೇಗೆ ಕಡಿಮೆ ಮಾಡುವುದು ಮತ್ತು ಮನಸ್ಸನ್ನು ಶಾಂತಿ ಮಾಡಿಕೊಳ್ಳುವುದು ಹೇಗೆ ಎಂದು ತಿಳಿಸಿಕೊಡಲಾಗುತ್ತದೆ.

ಈ ಅಧ್ಯಯನದಲ್ಲಿ ನಿಮ್ಮ ಭಾಗವಹಿಸುವಿಕೆ 1 ವರ್ಷದ ಕಾಲದಷ್ಟು ಇರುತ್ತದೆ. ಇದರಲ್ಲಿ ನೀವು ಮತ್ತು ನಿಮ್ಮ ಹೆಂಡತಿ ಇಬ್ಬರೂ ಒಟ್ಟಿಗೆ ಭಾಗವಹಿಸುವುದು ಬಹಳ ಮುಖ್ಯ. ಇದರಲ್ಲಿ ನೀವು ಒಬ್ಬರೇ ಅಥವಾ ನಿಮ್ಮ ಹೆಂಡತಿ ಒಬ್ಬರೇ ಭಾಗವಹಿಸುವುದು ಸಾಧ್ಯವಿಲ್ಲ. ನೀವು ದಂಪತಿಗಳಾಗಿ ಭಾಗವಹಿಸಬೇಕು.

ಈ ಅಧ್ಯಯನದಲ್ಲಿ ನಿಮ್ಮ ಎಲ್ಲ ವಿವರಗಳನ್ನು ಗೌಪ್ಯವಾಗಿ ಇಡಲಾಗುತ್ತದೆ. ನಾವು ನಿಮ್ಮ ಭಾಗವಹಿಸುವಿಕೆಯ ಬಗ್ಗೆ ಮತ್ತು ನಿಮ್ಮ ವಿವರಗಳನ್ನು ಭಾಗವಹಿಸದ ಇತರರೊಂದಿಗೆ ಹೇಳಿಕೊಳ್ಳುವುದಿಲ್ಲ.

ಭಾಗವಹಿಸುವುದಕ್ಕೆ ಏನೂ ಒತ್ತಾಯ ಇಲ್ಲ. ನೀವು ಭಾಗವಹಿಸುತ್ತೀರೋ ಇಲ್ಲವೋ ಎಂಬುದು ನಿಮ್ಮ ಸ್ವಂತ ಇಚ್ಛೆ.

ನಿಮ್ಮ ಬಳಿ ಮಾತನಾಡುವ ಮೊದಲು ನಾವು ನಿಮ್ಮ ಹೆಂಡತಿಯೊಂದಿಗೆ ಈ ಅಧ್ಯಯನದ ಬಗ್ಗೆ ಮಾತನಾಡಿದ್ದೆವು. ಅವರು ಇದರಲ್ಲಿ ಭಾಗವಹಿಸಲು ಬಯಸಿದ್ದಾರೆ. ಆದರೆ ಇನ್ನೂ ಒಪ್ಪಿಗೆ ತಿಳಿಸಲಿಲ್ಲ.

ಬೇಕಾದರೆ ನೀವು ಈ ಅಧ್ಯಯನದಲ್ಲಿ ಭಾಗವಹಿಸುವ ಬಗ್ಗೆ ಸ್ವಲ್ಪ ಯೋಚಿಸಿದ ನಂತರ ನಿಮ್ಮ ನಿರ್ಧಾರವನ್ನು ನಮಗೆ ತಿಳಿಸಬಹುದು.

ನಿಮಗೆ ಏನಾದರೂ ಪ್ರಶ್ನೆ ಇದ್ದರೆ ನನ್ನ ಹತ್ತಿರ ಕೇಳಲು ಹಿಂಜರಿಯಬೇಡಿ.

**1. ನಾನು ಕುಡಿಯುವುದನ್ನು ಕಡಿಮೆ ಮಾಡಿದ್ದರೆ ನನಗೆ ನಿಜವಾಗಿ ಏನು ಪ್ರಯೋಜನ ಇದೆ?**

ನಾನು ಹೇಳಿದ ಹಾಗೆ, ಈ ಅಧ್ಯಯನದಲ್ಲಿ ನಿಮಗೆ ನಿಮ್ಮ ಕುಡಿತವನ್ನು ಕಡಿಮೆ ಮಾಡುವ ವಿಧಾನಗಳನ್ನು ಹೇಳಿಕೊಡಲಾಗುತ್ತದೆ. ನಿಮ್ಮ ಕುಡಿತವನ್ನು ಕಡಿಮೆ ಮಾಡಿದ್ದರೆ

- ಕುಡಿತದ ವಿಷಯದ ಮೇಲೆ ಮನೆಯಲ್ಲಿ ನಡೆಯುವ ಚರ್ಚೆ ಮತ್ತು ಜಗಳ ಕಡಿಮೆ ಆಗಬಹುದು, ಖರ್ಚು ಕಡಿಮೆ ಆಗಬಹುದು,
- ಉಳಿತಾಯ ಜಾಸ್ತಿ ಆಗಿ ನಿಮ್ಮ ಆರ್ಥಿಕ ಸ್ಥಿತಿ ಒಳ್ಳೆಯದಾಗಬಹುದು,
- ಕುಟುಂಬದವರೊಂದಿಗೆ ಇರುವ ಸಂಬಂಧಗಳೆಲ್ಲ ಒಳ್ಳೆಯದಾಗಬಹುದು,
- ನಿಮ್ಮ ಮಾನಸಿಕ ಮತ್ತು ದೈಹಿಕ ಆರೋಗ್ಯ ಚನ್ನಗಿಡಬಹುದು,
- ಕೆಲಸ ಚನ್ನಾಗಿ ಮಾಡಿ ದುಡಿಯುವುದಕ್ಕೆ ಸುಲಭವಾಗ ಬಹುದು.

ಜೀವನದ ಇವೆಲ್ಲರಲ್ಲಿ ಸುಧಾರಣೆ ಕಂಡು ಬರುವ ಅವಕಾಶ ಇದೆ.

**2. ನನ್ನ ಕುಡಿತದ ಅಳತೆ ನೀವು ಹೇಗೆ ಮಾಡುತ್ತೀರಿ?**

ನೀವು ನಮ್ಮ ಜೊತೆ PHC ಗೆ ಬಂದಾಗ ಅಲ್ಲಿರುವ ಅಧ್ಯಯನದ ಸಿಬ್ಬಂದಿಗಳು ನಿಮಗೆ ನಿಮ್ಮ ಕುಡಿತದ ಅಭ್ಯಾಸದ ಬಗ್ಗೆ ಹಲವಾರು ಪ್ರಶ್ನೆಗಳನ್ನು ಕೇಳುತ್ತಾರೆ. ಈ ರೀತಿಯಲ್ಲಿ, ಅಧ್ಯಯನ ಸಿಬ್ಬಂದಿ ನಿಮ್ಮ ಕುಡಿತವನ್ನು ಅರ್ಥಮಾಡಿಕೊಳ್ಳಲು ನಿಮ್ಮನ್ನು PHC ಗೆ ವರ್ಷದಲ್ಲಿ 4 ಸಲ ಕರೆಯುತ್ತಾರೆ.

ಅದಾದಮೇಲೆ ನಾವು ನಿಮ್ಮ ಮನೆಗೆ ಕುಡಿತವನ್ನು ಅಳತೆ ಮಾಡುವ ಒಂದು ಯಂತ್ರವನ್ನು ತರುತ್ತೇವೆ. ನಿಮಗೆ ಅದನ್ನು ಹೇಗೆ ಬಳಸಬೇಕೆಂದು ಹೇಳಿಕೊಡುತ್ತೇವೆ. ನಾವು ಆ ಯಂತ್ರವನ್ನು ಬಳಸಿ ನಿಮ್ಮ ಕುಡಿತದ ಪ್ರಮಾಣ ಎಷ್ಟು ಇದೆ ಎಂದು ತಿಳಿದುಕೊಳ್ಳುತ್ತೇವೆ. ಈ ಯಂತ್ರ ವರ್ಷದಲ್ಲಿ 3 ಸಲ ನಿಮ್ಮ ಮನೆಗೆ ಬಂದು ಬಳಸಲು ಕೊಡುತ್ತೇವೆ.

**3. PHC ವರಗೆ ಹೋಗುವುದು ಬರುವುದಕ್ಕೆ ಬಹಳ ಖರ್ಚು ಆಗತ್ತೆ...**

PHC ಗಳಿಗೆ ಹೋಗಿ ಬರುವ ಪ್ರಯಾಣಕ್ಕೆ ಪ್ರಾಜೆಕ್ಟ್ ಕಡೆಯಿಂದ ನಿಮಗೆ ಮರುಪಾವತಿ ಕೊಡಲಾಗುತ್ತದೆ.

**4. ಬೇರೆಯವರಿಗೆ ನಾನು ಈ ತರದ ಅಧ್ಯಯನದಲ್ಲಿ ಭಾಗವಹಿಸುತ್ತಿದ್ದೇನೆ ಎಂದು/ನನಗೆ ಕುಡಿಯುವ ಅಭ್ಯಾಸ ಇದೆ ಎಂದು/ನನ್ನ ಸಂಬಂಧಗಳಲ್ಲಿ ಸಮಸ್ಯೆಗಳಿವೆ ಎಂದು ಗೊತ್ತಾದರೆ ಎಂದು ನನಗೆ ಭಯ ಆಗುತ್ತದೆ...**

ನಾನು ಹೇಳಿದ ಹಾಗೆ ನಿಮ್ಮ ಎಲ್ಲ ವಿವರಗಳನ್ನು ನಾವು ಖಡಾಖಂಡಿತವಾಗಿ ಗೌಪ್ಯವಾಗಿ ಇಡುತ್ತೇವೆ. ಎಲ್ಲಾ ಅಧ್ಯಯನದ ಸಿಬ್ಬಂದಿಗಳು ಮತ್ತು ನಾವು ASHA ಕಾರ್ಯಕರ್ತೆಯರು ನಿಮ್ಮ ಯಾವುದೇ ವಿವರಗಳನ್ನು ಯಾರಿಗೂ ಹೇಳುವುದಿಲ್ಲ.

**5. ಇದಕ್ಕೋಸ್ಕರ ನಾವು ಎಷ್ಟು ಸಲ PHC ಗೆ ಬರಬೇಕಾಗತ್ತೆ?**

ಅಧ್ಯಯನದಲ್ಲಿ ನಿಮಗೆ ಸಮಾಲೋಚನೆ ಕೊಡಲಾಗಿದ್ದರೆ ನಿಮಗೆ ಒಟ್ಟಾಗಿ ಒಂದು ವರ್ಷದಲ್ಲಿ 15 ಸಲ PHC ಗೆ ಬರಬೇಕಾಗತ್ತೆ. ಇದರಲ್ಲಿ 10 ಸಲ ಸಭೆಗಳಿಗೆ ಬರುವುದು, ಮತ್ತು ಉಳಿದ 5 ಸಲ ನಿಮ್ಮ ಸಂಬಂಧ ಮತ್ತು ಕುಡಿತವನ್ನು ಅಳತೆ ಮಾಡುವ ಪ್ರಶ್ನೆಗಳನ್ನು ಕೇಳುವುದಕ್ಕೆ ಬರಬೇಕಾಗುತ್ತದೆ.

ನಿಮಗೆ ಸಮಾಲೋಚನೆ ಕೊಡಲಾಗುತ್ತಿಲ್ಲ ಎಂದರೆ ಒಂದು ವರ್ಷದಲ್ಲಿ 6 ಸಲ ಬರಬೇಕಾಗತ್ತೆ. ಇದರಲ್ಲಿ 1 ಸಲ ನಿಮ್ಮ ಕುಡಿತದ ಬಗ್ಗೆ ನಿಮಗೆ ಸ್ವಲ್ಪ ಮಾಹತಿ ಮತ್ತು ಸಹಾಯ ನೀಡಲು ಮತ್ತು ಉಳಿದ 5 ಸಲ ನಿಮ್ಮ ಸಂಬಂಧ ಮತ್ತು ಕುಡಿತವನ್ನು ಅಳತೆ ಮಾಡುವ ಪ್ರಶ್ನೆಗಳನ್ನು ಕೇಳುವುದಕ್ಕೆ ಬರಬೇಕಾಗುತ್ತದೆ. ಇದರ ಬಗ್ಗೆ ಇನ್ನೂ ಮಾಹಿತಿ ಅಧ್ಯಯನದ ಸಿಬ್ಬಂದಿಗಳು ನಿಮಗೆ PHC ನಲ್ಲಿ ನೀಡುತ್ತಾರೆ.

**Appendix C**

**Script for the assessment team member to orient the female participant.**

Hello, my name is ............ I am from St. John's Research Institute, what is your name?

You may have already been given some information about our study by ASHA workers, can you tell me what information they have given you?

Yes! Well now I will give more details about the study. We aim to improve husband-wife relationship and improve home environment through counseling sessions in urban primary health centers in South India.

If you agree to participate in this study, you and your partner will both participate for one year.

During this one year we will interview you for about an hour and a half once every 3 months asking questions about your relationship. This interview will be done separately for you and your partner.

And, in our study, there are 2 groups, in those two groups, if you are selected for the first group, you will get the counseling program, in which you will have to come to meet us for 10 consultations like once a week, your husband will have to come for the first 3 consultations, and for the remaining 7 consultations you and your partner will have to come together.

If you are selected for the second group you will get an educational one hour program. This process takes place after the first interview.

A travel allowance will be given to you and your spouse for each of your visits.

Do you agree to participate in our study for 1 year?

ನಮಸ್ಕಾರ, ನನ್ನ ಹೆಸರು ............ ನಾನು ಸೇಂಟ್ ಜಾನ್ಸ್ ಸಂಶೋಧನಾ ಸಂಸ್ಥೆಯಿಂದ ಬಂದಿದ್ಧೇನೆ, ನಿಮ್ಮ ಹೆಸರೇನು ?

ನಿಮಗೆ ಈಗಾಗಲೇ ಆಶಾ ಕಾರ್ಯಕರ್ತರು ನಮ್ಮ ಅಧ್ಯನದ ಬಗ್ಗೆ ಕೆಲವು ಮಾಹಿತಿಗಳನ್ನು ನೀಡಿರಬಹುದು, ಅವರು ನಿಮಗೆ ಏನು ಮಾಹಿತಿ ಕೊಟ್ಟಿದ್ದಾರೆ ಎಂದು ನನಗೆ ಹೇಳಬಹುದಾ ?

ಹೌದ ! ಸರಿ ನಾನು ಈಗ ಅಧ್ಯಯನದ ಬಗ್ಗೆ ಇನ್ನಷ್ಟು ಮಾಹಿತಿಗಳನ್ನು ತಿಳಿಸುತ್ತೇನೆ. ನಾವು ದಕ್ಷಿಣ ಭಾರತದ ನಗರ ಪ್ರಾಥಮಿಕ ಆರೋಗ್ಯ ಕೇಂದ್ರಗಳಲ್ಲಿ ಸಮಾಲೋಚನೆ ಸಭೆಗಳ ಮೂಲಕ ಗಂಡ-ಹೆಂಡತಿ ಸಂಬಂಧವನ್ನು ಉತ್ತಮಗೊಳಿಸಿ ಮನೆಯ ವಾತಾವರಣವನ್ನು ಸುಖಕರವಾಗಿಸಲು ಮಾಡುವುದಾಗಿರುತ್ತದೆ.

ಈ ಅಧ್ಯಯನದಲ್ಲಿ ಭಾಗವಹಿಸಲು ಒಪ್ಪಿಕೊಂಡರೆ ಒಂದು ವರ್ಷಗಳ ಕಾಲ ನೀವು ಮತ್ತು ನಿಮ್ಮ ಸಂಗಾತಿ ಇಬ್ಬರು ಭಾಗವಹಿಸಬೇಕಾಗುತ್ತದೆ.

ಈ ಒಂದು ವರ್ಷದಲ್ಲಿ ನಾವು ನಿಮ್ಮನ್ನು 5 ಬಾರಿ ನಿಮ್ಮ ಸಂಬಂಧದ ವಿಷಯಗಳ ಕುರಿತು ಪ್ರಶ್ನೆಗಳನ್ನು ಕೇಳುವುದರ ಮೂಲಕ 3 ತಿಂಗಳಿಗೊಮ್ಮೆ ಸುಮಾರು ಒಂದೂವರೆ ಗಂಟೆಗಳ ಕಾಲ ಸಂದರ್ಶಿಸುತ್ತೇವೆ. ಈ ಸಂದರ್ಶನವು ನಿಮಗೂ ಮತ್ತು ನಿಮ್ಮ ಸಂಗಾತಿಗೂ ಪ್ರತ್ಯೇಕವಾಗಿ ಮಾಡಲಾಗುವುದು.

ಹಾಗೂ, ನಮ್ಮ ಅಧ್ಯಯನದಲ್ಲಿ 2 ಗುಂಪುಗಳಿರುತ್ತವೆ ಆ ಎರಡು ಗುಂಪುಗಳಲ್ಲಿ , ಮೊದಲನೇ ಗುಂಪಿಗೆ ನೀವು ಆಯ್ಕೆಯಾದರೆ ಸಮಾಲೋಚನೆ ಕಾರ್ಯಕ್ರಮವನ್ನು ಪಡೆಯುತ್ತಿರಾ, ಅದರಲ್ಲಿ ನೀವು ವಾರಕ್ಕೆ ಒಮ್ಮೆಯಂತೆ 10 ಸಮಾಲೋಚನೆಗಳಿಗೆ ನಮ್ಮನ್ನು ಭೇಟಿಯಾಗಲು ಬರಬೇಕಾಗುತ್ತದೆ, ಮೊದಲ 3 ಸಮಾಲೋಚನೆಯಲ್ಲಿ ನೀವು ಬರಬೇಕಾಗುತ್ತದೆ, ಮತ್ತು ಉಳಿದ 7 ಸಮಾಲೋಚನೆಯಲ್ಲಿ ನೀವು ಮತ್ತೆ ನಿಮ್ಮ ಸಂಗಾತಿ ಇಬ್ಬರು ಬರಬೇಕಾಗುತ್ತದೆ.

ಎರಡನೇ ಗುಂಪಿಗೆ ನೀವು ಆಯ್ಕೆಯಾದರೆ ಒಂದು ಶೈಕ್ಷಣಿಕ ಮಾಹಿತಿಯುಳ್ಳ ಒಂದು ಗಂಟೆಯ ಕಾರ್ಯಕ್ರಮವನ್ನು ಪಡೆಯುತ್ತೀರಿ. ಈ ಪ್ರಕ್ರಿಯೆ ಮೊದಲ ಸಂದರ್ಶನದ ನಂತರ ನಡೆಯುತ್ತದೆ.

ನಿಮ್ಮ ಪ್ರತಿ ಭೇಟಿಗೆ ನಿಮಗು ಮತ್ತು ನಿಮ್ಮ ಸಂಗಾತಿಗೂ ಸೇರಿ ಪ್ರಯಾಣ ಭತ್ಯೆಯನ್ನು ನೀಡಲಾಗುವುದು.

ನಿಮಗೆ 1 ವರ್ಷಗಳ ಕಾಲ ನಮ್ಮ ಅಧ್ಯಯನದಲ್ಲಿ ಭಾಗವಹಿಸಲು ಒಪ್ಪಿಗೆ ಇದೆಯೇ ?

**Appendix D**

**Script for assessment team member to orient the couple before they are split up for screening**

Greetings! My name is _____. How are you doing today?

I am here on behalf of BBMP and St. John’s Hospital to tell you about our new research project called Harmony.

We all face difficult and stressful situations in life, especially in our relationships. Marriages can be hard to navigate, and relationship issues can often contribute to a lot of stress. Our program here is designed to help couples improve their relationship, by working on trust, healthy communication, and solving problems, to deal with these inevitable challenges that we all experience in a better way.

Could I tell you both a little more about the potential benefits of participating in this program?

You may get the opportunity to learn how to communicate better with each other, work on solving problems together, and establish trust in your relationship. This will be done through counselling sessions provided by our trained staff. In these sessions, you may also learn how to relax your mind and body to cope with stressful situations and manage uncomfortable and strong emotions such as anger. A part of these counselling sessions is focused on understanding one’s drinking patterns, since that can also contribute to stress and challenges in one’s marriage. These sessions can therefore help you understand and thereby cut down on your drinking and take good care of your health.

The study requires that both of you join as a couple, and not individually, since we will be working with both of you to help you improve your relationship with each other.

In order to better understand if you can participate in the program and to provide you more information, we would like to ask you a few questions separately. Before we do that, I would like to assure that we will maintain complete confidentiality of your identifying details from our side, so that nobody outside the study knows about your participation, your relationship, or any other matters discussed with us.

Do you have any questions about what we have talked about this far? Please feel free to ask me.

Would you be willing to speak our staff here to see if our what we are offering in our program meets your needs?

[request husband and wife to be seated in front of the male and female interviewer respectively for screening procedures].

ನಮಸ್ತೆ! ನನ್ನ ಹೆಸರು _____. ಹೇಗಿದ್ದೀರಾ?

ನಾನು ಬಿ.ಬಿ. ಎಂ. ಪಿ ಮತ್ತು ಸೇಂಟ್ ಜಾನ್ಸ್ ಆಸ್ಪತ್ರೆಯ ಪರವಾಗಿ ಹಾರ್ಮನಿ ಎಂಬ ನಮ್ಮ ಹೊಸ ಸಂಶೋಧನಾ ಯೋಜನೆಯ ಬಗ್ಗೆ ಹೇಳಲು ಬಂದಿದ್ದೇನೆ.

ನಾವೆಲ್ಲರೂ ಜೀವನದಲ್ಲಿ ಕಷ್ಟಕರ ಮತ್ತು ಒತ್ತಡದ ಸಂದರ್ಭಗಳನ್ನು ಎದುರಿಸುತ್ತೇವೆ, ವಿಶೇಷವಾಗಿ ನಮ್ಮ ಸಂಬಂಧಗಳಲ್ಲಿ. ಮದುವೆಯನ್ನು ನಿಭಾಯಿಸಲು ಕಷ್ಟವಾಗಬಹುದು ಮತ್ತು ಸಂಬಂಧದ ಸಮಸ್ಯೆಗಳು ಹೆಚ್ಚಾಗಿ ಒತ್ತಡಕ್ಕೆ ಕಾರಣವಾಗಬಹುದು. ನಾವೆಲ್ಲರೂ ಉತ್ತಮ ರೀತಿಯಲ್ಲಿ ಅನುಭವಿಸುವ ಈ ಅನಿವಾರ್ಯ ಸವಾಲುಗಳನ್ನು ಎದುರಿಸಲು, ನಂಬಿಕೆ, ಆರೋಗ್ಯಕರ ಸಂವಹನ ಮತ್ತು ಸಮಸ್ಯೆಗಳನ್ನು ಪರಿಹರಿಸುವ ಮೂಲಕ ದಂಪತಿಗಳು ತಮ್ಮ ಸಂಬಂಧವನ್ನು ಸುಧಾರಿಸಲು ಸಹಾಯ ಮಾಡಲು ಇಲ್ಲಿ ನಮ್ಮ ಕಾರ್ಯಕ್ರಮವನ್ನು ವಿನ್ಯಾಸಗೊಳಿಸಲಾಗಿದೆ.

ಈ ಕಾರ್ಯಕ್ರಮದಲ್ಲಿ ಭಾಗವಹಿಸುವ ಸಂಭಾವ್ಯ ಪ್ರಯೋಜನಗಳ ಬಗ್ಗೆ ನಾನು ನಿಮ್ಮಿಬ್ಬರಿಗೂ ಸ್ವಲ್ಪ ಹೆಚ್ಚು ಹೇಳಬಹುದೇ?

ಪರಸ್ಪರ ಉತ್ತಮವಾಗಿ ಸಂವಹನ ಮಾಡುವುದು, ಸಮಸ್ಯೆಗಳನ್ನು ಪರಿಹರಿಸುವಲ್ಲಿ ಒಟ್ಟಿಗೆ ಕೆಲಸ ಮಾಡುವುದು ಮತ್ತು ನಿಮ್ಮ ಸಂಬಂಧದಲ್ಲಿ ನಂಬಿಕೆಯನ್ನು ಸ್ಥಾಪಿಸುವುದು ಹೇಗೆ ಎಂಬುದನ್ನು ಕಲಿಯಲು ನೀವು ಅವಕಾಶವನ್ನು ಪಡೆಯಬಹುದು. ನಮ್ಮ ತರಬೇತಿ ಪಡೆದ ಸಿಬ್ಬಂದಿ ಒದಗಿಸುವ ಕೌನ್ಸೆಲಿಂಗ್ ಸೆಷನ್‌ಗಳ ಮೂಲಕ ಇದನ್ನು ಮಾಡಲಾಗುತ್ತದೆ. ಈ ಅವಧಿಗಳಲ್ಲಿ, ಒತ್ತಡದ ಸಂದರ್ಭಗಳನ್ನು ನಿಭಾಯಿಸಲು ಮತ್ತು ಕೋಪದಂತಹ ಅಹಿತಕರ ಮತ್ತು ಬಲವಾದ ಭಾವನೆಗಳನ್ನು ನಿರ್ವಹಿಸಲು ನಿಮ್ಮ ಮನಸ್ಸು ಮತ್ತು ದೇಹವನ್ನು ಹೇಗೆ ವಿಶ್ರಾಂತಿ ಮಾಡುವುದು ಎಂಬುದನ್ನು ಸಹ ನೀವು ಕಲಿಯಬಹುದು. ಈ ಕೌನ್ಸೆಲಿಂಗ್ ಸೆಷನ್‌ಗಳ ಒಂದು ಭಾಗವು ಒಬ್ಬರ ಕುಡಿಯುವ ಮಾದರಿಗಳನ್ನು ಅರ್ಥಮಾಡಿಕೊಳ್ಳುವುದರ ಮೇಲೆ ಕೇಂದ್ರೀಕೃತವಾಗಿದೆ, ಏಕೆಂದರೆ ಅದು ಒಬ್ಬರ ಮದುವೆಯಲ್ಲಿ ಒತ್ತಡ ಮತ್ತು ಸವಾಲುಗಳಿಗೆ ಸಹ ಕೊಡುಗೆ ನೀಡುತ್ತದೆ. ಆದ್ದರಿಂದ ಈ ಸೆಷನ್‌ಗಳು ನಿಮಗೆ ಅರ್ಥಮಾಡಿಕೊಳ್ಳಲು ಸಹಾಯ ಮಾಡುತ್ತದೆ ಮತ್ತು ಆ ಮೂಲಕ ನಿಮ್ಮ ಕುಡಿತವನ್ನು ಕಡಿಮೆ ಮಾಡುತ್ತದೆ ಮತ್ತು ನಿಮ್ಮ ಆರೋಗ್ಯವನ್ನು ಚೆನ್ನಾಗಿ ನೋಡಿಕೊಳ್ಳುತ್ತದೆ.

ಅಧ್ಯಯನಕ್ಕೆ ನೀವಿಬ್ಬರೂ ಜೋಡಿಯಾಗಿ ಸೇರಬೇಕು, ಮತ್ತು ಪ್ರತ್ಯೇಕವಾಗಿ ಅಲ್ಲ, ಏಕೆಂದರೆ ನಾವು ನಿಮ್ಮಿಬ್ಬರೊಂದಿಗೆ ಪರಸ್ಪರ ಸಂಬಂಧವನ್ನು ಸುಧಾರಿಸಲು ಸಹಾಯ ಮಾಡುತ್ತೇವೆ.

ನೀವು ಕಾರ್ಯಕ್ರಮದಲ್ಲಿ ಭಾಗವಹಿಸಬಹುದೇ ಎಂಬುದನ್ನು ಚೆನ್ನಾಗಿ ಅರ್ಥಮಾಡಿಕೊಳ್ಳಲು ಮತ್ತು ನಿಮಗೆ ಹೆಚ್ಚಿನ ಮಾಹಿತಿಯನ್ನು ಒದಗಿಸಲು, ನಾವು ನಿಮಗೆ ಕೆಲವು ಪ್ರಶ್ನೆಗಳನ್ನು ಪ್ರತ್ಯೇಕವಾಗಿ ಕೇಳಲು ಬಯಸುತ್ತೇವೆ. ನಾವು ಅದನ್ನು ಮಾಡುವ ಮೊದಲು, ನಾವು ನಮ್ಮ ಕಡೆಯಿಂದ ನಿಮ್ಮ ಗುರುತಿಸುವ ವಿವರಗಳ ಸಂಪೂರ್ಣ ಗೌಪ್ಯತೆಯನ್ನು ಕಾಪಾಡಿಕೊಳ್ಳುತ್ತೇವೆ ಎಂದು ನಾನು ಭರವಸೆ ನೀಡಲು ಬಯಸುತ್ತೇನೆ, ಆದ್ದರಿಂದ ಅಧ್ಯಯನದ ಹೊರಗಿನ ಯಾರಿಗೂ ನಿಮ್ಮ ಭಾಗವಹಿಸುವಿಕೆ, ನಿಮ್ಮ ಸಂಬಂಧ ಅಥವಾ ನಮ್ಮೊಂದಿಗೆ ಚರ್ಚಿಸಿದ ಯಾವುದೇ ವಿಷಯಗಳ ಬಗ್ಗೆ ತಿಳಿದಿರುವುದಿಲ್ಲ.

ನಾವು ಇಲ್ಲಿಯವರೆಗೆ ಏನು ಮಾತನಾಡಿದ್ದೇವೆ ಎಂಬುದರ ಕುರಿತು ನೀವು ಯಾವುದೇ ಪ್ರಶ್ನೆಗಳನ್ನು ಹೊಂದಿದ್ದೀರಾ? ದಯವಿಟ್ಟು ನನ್ನನ್ನು ಕೇಳಲು ಹಿಂಜರಿಯಬೇಡಿ.

ನಮ್ಮ ಪ್ರೋಗ್ರಾಂನಲ್ಲಿ ನಾವು ನೀಡುತ್ತಿರುವುದು ನಿಮ್ಮ ಅಗತ್ಯಗಳನ್ನು ಪೂರೈಸುತ್ತದೆಯೇ ಎಂದು ನೋಡಲು ನಮ್ಮ ಸಿಬ್ಬಂದಿಯನ್ನು ಇಲ್ಲಿ ಮಾತನಾಡಲು ನೀವು ಸಿದ್ಧರಿದ್ದೀರಾ?

[ಸ್ಕ್ರೀನಿಂಗ್ ಕಾರ್ಯವಿಧಾನಗಳಿಗಾಗಿ ಪತಿ ಮತ್ತು ಹೆಂಡತಿಯನ್ನು ಕ್ರಮವಾಗಿ ಪುರುಷ ಮತ್ತು ಮಹಿಳಾ ಸಂದರ್ಶಕರ ಮುಂದೆ ಕುಳಿತುಕೊಳ್ಳಲು ವಿನಂತಿಸಿ]

**Appendix E**

**Project Harmony – Visit Schedule**

**
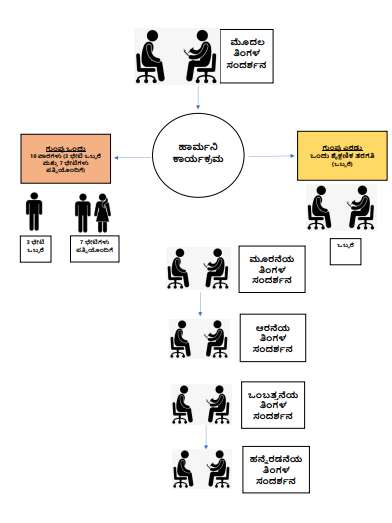
**

**Appendix F**

**Screening Forms**

**Husband**

| **Screening ID:_____________ Date** ____ / _______/_____  dd mm yy  **Age:** __________ **Int I** ____________ **PHC**_____________ | Referral Source | | |
| --- | --- | --- | --- |
|  | ☐ ASHA ☐ Self  ☐ Friend ☐ Any other | | |
| **1. Eligibility criteria**  **(Need to be Yes to 1a – 1h, and No to 1i to be eligible)** | | **Yes** | **No** |
| 1a. Married and above age of 18 years | | ☐  Eligible | ☐ |
| 1b. Currently living with Wife | | ☐  Eligible | ☐ |
| 1c. Understands Kannada fluently | | ☐  Eligible | ☐ |
| 1d. Willing to participate in all the program components.  (5 assessment session over one year and 10 weeks intervention session, if randomized) | | ☐  Eligible | ☐ |
| 1e. AUDIT-C Score > 4 (AUDIT-C Score___________) | | ☐  Eligible | ☐ |
| 1f. Short Blessed Cognitive Score <7 (SBT Score ____________) | | ☐  Eligible | ☐ |
| 1g. Severe Alcohol Dependence Questionnaire (score <31) (SADQ Score_______) | | ☐  Eligible | ☐ |
| 1h. CIWA – AR < 8 (CIWA-AR Score__________) | | ☐  Eligible | ☐ |
| 1i. Presence of any significant medical problems that makes it impossible to participate in study | | ☐ | ☐  Eligible |
| 2. **Enrollment**  Consented to participate and Enrolled. Participant Dummy ID number is ______________ | | | |
| 3. **Reasons for non-enrollment**  ☐ Does not meet the eligibility criteria mentioned above  ☐ Not willing to enroll (Please specify reason if any)___________________________________________    ☐ Any other reasons______________________________________________________________________ | | | |

**Screening form Wife**

| **Screening ID:_____________ Date** ____ / _______/_____  dd mm yy  **Age:** __________ **Int I** ____________ **PHC**_____________ | Referral Source | | |
| --- | --- | --- | --- |
|  | ☐ ASHA ☐ Self  ☐ Friend ☐ Any other | | |
| **1. Eligibility criteria**  **(Need to be Yes to 1a - 1g, 1i and No to 1h and 1j to be eligible)** | | **Yes** | **No** |
| 1a. Married and above age of 18 years | | ☐  Eligible | ☐ |
| 1b. Currently living with Husband | | ☐  Eligible | ☐ |
| 1c. Understands Kannada fluently | | ☐  Eligible | ☐ |
| 1d. Willing to participate in all the program components.  (5 assessment session over one year and 10 weeks intervention session, if randomized) | | ☐  Eligible | ☐ |
| 1e. Husband drinks Alcohol | | ☐  Eligible | ☐ |
| 1f. Short Blessed Cognitive Score <7 (SBT Score ____________) | | ☐  Eligible | ☐ |
| 1g. IVAWS – Presence of IPV in the past one year | | ☐  Eligible | ☐ |
| 1h. If Yes to 1g (above), hospitalization due to IPV in past 1 year | | ☐ | ☐  Eligible |
| 1i. AUDIT-C Score < 4 (AUDIT-C Score___________) | | ☐  Eligible | ☐ |
| 1j. Presence of any significant medical problems that makes it impossible to participate in study | | ☐ | ☐  Eligible |
| 2. **Enrollment**  Consented to participate and Enrolled. Participant Dummy ID number is ______________  3. **Schedule to meet the husband**  Chose to bring the husband over through – ☐ Self ☐ ASHA Worker ☐ Assessment Team Member | | | |
| 4. **Reasons for non-enrollment**  ☐ Does not meet the eligibility criteria mentioned above  ☐ Not willing to enroll (Please specify reason if any)___________________________________________    ☐ Any other reasons______________________________________________________________________ | | | |

**Appendix G**

**Script for women who have AUD at screening and want to seek help.**

Hello, ___________.

As you know, we have asked you several questions about your drinking habits. On the basis of our assessment, we calculated a score. This score indicates that your current alcohol consumption is within a range that is considered “problem drinking”, which is also referred to as an “alcohol use disorder”. You have indicated that you would like to seek help for this problem. For that reason, we have written referral letters to the NIMHANS Centre for Addiction Medicine as well as to the nearest general hospital. These letters are signed by the project in-charges who are psychiatrists. This letter also includes a brief description of the “problem drinking” that we mentioned earlier, along with the score that is based on the questions we asked you. This will help the doctor who sees you at NIMHANS or the general hospital better understand your current alcohol consumption patterns. If you wish to seek help, you may go to the NIMHANS OPD on any day other than Sunday, between 8 AM and 12 noon. In case of emergency, you may visit the NIMHANS Emergency Clinic any time on any day, as it is open 24/7.

Do you have any questions? I would be glad to answer them for you to the best of my ability. If I don’t know the answer to a question, I will find it out and get back to you.

ಹಲೋ, _____.

ನಿಮ್ಮ ಕುಡಿಯುವ ಅಭ್ಯಾಸದ ಕುರಿತು ನಾವು ನಿಮಗೆ ಹಲವಾರು ಪ್ರಶ್ನೆಗಳನ್ನು ಕೇಳಿದ್ದೇವೆ. ನಿಮ್ಮ ಕುಡಿಯುವ ನಡವಳಿಕೆಯ ನಮ್ಮ ಮೌಲ್ಯಮಾಪನದ ಆಧಾರದ ಮೇಲೆ, ನಿಮಗೆ ಅಂಕ ನೀಡಿದ್ದೇವೆ. ಈ ಅಂಕವು ನಿಮ್ಮ ಪ್ರಸ್ತುತ ಆಲ್ಕೋಹಾಲ್ ಸೇವನೆಯು "ಸಮಸ್ಯೆ ಕುಡಿತ" ಎಂದು ಪರಿಗಣಿಸಲ್ಪಡುವ ವ್ಯಾಪ್ತಿಯಲ್ಲಿದೆ ಎಂದು ಸೂಚಿಸುತ್ತದೆ ಮತ್ತು ನಾವು ಇದನ್ನು ಸಾಮಾನ್ಯವಾಗಿ "ಆಲ್ಕೋಹಾಲ್ ಬಳಕೆಯ ಅಸ್ವಸ್ಥತೆ" ಎಂದು ಕರೆಯುತ್ತೇವೆ. ಅದಕ್ಕಾಗಿ ನೀವು ಸಹಾಯವನ್ನು ಪಡೆಯಲು ಬಯಸುತ್ತೀರಿ ಎಂದು ಸೂಚಿಸಿದ್ದೀರಿ. ಆ ಕಾರಣಕ್ಕಾಗಿ, ನಾವು ಇಲ್ಲಿ ನಿಮ್ಹಾನ್ಸ್ ಸೆಂಟರ್ ಫಾರ್ ಅಡಿಕ್ಷನ್ ಮೆಡಿಸಿನ್ ಮತ್ತು ಹತ್ತಿರದ ಜನರಲ್ ಆಸ್ಪತ್ರೆಗೆ ಶಿಪಾರಸ್ಸು ಪತ್ರಗಳನ್ನು ಹೊಂದಿದ್ದೇವೆ. ಈ ಪತ್ರಗಳಿಗೆ ಸ್ವತಃ ಮನೋವೈದ್ಯರಾದ ಪ್ರಾಜೆಕ್ಟ್ ಇನ್‌ಚಾರ್ಜ್‌ಗಳು ಸಹಿ ಮಾಡಿದ್ದಾರೆ. ಈ ಪತ್ರವು ನಾವು ನಿಮಗೆ ಕೇಳಿದ ಪ್ರಶ್ನೆಗಳ ಅಂಕದೊಂದಿಗೆ ನಾವು ಮೊದಲೇ ಪ್ರಸ್ತಾಪಿಸಿದ “ಕುಡಿಯುವ ಸಮಸ್ಯೆ” ಯ ಸಂಕ್ಷಿಪ್ತ ವಿವರಣೆಯನ್ನು ಸಹ ಹೊಂದಿದೆ, ಇದರಿಂದ ನಿಮ್ಹಾನ್ಸ್ ಅಥವಾ ಜನರಲ್ ಆಸ್ಪತ್ರೆಯಲ್ಲಿ ನಿಮ್ಮನ್ನು ನೋಡುವ ವೈದ್ಯರು ನಿಮ್ಮ ಪ್ರಸ್ತುತ ಆಲ್ಕೊಹಾಲ್ ಸೇವನೆಯ ಬಗ್ಗೆ ಉತ್ತಮ ತಿಳುವಳಿಕೆಯನ್ನು ಹೊಂದಿರುತ್ತಾರೆ. ಮಾದರಿಗಳು. ನೀವು ಸಹಾಯವನ್ನು ಪಡೆಯಲು ಬಯಸಿದರೆ, ನೀವು ಭಾನುವಾರ ಹೊರತುಪಡಿಸಿ ಯಾವುದೇ ದಿನದಲ್ಲಿ 8 AM ಮತ್ತು 12 ಮಧ್ಯಾಹ್ನದ ನಡುವೆ ನಿಮ್ಹಾನ್ಸ್ OPD ಗೆ ಹೋಗಬಹುದು.

ನೀವು ಯಾವುದೇ ಪ್ರಶ್ನೆಗಳನ್ನು ಹೊಂದಿದ್ದೀರಾ? ನನ್ನ ತಿಳುವಳಿಕೆಯ ಮಟ್ಟಿಗೆ ನಿಮಗಾಗಿ ಅವರಿಗೆ ಉತ್ತರಿಸಲು ನಾನು ಸಂತೋಷಪಡುತ್ತೇನೆ.

**Appendix H**

**Script for women who report IPV at screening and are not eligible.**

*Hello, ______. You have shared with us the problems you have experienced at home and in your relationship with your husband. If you would like, you may talk to me about these issues too. I’m here to listen…*

*[Allow for the woman to speak if she wishes to ventilate. If not, do not press for her to share.]*

*I understand that it is not easy to talk about these matters. Thank you for choosing to share these matters with us. May I know if you are concerned/afraid that something like this may happen again?*

*[Encourage the woman to share with you her fears or concerns revolving around the likelihood of recurrence of violence at home].*

*Your safety is a priority to us. In situations like the ones you have described, it is important that you feel safe and are safe. I am going to give you a few contact numbers of people who you can reach out to for help in times when you feel unsafe in your own home or in your relationship. If you feel like your husband may hurt you, or if there is an incident of or increase in violence (screaming, throwing, pushing, hitting, forcing, controlling, etc.), we would like you to contact these numbers or let your ASHA know that you need help. Let me tell you who to call, and in what ways we may support you.*

**NIMHANS Legal Aid Clinic**

1. Advocate Hamsa Javaly: 9845214596
2. Advocate Veerabhadraiah: 9741497005

Address: NIMHANS Legal Aid Clinic, NIMHANS OPD, Hosur Road, Bengaluru, Karnataka 560029

Address (Head Office): Karnataka State Legal Services Authority

Nyaay Degula,

Siddaiah Road, Bengaluru, Karnataka - 560027

*You may call these two lawyers. They work at NIMHANS. They can provide you with legal support in case you wish to be protected. They can also get you medical support, as well as arrange for counseling if you wish to speak to someone.*

*If you call them, they will talk to you about your situation, and may ask you to go to the NIMHANS Legal Cell or their head office. If you wish, we can make an appointment for you and accompany you, or an ASHA can go with you to the NIMHANS Legal Cell.*

**Sakhi One Stop Centre**

Ms. Amitha: 080 26538977; 9164069641

Address: Sakhi-One Stop Centre (Nirbhaya), 2^nd^ Floor, BMTC Bus Pass Counter Building, Airport Counter, Majestic, Bengaluru, Karnataka – 560001

*If you call this number, Ms. Amitha can help provide you with shelter in case you wish to leave your home. They can also provide medical support, legal support and counselling. If you would like, we can make an appointment for you and accompany you, or an ASHA can go with you to the Sakhi Centre.*

*Do you have any questions about these contacts, or how to go about reaching out to them? Please feel free to ask me.*

*I would like to know if you would be comfortable taking this sheet of paper with phone numbers written on them with you. If not, we have other ways in which you can reach out to them for support. I can help store these numbers on your phone in a locked folder that others cannot access and show you how you can access them. I can also ask for an ASHA to keep this sheet of paper with her, so that you may contact her, and she can give you these numbers [ask for the name of a specific ASHA and note it down]. [Note down the option opted for by the participant].*

*Please don’t hesitate to reach out to them in case you feel unsafe or if you feel there is a chance that you may get hurt. It is important that you get the help that you need.*

ಹಾರ್ಮನಿ ಮಹಿಳಾ ಸಂಪನ್ಮೂಲಗಳ ಪಟ್ಟಿ - IPV ಅನ್ನು ವರದಿ ಮಾಡುವ ಮತ್ತು ಅನರ್ಹರಾಗಿರುವ ಮಹಿಳೆಯರು

ಹಲೋ, __. ನೀವು ಮನೆಯಲ್ಲಿ ಮತ್ತು ನಿಮ್ಮ ಗಂಡನೊಂದಿಗಿನ ಸಂಬಂಧದಲ್ಲಿ ನೀವು ಅನುಭವಿಸಿದ ಸಮಸ್ಯೆಗಳನ್ನು ನಮ್ಮೊಂದಿಗೆ ಹಂಚಿಕೊಂಡಿದ್ದೀರಿ. ನೀವು ಬಯಸಿದರೆ, ನೀವು ಈ ಸಮಸ್ಯೆಗಳ ಬಗ್ಗೆ ನನ್ನೊಂದಿಗೆ ಮಾತನಾಡಬಹುದು. ನಾನು ಕೇಳಲು ಇಲ್ಲಿದ್ದೇನೆ ...

[Allow for the woman to speak if she wishes to ventilate. If not, do not press for her to share.]

ಈ ವಿಷಯಗಳ ಬಗ್ಗೆ ಮಾತನಾಡುವುದು ಸುಲಭವಲ್ಲ ಎನ್ನುವುದು ನನಗೆ ಅರ್ಥವಾಗುತ್ತದೆ. ಈ ವಿಷಯಗಳನ್ನು ನಮ್ಮೊಂದಿಗೆ ಹಂಚಿಕೊಳ್ಳಲು ಆಯ್ಕೆ ಮಾಡಿಕೊಂಡಿದ್ದಕ್ಕೆ ಧನ್ಯವಾದಗಳು. ಮುಂದೆ ಇಂತಹ ಘಟನೆ ಮರುಕಳಿಸಬಹುದೆಂದು ನೀವು ಆತಂಕಗೊಂಡಿದ್ದೀರಾ ಅಥವಾ ಭಯಗೊಂಡಿದ್ದೀರಾ?

*[Encourage the woman to share with you her fears or concerns revolving around the likelihood of recurrence of violence at home].*

ನಿಮ್ಮ ಸುರಕ್ಷತೆಯು ನಮಗೆ ಆದ್ಯತೆಯಾಗಿದೆ. ನೀವು ವಿವರಿಸಿರುವಂತಹ ಸಂದರ್ಭಗಳಲ್ಲಿ, ನೀವು ಸುರಕ್ಷಿತವಾಗಿರುವುದು ಮತ್ತು ನಿಜವಾಗಿ ಸುರಕ್ಷಿತವಾಗಿರುವುದು ಮುಖ್ಯವಾಗಿದೆ. ನಿಮ್ಮ ಸ್ವಂತ ಮನೆಯಲ್ಲಿ ಅಥವಾ ನಿಮ್ಮ ಸಂಬಂಧದಲ್ಲಿ ನೀವು ಅಸುರಕ್ಷಿತ ಎಂದು ಭಾವಿಸುವ ಸಮಯದಲ್ಲಿ ಸಹಾಯಕ್ಕಾಗಿ ನೀವು ತಲುಪಬಹುದಾದ ಜನರ ಕೆಲವು ಸಂಪರ್ಕ ಸಂಖ್ಯೆಗಳನ್ನು ನಾನು ನಿಮಗೆ ನೀಡಲಿದ್ದೇನೆ. ನಿಮ್ಮ ಪತಿ ನಿಮಗೆ ನೋವುಂಟುಮಾಡಬಹುದು ಎಂದು ನೀವು ಭಾವಿಸಿದರೆ ಅಥವಾ ಹಿಂಸಾಚಾರದ ಘಟನೆ ಅಥವಾ ಹೆಚ್ಚಾದರೆ (ಕಿರುಚುವುದು, ಎಸೆಯುವುದು, ತಳ್ಳುವುದು, ಹೊಡೆಯುವುದು, ಒತ್ತಾಯಿಸುವುದು, ನಿಯಂತ್ರಿಸುವುದು, ಇತ್ಯಾದಿ), ಈ ಸಂಖ್ಯೆಗಳನ್ನು ಸಂಪರ್ಕಿಸಲು ನಾವು ಬಯಸುತ್ತೇವೆ. ಯಾರಿಗೆ ಕರೆ ಮಾಡಬೇಕು ಮತ್ತು ಯಾವ ರೀತಿಯಲ್ಲಿ ನಾವು ನಿಮ್ಮನ್ನು ಬೆಂಬಲಿಸಬಹುದು ಎಂದು ನಾನು ನಿಮಗೆ ಹೇಳುತ್ತೇನೆ.

ಸಂಖ್ಯೆಗಳು. ಯಾರಿಗೆ ಕರೆ ಮಾಡಬೇಕು ಮತ್ತು ಯಾವ ರೀತಿಯಲ್ಲಿ ನಾವು ನಿಮ್ಮನ್ನು ಬೆಂಬಲಿಸಬಹುದು ಎಂದು ನಾನು ನಿಮಗೆ ಹೇಳುತ್ತೇನೆ.

ನಿಮ್ಹಾನ್ಸ್ ಕಾನೂನು ನೆರವು ಕ್ಲಿನಿಕ್

ನ್ಯಾಯವಾದಿ ಹಂಸ ಜವಳಿ: 9845214596

ವಕೀಲ ವೀರಭದ್ರಯ್ಯ: 9741497005

ವಿಳಾಸ: ನಿಮ್ಹಾನ್ಸ್ ಲೀಗಲ್ ಏಡ್ ಕ್ಲಿನಿಕ್, ನಿಮ್ಹಾನ್ಸ್ ಒಪಿಡಿ, ಹೊಸೂರ್ ರೋಡ್, ಬೆಂಗಳೂರು, ಕರ್ನಾಟಕ 560029

ವಿಳಾಸ (ಪ್ರಧಾನ ಕಛೇರಿ): ಕರ್ನಾಟಕ ರಾಜ್ಯ ಕಾನೂನು ಸೇವೆಗಳ ಪ್ರಾಧಿಕಾರ

ನ್ಯಾಯ ದೇಗುಲಾ,

ಸಿದ್ದಯ್ಯ ರಸ್ತೆ, ಬೆಂಗಳೂರು, ಕರ್ನಾಟಕ – 560027

ನೀವು ಈ ಇಬ್ಬರು ವಕೀಲರನ್ನು ಸಂಪರ್ಕಿಸಬಹುದು. ಇವರು ನಿಮ್ಹಾನ್ಸ್‌ನಲ್ಲಿ ಕೆಲಸ ಮಾಡುತ್ತಾರೆ. ನೀವು ರಕ್ಷಣೆಯನ್ನು ಬಯಸಿದರೆ ಅವರು ನಿಮಗೆ ಕಾನೂನು ಬೆಂಬಲವನ್ನು ಒದಗಿಸಬಹುದು. ಅವರು ನಿಮಗೆ ವೈದ್ಯಕೀಯ ಬೆಂಬಲವನ್ನು ಪಡೆಯಬಹುದು, ಹಾಗೆಯೇ ನೀವು ಯಾರೊಂದಿಗಾದರೂ ಮಾತನಾಡಲು ಬಯಸಿದರೆ ಸಮಾಲೋಚನೆಗಾಗಿ ವ್ಯವಸ್ಥೆ ಮಾಡಬಹುದು.

ನೀವು ಅವರಿಗೆ ಕರೆ ಮಾಡಿದರೆ, ಅವರು ನಿಮ್ಮ ಪರಿಸ್ಥಿತಿಯ ಬಗ್ಗೆ ನಿಮ್ಮೊಂದಿಗೆ ಮಾತನಾಡುತ್ತಾರೆ ಮತ್ತು ನಿಮ್ಹಾನ್ಸ್ ಲೀಗಲ್ ಸೆಲ್ ಅಥವಾ ಅವರ ಮುಖ್ಯ ಕಚೇರಿಗೆ ಹೋಗಲು ನಿಮ್ಮನ್ನು ಕೇಳಬಹುದು. ನೀವು ಅವರನ್ನು ಸಂಪರ್ಕಿಸುವ ಮೊದಲು ನೀವು ನಮ್ಮನ್ನು (ನಮ್ಮ ಅಧ್ಯಯನದ ಫೋನ್ ಸಂಖ್ಯೆ) ಸಂಪರ್ಕಿಸಬಹುದು, ಇದರಿಂದ ನಾವು ನಿಮ್ಮ ಬಗ್ಗೆ ಅವರಿಗೆ ತಿಳಿಸಬಹುದು. ನೀವು ಬಯಸಿದರೆ, ನಾವು ನಿಮಗಾಗಿ ಅಪಾಯಿಂಟ್‌ಮೆಂಟ್ ಮಾಡಬಹುದು ಮತ್ತು ನಿಮ್ಹಾನ್ಸ್ ಲೀಗಲ್ ಸೆಲ್‌ಗೆ ನಿಮ್ಮೊಂದಿಗೆ ಹೋಗಬಹುದು.

ವಿಳಾಸ: ಸಖಿ-ಒನ್ ಸ್ಟಾಪ್ ಸೆಂಟರ್ (ನಿರ್ಭಯ), 2 ನೇ ಮಹಡಿ, BMTC ಬಸ್ ಪಾಸ್ ಕೌಂಟರ್ ಬಿಲ್ಡಿಂಗ್, ಏರ್‌ಪೋರ್ಟ್ ಕೌಂಟರ್, ಮೆಜೆಸ್ಟಿಕ್, ಬೆಂಗಳೂರು, ಕರ್ನಾಟಕ - 560001

ನೀವು ಈ ಸಂಖ್ಯೆಗೆ ಕರೆ ಮಾಡಿದರೆ, ನಿಮ್ಮ ಮನೆಯಿಂದ ಹೊರಹೋಗಲು ನೀವು ಬಯಸಿದಲ್ಲಿ ಶ್ರೀಮತಿ ಅಮಿತಾ ನಿಮಗೆ ಆಶ್ರಯವನ್ನು ಒದಗಿಸಲು ಸಹಾಯ ಮಾಡಬಹುದು. ಅವರು ವೈದ್ಯಕೀಯ ಬೆಂಬಲ, ಕಾನೂನು ಬೆಂಬಲ ಮತ್ತು ಸಮಾಲೋಚನೆಯನ್ನು ಸಹ ಒದಗಿಸಬಹುದು. ನೀವು ಅವರನ್ನು ಸಂಪರ್ಕಿಸುವ ಮೊದಲು ನೀವು ನಮ್ಮನ್ನು (ನಮ್ಮ ಅಧ್ಯಯನದ ಫೋನ್ ಸಂಖ್ಯೆ) ಸಂಪರ್ಕಿಸಬಹುದು, ಇದರಿಂದ ನಾವು ನಿಮ್ಮ ಬಗ್ಗೆ ಅವರಿಗೆ ತಿಳಿಸಬಹುದು. ನೀವು ಬಯಸಿದರೆ, ನಾವು ನಿಮಗಾಗಿ ಅಪಾಯಿಂಟ್‌ಮೆಂಟ್ ಮಾಡಬಹುದು ಮತ್ತು ನಿಮ್ಮೊಂದಿಗೆ ಸಖಿ ಕೇಂದ್ರಕ್ಕೆ ಹೋಗಬಹುದು.

ಈ ಸಂಪರ್ಕಗಳ ಕುರಿತು ನೀವು ಯಾವುದೇ ಪ್ರಶ್ನೆಗಳನ್ನು ಹೊಂದಿದ್ದೀರಾ ಅಥವಾ ಅವರನ್ನು ಹೇಗೆ ಸಂಪರ್ಕಿಸುವುದು? ದಯವಿಟ್ಟು ನನ್ನನ್ನು ಕೇಳಲು ಹಿಂಜರಿಯಬೇಡಿ.

ಫೋನ್ ಸಂಖ್ಯೆಗಳನ್ನು ಬರೆದಿರುವ ಈ ಕಾಗದ ಪತ್ರವನ್ನು ನಿಮ್ಮೊಂದಿಗೆ ತೆಗೆದುಕೊಂಡು ಹೋಗಲು ನಿಮಗೆ ಅನುಕೂಲಕರವಾಗಿದೆಯೇ ಎಂದು ನಾನು ತಿಳಿಯಲು ಬಯಸುತ್ತೇನೆ. ಇಲ್ಲದಿದ್ದರೆ, ನೀವು ಬೆಂಬಲಕ್ಕಾಗಿ ನಮ್ಮನ್ನು ಸಂಪರ್ಕಿಸಲು ನಾವು ಇತರ ಮಾರ್ಗಗಳನ್ನು ಹೊಂದಿದ್ದೇವೆ ನಾನು ಈ ಸಂಖ್ಯೆಗಳನ್ನು ನಿಮ್ಮ ಫೋನ್ನಲ್ಲಿ ಲಾಕ್ ಮಾಡಿದ ಫೋಲ್ಡರ್‌ನಲ್ಲಿ ಶೇಖರಿಸಿಡಲು ಸಹಾಯ ಮಾಡುತ್ತೇನೆ ಇತರರು ನೋಡಲು ಸಾಧ್ಯವಾಗದ ರೀತಿಯಲ್ಲಿ ಮತ್ತು ನೀವು ಅವುಗಳನ್ನು ಹೇಗೆ ನೋಡಬಹುದು ಎಂದು ನಿಮಗೆ ತೋರಿಸಬಹುದು ಈ ಕಾಗದ ಪತ್ರವನ್ನು ಆಶಾ ಅವರೊಂದಿಗೆ ಇಟ್ಟುಕೊಳ್ಳಲು ನಾನು ಕೇಳಬಹುದು, ಇದರಿಂದ ನೀವು ಅವರನ್ನು ಸಂಪರ್ಕಿಸಬಹುದು ಮತ್ತು ಅವರು ನಿಮಗೆ ಈ ಸಂಖ್ಯೆಗಳನ್ನು ನೀಡಬಹುದು [ನಿರ್ದಿಷ್ಟ ಆಶಾ ಅವರ ಹೆಸರನ್ನು ಕೇಳಿ ಮತ್ತು ಅದನ್ನು ಗಮನಿಸಿ]. ನೀವು ಬಯಸಿದರೆ, ನೀವು ನಮ್ಮ ಅಧ್ಯಯನದ ಫೋನ್ ಸಂಖ್ಯೆಯನ್ನು ಮಾತ್ರ ನಿಮ್ಮೊಂದಿಗೆ ಇಟ್ಟುಕೊಳ್ಳಬಹುದು ಮತ್ತು ನೀವು ಸಂಪನ್ಮೂಲಗಳಿಗೆ ಸಂಪರ್ಕ ಹೊಂದಲು ಬಯಸಿದರೆ ನೇರವಾಗಿ ನಮ್ಮ ಸಂಶೋಧನಾ ಸಿಬ್ಬಂದಿಗೆ ಕರೆ ಮಾಡಬಹುದು.

*. [Note down the option opted for by the participant].*

ನೀವು ಅಸುರಕ್ಷಿತರಾಗಿದ್ದರೆ ಅಥವಾ ನಿಮಗೆ ಗಾಯವಾಗುವ ಸಾಧ್ಯತೆಯಿದೆ ಎಂದು ನೀವು ಭಾವಿಸಿದರೆ ದಯವಿಟ್ಟು ನಮ್ಮನ್ನು (ನಮ್ಮ ಅಧ್ಯಯನದ ಫೋನ್ ಸಂಖ್ಯೆಯಲ್ಲಿ) ಸಂಪರ್ಕಿಸಲು ಹಿಂಜರಿಯಬೇಡಿ. ನಿಮ್ಮ ಸುರಕ್ಷತೆಯು ಅತ್ಯಂತ ಆದ್ಯತೆಯಾಗಿದೆ ಮತ್ತು ನೀವು ನಮ್ಮೊಂದಿಗೆ ಸಂಪರ್ಕದಲ್ಲಿದ್ದಾಗ ನೀವು ಸುರಕ್ಷಿತ ಕೈಯಲ್ಲಿರುವುದನ್ನು ಖಚಿತಪಡಿಸಿಕೊಳ್ಳಲು ನಾವು ನಮ್ಮ ಕೈಲಾದಷ್ಟು ಪ್ರಯತ್ನಿಸುತ್ತೇವೆ.

ಅಧ್ಯಯನದ ಫೋನ್ ಸಂಖ್ಯೆ: ನವೀಕರಿಸಲು.

**Appendix I**

**Basic Contact Sheet**


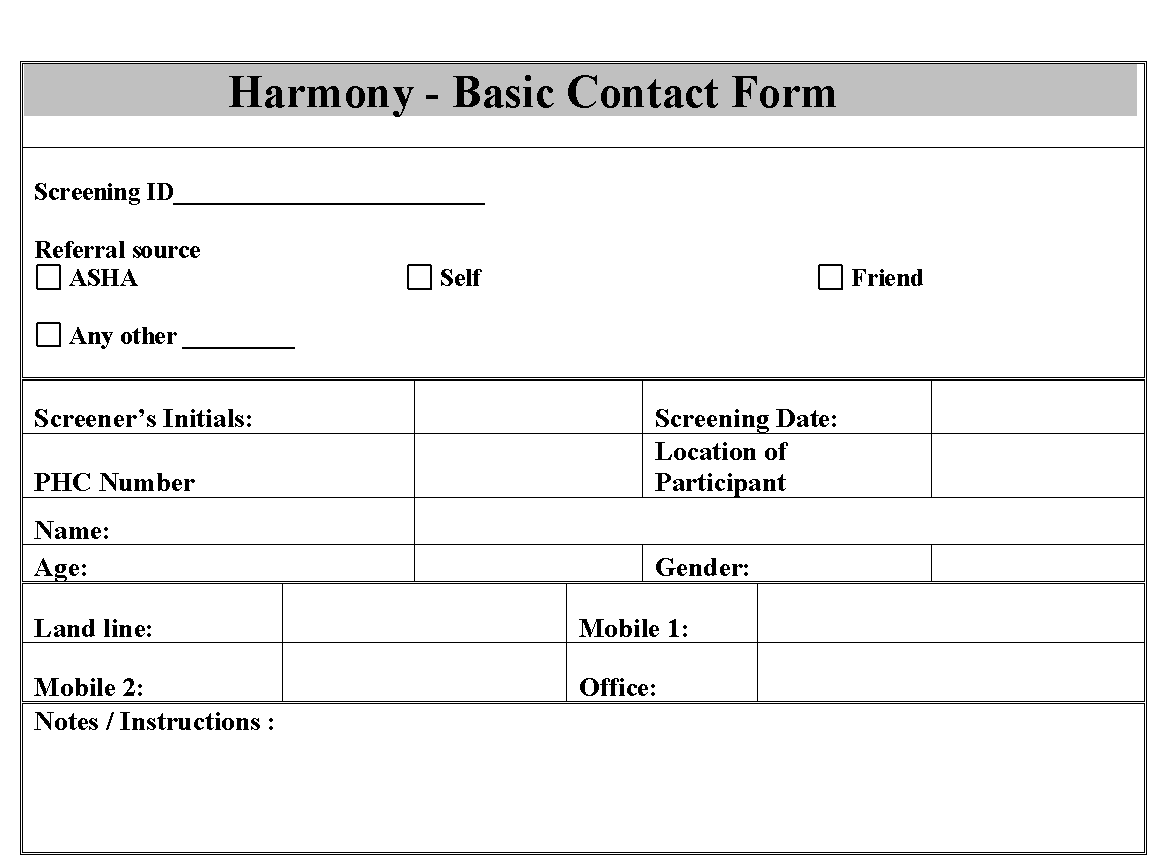


**Appendix J**

**Script for the assessment team member while contacting husband.**

Greetings! My name is _____. I am calling on behalf of BBMP and St. John’s Hospital to tell you about our new research project called Harmony. Is now a good time talk, Mr. ____?

Our program is designed to help couples improve their relationship, by working on trust, healthy communication, and solving problems, to deal with inevitable challenges that we all experience in life. [**Say this only to husbands whose wives said that they would tell their husbands:* We are speaking to everyone who comes to the PHC to see the doctor and telling them about our program. We spoke to your wife at the PHC when she had come here for consultation yesterday/(mention day). After explaining what the program is about to her, she told us that she would tell you about the study and that we would call…] Can I give you a brief description of our program right now?

…

We all face difficult and stressful situations in life, especially in our relationships. Marriages can be hard to navigate and relationship issues can often contribute to a lot of stress. As I mentioned earlier, our program here is designed to help couples improve their relationship. This will be done through counselling sessions provided by our trained staff. A part of these counselling sessions is focused on understanding one’s drinking patterns, since that can also contribute to stress and challenges in one’s marriage.

Before I tell you more, I would like to assure that we will maintain complete confidentiality of your identifying details from our side, so that nobody outside the study knows about your participation, your relationship, or any other matters discussed with us.

Do you have any questions about what we have talked about this far? Please feel free to ask me.

In order to better understand if you can participate in the program and to provide you more information, we would like you to come by the PHC some time. Our conversation at the PHC may go on for about an hour…I’m letting you know so that you can schedule your visit accordingly. What day would work for you? Time? I will be calling you a day before that just to confirm that you will be coming, if that’s okay.

It was nice speaking to you, Mr. _____. Thank you for your time! Looking forward to speaking with you in person on ____. Have a good day!

**ಪತಿಯನ್ನು ಸಂಪರ್ಕಿಸಲು ಮೌಲ್ಯಮಾಪನ ತಂಡದ ಸದಸ್ಯರು ಬಳಸುವ ಸ್ಕ್ರಿಪ್ಟ್**

ನಮಸ್ಕಾರ, ನನ್ನ ಹೆಸರು ............ ನಾನು ಸೇಂಟ್ ಜಾನ್ಸ್ ಸಂಶೋಧನಾ ಸಂಸ್ಥೆಯಿಂದ ಕರೆಮಾಡುತಿದ್ದೆನೆ,

ನಾವು ಹಾರ್ಮನಿ ಎಂಬ ಪ್ರಾಜೆಕ್ಟ್ ಮಾಡುತ್ತಿದ್ದೇವೆ . ದಕ್ಷಿಣ ಭಾರತದ ನಗರ ಪ್ರಾಥಮಿಕ ಆರೋಗ್ಯ ಕೇಂದ್ರಗಳಲ್ಲಿ 450 ವಿವಾಹಿತ ಜೋಡಿಗಳಿಗೆ ಸಮಾಲೋಚನೆ ಮೂಲಕ ಗಂಡ-ಹೆಂಡತಿ ಸಂಬಂಧವನ್ನು ಉತ್ತಮಗೊಳಿಸಿ ಮನೆಯ ವಾತಾವರಣವನ್ನು ಸುಖಕರವಾಗಿಸಲು ಮಾಡುವುದಾಗಿರುತ್ತದೆ

ನಿಮ್ಮ ಬಳಿ ಮಾತನಾಡುವ ಮೊದಲು ನಾವು ನಿಮ್ಮ ಹೆಂಡತಿ PHC ಗೆ WOMEN HEALTH APPOINTMENT ಗೆ ಬಂದಾಗ ನಮ್ಮ ಅಧ್ಯಯನದ ಬಗ್ಗೆ ಮಾತನಾಡಿದ್ದೆವು. ಹಾಗು ನಿಮ್ಮ ಫೋನ್ ನಂಬರ್ ತೆಗೆದುಕೊಂಡೆವು ನೀವು ನಮ್ಮ ಅಧ್ಯಯನದಲ್ಲಿ ಭಾವಹಿಸಲು ಬಯಸಿ PHC ಗೆ ಬಂದರೆ ನಮ್ಮ ಅಧ್ಯಯನದ ಬಗ್ಗೆ ಇನಷ್ಟು ಮಾಹಿತಿ ತಿಳಿಸಿಕೊಡುತ್ತೇವೆ.

**Appendix K**

**Referral letters to NIMHANS and/or to the nearest general hospital**

## **To,** ID No:

Date:

Dear Consultant Psychiatrist/Physician,

Project Harmony is a research study at St John's Research Institute, Bengaluru approved by the Government of India. This study implements and evaluates a community level psychosocial intervention that targets alcohol use disorder in men and intimate partner violence in couples residing under the jurisdiction of the ____________________ Primary Health Centre.

Mr./Ms. ______________, who is a participant in the study/is ineligible to participate in our study, was identified to present with the following.

- Alcohol Use Disorder (AUDIT-C score=_______ or AUDIT score=______)
- Severe dependence (SADQ score=______)
- Severe withdrawal symptoms (CIWA-AR score=_____)
- Clinically significant depressive symptoms with risk of self-harm/suicide (PHQ score=_____)

(Tick whichever is applicable).

We are hereby referring Mr./Ms. ________ to your institution for medical and/or psychiatric consultation. We would be grateful if you could do the needful and provide the individual with the necessary medical advice and treatment.

Thank you.

Sincerely,

Dr. K. Srinivasan

Main Principal Investigator

Project Harmony

St. John’s Research Institute

St. John’s Medical College and Hospital

Bengaluru, Karnataka - 560034

**Appendix L**

**Script for men who have AUD at screening, want to seek help and are not eligible.**

Hello, ___________.

As you know, we have asked you several questions about your drinking habits. On the basis of our assessment, we calculated a score. This score indicates that your current alcohol consumption is within a range that is considered “problem drinking, “which is also referred to as an “alcohol use disorder”. You have indicated that you would like to seek help with this problem. For that reason, we have written referral letters to the NIMHANS Centre for Addiction Medicine as well as to the nearest general hospital. These letters are signed by the project in-charges who are psychiatrists. This letter also includes a brief description of the “problem drinking” that we mentioned earlier, along with the score that is based on the questions we asked you. This will help the doctor who sees you at NIMHANS or the general hospital better understand your current alcohol consumption patterns. If you wish to seek help, you may go to the NIMHANS OPD on any day other than Sunday, between 8 AM and 12 noon. In case of emergency, you may visit the NIMHANS Emergency Clinic any time on any day, as it is open 24/7.

Do you have any questions? I would be glad to answer them for you to the best of my ability. If I don’t know the answer to a question, I will find it out and get back to you.

**ಪ್ರಾಜೆಕ್ಟ್ ಹಾರ್ಮನಿ - AUD ನೊಂದಿಗೆ ಪುರುಷರನ್ನು ಉಲ್ಲೇಖಿಸಲು ಸ್ಕ್ರಿಪ್ಟ್**

ಹಲೋ, _____.

ನಿಮ್ಮ ಕುಡಿಯುವ ಅಭ್ಯಾಸದ ಕುರಿತು ನಾವು ನಿಮಗೆ ಹಲವಾರು ಪ್ರಶ್ನೆಗಳನ್ನು ಕೇಳಿದ್ದೇವೆ. ನಿಮ್ಮ ಕುಡಿಯುವ ನಡವಳಿಕೆಯ ನಮ್ಮ ಮೌಲ್ಯಮಾಪನದ ಆಧಾರದ ಮೇಲೆ, ನಿಮಗೆ ಅಂಕ ನೀಡಿದ್ದೇವೆ. ಈ ಅಂಕವು ನಿಮ್ಮ ಪ್ರಸ್ತುತ ಆಲ್ಕೋಹಾಲ್ ಸೇವನೆಯು "ಸಮಸ್ಯೆ ಕುಡಿತ" ಎಂದು ಪರಿಗಣಿಸಲ್ಪಡುವ ವ್ಯಾಪ್ತಿಯಲ್ಲಿದೆ ಎಂದು ಸೂಚಿಸುತ್ತದೆ ಮತ್ತು ನಾವು ಇದನ್ನು ಸಾಮಾನ್ಯವಾಗಿ "ಆಲ್ಕೋಹಾಲ್ ಬಳಕೆಯ ಅಸ್ವಸ್ಥತೆ" ಎಂದು ಕರೆಯುತ್ತೇವೆ. ಅದಕ್ಕಾಗಿ ನೀವು ಸಹಾಯವನ್ನು ಪಡೆಯಲು ಬಯಸುತ್ತೀರಿ ಎಂದು ಸೂಚಿಸಿದ್ದೀರಿ. ಆ ಕಾರಣಕ್ಕಾಗಿ, ನಾವು ಇಲ್ಲಿ ನಿಮ್ಹಾನ್ಸ್ ಸೆಂಟರ್ ಫಾರ್ ಅಡಿಕ್ಷನ್ ಮೆಡಿಸಿನ್ ಮತ್ತು ಹತ್ತಿರದ ಜನರಲ್ ಆಸ್ಪತ್ರೆಗೆ ಶಿಪಾರಸ್ಸು ಪತ್ರಗಳನ್ನು ಹೊಂದಿದ್ದೇವೆ. ಈ ಪತ್ರಗಳಿಗೆ ಸ್ವತಃ ಮನೋವೈದ್ಯರಾದ ಪ್ರಾಜೆಕ್ಟ್ ಇನ್‌ಚಾರ್ಜ್‌ಗಳು ಸಹಿ ಮಾಡಿದ್ದಾರೆ. ಈ ಪತ್ರವು ನಾವು ನಿಮಗೆ ಕೇಳಿದ ಪ್ರಶ್ನೆಗಳ ಅಂಕದೊಂದಿಗೆ ನಾವು ಮೊದಲೇ ಪ್ರಸ್ತಾಪಿಸಿದ “ಕುಡಿಯುವ ಸಮಸ್ಯೆ” ಯ ಸಂಕ್ಷಿಪ್ತ ವಿವರಣೆಯನ್ನು ಸಹ ಹೊಂದಿದೆ, ಇದರಿಂದ ನಿಮ್ಹಾನ್ಸ್ ಅಥವಾ ಜನರಲ್ ಆಸ್ಪತ್ರೆಯಲ್ಲಿ ನಿಮ್ಮನ್ನು ನೋಡುವ ವೈದ್ಯರು ನಿಮ್ಮ ಪ್ರಸ್ತುತ ಆಲ್ಕೊಹಾಲ್ ಸೇವನೆಯ ಬಗ್ಗೆ ಉತ್ತಮ ತಿಳುವಳಿಕೆಯನ್ನು ಹೊಂದಿರುತ್ತಾರೆ. ಮಾದರಿಗಳು. ನೀವು ಸಹಾಯವನ್ನು ಪಡೆಯಲು ಬಯಸಿದರೆ, ನೀವು ಭಾನುವಾರ ಹೊರತುಪಡಿಸಿ ಯಾವುದೇ ದಿನದಲ್ಲಿ 8 AM ಮತ್ತು 12 ಮಧ್ಯಾಹ್ನದ ನಡುವೆ ನಿಮ್ಹಾನ್ಸ್ OPD ಗೆ ಹೋಗಬಹುದು.

ನೀವು ಯಾವುದೇ ಪ್ರಶ್ನೆಗಳನ್ನು ಹೊಂದಿದ್ದೀರಾ? ನನ್ನ ತಿಳುವಳಿಕೆಯ ಮಟ್ಟಿಗೆ ನಿಮಗಾಗಿ ಅವರಿಗೆ ಉತ್ತರಿಸಲು ನಾನು ಸಂತೋಷಪಡುತ್ತೇನೆ.

**Appendix M**

**Study Information Sheet and Consent Forms**

**St John’s Research Institute, Bangalore**

**and the University of California, San Francisco**

**Consent Form Husband**

**Title of the Study**: A randomized controlled trial of combined behavioural couples therapy with motivational interviewing to improve communication and reduce conflicts between couples and decrease harmful drinking among spouse in urban primary health centre, South India.

**1. Background and Purpose of the Study:**

This is a research study that examines the effects of an intervention in reducing alcohol and facilitate communication that reduces conflicts between married couples.

Dr. K. Srinivasan from the St. John’s Research Institute, Bangalore, and his research team will explain the study to you. This study is funded by the United States National Institutes of Health and is being conducted in collaboration with Drs. Maria Ekstrand and Bibhav Acharya from the University of California, San Francisco.

**2. Researcher Statement:**

We are asking you to be in a research study. The purpose of this consent form is to give you the information you will need to help you decide whether to be in the study or not. Please read the form carefully. You may ask questions about the purpose of the research, what we would ask you to do, the possible risks and benefits, your rights as a volunteer, and anything else about the research or this form that is not clear. When we have answered all your questions, you can decide if you want to be in the study or not. This process is called “informed consent”. We will give you a copy of this form for your records.

**3. How many people will take part in this study?**

About 420 married couples will take part in this study.

**4. What is being done as a part of the study?**

If you choose to participate in this study, the following will happen:

- You will be interviewed every three months for a year about issues related to your relationship with your spouse, regarding reducing conflicts in your relationship and reducing harmful drinking. Each time, your spouse will be interviewed separately as well.
- Each interview will take approximately an hour and will be held in a private room at a time that is convenient for both you and the interviewer.
- You will also be asked to use a Breathalyzer device to measure your alcohol level in your blood. This will happen once a day for one week after the first interview, and again for one week before each of the remaining four interviews. To take an alcohol measurement, you simply need to blow once into a straw connected to the device. The device will show the alcohol level it measured. The Breathalyzer will be issued to you by a member of our study team. They will demonstrate you how to use it. Our staff will coordinate with you to collect the device on the day of your interview and return it to the study team. If you want to know the test results, the study interviewer will share them with you
- You will have a 50% chance of being invited after your first interview to participate in an intervention program that is designed to help you reduce your alcohol use and help you and your wife to improve communication and reduce conflicts. It is decided completely at random (like a lottery) if you will be in the intervention group or in the group that receives a delayed shorter intervention.

- If you are part of the 10-session intervention program, you will first receive four weekly sessions with a study counsellor about your alcohol use; in the fourth session, your wife will attend with you. After that, you and your wife will participate together in six more weekly sessions of about one hour each that can help improve communication and support between the husband and the wife.
- If you are in the delayed intervention program, you will be offered a total of 5 sessions, including one brief educational program in the next two months about alcohol use disorder and referrals to de-addiction services to help in reducing your alcohol use. In addition to this, after your last interview at 12 months, we will offer you and your wife the chance to attend four, one-hour long counselling sessions conducted by a study counsellor. In this, you will attend the first session alone to discuss your alcohol use, and then three sessions with your wife to help understand your alcohol use and improve communication and support between the husband and the wife.
- Since this program is meant to help improve communication with your spouse, both of you must agree to take part in the study. If either you or your spouse decline, neither of you can take part.

**5. Risks and Discomforts**:

You may experience some difficulty or discomfort talking about your relationship experiences or answering some of our other questions, but you are free to refuse to answer any questions that make you uncomfortable. While the members of the study team will not share anything that you tell us with others and we have put multiple measures in place to keep your information private (see section 9), there is always a small risk of loss of confidentiality. Although we have not found this in our previous programs, it is also possible that discussing tensions in your relationship with your counsellor can lead to more tensions or conflicts while you are at home.

**6. Benefits:**

Your response to the interview questions will help us understand factors that influence communication and conflicts between couples. There are no direct benefits to you for participating in this study.

**7. Reimbursement:**

In return for your time, effort and travel expenses, both you and your spouse will receive:

• Rs. 350 for completing the first interview

• Rs. 400 for completing 3-month follow-up

• Rs. 450 for completing 6-month follow-up

• Rs. 500 for completing 9-month follow-up

• Rs. 550 for completing 12-month follow-up

If you are in the intervention group, you and your spouse will receive a travel reimbursement of approximately Rs. 200 each time you attend an intervention session.

In the case one of you has to miss work to attend an intervention session, we will pay you Rs. 450 as compensation for lost wages. If both you and your spouse have to miss work to attend, we will reschedule to a more convenient time.

If you blow into the breathalyzer at least 4 days in a row in the week before your 12-month follow-up, you will receive Rs. 200.

**8. Alternative Procedures**:

You are free to choose not to participate in the study, and we assure you that your decision will not have any negative consequences. It will not affect your regular treatment in the PHC. You are also free to stop participating in the study at any moment and for any reason without any negative consequences to you.

**9. Confidentiality:**

We will do our best to make sure that the personal information gathered for this study is kept private and we will never use your name or personal information if the study findings are published or presented in scientific meetings. We may share your research information with other scientists in the future, but we will never share any information that could identify you.

Organizations that may look at and/or copy your de-identified research records for research, quality assurance, and data analysis include:

The US National Institutes of Health (who is funding this study), UCSF’s Institutional Review Board, and St. John’s Institutional Ethical Review Board.

Again, none of these groups will have access to any information that could identify you.

A description of this clinical trial will be available on http://www.ClinicalTrials.gov, as required by U.S. Law and and https://ctri.nic.in/. These websites will not include information that can identify you. At most, the websites will include a summary of the results. You can search these websites at any time.

For any clarifications/questions at any point of time, contact any of the below-mentioned study personnel:

Dr. K. Srinivasan. 9845038195

The Institutional Ethical Committee of St. John’s Medical College Bangalore, which reviews the ethics of all studies with human subjects, has approved this study. If you have any questions or concerns about this study, you should contact:

Dr. Jayanthi Savio,

Member Secretary,

Institutional Ethics Committee

Ground Floor, (Next to Cardinal Gracias Hall/Room No. 117)

St. John’s Medical College, Sarjapur Road, Bangalore – 560034

Contact No: 080-49466346/48) / Email: [sjmc.ierb@stjohns.in](mailto:sjmc.ierb@stjohns.in)

**CONSENT FORM**

Participant's Statement:

I voluntarily accept to participate in the study conducted by the staff of St. John's Research Institute and the University of California, San Francisco. The nature, demands, and hazards involved in this study have been fully explained to me. I understand that I may withdraw from this study at any time for any reason without it affecting my medical treatment in the PHC/hospital.

I consent to the release of scientific data resulting from my participation in this study to the Principal Investigators for use by them for scientific purposes. The Principal Investigators will do their utmost to ensure that my privacy and confidentiality are protected. I understand that only Dr. Srinivasan, the project manager, and St John’s study staff who need to contact me will have access to any information that could identify me. Data that may be shared with other study team members or other scientists will not include any names.

I also understand that by my participation in this study, I am not waiving any of my legal rights. I understand that in the case of any problem I can contact the concerned investigators in this study or the St John’s Institutional Ethical Committee.

Date: ________________________________________

Name of the Husband: __________________________

Signature: ____________________________________

Witness: ______________________________________

Investigator's Statement:

Your participation in this study will help us better understand communication and conflicts in marital relationships and design programs to reduce conflicts and improve communication.

I have carefully explained the nature, demands, and foreseeable risks of the above study to the subject.

Date:_________________ Signature:__________________________

Name:_____________________________

**ಸೇಂಟ್ ಜಾನ್ಸ್ ಸಂಶೋಧನಾ ಸಂಸ್ಥೆ, ಬೆಂಗಳೂರು**

**ಮತ್ತು ಕ್ಯಾಲಿಫೋರ್ನಿಯಾ ವಿಶ್ವವಿದ್ಯಾಲಯ, ಸ್ಕ್ಯಾನ್ ಫ್ರಾನ್ಸಿಸ್ಕೋ**

**ಒಪ್ಪಿಗೆ ಪತ್ರ – ಪತಿ**

**ಅಧ್ಯಯನದ ಶೀರ್ಷಿಕೆ:** ದಂಪತಿಗಳ ನಡುವಿನ ಸಂವಹನವನ್ನು ಸುಧಾರಿಸಿ, ಜಗಳಗಳನ್ನು ಕಡಿಮೆ ಮಾಡಲು ಮತ್ತು ಸಂಗಾತಿಯ ಹಾನಿಕಾರಕ ಕುಡಿತವನ್ನು ಕಡಿಮೆ ಮಾಡಲು ದಕ್ಷಿಣ ಭಾರತದ ನಗರ ಪ್ರಾಥಮಿಕ ಆರೋಗ್ಯ ಕೇಂದ್ರಗಳಲ್ಲಿ ಪ್ರೇರಕ ಸಂದರ್ಶನ ಹಾಗು ಸಂಯೋಜಿತ ವರ್ತನೆಯ ಜೋಡಿಗಳ ಚಿಕಿತ್ಸೆಯ ಯಾದೃಚ್ಛಿಕ ನಿಯಂತ್ರಿತ ಪ್ರಯೋಗ.

**1. ಅಧ್ಯಯನದ ಹಿನ್ನೆಲೆ ಮತ್ತು ಉದ್ದೇಶ:**

ಈ ಸಂಶೋಧನಾ ಅಧ್ಯಯನವು ಕುಡಿತವನ್ನು ಕಡಿಮೆಗೊಳಿಸಿ, ವಿವಾಹಿತ ದಂಪತಿಗಳ ಮಧ್ಯೆ ಸಂವಹನವನ್ನು ಸುಗಮಗೊಳಿಸಿ ಅವರ ನಡುವಿನ ಜಗಳವನ್ನು ಕಡಿಮೆಗೊಳಿಸುವಲ್ಲಿ ಮಧ್ಯಸ್ಥಿಕೆಯ ಕಾರ್ಯಕ್ರಮದ ಪರಿಣಾಮಗಳನ್ನು ಪರಿಶೀಲಿಸುವುದಾಗಿದೆ.

ಬೆಂಗಳೂರಿನ ಸೇಂಟ್ ಜಾನ್ಸ್ ಸಂಶೋಧನಾ ಸಂಸ್ಥೆಯ ಡಾ. ಕೆ. ಶ್ರೀನಿವಾಸನ್ ಮತ್ತು ಅವರ ಸಂಶೋಧನಾ ತಂಡವು ಈ ಅಧ್ಯಯನದ ಬಗ್ಗೆ ನಿಮಗೆ ವಿವರಿಸುತ್ತಾರೆ. ಈ ಅಧ್ಯಯನ ಯುನೈಟೆಡ್ ಸ್ಟೇಟ್ಸ್ ನ್ಯಾಷನಲ್ ಇನ್‌ಸ್ಟಿಟ್ಯೂಟ್ ಆಫ್ ಹೆಲ್ತ್‌ನಿಂದ ಧನಸಹಾಯ ದೊರೆಯುತ್ತದೆ ಮತ್ತು ಸ್ಯಾನ್ ಫ್ರಾನ್ಸಿಸ್ಕೋದ ಕ್ಯಾಲಿಫೋರ್ನಿಯಾ ವಿಶ್ವವಿದ್ಯಾಲಯದ ಡಾ. ಮಾರಿಯಾ ಎಕ್ಸ್‌ಟ್ರಾಂಡ್ ಮತ್ತು ಬಿಭವ್ ಆಚಾರ್ಯ ಅವರ ಸಹಯೋಗದೊಂದಿಗೆ ನಡೆಸಲಾಗುತ್ತಿದೆ.

**2. ಸಂಶೋಧಕರ ಹೇಳಿಕೆ:**

ಈ ಸಂಶೋಧನಾ ಅಧ್ಯಯನದಲ್ಲಿರಲು ನಾವು ನಿಮ್ಮನ್ನು ಕೇಳುತ್ತಿದ್ದೇವೆ. ಈ ಸಮ್ಮತಿ ಪತ್ರದ ಉದ್ದೇಶವು ಅಧ್ಯಯನದಲ್ಲಿ ಇರಬೇಕೇ ಅಥವಾ ಬೇಡವೇ ಎಂಬುದನ್ನು ನಿರ್ಧರಿಸಲು ನಿಮಗೆ ಸಹಾಯ ಮಾಡುವ ಮಾಹಿತಿಯನ್ನು ನಿಮಗೆ ನೀಡುವುದಾಗಿದೆ. ದಯವಿಟ್ಟು ಈ ಸಮ್ಮತಿ ಪತ್ರವನ್ನು ಎಚ್ಚರಿಕೆಯಿಂದ ಓದಿ. ಸಂಶೋಧನೆಯ ಉದ್ದೇಶ, ನೀವು ಏನು ಮಾಡಬೇಕು, ನಾವು ನಿಮ್ಮನ್ನು ಏನು ಮಾಡಬೇಕೆಂದು ಕೇಳುತ್ತೇವೆ, ಸಂಭವನೀಯ ಅಪಾಯಗಳು ಮತ್ತು ಪ್ರಯೋಜನಗಳು, ಸ್ವಂತ ಇಚ್ಛೆಯಿಂದ ಭಾಗವಹಿಸುತ್ತಿರುವ ನಿಮ್ಮ ಹಕ್ಕುಗಳು ಮತ್ತು ಇನ್ನಿತರೆ ಸಂಶೋಧನ ಅಥವಾ ಸಮ್ಮತಿ ಪತ್ರದಲ್ಲಿ ಸ್ಪಷ್ಟವಾಗಿಲ್ಲದ ಬೇರೆ ಯಾವುದನ್ನಾದರೂ ಕುರಿತು ನೀವು ಪ್ರಶ್ನೆಗಳನ್ನು ಕೇಳಬಹುದು ನಿಮ್ಮ ಎಲ್ಲಾ ಪ್ರಶ್ನೆಗಳಿಗೆ ನಾವು ಉತ್ತರಿಸಿದ ನಂತರ ನೀವು ಅಧ್ಯಯನದಲ್ಲಿ ಇರಬೇಕೆ ಅಥವಾ ಬೇಡವೇ ಎಂಬುದನ್ನು ನಿರ್ಧರಿಸಬಹುದು. ಈ ಪ್ರಕ್ರಿಯೆಯನ್ನು ಮಾಹಿತಿಯುಳ್ಳ ಸಮ್ಮತಿ” ಎಂದು ಕರೆಯಲಾಗುತ್ತದೆ, ದಾಖಲೆಗಳಿಗಾಗಿ ನಾವು ಈ ಪತ್ರದ ನಕಲು ಪ್ರತಿಯನ್ನು ನಿಮಗೆ ನೀಡುತ್ತೇವೆ.

**3. ಈ ಅಧ್ಯಯನದಲ್ಲಿ ಎಷ್ಟು ಜನರು ಭಾಗವಹಿಸುತ್ತಾರೆ?**

ಸುಮಾರು 420 ವಿವಾಹಿತ ಜೋಡಿಗಳು ಈ ಅಧ್ಯಯನದಲ್ಲಿ ಪಾಲ್ಗೊಳ್ಳಲಿದ್ದಾರೆ.

**4. ಅಧ್ಯಯನದ ಭಾಗವಾಗಿ ಏನು ಮಾಡಲಾಗುತ್ತಿದೆ?**

ನೀವು ಈ ಅಧ್ಯಯನದಲ್ಲಿ ಭಾಗವಹಿಸಲು ಆಯ್ಕೆ ಮಾಡಿದರೆ, ಈ ಕೆಳಗಿನವುಗಳು ಸಂಭವಿಸುತ್ತವೆ:

- ನಿಮ್ಮ ಸಂಬಂಧದಲ್ಲಿನ ಜಗಳಗಳನ್ನು ಕಡಿಮೆ ಮಾಡುವ ಮತ್ತು ಹಾನಿಕಾರಕ ಮದ್ಯಪಾನವನ್ನು ಕಡಿಮೆ ಮಾಡುವ ಕುರಿತು ನೀವು ಪ್ರತಿ ಮೂರು ತಿಂಗಳಿಗೊಮ್ಮೆ ಸಂದರ್ಶಿಸಲ್ಪಡುತ್ತೀರಿ. ಪ್ರತಿ ಬಾರಿಯೂ ನಿಮ್ಮ ಸಂಗಾತಿಯನ್ನು ಪ್ರತ್ಯೇಕವಾಗಿ ಸಂದರ್ಶನ ಮಾಡಲಾಗುತ್ತದೆ.
- ಪ್ರತಿ ಸಂದರ್ಶನವು ಸರಿಸುಮಾರು ಒಂದು ಗಂಟೆ ತೆಗೆದುಕೊಳ್ಳುತ್ತದೆ. ನಿಮಗೆ ಮತ್ತು ಸಂದರ್ಶಕರಿಗೆ ಅನುಕೂಲಕರವಾದ ಸಮಯದಲ್ಲಿ ಖಾಸಗಿ ಕೊಠಡಿಯಲ್ಲಿ ನಡೆಯುತ್ತದೆ.
- ನಿಮ್ಮ ರಕ್ತದಲ್ಲಿ ಮದ್ಯದ ಮಟ್ಟವನ್ನು ಅಳೆಯಲು ನೀವು ಬ್ರೆತ್‌ಅನ್ಲೈಸರ್ (Breathalyzer) ಎಂಬ ಉಪಕರಣವನ್ನು ಬಳಸಲು ಕೇಳಲಾಗುತ್ತದೆ. ಇದು ಮೊದಲ ಸಂದರ್ಶನದ ನಂತರ ಒಂದು ವಾರದವರೆಗೆ ಪ್ರತಿ ದಿನ ಒಂದು ಬಾರಿ ನಡೆಯುತ್ತದೆ ಮತ್ತು ನಂತರದ ನಾಲ್ಕು ಸಂದರ್ಶನಗಳ ಪ್ರತ್ಯೇಕ ವಾರಗಳಿಗೂ ಇದೇ ರೀತಿ ಮತ್ತೆ ನಡೆಯುತ್ತದೆ. ಮದ್ಯದ ಮಟ್ಟವನ್ನು ಅಳೆಯಲು, ನೀವು ಉಪಕರಣಕ್ಕೆ ಜೋಡಿಸಿದ ಸ್ಟ್ರಾವ್‌ನಲ್ಲಿಗೆ ಒಂದು ಬಾರಿ ಊದಬೇಕಾಗುತ್ತದೆ. ಉಪಕರಣವು ಅಳೆಯಲಾದ ಮದ್ಯದ ಮಟ್ಟವನ್ನು ತೋರಿಸುತ್ತದೆ.

ಬ್ರೆತ್‌ಅನ್ಲೈಸರ್ ಅನ್ನು ನಮ್ಮ ಅಧ್ಯಯನ ತಂಡದ ಸದಸ್ಯರು ನಿಮಗೆ ನೀಡುತ್ತಾರೆ. ಅವರು ಇದನ್ನು ಹೇಗೆ ಬಳಸುವುದು ಎಂಬುದನ್ನು ನಿಮಗೆ ತೋರಿಸಿಕೊಡುತ್ತಾರೆ. ಉಪಕರಣವನ್ನು ನಿಮ್ಮ ಸಂದರ್ಶನದ ದಿನದಲ್ಲಿ ನೀಡುವುದು ಮತ್ತು ಮತ್ತೆ ಅಧ್ಯಯನ ತಂಡಕ್ಕೆ ಹಿಂತಿರುಗಿಸುವ ಪ್ರಕ್ರಿಯೆಯಲ್ಲಿ ನಮ್ಮ ಸಿಬ್ಬಂದಿ ನಿಮಗೆ ಸಹಕಾರ ನೀಡುತ್ತಾರೆ.

ನೀವು ಪರೀಕ್ಷೆಯ ಫಲಿತಾಂಶಗಳನ್ನು ತಿಳಿದುಕೊಳ್ಳಲು ಇಚ್ಛಿಸಿದರೆ, ಅಧ್ಯಯನ ಸಂದರ್ಶನಗಾರರು ಅವುಗಳನ್ನು ನಿಮ್ಮೊಂದಿಗೆ ಹಂಚಿಕೊಳ್ಳುತ್ತಾರೆ.

- ನಿಮಗೆ ಮತ್ತು ನಿಮ್ಮ ಹೆಂಡತಿಗೆ ನಿಮ್ಮ ಕುಡಿತವನ್ನು ಕಡಿಮೆ ಮಾಡಲು ಸಹಾಯ ನೀಡುವ ಮತ್ತು ನಿಮ್ಮಿಬ್ಬರ ನಡುವಿನ ಮಾತುಕಥೆ ಉತ್ತಮಗೊಳ್ಳಿಸುವ ಮತ್ತು ಸಂಘರ್ಷವನ್ನು ಕಡಿಮೆ ಮಾಡಲು ಸಹಾಯ ನೀಡುವ ಒಂದು ಮಧ್ಯಸ್ಥಿಕೆ ಯೋಜನೆಯಲ್ಲಿ ಭಾಗವಹಿಸಲು 50% ಅವಕಾಶ ಇರುವುತ್ತದೆ.
- ನೀವು ಆ ಮಧ್ಯಸ್ಥಿಕೆ ಯೋಜನೆಯಲ್ಲಿ ಭಾಗವಹಿಸಿದ್ದರೆ, ನಮ್ಮ ಅಧ್ಯಯನದ ಕೌನ್ಸೆಲರ್ ನಿಮಗೆ ನಿಮ್ಮ ಕುಡಿತದ ಬಗ್ಗೆ 4 ಸಭೆಗಳು ನೀಡುತ್ತಾರೆ; ನಾಲಕ್ಕನೆ ಸಭೆಯಲ್ಲಿ, ನೀವು ಮತ್ತು ನಿಮ್ಮ ಹೆಂಡತಿ ಸಂಬಂಧದಲ್ಲಿ ಕುಡಿತದ ಪಾತ್ರವನ್ನು ತಿಳಿಯಲು ನಿಮ್ಮ ಹೆಡತಿಯೂ ಹಾಜರಾಗುತ್ತೀರಿ. ಅದಾದಮೇಲೆ, ನೀವು ಮತ್ತು ನಿಮ್ಮ ಹೆಂಡತಿ ನಿಮ್ಮಿಬ್ಬರ ನಡುವಿನ ಮಾತುಕಥೆ ಮತ್ತು ಗಂಡ-ಹೆಂಡತಿಯ ನಡುವಿನ ಬೆಂಬಲವನ್ನು ಉತ್ತಮಗೊಳ್ಳಿಸಲು ಇನ್ನೂ 6 ಸಭೆಗಳಲ್ಲಿ ಭಾಗವಿಸುತ್ತೀರಿ.
- ನೀವು ಮತ್ತು ನಿಮ್ಮ ಹೆಂಡತಿ ತಡವಾದ ಮಧ್ಯಸ್ಥಿಕೆ ಯೋಜನೆಯಲ್ಲಿ ಭಾಗವಹಿಸಿದ್ದರೆ, ನಿಮಗೆ ಮದ್ಯಪಾನದ ಸಮಸ್ಯೆಯ ಬಗ್ಗೆ ಮತ್ತು ಡಿ-ಅಡಿಕ್ಷನ್ ಸೇವೆಗಳಿಗೆ ಹೋಗಲು ಮಾಹತಿ ನೀಡಲಾಗುತ್ತದೆ. ಜೊತೆಗೆ, ಈ ಅಧ್ಯಯನದ ಕೊನೆಯ 12 ತಿಂಗಳ ಸಂದರ್ಶನದ ನಂತರ, ನೀವು ಮತ್ತು ನಿಮ್ಮ ಹೆಂಡತಿಯನ್ನು 4, ಒಂದು ಘಂಟೆಯ ಕೌನ್ಸೆಲಿಂಗ್ ಸಭೆಗಳಲ್ಲಿ ಭಾಗವಹಿಸಲು ಆಹ್ವಾನಿಸಲಾಗುತ್ತದೆ. ಇದರಲ್ಲಿ, ನೀವು ಮೊದಲನೇ ಸಭೆ ಒಬ್ಬರೇ ಹಾಜಿರಾಗಿ ಕೌನ್ಸೆಲರ್ ಜೊತೆ ನಿಮ್ಮ ಕುಡಿತದ ಬಗ್ಗೆ ಚರ್ಚಿಸುತ್ತೀರಿ. ಅದಾದಮೇಲೆ, ಮೂರು ಸಭೆಗಳು ನೀವು ಮತ್ತು ನಿಮ್ಮ ಹೆಂಡತಿ ಇಬ್ಬರೂ ಹಾಜಿರಾಗಿ ನಿಮ್ಮ ಕುಡಿತದ ಬಗ್ಗೆ, ನಿಮ್ಮಿಬ್ಬರ ನಡುವಿನ ಮಾತುಕಥೆ ಮತ್ತು ಬೆಂಬಲವನ್ನು ಉತ್ತಮಗೊಳ್ಳಿಸುವ ಬಗ್ಗೆ ಚರ್ಚಿಸುವ ಅವಕಾಶ ಇರುವುತ್ತದೆ.
- ಈ ಕಾರ್ಯಕ್ರಮವು ನಿಮ್ಮ ಸಂಗಾತಿಯೊಂದಿಗೆ ಸಂವಹನವನ್ನು ಸುಧಾರಿಸಲು ಸಹಾಯ ಮಾಡುವುದರಿಂದ, ನೀವಿಬ್ಬರೂ ಅಧ್ಯಯನದಲ್ಲಿ ಭಾಗವಹಿಸಲು ಒಪ್ಪಿಕೊಳ್ಳಬೇಕು. ನೀವು ಅಥವಾ ನಿಮ್ಮ ಸಂಗಾತಿ ನಿರಾಕರಿಸಿದರೆ, ನೀವಿಬ್ಬರೂ ಭಾಗವಹಿಸಲು ಸಾಧ್ಯವಿಲ್ಲ.

**5. ಅಪಾಯಗಳು ಮತ್ತು ಆರಾಮದಾಯಕವಲ್ಲದ ವಿಷಯಗಳು:**

ನಿಮ್ಮ ಸಂಬಂಧದ ಅನುಭವಗಳ ಬಗ್ಗೆ, ಮಾತನಾಡಲು ಅಥವಾ ನಮ್ಮ ಇತರ ಕೆಲವು ಪ್ರಶ್ನೆಗಳಿಗೆ ಉತ್ತರಿಸಲು ನೀವು ಕೆಲವು ತೊಂದರೆ ಅಥವಾ ಮುಜುಗರವನ್ನು ಅನುಭವಿಸಬಹುದು, ನಿಮಗೆ ಅಹಿತಕರವಾದ ಯಾವುದೇ ಪ್ರಶ್ನೆಗಳಿಗೆ ಉತ್ತರಿಸಲು ನೀವು ಮುಕ್ತವಾಗಿ ನಿರಾಕರಿಸಬಹುದು. ಅಧ್ಯಯನ ತಂಡದ ಸದಸ್ಯರು ನೀವು ನಮಗೆ ಹೇಳುವ ಯಾವುದೇ ವಿಷಯಗಳನ್ನು ಇತರರೊಂದಿಗೆ ಹಂಚಿಕೊಳ್ಳುವುದಿಲ್ಲ. ನಿಮ್ಮ ಮಾಹಿತಿಯನ್ನು ಖಾಸಗಿಯಾಗಿಡಲು ನಾವು ಹಲವಾರು ಕ್ರಮಗಳನ್ನು ತೆಗೆದುಕೊಂಡಿದ್ದೇವೆ (9ನೇ ವಿಯಾಭಗ ನೋಡಿ), ಆದರೂ ಗೌಪ್ಯತೆಯ ಕಳೆದುಕೊಳ್ಳುವ ಅವಕಾಶ ಇರುತ್ತದೆ. ನಿಮ್ಮ ಕೌಂಸೆಲ್ಲೋರ್ ಒಂದಿಗೆ ನಿಮ್ಮ ಸಂಬಂಧದಲ್ಲಿನ ಸಮಸ್ಯೆಗಳನ್ನು ಚರ್ಚಿಸುವುದರಿಂದ ನಿಮ್ಮ ಮನೆ ಅಥವಾ ಸಂಬಂಧದಲ್ಲಿ ಸಮಸ್ಯೆ ಅಥವಾ ಸಂಘರ್ಷಗಳು ಹೆಚ್ಚಾಗುವ ಅಪಾಯವೂ ಇದೆ. ಆದರೆ, ನಮ್ಮ ಹಿಂದಿನ ಯಾವುದೇ ಕಾರ್ಯಕ್ರಮಗಳಲ್ಲಿ ನಾವು ಇದನ್ನು ಕಂಡುಕೊಂಡಿಲ್ಲ.

**6. ಪ್ರಯೋಜನಗಳು:**

ಸಂದರ್ಶನದ ಪ್ರಶ್ನೆಗೆ ನಿಮ್ಮ ಪ್ರತಿಕ್ರಿಯೆಯು ದಂಪತಿಗಳ ನಡುವಿನ ಸಂವಹನ ಮತ್ತು ಜಗಳಗಳ ಮೇಲೆ ಪ್ರಭಾವ ಬೀರುವ ಅಂಶಗಳನ್ನು ಅರ್ಥಮಾಡಿಕೊಳ್ಳಲು ನಮಗೆ ಸಹಾಯ ಮಾಡುತ್ತದೆ. ಈ ಅಧ್ಯಯನದಲ್ಲಿ ಭಾಗವಹಿಸುವುದರಿಂದ ನಿಮಗೆ ಯಾವುದೇ ನೇರ ಪ್ರಯೋಜನಗಳಿಲ್ಲ.

**7. ಮರುಪಾವತಿ:**

ನಿಮ್ಮ ಸಮಯ, ಶ್ರಮ ಮತ್ತು ಪ್ರಯಾಣ ವೆಚ್ಚಗಳಿಗೆ ಪ್ರತಿಯಾಗಿ, ನಿಮಗೆ ಮತ್ತು ನಿಮ್ಮ ಸಂಗಾತಿಗೆ:

- ಮೊದಲ ಸಂದರ್ಶನವನ್ನು ಪೂರ್ಣಗೊಳಿಸಲು 350 ರೂ.
- ಮೂರನೆಯ ತಿಂಗಳ ಮರು ಸಂದರ್ಶನ ಪೂರ್ಣಗೊಳಿಸಲು 400 ರೂ.
- ಆರನೆಯ ತಿಂಗಳ ಮರು ಸಂದರ್ಶನ ಪೂರ್ಣಗೊಳಿಸಲು 450 ರೂ.
- ಒಂಬತ್ತನೆಯ ತಿಂಗಳ ಮರು ಸಂದರ್ಶನ ಪೂರ್ಣಗೊಳಿಸಲು 500 ರೂ
- ಹನ್ನೆರಡನೆಯ ತಿಂಗಳ ಸಂದರ್ಶನ ಪೂರ್ಣಗೊಳಿಸಲು 550 ರೂ ನೀಡಲಾಗುವುದು.

ನೀವು ಮಧ್ಯಸ್ಸಿಕ ಕಾರ್ಯಕ್ರಮದಲ್ಲಿದ್ದರೆ, ನೀವು ಮತ್ತು ನಿಮ್ಮ ಸಂಗಾತಿಯು ಪ್ರತಿ ಭೇಟಿಗೆ ಸರಿಸುಮಾರು 200 ರೂ. ಪ್ರಯಾಣ ಮರುಪಾವತಿಯನ್ನು ಸಹ ಪಡೆಯುತ್ತೀರಿ.

ನಿಮ್ಮಲ್ಲಿ ಯಾರಾದರೂ ಒಬ್ಬರು ಮಧ್ಯಸ್ಥಿಕೆ ಅಧಿವೇಶನಕ್ಕೆ ಹಾಜರಾಗಲು ಕೆಲಸಕ್ಕೆ ರಜೆ ಹಾಕುವ ಪರಿಸ್ಥಿತಿ ಬಂದರೆ, ಕಳೆದುಹೋದ ವೇತನಕ್ಕೆ ಪರಿಹಾರವಾಗಿ ನಾವು ನಿಮಗೆ ₹450 ಪಾವತಿಸುತ್ತೇವೆ. ನೀವು ಮತ್ತು ನಿಮ್ಮ ಸಂಗಾತಿಯು ಇಬ್ಬರೂ ಹಾಜರಾಗಲು ಕೆಲಸಕ್ಕೆ ರಜೆ ಹಾಕುವ ಪರಿಸ್ಥಿತಿ ಬಂದರೆ, ಮತ್ತೊಂದು ದಿನ ಇನ್ನೂ ಹೆಚ್ಚು ಅನುಕೂಲಕರ ಸಮಯಕ್ಕೆ ಮಧ್ಯಸ್ಥಿಕೆ ಅಧಿವೇಶನವನ್ನು ಮುಂದೂಡುತೇವೆ.

ನೀವು 12ನೇ ತಿಂಗಳ ಅನುಸರಣಾ ಸಂದರ್ಶನದ ಮೊದಲಿನ ವಾರದಲ್ಲಿ ಕನಿಷ್ಠ ನಾಲ್ಕು ದಿನಗಳ ಕಾಲ ಮುಂದುವರಿದು ಬ್ರೆತ್‌ಅನ್ಲೈಸರ್‌ಗೆ ಊದಿದರೆ, ನಿಮಗೆ ರೂ. 200 ನೀಡಲಾಗುತ್ತದೆ.

**8. ಪರ್ಯಾಯ ಕಾರ್ಯವಿಧಾನಗಳು:**

ಅಧ್ಯಯನದಲ್ಲಿ ಭಾಗವಹಿಸದಿರಲು ನೀವು ಸ್ವತಂತ್ರರಾಗಿದ್ದೀರಿ ಮತ್ತು ನಿಮ್ಮ ನಿರ್ಧಾರವು ಯಾವುದೇ ಋಣಾತ್ಮಕ ಪರಿಣಾಮಗಳನ್ನು ಹೊಂದಿರುವುದಿಲ್ಲ ಎಂದು ನಾವು ನಿಮಗೆ ಭರವಸೆ ನೀಡುತ್ತೇವೆ. ಇದು ಪಿ.ಹೆಚ್.ಸಿಯಲ್ಲಿ ನಿಮ್ಮ ನಿಯಮಿತ ಚಿಕಿತ್ಸೆಯ ಮೇಲೆ ಪರಿಣಾಮ ಬೀರುವುದಿಲ್ಲ. ನಿಮಗೆ ಯಾವುದೇ ಋಣಾತ್ಮಕ ಪರಿಣಾಮಗಳಿಲ್ಲದೆ ಯಾವುದೇ ಕ್ಷಣದಲ್ಲಿ ಮತ್ತು ಯಾವುದೇ ಕಾರಣಕ್ಕಾಗಿ ಅಧ್ಯಯನದಲ್ಲಿ ಭಾಗವಹಿಸುವುದನ್ನು ನಿಲ್ಲಿಸಲು ನೀವು ಸ್ವತಂತ್ರರಾಗಿದ್ದೀರಿ.

**9. ಗೌಪ್ಯತೆ :**

ಈ ಅಧ್ಯಯನಕ್ಕಾಗಿ ಸಂಗ್ರಹಿಸಲಾದ ವೈಯಕ್ತಿಕ ಮಾಹಿತಿಯನ್ನು ಖಾಸಗಿಯಾಗಿ ಇರಿಸಲಾಗಿದೆಯೆ ಎಂದು ಖಚಿತಪಡಿಸಿಕೊಳ್ಳಲು ನಾವು ನಮ್ಮ ಕೈಲಾದಷ್ಟು ಪ್ರಯತ್ನಿಸುತ್ತೇವೆ ಮತ್ತು ಅಧ್ಯಯನದ ಸಂಶೋಧನೆಗಳನ್ನು ಪ್ರಕಟಿಸಿದರೆ ಅಥವಾ ವೈಜ್ಞಾನಿಕ ಸಭೆಗಳಲ್ಲಿ ಪ್ರಸ್ತುತಪಡಿಸಿದರೆ ನಿಮ್ಮ ಹೆಸರು ಅಥವಾ ವೈಯಕ್ತಿಕ ಮಾಹಿತಿಯನ್ನು ನಾವು ಎಂದಿಗೂ ಬಳಸುವುದಿಲ್ಲ. ನಿಮ್ಮ ಸಂಶೋಧನಾ ಮಾಹಿತಿಯನ್ನು ನಾವು ಭವಿಷ್ಯದಲ್ಲಿ ಇತರ ವಿಜ್ಞಾನಿಗಳೊಂದಿಗೆ ಹಂಚಿಕೊಳ್ಳಬಹುದು, ಆದರೆ ನಿಮ್ಮನ್ನು ಗುರುತಿಸುವ ಯಾವುದೇ ಮಾಹಿತಿಯನ್ನು ನಾವು ಎಂದಿಗೂ ಹಂಚಿಕೊಳ್ಳುವುದಿಲ್ಲ.

ಸಂಶೋಧನೆ, ಗುಣಮಟ್ಟದ ಭರವಸೆ ಮತ್ತುದತ್ತಾಂಶ ವಿಶ್ಲೇಷಣಾ ಸಭೆಗಳಿಗಾಗಿ ನಿಮ್ಮ ಗುರುತಿಸಲಾಗದ ಸಂಶೋಧನಾ ದಾಖಲೆಗಳನ್ನು ನೋಡುವ ಮತ್ತು/ಅಥವಾ ನಕಲಿಸುವ ಸಂಸ್ಥೆಗಳು ಇವುಗಳು :

US ನ್ಯಾಷನಲ್ ಇನ್‌ಸ್ಟಿಟ್ಯೂಟ್ ಆಫ್ ಹೆಲ್ತ್, UCSF ನ ಮಾನವ ಸಂಶೋಧನೆಯ ಸಮಿತಿ, ಮತ್ತು ಸೇಂಟ್ ಜಾನ್ ಇನ್‌ಸ್ಟಿಟ್ಯೂಶನಲ್ ಎಥಿಕಲ್ ರಿವ್ಯೂ ಬೋರ್ಡ್.

ಮತ್ತೊಮ್ಮೆ, ಈ ಗುಂಪುಗಳಲ್ಲಿ ನಿಮ್ಮನ್ನು ಗುರುತಿಸಬಹುದಾದ ಯಾವುದೇ ಮಾಹಿತಿಯನ್ನು ಹಂಚಿಕೊಳ್ಳುವ ಸಾಧ್ಯವಿಲ್ಲ.

ಈ ಕ್ಲಿನಿಕಲ್ ಪ್ರಯೋಗದ ವಿವರಣೆಯು ಯು.ಎಸ್. ಕಾನೂನು ಪ್ರಕಾರ http://www.ClinicalTrials.gov ನಲ್ಲಿ ಲಭ್ಯವಿರುತ್ತದೆ ಮತ್ತು ಮತ್ತು https://ctri.nic.in/ ನಲ್ಲೂ ಲಭ್ಯವಿರುತ್ತದೆ. ಈ ವೆಬ್‌ಸೈಟ್‌ಗಳಲ್ಲಿ ನಿಮ್ಮನ್ನು ಗುರುತಿಸಬಹುದಾದ ಮಾಹಿತಿ ಇರುವುದಿಲ್ಲ. ಹೆಚ್ಚೆಂದರೆ, ವೆಬ್‌ಸೈಟ್‌ಗಳು ಅಧ್ಯಯನದ ಫಲಿತಾಂಶಗಳ ಸಾರಾಂಶವನ್ನು ತೋರಿಸುವುತ್ತಾರೆ. ನೀವು ಯಾವುದೇ ಸಮಯದಲ್ಲಿ ಈ ವೆಬ್‌ಸೈಟ್‌ಗಳನ್ನು ಹುಡುಕಬಹುದು.

ಯಾವುದೇ ಸಮಯದಲ್ಲಿ ಯಾವುದೇ ಸ್ಪಷ್ಟಿಕರಣಗಳು/ಪ್ರಶ್ನೆಗಳಿಗಾಗಿ, ಕಳಗೆ ತಿಳಿಸಲಾದ ಯಾವುದೇ ಅಧ್ಯಯನ ಸಿಬ್ಬಂದಿಯನ್ನು ಸಂಪರ್ಕಿಸಿ:

ಡಾ. ಕೆ.ಶ್ರೀನಿವಾಸನ್ : 9845038195

ಸೇಂಟ್ ಜಾನ್ಸ್ ಮೆಡಿಕಲ್ ಕಾಲೇಜಿನ ಬೆಂಗಳೂರಿನ ಸಂಸ್ಥೆಯ ನೈತಿಕ ಸಮಿತಿಯು, ಮಾನವ ವಿಷಯಗಳೊಂದಿಗೆ ಎಲ್ಲಾ ಅಧ್ಯಯನಗಳ ನೈತಿಕತೆಯನ್ನು ಪರಿಶೀಲಿಸುತ್ತದೆ ಮತ್ತು ಈ ಅಧ್ಯಯನವನ್ನು ಅನುಮೋದಿಸಿದೆ. ಈ ಅಧ್ಯಯನದ ಕುರಿತು ನೀವು ಯಾವುದೇ ಪ್ರಶ್ನೆಗಳನ್ನು ಅಥವಾ ಕಾಳಜಿಗಳನ್ನು ಹೊಂದಿದ್ದರೆ, ನೀವು ಸಂಪರ್ಕಿಸಬೇಕು:

ಡಾ.ಜಯಂತಿ ಸಾವಿಯೋ,

ಸದಸ್ಯ ಕಾರ್ಯದರ್ಶಿ,

ಸಾಂಸ್ಥಿಕ ನೈತಿಕ ಸಮಿತಿ

ನೆಲ ಮಹಡಿ, (ಕಾರ್ಡಿನಲ್ ಗ್ರೇಸಿಯಾಸ್ ಹಾಲ್/ಕೊಠಡಿ ಸಂಖ್ಯೆ 117 ರ ಪಕ್ಕದಲ್ಲಿ)

ಸೇಂಟ್ ಜಾನ್ಸ್ ವೈದ್ಯಕೀಯ ಕಾಲೇಜು, ಸರ್ಜಾಪುರ ರಸ್ತೆ, ಬೆಂಗಳೂರು 560034

ಸಂಪರ್ಕ ಸಂಖ್ಯೆ : 080-49466346/48) / ಇಮೇಲ್ : sjmc.ierb@stjohns.in

**ಒಪ್ಪಿಗೆ ಪತ್ರ**

**ಭಾಗವಹಿಸುವವರ ಹೇಳಿಕೆ:**

ಸೇಂಟ್ ಜಾನ್ಸ್‌ ಸಂಶೋಧನಾ ಸಂಸ್ಥೆ ಮತ್ತು ಸ್ಕ್ಯಾನ್ ಫ್ರಾನ್ಸಿಸ್ಕೋದ ಕ್ಯಾಲಿಫೋರ್ನಿಯಾ ವಿಶ್ವವಿದ್ಯಾಲಯದ ಸಿಬ್ಬಂದಿ ನಡೆಸಿದ ಅಧ್ಯಯನದಲ್ಲಿ ಭಾಗವಹಿಸಲು ನಾನು ಸ್ವಯಂಪ್ರೇರಣೆಯಿಂದ ಒಪ್ಪಿಕೊಳ್ಳುತ್ತೇನೆ. ಈ ಅಧ್ಯಯನದಲ್ಲಿ ಒಳಗೊಂಡಿರುವ ಸ್ವಭಾವ, ಬೇಡಿಕೆಗಳು ಮತ್ತು ಅಪಾಯಗಳನ್ನು ನನಗೆ ಸಂಪೂರ್ಣವಾಗಿ ವಿವರಿಸಲಾಗಿದೆ.

ಪಿ.ಹೆಚ್.ಸಿ/ಆಸ್ಪತ್ರೆಯಲ್ಲಿನ ನನ್ನ ವೈದ್ಯಕೀಯ ಚಿಕಿತ್ಸೆಗೆ ಯಾವುದೇ ಪರಿಣಾಮ ಬೀರದೆ ಯಾವುದೇ ಕಾರಣಕ್ಕೂ ನಾನು ಈ ಅಧ್ಯಯನದಿಂದ ಯಾವುದೇ ಸಮಯದಲ್ಲಿ ಹಿಂದೆ ಸರಿಯಬಹುದು ಎಂದು ನಾನು ಅರ್ಥಮಾಡಿಕೊಂಡಿದ್ದೇನೆ.

ಈ ಅಧ್ಯಯನದಲ್ಲಿ ನನ್ನ ಭಾಗವಹಿಸುವಿಕೆಯಿಂದ ಉಂಟಾಗುವ ವೈಜ್ಞಾನಿಕ ದತ್ತಾಂಶವನ್ನು ವೈಜ್ಞಾನಿಕ ಉದ್ದೇಶಗಳಿಗಾಗಿ ಅವರು ಬಳಸುವುದಕ್ಕಾಗಿ ಪ್ರಧಾನ ತನಿಖಾಧಿಕಾರಿಗಳಿಗೆ ಬಿಡುಗಡೆ ಮಾಡಲು ನಾನು ಸಮ್ಮತಿಸುತ್ತೇನೆ. ನನ್ನ ಗೌಪ್ಯತೆ ಮತ್ತು ಗೌಪ್ಯತೆಯನ್ನು ರಕ್ಷಿಸಲು ಪ್ರಧಾನ ತನಿಖಾಧಿಕಾರಿಗಳು ತಮ್ಮ ಕೈಲಾದಷ್ಟು ಪ್ರಯತ್ನಿಸುತ್ತಾರೆ. ಡಾ. ಶ್ರೀನಿವಾಸನ್, ಪ್ರಾಜೆಕ್ಟ್ ಮ್ಯಾನೇಜರ್, ಮತ್ತು ನನ್ನನ್ನು ಸಂಪರ್ಕಿಸುವ ಸಂತ ಜಾನ್ಸ್ ಅಲ್ಲಿರುವ ಅಧ್ಯಯನ ತಂಡದವರಿಗೆ ಮಾತ್ರ ನನ್ನನ್ನು ಗುರುತಿಸುವ ಮಾಹಿತಿ ಇರುವುತ್ತದೆ. ಇತರ ಅಧ್ಯಯನ ತಂಡ ಸದಸ್ಯರು ಮತ್ತು ಇತರ ವಿಗ್ಯಾನಿಗಳೊಂದಿಗೆ ಹಂಚುವ ಮಾಹಿತಿಯಲ್ಲಿ ನನ್ನನ್ನು ಗುರುತಿಸುವ ಯಾವುದೇ ಮಾಹಿತಿ ಇರುವುದಿಲ್ಲ.

ಈ ಅಧ್ಯಯನದಲ್ಲಿ ನನ್ನ ಭಾಗವಹಿಸುವಿಕೆಯಿಂದ, ನಾನು ನನ್ನ ಯಾವುದೇ ಕಾನೂನು ಹಕ್ಕುಗಳನ್ನು ಬಿಟ್ಟುಕೊಡುತ್ತಿಲ್ಲ ಎಂದು ನಾನು ಅರ್ಥಮಾಡಿಕೊಂಡಿದ್ದೇನೆ. ಯಾವುದೇ ಸಮಸ್ಯೆಯ ಸಂದರ್ಭದಲ್ಲಿ ನಾನು ಈ ಅಧ್ಯಯನದಲ್ಲಿ ಸಂಬಂಧಿಸಿದ ತನಿಖಾಧಿಕಾರಿಗಳನ್ನು ಸಂಪರ್ಕಿಸಬಹುದು ಎಂದು ನಾನು ಅರ್ಥಮಾಡಿಕೊಂಡಿದ್ದೇನೆ.

ದಿನಾಂಕ: _____________ ಹೆಸರು:____________________ ಸಹಿ: _____________________ ಸಾಕ್ಷಿ:____________

**ತನಿಖಾಧಿಕಾರಿಯ ಹೇಳಿಕೆ:**

ಈ ಅಧ್ಯಯನದಲ್ಲಿ ನಿಮ್ಮ ಭಾಗವಹಿಸುವಿಕೆಯು ವೈವಾಹಿಕ ಸಂಬಂಧಗಳಲ್ಲಿನ ಸಂವಹನ ಮತ್ತು ಜಗಳಗಳನ್ನು ಚೆನ್ನಾಗಿ ಅರ್ಥಮಾಡಿಕೊಳ್ಳಲು ನಮಗೆ ಸಹಾಯ ಮಾಡುತ್ತದೆ ಮತ್ತು ಜಗಳಗಳನ್ನು ಕಡಿಮೆ ಮಾಡಿ ಸಂವಹನವನ್ನು ಸುಧಾರಿಸಲು ವಿನ್ಯಾಸಗೊಳಿಸಲಾದ ಕಾರ್ಯಕ್ರಮಗಳನ್ನು ರೂಪಿಸುತ್ತದೆ.

ವಿಷಯಕ್ಕೆ ಮೇಲಿನ ಅಧ್ಯಯನದ ಸ್ವರೂಪ, ಬೇಡಿಕೆಗಳು ಮತ್ತು ನಿರೀಕ್ಷಿತ ಅಪಾಯಗಳನ್ನು ನಾನು ಎಚ್ಚರಿಕೆಯಿಂದ ವಿವರಿಸಿದ್ದೇನೆ

ದಿನಾಂಕ: ________________

ಹೆಸರು:__________________ ಸಹಿ: _____________________

## **St John’s Research Institute, Bangalore**

## **and the University of California, San Francisco**

**Consent Form Wife**

**Title of the Study**: A randomized controlled trial of combined behavioural couples therapy with motivational interviewing to improve communication and reduce conflicts between couples and decrease harmful drinking among spouse in urban primary health centre, South India.

1. **Background and Purpose of the Study:**

This is a research study that examines the effects of an intervention in reducing alcohol and facilitate communication that reduces conflicts between married couples.

Dr. K. Srinivasan from the St. John’s Research Institute, Bangalore and his research team will explain the study to you. This study is funded by the United States National Institutes of Health and is being conducted in collaboration with Drs. Maria Ekstrand and Bibhav Acharya from the University of California, San Francisco.

**2. Researcher Statement:**

We are asking you to be in a research study. The purpose of this consent form is to give you the information you will need to help you decide whether to be in the study or not. Please read the form carefully. You may ask questions about the purpose of the research, what we would ask you to do, the possible risks and benefits, your rights as a volunteer, and anything else about the research or this form that is not clear. When we have answered all your questions, you can decide if you want to be in the study or not. This process is called “informed consent”. We will give you a copy of this form for your records.

1. **How many people will take part in this study?**

About 420 married couples will take part in this study.

1. **What is being done as a part of the Study?**

If you choose to participate in this study, the following will happen:

- You will be interviewed every three months for a year about issues regarding reducing conflicts in your relationship and reducing harmful drinking. Each time, your spouse will be interviewed separately as well.
- Each interview will take approximately an hour and will be held in a private room at a time that is convenient for both you and the interviewer.
- Your husband will be asked to blow into a Breathalyzer device to measure the alcohol level in his blood, once a day for a week right before each interview. The Breathalyzer will be brought to your home by an ASHA and she will pick it back up the day of the interview before accompanying you and your spouse to the interview location. Neither you nor the ASHA will see the test results.
- You will have a 50% chance of being invited to participate in an intervention/program that is designed to help your husband reduce his alcohol use and help you and your husband to improve communication and reduce conflicts. It is decided completely at random (like a lottery) if you will be in the intervention group or in the group that receives a delayed, shorter intervention.
- ***If you are part of the 10-session intervention program***, your husband will first receive four weekly sessions with a study counsellor about his alcohol use; in the fourth session, you will attend with him to understand the role of alcohol in your relationship. After that, you and your husband will participate together in six more weekly sessions of about one hour each that can help improve communication and support between a husband and wife.
- ***If you are part of the delayed intervention program***, referrals for organizational resources and information will be provided to you on topics of relationship conflict and mental health in a session within two months of your joining the study. Your husband will be offered one brief educational program about alcohol use disorder and referrals to de-addiction services to help in reducing his alcohol use. In addition to this, after your last interview at 12 months, you and your husband will be invited to attend four, one-hour long counselling sessions conducted by a study counsellor. In this, your husband will attend the first session alone to discuss his alcohol use, and then you and your husband will both attend three sessions to help understand his alcohol use, and improve communication and support between the husband and the wife.
- Since this program is meant to help improve communication with your spouse, both of you must agree to take part in the study. If either you or your spouse decline, neither of you can take part.

1. **Risks and Discomforts**:

You may experience some difficulty or discomfort talking about your relationship experiences or answering some of our other questions, but you are free to refuse to answer any questions that make you uncomfortable. While the members of the study team will not share anything that you tell us with others and we have put multiple measures in place to keep your information private (see section 9), there is always a small risk of loss of confidentiality. Although we have not found this in our previous programs, it is also possible that discussing tensions in your relationship with your counsellor can lead to more tensions or conflicts while you are at home.

**6. Benefits:**

Your response to the interview question will help us understand factors that influence communication and conflicts between couples. There are no direct benefits to you for participating in this study.

1. **Reimbursement:**

In return for your time, effort and travel expenses, both you and your spouse will receive:

• Rs. 350 for completing the first interview

• Rs. 400 for completing 3-month follow-up

• Rs. 450 for completing 6-month follow-up

• Rs. 500 for completing 9-month follow-up

• Rs. 550 for completing 12-month follow-up

If you are in the intervention group, you and your spouse will receive a travel reimbursement of approximately Rs. 200 each time you attend an intervention session.

In the case one of you has to miss work to attend an intervention session, we will pay you ₹450 as compensation for lost wages. If both you and your spouse have to miss work to attend, we will reschedule to a more convenient time.

1. **Alternative Procedures**:

You are free to choose not to participate in the study, and we assure you that your decision will not have any negative consequences. It will not affect your regular treatment in the PHC. You are also free to stop participating in the study at any moment and for any reason without any negative consequences to you.

1. **Confidentiality:**

We will do our best to make sure that the personal information gathered for this study is kept private and we will never use your name or personal information if the study findings are published or presented in scientific meetings. We may share your research information with other scientists in the future, but we will never share any information that could identify you.

Organizations that may look at and/or copy your de-identified research records for research, quality assurance, and data analysis include:

The US National Institutes of Health (who is funding this study), UCSF’s Institutional Review Board, and St. John’s Institutional Ethical Review Board.

Again, none of these groups will have access to any information that could identify you.

A description of this clinical trial will be available on http://www.ClinicalTrials.gov, as required by U.S. Law and and https://ctri.nic.in/. These websites will not include information that can identify you. At most, the websites will include a summary of the results. You can search these websites at any time.

For any clarifications/questions at any point of time, contact any of the below-mentioned study personnel:

Dr. K. Srinivasan. 9845038195

The Institutional Ethical Committee of St. John’s Medical College Bangalore, which reviews the ethics of all studies with human subjects, has approved this study. If you have any questions or concerns about this study, you should contact:

Dr. Jayanthi Savio,

Member Secretary,

Institutional Ethics Committee

Ground Floor, (Next to Cardinal Gracias Hall/Room No. 117)

St. John’s Medical College, Sarjapur Road, Bangalore – 560034

Contact No: 080-49466346/48) / Email: [sjmc.ierb@stjohns.in](mailto:sjmc.ierb@stjohns.in)

**CONSENT FORM**

Participant's Statement:

I voluntarily accept to participate in the study conducted by the staff of St. John's Research Institute and the University of California, San Francisco. The nature, demands, and hazards involved in this study have been fully explained to me. I understand that I may withdraw from this study at any time for any reason without it affecting my medical treatment in the PHC/hospital.

I consent to the release of scientific data resulting from my participation in this study to the Principal Investigators for use by them for scientific purposes. The Principal Investigators will do their utmost to ensure that my privacy and confidentiality are protected. I understand that only Dr. Srinivasan, the project manager, and St. John’s study staff who need to contact me will have access to any information that could identify me. Data that may be shared with other study team members or other scientists will not include any names.

I also understand that by my participation in this study, I am not waiving any of my legal rights. I understand that in the case of any problem I can contact the concerned investigators in this study or the St John’s Institutional Ethical Committee.

Date: ________________________________________

Name of the Wife: __________________________

Signature: ____________________________________

Witness: ______________________________________

Investigator's Statement:

Your participation in this study will help us better understand communication and conflicts in marital relationships and design programs to reduce conflicts and improve communication.

I have carefully explained the nature, demands, and foreseeable risks of the above study to the subject.

Date:_________________ Signature:__________________________

Name:_____________________________

**ಸೇಂಟ್ ಜಾನ್ಸ್ ಸಂಶೋಧನಾ ಸಂಸ್ಥೆ, ಬೆಂಗಳೂರು**

**ಮತ್ತು ಕ್ಯಾಲಿಫೋರ್ನಿಯಾ ವಿಶ್ವವಿದ್ಯಾಲಯ, ಸ್ಕ್ಯಾನ್ ಫ್ರಾನ್ಸಿಸ್ಕೋ.**

**ಒಪ್ಪಿಗೆ ಪತ್ರ - ಪತ್ನಿ**

ಅಧ್ಯಯನದ ಶೀರ್ಷಿಕೆ: ದಂಪತಿಗಳ ನಡುವಿನ ಸಂವಹನವನ್ನು ಸುಧಾರಿಸಿ, ಜಗಳಗಳನ್ನು ಕಡಿಮೆ ಮಾಡಲು ಮತ್ತು ಸಂಗಾತಿಯ ಹಾನಿಕಾರಕ ಕುಡಿತವನ್ನು ಕಡಿಮೆ ಮಾಡಲು ದಕ್ಷಿಣ ಭಾರತದ ನಗರ ಪ್ರಾಥಮಿಕ ಆರೋಗ್ಯ ಕೇಂದ್ರಗಳಲ್ಲಿ ಪ್ರೇರಕ ಸಂದರ್ಶನ ಹಾಗು ಸಂಯೋಜಿತ ವರ್ತನೆಯ ಜೋಡಿಗಳ ಚಿಕಿತ್ಸೆಯ ಯಾದೃಚ್ಛಿಕ ನಿಯಂತ್ರಿತ ಪ್ರಯೋಗ,

**1. ಅಧ್ಯಯನದ ಹಿನ್ನೆಲೆ ಮತ್ತು ಉದ್ದೇಶ:**

ಈ ಸಂಶೋಧನಾ ಅಧ್ಯಯನವು ಕುಡಿತವನ್ನು ಕಡಿಮೆಗೊಳಿಸಿ, ವಿವಾಹಿತ ದಂಪತಿಗಳ ಮಧ್ಯೆ ಸಂವಹನವನ್ನು ಸುಗಮಗೊಳಿಸಿ ಅವರ ನಡುವಿನ ಜಗಳವನ್ನು ಕಡಿಮೆಗೊಳಿಸುವಲ್ಲಿ ಮಧ್ಯಸ್ಥಿಕೆಯ ಕಾರ್ಯಕ್ರಮದ ಪರಿಣಾಮಗಳನ್ನು ಪರಿಶೀಲಿಸುವುದಾಗಿದೆ.

ಬೆಂಗಳೂರಿನ ಸೇಂಟ್ ಜಾನ್ಸ್ ಸಂಶೋಧನಾ ಸಂಸ್ಥೆಯ ಡಾ. ಕೆ. ಶ್ರೀನಿವಾಸನ್ ಮತ್ತು ಅವರ ಸಂಶೋಧನಾ ತಂಡವು ಈ ಅಧ್ಯಯನದ ಬಗ್ಗೆ ನಿಮಗೆ ವಿವರಿಸುತ್ತಾರೆ. ಈ ಅಧ್ಯಯನ ಯುನೈಟೆಡ್ ಸ್ಟೇಟ್ಸ್ ನ್ಯಾಷನಲ್ ಇನ್‌ಸ್ಟಿಟ್ಯೂಟ್ ಆಫ್ ಹೆಲ್ತ್‌ನಿಂದ ಧನಸಹಾಯ ದೊರೆಯುತ್ತದೆ ಮತ್ತು ಸ್ಯಾನ್ ಫ್ರಾನ್ಸಿಸ್ಕೋದ ಕ್ಯಾಲಿಫೋರ್ನಿಯಾ ವಿಶ್ವವಿದ್ಯಾಲಯದ ಡಾ. ಮಾರಿಯಾ ಎಕ್ಸ್‌ಟ್ರಾಂಡ್ ಮತ್ತು ಬಿಭವ್ ಆಚಾರ್ಯ ಅವರ ಸಹಯೋಗದೊಂದಿಗೆ ನಡೆಸಲಾಗುತ್ತಿದೆ.

**2. ಸಂಶೋಧಕರ ಹೇಳಿಕೆ:**

ಈ ಸಂಶೋಧನಾ ಅಧ್ಯಯನದಲ್ಲಿರಲು ನಾವು ನಿಮ್ಮನ್ನು ಕೇಳುತ್ತಿದ್ದೇವೆ. ಈ ಸಮ್ಮತಿ ಪತ್ರದ ಉದ್ದೇಶವು ಅಧ್ಯಯನದಲ್ಲಿ ಇರಬೇಕೇ ಅಥವಾ ಬೇಡವೇ ಎಂಬುದನ್ನು ನಿರ್ಧರಿಸಲು ನಿಮಗೆ ಸಹಾಯ ಮಾಡುವ ಮಾಹಿತಿಯನ್ನು ನಿಮಗೆ ನೀಡುವುದಾಗಿದೆ. ದಯವಿಟ್ಟು ಈ ಸಮ್ಮತಿ ಪತ್ರವನ್ನು ಎಚ್ಚರಿಕೆಯಿಂದ ಓದಿ. ಸಂಶೋಧನೆಯ ಉದ್ದೇಶ, ನೀವು ಏನು ಮಾಡಬೇಕು, ನಾವು ನಿಮ್ಮನ್ನು ಏನು ಮಾಡಬೇಕೆಂದು ಕೇಳುತ್ತೇವೆ, ಸಂಭವನೀಯ ಅಪಾಯಗಳು ಮತ್ತು ಪ್ರಯೋಜನಗಳು, ಸ್ವಂತ ಇಚ್ಛೆಯಿಂದ ಭಾಗವಹಿಸುತ್ತಿರುವ ನಿಮ್ಮ ಹಕ್ಕುಗಳು ಮತ್ತು ಇನ್ನಿತರೆ ಸಂಶೋಧನ ಅಥವಾ ಸಮ್ಮತಿ ಪತ್ರದಲ್ಲಿ ಸ್ಪಷ್ಟವಾಗಿಲ್ಲದ ಬೇರೆ ಯಾವುದನ್ನಾದರೂ ಕುರಿತು ನೀವು ಪ್ರಶ್ನೆಗಳನ್ನು ಕೇಳಬಹುದು ನಿಮ್ಮ ಎಲ್ಲಾ ಪ್ರಶ್ನೆಗಳಿಗೆ ನಾವು ಉತ್ತರಿಸಿದ ನಂತರ ನೀವು ಅಧ್ಯಯನದಲ್ಲಿ ಇರಬೇಕೆ ಅಥವಾ ಬೇಡವೇ ಎಂಬುದನ್ನು ನಿರ್ಧರಿಸಬಹುದು. ಈ ಪ್ರಕ್ರಿಯೆಯನ್ನು ಮಾಹಿತಿಯುಳ್ಳ ಸಮ್ಮತಿ” ಎಂದು ಕರೆಯಲಾಗುತ್ತದೆ, ದಾಖಲೆಗಳಿಗಾಗಿ ನಾವು ಈ ಪತ್ರದ ನಕಲು ಪ್ರತಿಯನ್ನು ನಿಮಗೆ ನೀಡುತ್ತೇವೆ.

**3. ಈ ಅಧ್ಯಯನದಲ್ಲಿ ಎಷ್ಟು ಜನರು ಭಾಗವಹಿಸುತ್ತಾರೆ?**

ಸುಮಾರು 420 ವಿವಾಹಿತ ಜೋಡಿಗಳು ಈ ಅಧ್ಯಯನದಲ್ಲಿ ಪಾಲ್ಗೊಳ್ಳಲಿದ್ದಾರೆ.

**4. ಅಧ್ಯಯನದ ಭಾಗವಾಗಿ ಏನು ಮಾಡಲಾಗುತ್ತಿದೆ?**

ನೀವು ಈ ಅಧ್ಯಯನದಲ್ಲಿ ಭಾಗವಹಿಸಲು ಆಯ್ಕೆ ಮಾಡಿದರೆ, ಈ ಕೆಳಗಿನವುಗಳು ಸಂಭವಿಸುತ್ತವೆ:

- ನಿಮ್ಮ ಸಂಗಾತಿಯೊಂದಿಗಿನ ನಿಮ್ಮ ಸಂಬಂಧಕ್ಕೆ ಸಂಬಂಧಿಸಿದ ಸಮಸ್ಯೆಗಳ ಬಗ್ಗೆ ಒಂದು ವರ್ಷದವರೆಗೆ ಪ್ರತಿ ಮೂರು ತಿಂಗಳಿಗೊಮ್ಮೆ ನಿಮ್ಮನ್ನು ಸಂದರ್ಶಿಸಲಾಗುತ್ತದೆ ಪ್ರತಿ ಬಾರಿಯೂ ನಿಮ್ಮ ಸಂಗಾತಿಯನ್ನು ಪ್ರತ್ಯೇಕವಾಗಿ ಸಂದರ್ಶನ ಮಾಡಲಾಗುತ್ತದೆ.
- ಪ್ರತಿ ಸಂದರ್ಶನವು ಸರಿಸುಮಾರು ಒಂದು ಗಂಟೆ ತೆಗೆದುಕೊಳ್ಳುತ್ತದೆ ನಿಮಗೆ ಮತ್ತು ಸಂದರ್ಶಕರಿಗೆ ಅನುಕೂಲಕರವಾದ ಸಮಯದಲ್ಲಿ ಖಾಸಗಿ ಕೊಠಡಿಯಲ್ಲಿ ನಡೆಯುತ್ತದೆ.
- ಪ್ರತಿ ಸಂದರ್ಶನಕ್ಕೂ ಒಂದು ವಾರದ ಮೊದಲು ನಿಮ್ಮ ಪತಿಗೆ ದಿನಕ್ಕೆ ಒಮ್ಮೆ, ಅವರ ರಕ್ತದಲ್ಲಿನ ಆಲ್ಕೋಹಾಲ್ ಮಟ್ಟವನ್ನು ಅಳೆಯಲು ಬ್ರೀಥಲೈಜರ್ ಸಾಧನವನ್ನು ಊದಲು ಕೇಳಲಾಗುತ್ತದೆ. ಆಶಾ ಕಾರ್ಯಕರ್ತೆಯರು ಬ್ರೀಥಲೈಜರ್ ಅನ್ನು ನಿಮ್ಮ ಮನೆಗೆ ತರುತ್ತಾರೆ ಮತ್ತು ಅವರು ನಿಮ್ಮನ್ನು ಮತ್ತು ನಿಮ್ಮ ಸಂಗಾತಿಯನ್ನು ಸಂದರ್ಶನ ಸ್ಥಳಕ್ಕೆ ಕರೆದುಕೊಂಡು ಹೋಗುವ ಮೊದಲು ಸಂದರ್ಶನದ ದಿನದಂದು ಆ ಮಷೀನ್ ಅನ್ನು ಹಿಂದೆ ತೆಗೆದುಕೊಳ್ಳುತ್ತಾರೆ. ನೀವು ಅಥವಾ ಆಶಾ ಕಾರ್ಯಕರ್ತೆಯೂ ಈ ಸಾಧನೆಯ ಪರೀಕ್ಷಾ ಫಲಿತಾಂಶಗಳನ್ನು ನೋಡುವುದಿಲ್ಲ.
- ನಿಮಗೆ ಮತ್ತು ನಿಮ್ಮ ಪತಿಗೆ ಅವರ ಕುಡಿತವನ್ನು ಕಡಿಮೆ ಮಾಡಲು ಸಹಾಯ ನೀಡುವ ಮತ್ತು ನಿಮ್ಮಿಬ್ಬರ ನಡುವಿನ ಮಾತುಕಥೆ ಉತ್ತಮಗೊಳ್ಳಿಸುವ ಮತ್ತು ಸಂಘರ್ಷವನ್ನು ಕಡಿಮೆ ಮಾಡಲು ಸಹಾಯ ನೀಡುವ ಒಂದು ಮಧ್ಯಸ್ಥಿಕೆ ಯೋಜನೆಯಲ್ಲಿ ಭಾಗವಹಿಸಲು 50% ಅವಕಾಶ ಇರುವುತ್ತದೆ.
- ನೀವು ಆ ಮಧ್ಯಸ್ಥಿಕೆ ಯೋಜನೆಯಲ್ಲಿ ಭಾಗವಹಿಸಿದ್ದರೆ, ನಮ್ಮ ಅಧ್ಯಯನದ ಕೌನ್ಸೆಲರ್ ನಿಮ್ಮ ಪತಿಗೆ ಅವರ ಕುಡಿತದ ಬಗ್ಗೆ 4 ಸಭೆಗಳು ನೀಡುತ್ತಾರೆ; ನಾಲಕ್ಕನೆ ಸಭೆಯಲ್ಲಿ, ನಿಮ್ಮಿಬ್ಬರ ಸಂಬಂಧದಲ್ಲಿ ಕುಡಿತದ ಪಾತ್ರವನ್ನು ತಿಳಿಯಲು ನೀವೂ ಹಾಜರಾಗುತ್ತೀರಿ. ಅದಾದಮೇಲೆ, ನೀವು ಮತ್ತು ನಿಮ್ಮ ಪತಿ ನಿಮ್ಮಿಬ್ಬರ ನಡುವಿನ ಮಾತುಕಥೆ ಮತ್ತು ಗಂಡ-ಹೆಂಡತಿಯ ನಡುವಿನ ಬೆಂಬಲವನ್ನು ಉತ್ತಮಗೊಳ್ಳಿಸಲು ಇನ್ನೂ 6 ಸಭೆಗಳಲ್ಲಿ ಭಾಗವಿಸುತ್ತೀರಿ.
- ನೀವು ಮತ್ತು ನಿಮ್ಮ ಪತಿ ತಡವಾದ ಮಧ್ಯಸ್ಥಿಕೆ ಯೋಜನೆಯಲ್ಲಿ ಭಾಗವಹಿಸಿದ್ದರೆ, ಅಧ್ಯಯನವನ್ನು ಸೇರಿದ 2 ತಿಂಗಳಗಳ ಒಳಗೆ ನಿಮಗೆ ಸಂಬಂಧದಲ್ಲಿರುವ ಸಂಘರ್ಷ ಮತ್ತು ಮಾನಸಿಕ ಆರೋಗ್ಯದ ವಿಷಯಗಳಲ್ಲಿ ಅಗತ್ಯವಿರುವ ಯಾವುದೇ ಸಹಾಯಕ್ಕಾಗಿ ಸಮುದಾಯ ಸಂಪನ್ಮೂಲಗಳಿಗೆ ನಾವು ನಿಮಗೆ ಮಾಹಿತಿಗಳನ್ನು ಒದಗಿಸುತ್ತೇವೆ. ನಿಮ್ಮ ಪತಿಗೆ ಮದ್ಯಪಾನದ ಸಮಸ್ಯೆಯ ಬಗ್ಗೆ ಮತ್ತು ಡಿ-ಅಡಿಕ್ಷನ್ ಸೇವೆಗಳಿಗೆ ಹೋಗಲು ಮಾಹತಿ ನೀಡಲಾಗುತ್ತದೆ. ಜೊತೆಗೆ, ಈ ಅಧ್ಯಯನದ ಕೊನೆಯ 12 ತಿಂಗಳ ಸಂದರ್ಶನದ ನಂತರ, ನೀವು ಮತ್ತು ನಿಮ್ಮ ಪತಿಯನ್ನು 4, ಒಂದು ಘಂಟೆಯ ಕೌನ್ಸೆಲಿಂಗ್ ಸಭೆಗಳಲ್ಲಿ ಭಾಗವಹಿಸಲು ಆಹ್ವಾನಿಸಲಾಗುತ್ತದೆ. ಇದರಲ್ಲಿ, ನಿಮ್ಮ ಪತಿ ಮೊದಲನೇ ಸಭೆ ಒಬ್ಬರೇ ಹಾಜಿರಾಗಿ ಕೌನ್ಸೆಲರ್ ಜೊತೆ ಅವರ ಕುಡಿತದ ಬಗ್ಗೆ ಚರ್ಚಿಸುತ್ತಾರೆ. ಅದಾದಮೇಲೆ, ಮೂರು ಸಭೆಗಳು ನೀವು ಮತ್ತು ನಿಮ್ಮ ಪತಿ ಇಬ್ಬರೂ ಹಾಜಿರಾಗಿ ಅವರ ಕುಡಿತದ ಬಗ್ಗೆ, ನಿಮ್ಮಿಬ್ಬರ ನಡುವಿನ ಮಾತುಕಥೆ ಮತ್ತು ಬೆಂಬಲವನ್ನು ಉತ್ತಮಗೊಳ್ಳಿಸುವ ಬಗ್ಗೆ ಚರ್ಚಿಸುವ ಅವಕಾಶ ಇರುವುತ್ತದೆ.
- ಈ ಕಾರ್ಯಕ್ರಮವು ನಿಮ್ಮ ಸಂಗಾತಿಯೊಂದಿಗೆ ಸಂವಹನವನ್ನು ಸುಧಾರಿಸಲು ಸಹಾಯ ಮಾಡುವುದರಿಂದ, ನೀವಿಬ್ಬರೂ ಅಧ್ಯಯನದಲ್ಲಿ ಭಾಗವಹಿಸಲು ಒಪ್ಪಿಕೊಳ್ಳಬೇಕು. ನೀವು ಅಥವಾ ನಿಮ್ಮ ಸಂಗಾತಿ ನಿರಾಕರಿಸಿದರೆ, ನೀವಿಬ್ಬರೂ ಭಾಗವಹಿಸಲು ಸಾಧ್ಯವಿಲ್ಲ.

**5. ಅಪಾಯಗಳು ಮತ್ತು ಆರಾಮದಾಯಕವಲ್ಲದ ವಿಷಯಗಳು:**

ನಿಮ್ಮ ಸಂಬಂಧದ ಅನುಭವಗಳ ಬಗ್ಗೆ, ಮಾತನಾಡಲು ಅಥವಾ ನಮ್ಮ ಇತರ ಕೆಲವು ಪ್ರಶ್ನೆಗಳಿಗೆ ಉತ್ತರಿಸಲು ನೀವು ಕೆಲವು ತೊಂದರೆ ಅಥವಾ ಮುಜುಗರವನ್ನು ಅನುಭವಿಸಬಹುದು, ನಿಮಗೆ ಅಹಿತಕರವಾದ ಯಾವುದೇ ಪ್ರಶ್ನೆಗಳಿಗೆ ಉತ್ತರಿಸಲು ನೀವು ಮುಕ್ತವಾಗಿ ನಿರಾಕರಿಸಬಹುದು. ಅಧ್ಯಯನ ತಂಡದ ಸದಸ್ಯರು ನೀವು ನಮಗೆ ಹೇಳುವ ಯಾವುದೇ ವಿಷಯಗಳನ್ನು ಇತರರೊಂದಿಗೆ ಹಂಚಿಕೊಳ್ಳುವುದಿಲ್ಲ. ನಿಮ್ಮ ಮಾಹಿತಿಯನ್ನು ಖಾಸಗಿಯಾಗಿಡಲು ನಾವು ಹಲವಾರು ಕ್ರಮಗಳನ್ನು ತೆಗೆದುಕೊಂಡಿದ್ದೇವೆ (9ನೇ ವಿಯಾಭಗ ನೋಡಿ), ಆದರೂ ಗೌಪ್ಯತೆಯ ಕಳೆದುಕೊಳ್ಳುವ ಅವಕಾಶ ಇರುತ್ತದೆ. ನಿಮ್ಮ ಕೌಂಸೆಲ್ಲೋರ್ ಒಂದಿಗೆ ನಿಮ್ಮ ಸಂಬಂಧದಲ್ಲಿನ ಸಮಸ್ಯೆಗಳನ್ನು ಚರ್ಚಿಸುವುದರಿಂದ ನಿಮ್ಮ ಮನೆ ಅಥವಾ ಸಂಬಂಧದಲ್ಲಿ ಸಮಸ್ಯೆ ಅಥವಾ ಸಂಘರ್ಷಗಳು ಹೆಚ್ಚಾಗುವ ಅಪಾಯವೂ ಇದೆ. ಆದರೆ, ನಮ್ಮ ಹಿಂದಿನ ಯಾವುದೇ ಕಾರ್ಯಕ್ರಮಗಳಲ್ಲಿ ನಾವು ಇದನ್ನು ಕಂಡುಕೊಂಡಿಲ್ಲ.

**6. ಪ್ರಯೋಜನಗಳು:**

ಸಂದರ್ಶನದ ಪ್ರಶ್ನೆಗೆ ನಿಮ್ಮ ಪ್ರತಿಕ್ರಿಯೆಯು ದಂಪತಿಗಳ ನಡುವಿನ ಸಂವಹನ ಮತ್ತು ಜಗಳಗಳ ಮೇಲೆ ಪ್ರಭಾವ ಬೀರುವ ಅಂಶಗಳನ್ನು ಅರ್ಥಮಾಡಿಕೊಳ್ಳಲು ನಮಗೆ ಸಹಾಯ ಮಾಡುತ್ತದೆ. ಈ ಅಧ್ಯಯನದಲ್ಲಿ ಭಾಗವಹಿಸುವುದರಿಂದ ನಿಮಗೆ ಯಾವುದೇ ನೇರ ಪ್ರಯೋಜನಗಳಿಲ್ಲ.

**7. ಮರುಪಾವತಿ:**

ನಿಮ್ಮ ಸಮಯ, ಶ್ರಮ ಮತ್ತು ಪ್ರಯಾಣ ವೆಚ್ಚಗಳಿಗೆ ಪ್ರತಿಯಾಗಿ, ನಿಮಗೆ ಮತ್ತು ನಿಮ್ಮ ಸಂಗಾತಿಗೆ

- ಮೊದಲ ಸಂದರ್ಶನವನ್ನು ಪೂರ್ಣಗೊಳಿಸಲು 350 ರೂ.
- ಮೂರನೆಯ ತಿಂಗಳ ಮರು ಸಂದರ್ಶನ ಪೂರ್ಣಗೊಳಿಸಲು 400 ರೂ.
- ಆರನೆಯ ತಿಂಗಳ ಮರು ಸಂದರ್ಶನ ಪೂರ್ಣಗೊಳಿಸಲು 450 ರೂ.
- ಒಂಬತ್ತನೆಯ ತಿಂಗಳ ಮರು ಸಂದರ್ಶನ ಪೂರ್ಣಗೊಳಿಸಲು 500 ರೂ
- ಹನ್ನೆರಡನೆಯ ತಿಂಗಳ ಸಂದರ್ಶನ ಪೂರ್ಣಗೊಳಿಸಲು 550 ರೂ ನೀಡಲಾಗುವುದು.

ನೀವು ಮಧ್ಯಸ್ಸಿಕ ಕಾರ್ಯಕ್ರಮದಲ್ಲಿದ್ದರೆ, ನೀವು ಮತ್ತು ನಿಮ್ಮ ಸಂಗಾತಿಯು ಪ್ರತಿ ಭೇಟಿಗೆ ಸರಿಸುಮಾರು 200 ರೂ. ಪ್ರಯಾಣ ಮರುಪಾವತಿಯನ್ನು ಸಹ ಪಡೆಯುತ್ತೀರಿ.

ನಿಮ್ಮಲ್ಲಿ ಒಬ್ಬರು ಮಧ್ಯಸ್ಥಿಕೆ ಅಧಿವೇಶನಕ್ಕೆ ಹಾಜರಾಗಲು ಕೆಲಸಕ್ಕೆ ರಜೆ ಹಾಕುವ ಪರಿಸ್ಥಿತಿ ಬಂದರೆ, ಕಳೆದುಹೋದ ವೇತನಕ್ಕೆ ಪರಿಹಾರವಾಗಿ ನಾವು ನಿಮಗೆ ₹450 ಪಾವತಿಸುತ್ತೇವೆ. ನೀವು ಮತ್ತು ನಿಮ್ಮ ಸಂಗಾತಿಯು ಇಬ್ಬರೂ ಹಾಜರಾಗಲು ಕೆಲಸಕ್ಕೆ ರಜೆ ಹಾಕುವ ಪರಿಸ್ಥಿತಿ ಬಂದರೆ, ಮತ್ತೊಂದು ದಿನ ಇನ್ನೂ ಹೆಚ್ಚು ಅನುಕೂಲಕರ ಸಮಯಕ್ಕೆ ಮಧ್ಯಸ್ಥಿಕೆ ಅಧಿವೇಶನವನ್ನು ಮುಂದೂಡುತೇವೆ.

**8. ಪರ್ಯಾಯ ಕಾರ್ಯವಿಧಾನಗಳು:**

ಅಧ್ಯಯನದಲ್ಲಿ ಭಾಗವಹಿಸದಿರಲು ನೀವು ಸ್ವತಂತ್ರರಾಗಿದ್ದೀರಿ ಮತ್ತು ನಿಮ್ಮ ನಿರ್ಧಾರವು ಯಾವುದೇ ಋಣಾತ್ಮಕ ಪರಿಣಾಮಗಳನ್ನು ಹೊಂದಿರುವುದಿಲ್ಲ ಎಂದು ನಾವು ನಿಮಗೆ ಭರವಸೆ ನೀಡುತ್ತೇವೆ. ಇದು ಪಿ.ಹೆಚ್.ಸಿಯಲ್ಲಿ ನಿಮ್ಮ ನಿಯಮಿತ ಚಿಕಿತ್ಸೆಯ ಮೇಲೆ ಪರಿಣಾಮ ಬೀರುವುದಿಲ್ಲ. ನಿಮಗೆ ಯಾವುದೇ ಋಣಾತ್ಮಕ ಪರಿಣಾಮಗಳಿಲ್ಲದೆ ಯಾವುದೇ ಕ್ಷಣದಲ್ಲಿ ಮತ್ತು ಯಾವುದೇ ಕಾರಣಕ್ಕಾಗಿ ಅಧ್ಯಯನದಲ್ಲಿ ಭಾಗವಹಿಸುವುದನ್ನು ನಿಲ್ಲಿಸಲು ನೀವು ಸ್ವತಂತ್ರರಾಗಿದ್ದೀರಿ.

**9. ಗೌಪ್ಯತೆ :**

ಈ ಅಧ್ಯಯನಕ್ಕಾಗಿ ಸಂಗ್ರಹಿಸಲಾದ ವೈಯಕ್ತಿಕ ಮಾಹಿತಿಯನ್ನು ಖಾಸಗಿಯಾಗಿ ಇರಿಸಲಾಗಿದೆಯೆ ಎಂದು ಖಚಿತಪಡಿಸಿಕೊಳ್ಳಲು ನಾವು ನಮ್ಮ ಕೈಲಾದಷ್ಟು ಪ್ರಯತ್ನಿಸುತ್ತೇವೆ ಮತ್ತು ಅಧ್ಯಯನದ ಸಂಶೋಧನೆಗಳನ್ನು ಪ್ರಕಟಿಸಿದರೆ ಅಥವಾ ವೈಜ್ಞಾನಿಕ ಸಭೆಗಳಲ್ಲಿ ಪ್ರಸ್ತುತಪಡಿಸಿದರೆ ನಿಮ್ಮ ಹೆಸರು ಅಥವಾ ವೈಯಕ್ತಿಕ ಮಾಹಿತಿಯನ್ನು ನಾವು ಎಂದಿಗೂ ಬಳಸುವುದಿಲ್ಲ. ನಿಮ್ಮ ಸಂಶೋಧನಾ ಮಾಹಿತಿಯನ್ನು ನಾವು ಭವಿಷ್ಯದಲ್ಲಿ ಇತರ ವಿಜ್ಞಾನಿಗಳೊಂದಿಗೆ ಹಂಚಿಕೊಳ್ಳಬಹುದು, ಆದರೆ ನಿಮ್ಮನ್ನು ಗುರುತಿಸುವ ಯಾವುದೇ ಮಾಹಿತಿಯನ್ನು ನಾವು ಎಂದಿಗೂ ಹಂಚಿಕೊಳ್ಳುವುದಿಲ್ಲ.

ಸಂಶೋಧನೆ, ಗುಣಮಟ್ಟದ ಭರವಸೆ ಮತ್ತುದತ್ತಾಂಶ ವಿಶ್ಲೇಷಣಾ ಸಭೆಗಳಿಗಾಗಿ ನಿಮ್ಮ ಗುರುತಿಸಲಾಗದ ಸಂಶೋಧನಾ ದಾಖಲೆಗಳನ್ನು ನೋಡುವ ಮತ್ತು/ಅಥವಾ ನಕಲಿಸುವ ಸಂಸ್ಥೆಗಳು ಇವುಗಳು.

US ನ್ಯಾಷನಲ್ ಇನ್‌ಸ್ಟಿಟ್ಯೂಟ್ ಆಫ್ ಹೆಲ್ತ್, UCSF ನ ಮಾನವ ಸಂಶೋಧನೆಯ ಸಮಿತಿ, ಮತ್ತು ಸೇಂಟ್ ಜಾನ್ ಇನ್‌ಸ್ಟಿಟ್ಯೂಶನಲ್ ಎಥಿಕಲ್ ರಿವ್ಯೂ ಬೋರ್ಡ್.

ಮತ್ತೊಮ್ಮೆ, ಈ ಗುಂಪುಗಳಲ್ಲಿ ನಿಮ್ಮನ್ನು ಗುರುತಿಸಬಹುದಾದ ಯಾವುದೇ ಮಾಹಿತಿಯನ್ನು ಹಂಚಿಕೊಳ್ಳುವ ಸಾಧ್ಯವಿಲ್ಲ.

ಯಾವುದೇ ಸಮಯದಲ್ಲಿ ಯಾವುದೇ ಸ್ಪಷ್ಟಿಕರಣಗಳು/ಪ್ರಶ್ನೆಗಳಿಗಾಗಿ, ಕಳಗೆ ತಿಳಿಸಲಾದ ಯಾವುದೇ ಅಧ್ಯಯನ ಸಿಬ್ಬಂದಿಯನ್ನು ಸಂಪರ್ಕಿಸಿ:

ಡಾ. ಕೆ.ಶ್ರೀನಿವಾಸನ್ : 9845038195

ಸೇಂಟ್ ಜಾನ್ಸ್ ಮೆಡಿಕಲ್ ಕಾಲೇಜಿನ ಬೆಂಗಳೂರಿನ ಸಂಸ್ಥೆಯ ನೈತಿಕ ಸಮಿತಿಯು, ಮಾನವ ವಿಷಯಗಳೊಂದಿಗೆ ಎಲ್ಲಾ ಅಧ್ಯಯನಗಳ ನೈತಿಕತೆಯನ್ನು ಪರಿಶೀಲಿಸುತ್ತದೆ ಮತ್ತು ಈ ಅಧ್ಯಯನವನ್ನು ಅನುಮೋದಿಸಿದೆ. ಈ ಅಧ್ಯಯನದ ಕುರಿತು ನೀವು ಯಾವುದೇ ಪ್ರಶ್ನೆಗಳನ್ನು ಅಥವಾ ಕಾಳಜಿಗಳನ್ನು ಹೊಂದಿದ್ದರೆ, ನೀವು ಸಂಪರ್ಕಿಸಬೇಕು:

ಡಾ.ಜಯಂತಿ ಸಾವಿಯೋ,

ಸದಸ್ಯ ಕಾರ್ಯದರ್ಶಿ,

ಸಾಂಸ್ಥಿಕ ನೈತಿಕ ಸಮಿತಿ

ನೆಲ ಮಹಡಿ, (ಕಾರ್ಡಿನಲ್ ಗ್ರೇಸಿಯಾಸ್ ಹಾಲ್/ಕೊಠಡಿ ಸಂಖ್ಯೆ 117 ರ ಪಕ್ಕದಲ್ಲಿ)

ಸೇಂಟ್ ಜಾನ್ಸ್ ವೈದ್ಯಕೀಯ ಕಾಲೇಜು, ಸರ್ಜಾಪುರ ರಸ್ತೆ, ಬೆಂಗಳೂರು 560034

ಸಂಪರ್ಕ ಸಂಖ್ಯೆ : 080-49466346/48) / ಇಮೇಲ್ : sjmc.ierb@stjohns.in

**ಒಪ್ಪಿಗೆ ಪತ್ರ**

**ಭಾಗವಹಿಸುವವರ ಹೇಳಿಕೆ:**

ಸೇಂಟ್ ಜಾನ್ಸ್‌ ಸಂಶೋಧನಾ ಸಂಸ್ಥೆ ಮತ್ತು ಸ್ಕ್ಯಾನ್ ಫ್ರಾನ್ಸಿಸ್ಕೋದ ಕ್ಯಾಲಿಫೋರ್ನಿಯಾ ವಿಶ್ವವಿದ್ಯಾಲಯದ ಸಿಬ್ಬಂದಿ ನಡೆಸಿದ ಅಧ್ಯಯನದಲ್ಲಿ ಭಾಗವಹಿಸಲು ನಾನು ಸ್ವಯಂಪ್ರೇರಣೆಯಿಂದ ಒಪ್ಪಿಕೊಳ್ಳುತ್ತೇನೆ. ಈ ಅಧ್ಯಯನದಲ್ಲಿ ಒಳಗೊಂಡಿರುವ ಸ್ವಭಾವ, ಬೇಡಿಕೆಗಳು ಮತ್ತು ಅಪಾಯಗಳನ್ನು ನನಗೆ ಸಂಪೂರ್ಣವಾಗಿ ವಿವರಿಸಲಾಗಿದೆ. ಪಿ.ಹೆಚ್.ಸಿ/ಆಸ್ಪತ್ರೆಯಲ್ಲಿನ ನನ್ನ ವೈದ್ಯಕೀಯ ಚಿಕಿತ್ಸೆಗೆ ಯಾವುದೇ ಪರಿಣಾಮ ಬೀರದೆ ಯಾವುದೇ ಕಾರಣಕ್ಕೂ ನಾನು ಈ ಅಧ್ಯಯನದಿಂದ ಯಾವುದೇ ಸಮಯದಲ್ಲಿ ಹಿಂದೆ ಸರಿಯಬಹುದು ಎಂದು ನಾನು ಅರ್ಥಮಾಡಿಕೊಂಡಿದ್ದೇನೆ.

ಈ ಅಧ್ಯಯನದಲ್ಲಿ ನನ್ನ ಭಾಗವಹಿಸುವಿಕೆಯಿಂದ ಉಂಟಾಗುವ ವೈಜ್ಞಾನಿಕ ದತ್ತಾಂಶವನ್ನು ವೈಜ್ಞಾನಿಕ ಉದ್ದೇಶಗಳಿಗಾಗಿ ಅವರು ಬಳಸುವುದಕ್ಕಾಗಿ ಪ್ರಧಾನ ತನಿಖಾಧಿಕಾರಿಗಳಿಗೆ ಬಿಡುಗಡೆ ಮಾಡಲು ನಾನು ಸಮ್ಮತಿಸುತ್ತೇನೆ. ನನ್ನ ಗೌಪ್ಯತೆ ಮತ್ತು ಗೌಪ್ಯತೆಯನ್ನು ರಕ್ಷಿಸಲು ಪ್ರಧಾನ ತನಿಖಾಧಿಕಾರಿಗಳು ತಮ್ಮ ಕೈಲಾದಷ್ಟು ಪ್ರಯತ್ನಿಸುತ್ತಾರೆ. ಡಾ. ಶ್ರೀನಿವಾಸನ್, ಪ್ರಾಜೆಕ್ಟ್ ಮ್ಯಾನೇಜರ್, ಮತ್ತು ನನ್ನನ್ನು ಸಂಪರ್ಕಿಸುವ ಸಂತ ಜಾನ್ಸ್ ಅಲ್ಲಿರುವ ಅಧ್ಯಯನ ತಂಡದವರಿಗೆ ಮಾತ್ರ ನನ್ನನ್ನು ಗುರುತಿಸುವ ಮಾಹಿತಿ ಇರುವುತ್ತದೆ. ಇತರ ಅಧ್ಯಯನ ತಂಡ ಸದಸ್ಯರು ಮತ್ತು ಇತರ ವಿಗ್ಯಾನಿಗಳೊಂದಿಗೆ ಹಂಚುವ ಮಾಹಿತಿಯಲ್ಲಿ ನನ್ನನ್ನು ಗುರುತಿಸುವ ಯಾವುದೇ ಮಾಹಿತಿ ಇರುವುದಿಲ್ಲ.

ಈ ಅಧ್ಯಯನದಲ್ಲಿ ನನ್ನ ಭಾಗವಹಿಸುವಿಕೆಯಿಂದ, ನಾನು ನನ್ನ ಯಾವುದೇ ಕಾನೂನು ಹಕ್ಕುಗಳನ್ನು ಬಿಟ್ಟುಕೊಡುತ್ತಿಲ್ಲ ಎಂದು ನಾನು ಅರ್ಥಮಾಡಿಕೊಂಡಿದ್ದೇನೆ. ಯಾವುದೇ ಸಮಸ್ಯೆಯ ಸಂದರ್ಭದಲ್ಲಿ ನಾನು ಈ ಅಧ್ಯಯನದಲ್ಲಿ ಸಂಬಂಧಿಸಿದ ತನಿಖಾಧಿಕಾರಿಗಳನ್ನು ಸಂಪರ್ಕಿಸಬಹುದು ಎಂದು ನಾನು ಅರ್ಥಮಾಡಿಕೊಂಡಿದ್ದೇನೆ.

ದಿನಾಂಕ: _____________ ಹೆಸರು:____________________ ಸಹಿ: _____________________ ಸಾಕ್ಷಿ:____________

**ತನಿಖಾಧಿಕಾರಿಯ ಹೇಳಿಕೆ:**

ಈ ಅಧ್ಯಯನದಲ್ಲಿ ನಿಮ್ಮ ಭಾಗವಹಿಸುವಿಕೆಯು ವೈವಾಹಿಕ ಸಂಬಂಧಗಳಲ್ಲಿನ ಸಂವಹನ ಮತ್ತು ಜಗಳಗಳನ್ನು ಚೆನ್ನಾಗಿ ಅರ್ಥಮಾಡಿಕೊಳ್ಳಲು ನಮಗೆ ಸಹಾಯ ಮಾಡುತ್ತದೆ ಮತ್ತು ಜಗಳಗಳನ್ನು ಕಡಿಮೆ ಮಾಡಿ ಸಂವಹನವನ್ನು ಸುಧಾರಿಸಲು ವಿನ್ಯಾಸಗೊಳಿಸಲಾದ ಕಾರ್ಯಕ್ರಮಗಳನ್ನು ರೂಪಿಸುತ್ತದೆ.

ವಿಷಯಕ್ಕೆ ಮೇಲಿನ ಅಧ್ಯಯನದ ಸ್ವರೂಪ, ಬೇಡಿಕೆಗಳು ಮತ್ತು ನಿರೀಕ್ಷಿತ ಅಪಾಯಗಳನ್ನು ನಾನು ಎಚ್ಚರಿಕೆಯಿಂದ ವಿವರಿಸಿದ್ದೇನೆ

ದಿನಾಂಕ: ________________ ಹೆಸರು:___________________

ಸಹಿ: _____________________

**Appendix N**

**Breathalyzer Usage Script**

We are going to give you a device that you need to use from today for the next 7 days. It is called a breathalyzer, which is used to measure the alcohol content in the breath. I will show you how to use it.

[Team members will show a demonstration of the breathalyzer by explaining the following].

A breathalyzer is an electronic device that needs to be charged every two days. There is an indication [show the charge indicator] and we give you a charger that you can use to charge the device.

To record the exhaled air, you will need to:

1. Make sure the device is turned on before use. Press [show the Switch On/Off button] for 2 seconds until the screen lights up. Then attach the straw to the connector on the device, making sure the straw is connected correctly. You will be given straws. Please use a new straw each time you use the device.
2. Once turned on, wait until the screen shows 'blow' in green. The device gives a long beep, during which you have to blow air out of your mouth after taking a deep breath. You can stop blowing air after the beep stops. You will be able to find the alcohol measurement on the screen for some time.
3. Please request a family member to take a picture of you while you are using the device. Please send this picture to our study mobile number via WhatsApp or MMS.
4. For the next seven days you need to use it once a day and if possible, try to maintain a specific time in the day to record the blow.
5. After using the device, press the "Switch On / Off" button for 3 counts until the light on the screen turns off.

Please let me know if you understood what I have explained to you. Do you want me to explain it again? Would you like me to repeat what I explained to you?... Do you have any questions?

After 7 days of use the ASHA worker will collect the device from you on the 8th day. If they can't collect it, they'll let us know. We will then contact you and schedule a date to collect the breathalyzer from you, along with the charger, ziplock bag with used straws, and device mouthpiece.

Please make sure you keep the device safe and away from children at home.

Please let me know if you can understand what I have explained to you. Do you want me to explain it again?

Thank you

**ಬ್ರೀಥಲೈಸರ್ ಸ್ಕ್ರಿಪ್ಟ್**

ಇಂದಿನಿಂದ ಮುಂದಿನ 7 ದಿನಗಳವರೆಗೆ ನೀವು ಬಳಸಬೇಕಾದ ಸಾಧನವನ್ನು ನಾವು ನಿಮಗೆ ನೀಡಲಿದ್ದೇವೆ. ಇದನ್ನು ಬ್ರೀಥಲೈಸರ್ ಎಂದು ಕರೆಯಲಾಗುತ್ತದೆ, ಇದನ್ನು ಉಸಿರಾಟದಲ್ಲಿರುವ ಆಲ್ಕೋಹಾಲ್ ಅಂಶವನ್ನು ಅಳೆಯಲು ಬಳಸಲಾಗುತ್ತದೆ. ಇದನ್ನು ಹೇಗೆ ಬಳಸಬೇಕೆಂದು ನಾನು ನಿಮಗೆ ತೋರಿಸುತ್ತೇನೆ.

[Team members will show a demonstration of the breathalyzer by explaining the following].

ಬ್ರೀಥ್‌ಲೈಜರ್ ಎನ್ನುವುದು ಎಲೆಕ್ಟ್ರಾನಿಕ್ ಸಾಧನವಾಗಿದ್ದು, ಇದನ್ನು ಎರಡು ದಿನಕ್ಕೊಮ್ಮೆ ಚಾರ್ಜ್ ಮಾಡಬೇಕಾಗುತ್ತದೆ. ಇಲ್ಲಿ ಒಂದು ಸೂಚನೆಯಿದೆ *[*show the charge indicator] ಮತ್ತು ಸಾಧನವನ್ನು ಚಾರ್ಜ್ ಮಾಡಲು ನೀವು ಬಳಸಬಹುದಾದ ಚಾರ್ಜರ್ ಅನ್ನು ನಾವು ನಿಮಗೆ ನೀಡುತ್ತೇವೆ.

ಉಸಿರ ಗಾಳಿವನ್ನು ರೆಕಾರ್ಡ್ ಮಾಡಲು ನೀವು ಇದನ್ನು ಮಾಡಬೇಕಾಗಿದೆ:

1. ಬಳಸುವ ಮೊದಲು ಸಾಧನವನ್ನು ಆನ್ ಮಾಡಲಾಗಿದೆಯೇ ಎಂದು ಖಚಿತಪಡಿಸಿಕೊಳ್ಳಿ. ಪರದೆಯಲ್ಲಿ ಬೆಳಕು ಬರೋವರೆಗೂ 2 ಸೆಕೆಂಡುಗಳ ಕಾಲ ಈ ಬಟ್ಟನ್ ಅನ್ನು [show the Switch On/Off button]ಒತ್ತಿರಿ. ನಂತರ ಸಾಧನದಲ್ಲಿನ ಕನೆಕ್ಟರ್‌ಗೆ ಸ್ಟ್ರಾವನ್ನು ಲಗತ್ತಿಸಿ, ಸ್ಟ್ರಾ ಸರಿಯಾದ ರೀತಿಯಲ್ಲಿ ಸಂಪರ್ಕಗೊಂಡಿದೆ ಎಂದು ಖಚಿತಪಡಿಸಿಕೊಳ್ಳಿ. ನಿಮಗೆ ಸ್ಟ್ರಾಗಳನ್ನು ನೀಡಲಾಗುವುದು. ನೀವು ಪ್ರತಿ ಬಾರಿ ಸಾಧನವನ್ನು ಬಳಸಿದಾಗ ದಯವಿಟ್ಟು ಹೊಸ ಸ್ಟ್ರಾವನ್ನು ಬಳಸಿ.
2. ಒಮ್ಮೆ ಆನ್ ಮಾಡಿ, ಪರದೆಯ ಹಸಿರು ಬಣ್ಣದಲ್ಲಿ 'ಬ್ಲೋ' ತೋರಿಸುವವರೆಗೂ ಕಾಯಿರಿ. ಸಾಧನವು ದೀರ್ಘವಾದ ಬೀಪ್ ಅನ್ನು ನೀಡುತ್ತದೆ, ಅದರ ಸಮಯದಲ್ಲಿ ನೀವು ಆಳವಾದ ಉಸಿರನ್ನು ತೆಗೆದುಕೊಂಡ ನಂತರ ನಿಮ್ಮ ಬಾಯಿಯಿಂದ ಗಾಳಿಯನ್ನು ಊದಬೇಕಾಗುತ್ತದೆ. ಬೀಪ್ ನಿಂತ ನಂತರ ನೀವು ಗಾಳಿ ಊದುವುದನ್ನು ನಿಲ್ಲಿಸಬಹುದು. ನೀವು ಸ್ವಲ್ಪ ಸಮಯದವರೆಗೆ ಪರದೆಯಲ್ಲಿ ಆಲ್ಕೋಹಾಲ್ ಅಳತೆಯನ್ನು ಕಂಡುಹಿಡಿಯಲು ಸಾಧ್ಯವಾಗುತ್ತದೆ.
3. ನೀವು ಸಾಧನಕ್ಕೆ ಬಳಸುತ್ತಿರುವಾಗ ನಿಮ್ಮ ಚಿತ್ರವನ್ನು ತೆಗೆದುಕೊಳ್ಳಲು ದಯವಿಟ್ಟು ಕುಟುಂಬದ ಸದಸ್ಯರನ್ನು ವಿನಂತಿಸಿ. ದಯವಿಟ್ಟು ಈ ಚಿತ್ರವನ್ನು ನಮ್ಮ ಅಧ್ಯಯನದ ಮೊಬೈಲ್ ಸಂಖ್ಯೆಗೆ WhatsApp ಮೂಲಕ ಅಥವಾ MMS ಮೂಲಕ ಕಳುಹಿಸಿ.
4. ಮುಂದಿನ ಏಳು ದಿನಗಳವರೆಗೆ ನೀವು ದಿನಕ್ಕೆ ಒಮ್ಮೆ ಬಳಸಬೇಕಾಗುತ್ತದೆ ಮತ್ತು ಸಾಧ್ಯವಾದರೆ, ಬ್ಲೋ ಅನ್ನು ರೆಕಾರ್ಡ್ ಮಾಡಲು ದಿನದಲ್ಲಿ ನಿರ್ದಿಷ್ಟ ಸಮಯವನ್ನು ನಿರ್ವಹಿಸಲು ಪ್ರಯತ್ನಿಸಿ.
5. ಸಾಧನವನ್ನು ಬಳಸಿದ ನಂತರ, ಪರದೆಯ ಮೇಲೆ ಬೆಳಕು ಆಫ್ ಆಗುವವರೆಗೆ 3 ಎಣಿಕೆಗಳಿಗಾಗಿ "ಸ್ವಿಚ್ ಆನ್ / ಆಫ್" ಬಟನ್ ಅನ್ನು ಒತ್ತಿರಿ.

ನಾನು ನಿಮಗೆ ವಿವರಿಸಿದ್ದನ್ನು ನೀವು ಅರ್ಥಮಾಡಿಕೊಳ್ಳಲು ಸಾಧ್ಯವಾದರೆ ದಯವಿಟ್ಟು ನನಗೆ ತಿಳಿಸಿ. ನಾನು ಅದನ್ನು ಮತ್ತೊಮ್ಮೆ ವಿವರಿಸಲು ನೀವು ಬಯಸುತ್ತೀರಾ? ನಾನು ನಿಮಗೆ ವಿವರಿಸಿದ್ದನ್ನು ಪುನರಾವರ್ತಿಸಲು ನೀವು ಬಯಸುವಿರಾ?... ನಿಮಗೆ ಯಾವುದೇ ಪ್ರಶ್ನೆಗಳಿವೆಯೇ?

7 ದಿನಗಳ ಬಳಕೆಯ ನಂತರ ಆಶಾ ಕಾರ್ಯಕರ್ತೆ 8ನೇ ದಿನದಂದು ಅದನ್ನು ನಿಮ್ಮಿಂದ ಸಂಗ್ರಹಿಸುತ್ತಾರೆ. ಅವರು ಅದನ್ನು ಸಂಗ್ರಹಿಸಲು ಸಾಧ್ಯವಾಗದಿದ್ದರೆ, ಅವರು ನಮಗೆ ತಿಳಿಸುತ್ತಾರೆ. ನಂತರ ನಾವು ನಿಮ್ಮನ್ನು ಸಂಪರ್ಕಿಸುತ್ತೇವೆ ಮತ್ತು ಚಾರ್ಜರ್, ಬಳಸಿದ ಸ್ಟ್ರಾಗಳೊಂದಿಗೆ ಜಿಪ್‌ಲಾಕ್ ಬ್ಯಾಗ್ ಮತ್ತು ಸಾಧನದ ಮೌತ್‌ಪೀಸ್ ಜೊತೆಗೆ ನಿಮ್ಮಿಂದ ಬ್ರೀತ್‌ಲೈಜರ್ ಅನ್ನು ಸಂಗ್ರಹಿಸಲು ದಿನಾಂಕವನ್ನು ನಿಗದಿಪಡಿಸುತ್ತೇವೆ.

ದಯವಿಟ್ಟು ನೀವು ಸಾಧನವನ್ನು ಸುರಕ್ಷಿತವಾಗಿರಿಸಿದ್ದೀರಿ ಮತ್ತು ಮನೆಯಲ್ಲಿ ಮಕ್ಕಳಿಂದ ದೂರವಿರಿ ಎಂದು ಖಚಿತಪಡಿಸಿಕೊಳ್ಳಿ.

ನಾನು ನಿಮಗೆ ವಿವರಿಸಿದ್ದನ್ನು ನೀವು ಅರ್ಥಮಾಡಿಕೊಳ್ಳಲು ಸಾಧ್ಯವಾದರೆ ದಯವಿಟ್ಟು ನನಗೆ ತಿಳಿಸಿ. ನಾನು ಅದನ್ನು ಮತ್ತೊಮ್ಮೆ ವಿವರಿಸಲು ನೀವು ಬಯಸುವಿರಾ?

ಧನ್ಯವಾದಗಳು.

**Appendix O**

**Assessment team tracking form**


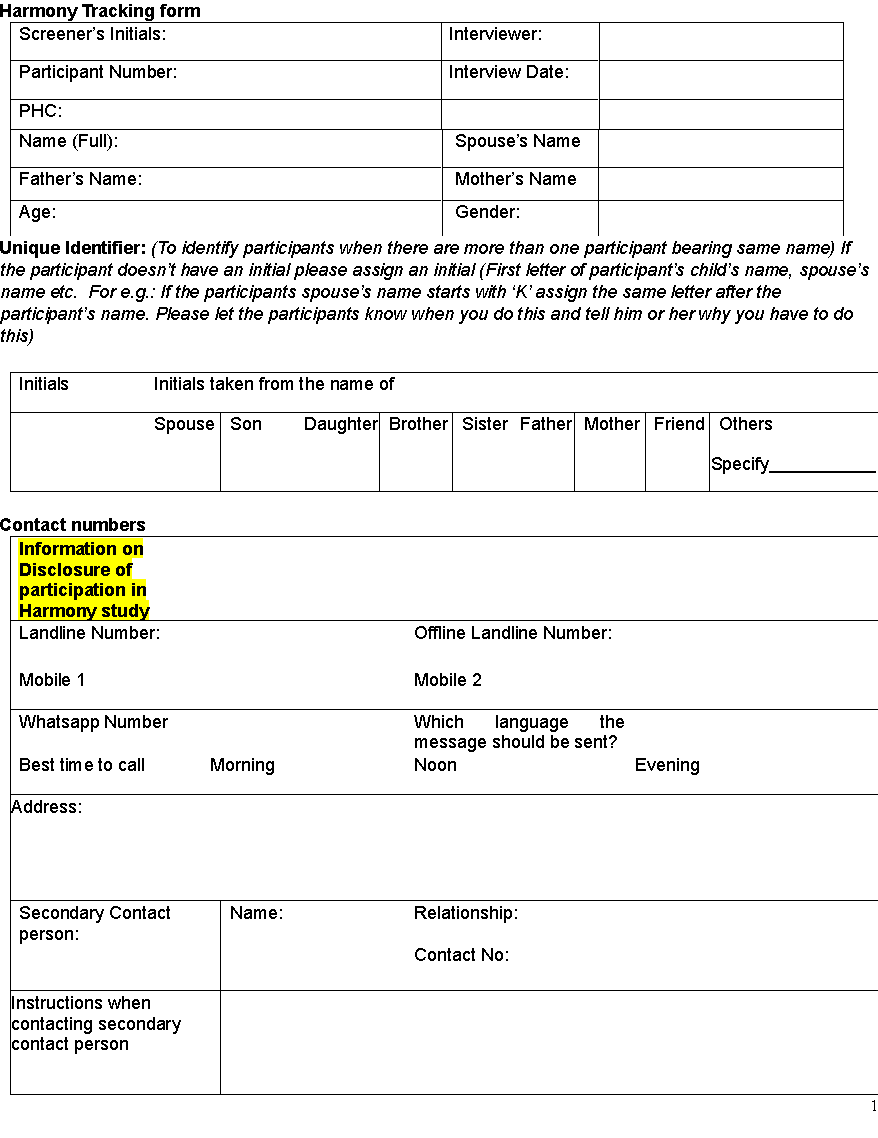


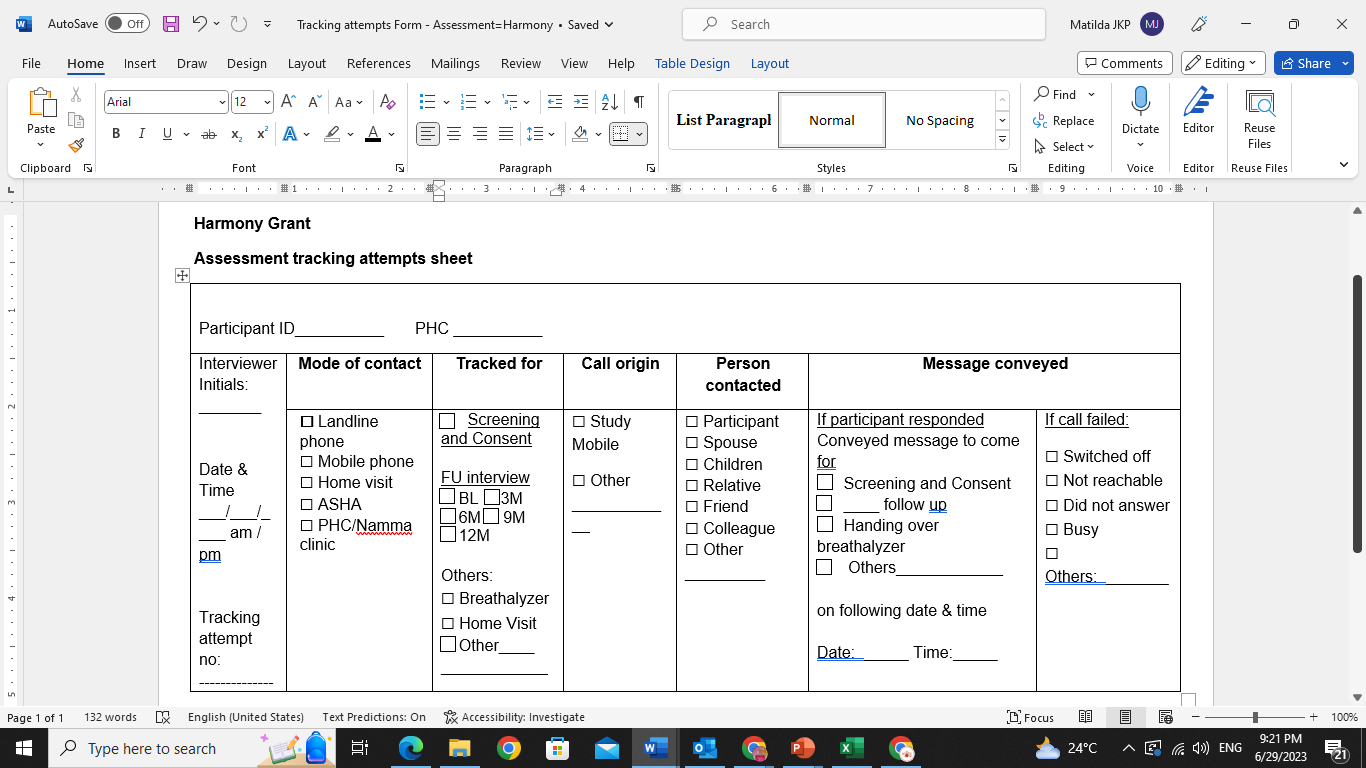


**Appendix P**

**Intervention Master Sheet**


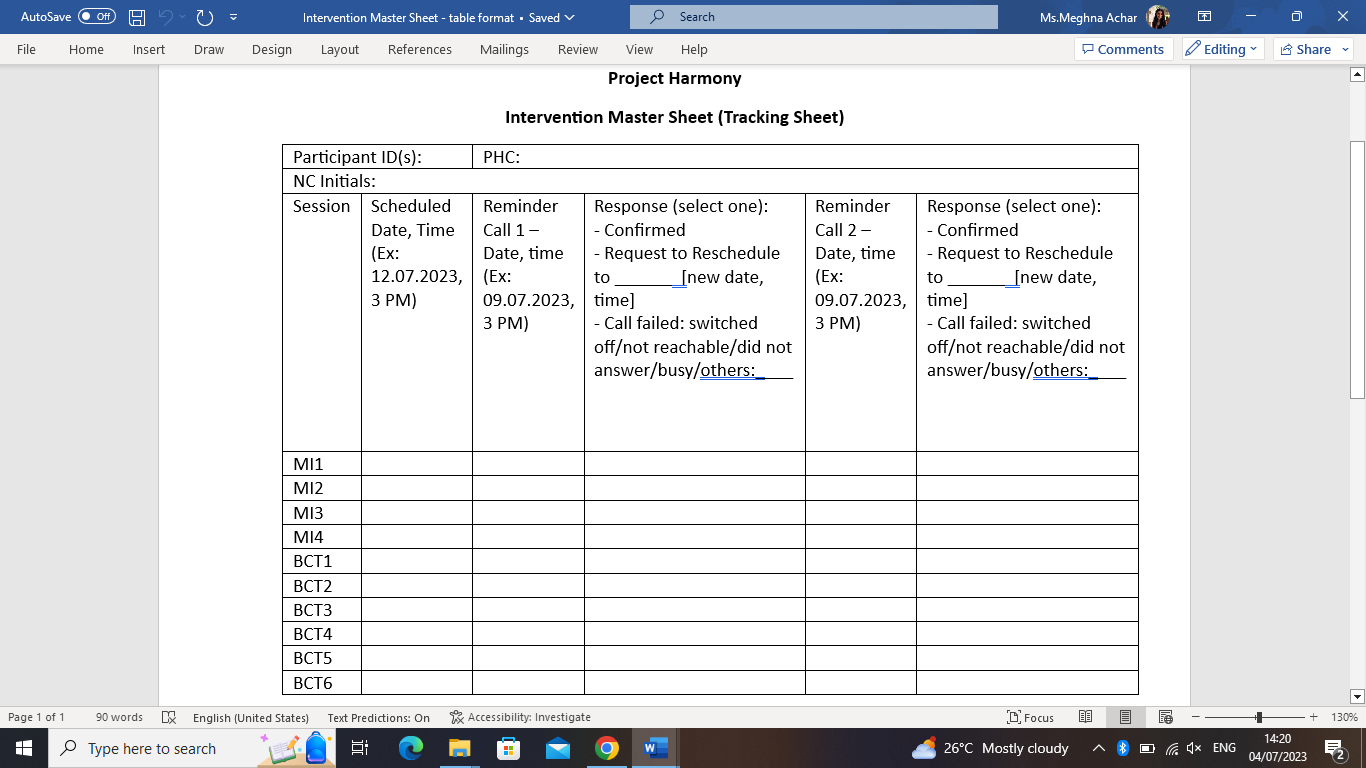


**Appendix Q**

**Control Arm Tracking Sheet**


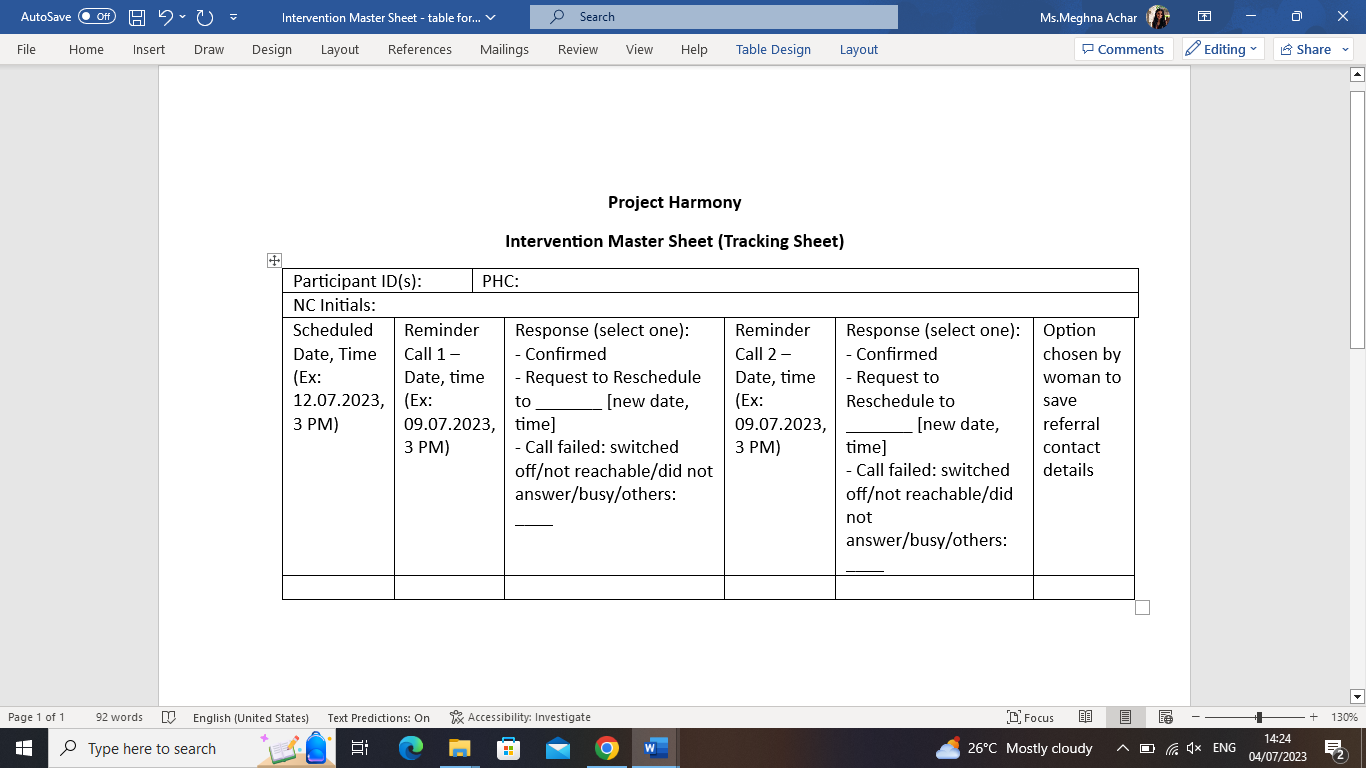


**Appendix R**

**Assessment team certification form**

**Evaluation Form – Wife**

**Date:**

**Name of the Interviewer:**

**Observer’s name:**

**Instructions:**

*The interviewer will be asked to conduct the following sections.*

*Observers will indicate whether they think the Interviewer has adequately demonstrated proficiency by marking 1 – Completed. If the observer thinks the Interviewer did not demonstrate proficiency in conducting a given section and needs additional training, they may mark 0 and write down points for additional training.*

*Observers may note down comments if any, for discussion with other observers after evaluation.*

| **1. Introduction of the study to the participant** | The interviewer greets and explains the study to the participant in brief. This includes, introducing self, place of work, brief about the study and assisting the participant understand their visit schedules The Interviewer requests the participant to explain back to them what has been explained and checks with them if they are interested in participating and if they can go ahead with the next study procedures to check eligibility. | 1 – Completed  0 – Needs additional training in the following aspects  1. _______________________  2. _______________________  3. _______________________  4. _______________________  5. _______________________ |
| --- | --- | --- |
| **2. Administration of the Screening Form** | The interviewer uses the Screening form to assess if the participant is eligible to participate in the study.  Before asking sensitive questions on IFVS, the interviewer alerts the participants that he / she is going to ask a few sensitive questions.  After completing the screening, the interviewer totals the scores and determines eligibility.  If eligible the interviewer communicates to the participant that she is eligible to participate in the study and checks with her in what ways she would like us to contact her husband to help him visit the PHC/Namma Clinic to meet us.  If the participant is not eligible the interviewer will communicate this and will tell the participant that he/she will be taking her through referrals for through the Women’s Resources List. | 1 – Completed  0 – Needs additional training in the following aspects  1. _______________________  2. _______________________  3. _______________________  4. _______________________  5. _______________________ |
| **4. Administration of the consent form** | The Interviewer reads the consent form to the participant, explains each paragraph and requests the participant to explain back to them what they understood.  If the participant had difficulty in understanding or was not able to reply accurately the interviewer will explain again. | 1 – Completed  0 – Needs additional training in the following aspects  1. _______________________  2. _______________________  3. _______________________  4. _______________________  5. _______________________ |
| **5. Conducting Baseline Interview** | The Interviewer will conduct the baseline interview after the consent is signed.  Before conducting the interview, the interviewer will let the participant know how much time approximately the interview is going to take. If the participant is willing to stay for the time frame communicated, the interviewer will conduct the interview. If not, the interviewer will try to fix another date and reschedule the interview.  The interviewer will go through each section in REDCap exactly according to order in the REDCap application.  The observers will rate the interviewers on the following components:   1. Beginning of the interview 2. Body posture 3. Eye contact 4. Frequency of interruptions 5. Use of silence 6. Use of facilitation / visuals 7. Ability to keep the discussion relevant. 8. Non-Judgmental 9. Empathy 10. Warmth 11. Questioning style 12. Ending of the interview | 1 – Completed  0 – Needs additional training in the following aspects  1. _______________________  2. _______________________  3. _______________________  4. _______________________  5. _______________________ |

**Evaluation Form – Husband**

**Date:**

**Name of the Interviewer:**

**Observer’s name:**

**Instructions:**

*The interviewer will be asked to conduct the following sections.*

*Observers will indicate whether they think the Interviewer has adequately demonstrated proficiency by marking 1 – Completed. If the observer thinks the Interviewer did not demonstrate proficiency in conducting a given section and needs additional training, they may mark 0 – Not completed.*

*Observers may note down comments if any, for discussion with other observers after evaluation.*

| **1. Introduction of the study to the participant** | The interviewer greets and explains the study to the participant in brief. This includes, introducing self, place of work, brief about the study and assisting the participant understand their visit schedules The Interviewer requests the participant to explain back to them what has been explained and checks with them if they are interested in participating and if they can go ahead with the next study procedures to check eligibility. | 1 – Completed  0 – Needs additional training in the following aspects  1. _______________________  2. _______________________  3. _______________________  4. _______________________  5. _______________________ |
| --- | --- | --- |
| **2. Administration of the Screening Form** | The interviewer uses the Screening form to assess if the participant is eligible to participate in the study.  Before asking sensitive questions on Alcohol, the interviewer alerts the participants that he / she is going to ask a few sensitive questions.  After completing the screening, the interviewer totals the scores and determines eligibility.  If eligible the interviewer communicates to the participant that he is eligible to participate in the study and that he / she be explaining the consent form to them.  If the participant is not eligible the interviewer will communicate this and will tell the participant that he/she will be taking him through a Brief Education Module. | 1 – Completed  0 – Needs additional training in the following aspects  1. _______________________  2. _______________________  3. _______________________  4. _______________________  5. _______________________ |
| **3. Administration of the consent form** | The Interviewer reads the consent form to the participant, explains each paragraph and requests the participant to explain back to them what they understood.  If the participant had difficulty in understanding or was not able to reply accurately the interviewer will explain again. | 1 – Completed  0 – Needs additional training in the following aspects  1. _______________________  2. _______________________  3. _______________________  4. _______________________  5. _______________________ |
| **4. Conducting Baseline Interview** | The Interviewer will conduct the baseline interview after the consent is signed.  Before conducting the interview, the interviewer will let the participant know how much time approximately the interview is going to take. If the participant is willing to stay for the time frame communicated, the interviewer will conduct the interview. If not, the interviewer will try to fix another date and reschedule the interview.  The interviewer will go through each section in REDCap exactly according to order in the REDCap application.  The observers will rate the interviewers on the following components:   1. Beginning of the interview 2. Body posture 3. Eye contact 4. Frequency of interruptions 5. Use of silence 6. Use of visuals 7. Ability to keep the discussion relevant. 8. Non-judgemental 9. Empathy 10. Warmth 11. Questioning style   Ending of the interview | 1 – Completed  0 – Needs additional training in the following aspects  1. _______________________  2. _______________________  3. _______________________  4. _______________________  5. _______________________ |

**Appendix S**

**Death Report Form - IEC**

To

The Member Secretary

Institutional Ethics Committee

St. John’s Medical College, Bangalore

Dear Doctor,

**Subject: Submission of reporting of onsite serious adverse events (SAE)**

| **1. IEC Study Ref. No.** |  | |
| --- | --- | --- |
| **2. Study / Protocol No.**  **( For drug/ device trials/ any other)** |  | |
| **3. Title of project** |  | |
| **4. Principal Investigator** | | |
| Name |  | |
| Designation and Department |  | |
| Mobile No & email ID |  | |
| **5. Suspected Adverse Reaction**  **(diagnosis)** |  | |
| **6. Report date:** |  | |
| **7. Date of onset of SAE:** |  | |
| **8. Report type** | | |
| a. Initial |  | |
| b. Follow up -------------  If Follow-up report, state date of Initial report & SAE No |  | |
| c. Final : |  | |
| **9. Patient Information** | | |
| a. Patient Initial and Case No./ Subject ID. |  | |
| b. Age |  | |
| c. Gender |  | |
| d. Height |  | |
| e. Weight |  | |
| **10. Information related to no. of recruitment/prior SAE and death :**     |  | Total number of  Recruitment at | Total number of SAE (prior) occurred at | Number of similar SAEs (prior) occurred for same study at | Total number of death at | | --- | --- | --- | --- | --- | | This site |  |  |  |  | | Other site (s) |  |  |  |  | | | |
| **11. Tick which eve is applicable for serious adverse event**  A] Expected event [ ] Unexpected event [ ]  B] Hospitalization [ ] Increased hospital stay [ ] Death [ ] Others [ ]    In case of Death, state probable cause of death ………………………………………………  (If other, please specify: ………………………………………………………………………….  C] No permanent significant functional/ cosmetic impairment [ ]  Permanent significant functional/ cosmetic impairment [ ]  Not applicable [ ] | | |
| **12. If there was a research related injury/hospitalization, the cost of treatment / hospitalization was borne by :**  Patient [ ] Institute [ ] Sponsor/CRO [ ] | | |
| **13. Suspect drug information** | | |
| a. Suspect drug (include generic name) device/intervention | |  |
| b. Indication(s) for which suspect drug was prescribed or tested | |  |
| c. Daily dose and regimen | |  |
| d. Route(s) of administration | |  |
| e. Dosage Form and Strength | |  |
| f. Therapy dates (start and stopped date) | |  |
| **14. Did the reaction decline after stopping the drug/procedure (Dechallenge&Rechallenge Information) :**  Yes [ ] No [ ] NA [ ] | | |
| **Concomitant drugs history and lab investigations** | |  |
| **15. Concomitant drug (s) and date of administration** | |  |
| **16. Relevant test/ laboratory data with dates:** | |  |
| **17. Patient relevant history (e.g. diagnosis, allergies)** | |  |
| **Reaction information** | | |
| **18. Description of adverse event** | | |
| a. Start date (and time) of onset of reaction | |  |
| b. Stop date (and time) or duration of reaction | |  |
| c. Setting (e.g. hospital, out- patient clinic, home nursing home) | |  |
| d. [Full description of reaction(s) including body site and severity, as well as the criterion (or criteria) for regarding the report as serious. In addition to description of the reported signs and symptoms, whenever possible, describe a specific diagnosis for the reaction, indicate if this is follow-up report and if so, include follow-up information only]: | |  |
| **19. Describe the medical treatment provided for adverse reaction (if any) to research subject. This is an update on treatment given during hospitalization:** | |  |
| **20. Outcome:**  Resolved [ ] Ongoing [ ] Death [ ] | | |
| **21. Was the research continued on the research protocol?**  Yes [ ] No [ ] NA ( Mark ‘NA’ in case of death) [ ] | | |
| **22. Has this information been communicated to sponsor/ CRO/ regulatory agencies?**  Yes [ ] No [ ]  Provide details if communicated ( including date) : | | |
| **23. In your opinion, does this reaction require any alteration in trial protocol?**  Yes [ ] No [ ]  **If yes then please specify:** | | |
| **24. Causality Assessment:** | | |
| Signature : ………………………… Date : ………………  **Upon receipt of this report, the IEC will decide whether additional information is needed or whether further investigation of the reaction is required.** | | |

| **Discussion following EC review:**  Motion : [ ] Approved  [ ] Further clarification required  Clarifications :  **Reviewer’s Signature : ______________________________**  **Name of the Reviewer : ______________________________** |
| --- |

**Appendix T**

**Death Report Form – UCSF**

**Reporting format for Death of Participant**

| **Identification Information** | | | | |
| --- | --- | --- | --- | --- |
| Participant ID |  | Site Name | |  |
| Site code |  | | | |
| Date of Recruitment: - | | | | |
| Date of Completion of follow up: | | | | |
| Date of last interview: | | | | |
| PHQ score at the time of  the last interview: | | | Suicidal Ideation Score:  administered: Yes /No | |
| Referral: No | | | | |
| Referred to *(Mention details in other relevant information box)* | | | | |

| **Details on the death of the participant** | | | | |
| --- | --- | --- | --- | --- |
| Date of death (If known) |  | Date of death informed: | |  |
| Initials of the Staff to whom information conveyed: | | |  | |
| Date of verification |  | Initials of the Staff completed verification | |  |
| Source of verification |  | | | |

| Place of death |  |
| --- | --- |
| Cause of the death: |  |

| Reported to: | | | | |
| --- | --- | --- | --- | --- |
| Project Manager | Date |  | Reported by |  |
| UCSF | Date |  | Reported by |  |
| IEC | Date |  | Reported by |  |
| Principal investigators | Date |  | Reported by |  |
| Data team | Date |  | Reported by |  |

| Any other relevant information: |
| --- |

**Appendix U**

**NC script for women in the intervention arm – MI #4**

To the husband before the start of MI session 4:

Hello, can I speak to you [refer to the wife]/your wife [refer to the husband] separately for a short while…around 10-15 minutes? As you and I [refer to husband] have had 3 sessions to work together, I would like to speak to your wife briefly regarding what we have done here for the last three sessions and the plan that we have going forward. This is also an opportunity to get to know her just like we had a chance to get to know you in these past sessions.

With the wife:

Hello, my name is ______ and I work as a nurse-counselor in Project Harmony. We spoke on the phone earlier when I had called to schedule today’s session and also to remind you about this session. As you know, you & your husband have enrolled in this program as a couple to- nimma sambhandha vannu uttamagolisalu haagu, nimma gandana kudiyuva abhysavannu kadime maadalu. Over the past 3 weeks, I have had 1-hour long sessions once a week with your husband to help him cut down/stop his drinking. Today’s session is the last session in terms of focusing on his drinking. After today’s session, we will focus mainly on your relationship with your husband.

I know you have shared some information about what goes on at home and in your relationship with the research staff earlier. Since this is the first time you and I are meeting, I wanted to assure you that this is a safe place for you to talk about your issues and concerns. You can talk to me about these matters right now, if you would like…

Thank you for sharing these matters with me. I can see that you are going through a lot right now. (If the wife does not share anything, state the same). As our team has told you earlier, our goals are to bring down your husband’s heavy drinking, and to help improve your relationship with your husband, so that the environment at home is more peaceful and loving. In the coming 6-7 sessions we will look at and help you and your husband learn different ways in which you can better/strengthen your relationship with each other.

During this time, if there are instances where you feel unsafe in your own home or in your relationship [Nimma madhye jagala jaastiaaguthide, athava, nimage enadharu thondare aagabhudu emba hedarike idhare, athava nimma gandaninda thondare aagidhare…it is important that you contact us on our study phone number and let us know. If there is an increase in violent behavior of your husband and you feel he is going to hurt you, or he does indeed hurt you…it is important that you contact us on our study number, written here [show study number to wife].

I will not be able to speak to you separately after this even if you feel like you need to tell me something about your relationship. However, I assure you that the person with whom you will be speaking to will be a colleague of mine who will know your case and they will help you out. Since I am the support person for both of you, I cannot talk to you separately without your husband being present and I cannot talk to your husband separately without you being present.

So, if you wish to speak to someone regarding your relationship, we will connect you to another research team member for you to talk about what is on your mind. I assure you that I will do my best for you to feel like our sessions are a safe place for you to open up about your relationship matter and talk. Despite this if you feel like you would like to talk to someone, do not hesitate to let me know, or call our study phone number, and I will connect you to another research team member who can speak to you.

All our sessions are confidential…whatever you share in here or during the session will be private. I will do my best to ensure you feel listened to and understood during the upcoming couple sessions.

Do you have any questions…or any concerns related to what we just discussed?

**Appendix V**

**Script for women who report IPV – Control Arm**

*Hello. My name is ______ and I am a staff member of Project Harmony. Right before this, we spoke to your husband about his problems related to heavy drinking and provided him with referral letters to get help should he so desire. Before we begin, I would like to assure you that what you share with me here will not be disclosed to your husband. This is private and entirely confidential.*

*You have talked to other research staff here about the problems you have been experiencing at home and in your relationship with your husband. If you would like, you may talk to me about these issues too. I’m here to listen…*

*[Allow for participant to speak if she wishes to ventilate. If not, do not press for her to share.]*

*I understand that it is not easy to talk about these matters. Thank you for choosing to share these matters with us. You have told us that you have experienced all of this in the recent past. May I know if you are concerned/afraid that something like this may happen again?*

*[Encourage participant to share with you her fears or concerns revolving around the likelihood of recurrence of violence at home].*

*Your safety is a priority to us. In situations like the ones you have described, it is important that you feel safe and are safe. I am going to give you our study phone number – you can reach out to us for help in times where you feel unsafe in your own home or in your relationship. If you feel like your husband may hurt you, or if there is an incident of or increase in violence (screaming, throwing, pushing, hitting, forcing, controlling, etc.), we would like you to contact us on this number or let your ASHA know that you need help.*

*Please don’t hesitate to reach out to us (on our study phone number) in case you feel unsafe or if you feel there is a chance that you may get hurt. Your safety is of utmost priority, and we will try our best to ensure that you are in safe hands if you get in touch with us.*

*Do you have any questions about contacting us, or how to go about reaching out to us? Please feel free to ask me.*

Study phone number: *to be updated.*

**Appendix W**

**MI Certification Form**

**Date:**

**Nurse-counselor’s name:**

**Observer’s name:**

**SECTION 1 – CONTENT**

**Instructions:** *NC will be asked to conduct the following sections. Observers will indicate whether they think the NC has adequately demonstrated proficiency by marking 1 – Completed. If the observer thinks the NC did not demonstrate proficiency in conducting a given section and needs additional training, they may mark 0 – Not completed. Observers may note down comments if any, for discussion with other observers after evaluation.*

| **1. Setting the agenda. Task: NC is to set the agenda for any 1 of the 4 sessions.** | The NC explains the rationale behind setting the agenda to the participant. They set the agenda clearly for a given session by listing out all the sections they would be covering in that session in order. The NC checks in with the participants to see if they would like to add to the agenda for that session and if they have any questions. | - 1 – Completed. - 0 – Needs additional training.   Comments: |
| --- | --- | --- |
| **2. Help participant understand his drinking.** | NC uses scores and responses from AUDIT from baseline assessment, asks questions to elicit information about participant’s drinking patterns, summarizes, and provides additional information about implications of his current drinking patterns. Provides personalized feedback and psychoeducation. | - 1 – Completed. - 0 – Needs additional training.   Comments: |
| **3. Goal setting and action plan.** | NC helps to set drinking and other related goals; helps to set change and action plan (using the CAPW and Drinking Diary). | - 1 – Completed. - 0 – Needs additional training.   Comments: |
| **4. Conduct drink refusal skills training.** | NC explores and elicits information from the participant about situations in which participant gives in to having a drink. Asks the participant about how they usually respond when someone urges them to drink. NC suggests ways of responding and refusing to drink. NC role plays with participant to demonstrate skill. Asks participant if they have any concerns or questions, or if they foresee any challenges practicing the skill. | - 1 – Completed. - 0 – Needs additional training.   Comments: |
| **5. Conduct handling difficult emotions skills training.** | NC explains to the participant what emotions are. Asks participant if they think there is a relationship between emotions and drinking. Explores and explains relationship between strong emotions and drinking as a coping mechanism. Identifies situations in which participant most commonly experiences strong emotions.  Suggests grounding exercise. Helps participant practice grounding exercise in the session. Asks the participant if they have any concerns or questions, or if they foresee any challenges practicing the skill. Encourages the participant to practice grounding in less stressful *and* highly stressful situations alike. | - 1 – Completed. - 0 – Needs additional training.   Comments: |
| **6. Conduct problem-solving skills training.** | NC explores participant’s problems. Helps participant narrow down to one problem. Introduces the problem-solving steps. Provides rationale for using the steps. Takes participant through each step using the problem identified earlier. Asks participant if they have any questions or concerns. Explains that problem-solving is a skill that gets better with practice. | - 1 – Completed. - 0 – Needs additional training.   Comments: |
| **7. Conduct resisting the urge to drink skills.** | NC guides the participant in identifying triggers that lead to an urge. Explores strategies participant is already using to resist urges. Uses handout to suggest additional strategies to resist the urge to drink. Asks participant which of the suggested ways they would like to use in the following weeks. NC checks in with the participant to see if they have any concerns or questions. | - 1 – Completed. - 0 – Needs additional training.   Comments: |
| **8. Lapse and relapse prevention.** | NC explains lapse and relapse, differentiates between the two. Uses the chart to demonstrate triggers🡪urge🡪lapse🡪relapse. Identifies internal and external trigger.  Lapse and relapse prevention: identify trigger, avoid trigger, cope with triggers differently. | - 1 – Completed. - 0 – Needs additional training.   Comments: |
| **9. Lapse management.** | Lapse management: identify reasons for the lapse, revisit relevant skills, self-talk. | - 1 – Completed. - 0 – Needs additional training.   Comments: |
| **10. Relapse Management.** | Relapse management: identify reasons for relapse, revisits relevant skills. | - 1 – Completed. - 0 – Needs additional training.   Comments: |
| **11. Handouts.** | NC uses relevant handouts in a comprehensible manner while conducting the sections above. | - 1 – Completed. - 0 – Needs additional training.   Comments: |

**SECTION 2 – COUNSELOR SKILLS**

**Instructions:** *Observers will rate the NC on the following parameter at the end of the evaluation. The ratings for the following must be based on the NC’s overall performance across the sections.*

| **1. Rolling with resistance.** | **NC avoids a direct head-on argument or confrontation** with the participant, but instead simply reflects the resistance and demonstrates that they heard him. Shifts focus and encourages him to come up with possible solutions himself to help him feel empowered and not attacked. | - 0 – No. - 1 – Yes.   Comments: |
| --- | --- | --- |
| **2. Developing discrepancy.** | Builds awareness of the gap between the participant’s current behaviors and his goals/desired outcomes. Discourages sustain talk, creates or heightens the internal conflicts of the participant relative to his substance abuse. | - 0 – No. - 1 – Yes.   Comments: |
| **3. Change talk.** | Elicits discussion of change (self-motivational statements of change) through evocative questions/comments deigned to promote increased awareness of & concern for the problem, increase intent/optimism to change, or encourage elaboration on a topic related to change. | - 0 – No. - 1 – Yes.   Comments: |
| **4. Affirmations.** | Verbally reinforces participant’s strengths, abilities or efforts to change his/her behavior. | - 0 – No. - 1 – Yes.   Comments: |
| **5. Open-ended questions.** | Uses open-ended questions when appropriate to elicit information from and encourage change talk in the participant. | - 0 – No. - 1 – Yes.   Comments: |
| **6. Involving significant other (wife).** | Involves or enquires about involving wife in the sessions. | - 0 – No. - 1 – Yes.   Comments: |
| **7. Set/review homework.** | Plans/reminds participant about Action Plan and any specific homework. | - 0 – No. - 1 – Yes.   Comments: |
| **8. Appropriate use of silence and elicitation techniques** | The NC displays the deliberate use of silence to encourage the participant's self-exploration and autonomy, to elicit information and foster participant engagement in the session. Uses verbal ("Hmm", "and...", "Tell me more...", "I'd like to know more...") and non-verbal (steady eye contact, nodding, facial expressions, open body language, etc.) communication to allow and encourage participant to speak and elaborate on important matters. | - 0 – No. - 1 – Yes.   Comments: |
| **7. Handling “Difficult” Situations.** | During challenging or difficult situations during the session, the NC maintains composure and responds appropriately. | - 0 – No. - 1 – Yes.   Comments: |

**Appendix X**

**BCT Certification Form**

**Date:**

**Nurse-counselor’s name:**

**Observer’s name:**

**SECTION 1 – CONTENT**

**Instructions:** *NC will be asked to conduct the following sections. Observers will indicate whether they think the NC has adequately demonstrated proficiency by marking 1 – Completed. If the observer thinks the NC did not demonstrate proficiency in conducting a given section and needs additional training, they may mark 0. Observers may note down comments if any, for discussion with other observers after evaluation.*

| **1. Orient the participant to couples’ sessions.** | The NC introduces the participants to couple sessions by discussing all the couple sessions guidelines and the rationale behind each guideline. The NC enquires if the couple has any concerns or questions. | - 1 – Completed. - 0 – Needs additional training.   Comments: |
| --- | --- | --- |
| **2. Discuss relationship conflict and violence.** | The NC explores the nature of the couple’s relationship and relationship conflict – by gently probing the violent nature of the couple’s relationship. The NC must allow them to ventilate and provide sensible validation. NC explains that everyone experiences stress in their lives, and that some people deal with stressors by drinking heavily. The NC explains how people who have problems with drinking tend to have relationship problems. While the NC may normalize disagreement between couples, the NC must explain to the couple that there are better ways of resolving conflict between them, in ways that don’t negatively affect either partner. The NC then enquires if the couple would like to learn a few ways of improving the quality of their relationship. | - 1 – Completed. - 0 – Needs additional training.   Comments: |
| **3. Daily trust contract.** | Introduce the participants to the daily trust contract. Introduce the daily trust calendar and how to mark it upon completion. Address concerns, if any.  The NC models the trust discussion, followed by engaging the couple in a roleplay to practice the daily trust discussion in the session. NC strongly recommends that they have the daily trust discussion at home by fixing a time to do it. | - 1 – Completed. - 0 – Needs additional training.   Comments: |
| **4. Caring activity 1.** | The NC uses the couple guideline “Act Differently Before You Feel Differently” to provide a rationale for the caring activity. The NC helps each partner identify ways in which they may act more caringly towards the other. The NC hands them the “Catch your partner doing something nice!” handout to the couple to fill out and bring to the next session. | - 1 – Completed. - 0 – Needs additional training.   Comments: |
| **5. Caring activity 2.** | The NC reviews the previous caring activity and explains to the couple that it is important to notice their partner doing something nice for them *and* tell them how it made them feel. The NC models the skill and then encourages the couple to role play the Catch and Tell skill with each other. The NC encourages the couple to practice this at home whenever appropriate. | - 1 – Completed. - 0 – Needs additional training.   Comments: |
| **6. Coping skills.** | The NC discusses stressors faced by each partner. Introduces “Catch Yourself” and helps each partner identify their own signs of heightened emotions. NC then introduces Time-outs/Breaks. The NC facilitates the couple to practice breathing exercise. NC introduces positive self-talk and helps participants identify statements that would work for them. NC encourages the couple to apply these strategies to various stressful situations across varied settings. | - 1 – Completed. - 0 – Needs additional training.   Comments: |
| **7. Good communication.** | NC introduces and defines good communication using the sender-receiver illustration. Discusses components of good communication – non-verbal communication, tone of voice and taking turns. Facilitates participants to identify which aspects of their communication skills they would like to improve upon. Introduces and lays the ground rules for the communication session activity. Demonstrates the skill and facilitates practice of the activity by conducting roleplays by the couple. Encourages the couple to practice the skill at home 1-2 times over the following week. | - 1 – Completed. - 0 – Needs additional training.   Comments: |
| **8. Joint problem-solving.** | NC explains the rationale behind learning joint problem-solving skills. Takes an example from the couple’s life and facilitates joint problem-solving using the 5 steps and the handout. Helps the couple identify a problem (time-limited) that they may attempt to solve using the steps over the following week and review in the next session. | - 1 – Completed. - 0 – Needs additional training.   Comments: |
| **9. Active listening and understanding.** | NC uses the sender-receiver illustration to emphasize the importance of good listening skills. Discusses active listening and understanding. Provides examples and rationale for using the skill. Helps each partner practice the skill while the other partner speaks on a topic that makes them experience negative emotions. NC suggests sentence stems that they may use to engage in active listening and understanding. | - 1 – Completed. - 0 – Needs additional training.   Comments: |
| **10. Expressing feelings directly.** | NC discusses and normalizes disagreements in relationships and suggests that it is important to express negative feelings towards one’s partner in a civil manner. Describes and distinguishes between blaming statements and I-statements. Provides the “formula” for expressing feelings directly using I-statements. Provides examples of their own. Facilitates practice of the skill through roleplays between the couple. Provides feedback. | - 1 – Completed. - 0 – Needs additional training.   Comments: |
| **11. Revisit lapse and relapse prevention and management.** | NC enquires if participants can continue to distinguish between a lapse and relapse. Reviews skills used by participant in avoiding triggers, resisting urges, preventing and managing lapses and relapses. Emphasizes that “inconsistent progress is the rule” and suggests that participants not be disheartened by minor setbacks, but instead continue practicing the skills and steadily put in effort to maintain progress. | - 1 – Completed. - 0 – Needs additional training.   Comments: |
| **12. Make sustainable relationship goals.** | NC uses BCT activities master sheet to collaboratively mark the activities that the couple would like to keep practicing after sessions come to an end, help them ascertain the frequency of the activities as well. Identify potential challenges in carrying selected activities out. Help address foreseen challenges. | - 1 – Completed. - 0 – Needs additional training.   Comments: |
| **13. Handouts.** | NC uses relevant handouts in a comprehensible manner while conducting the sections above. | - 1 – Completed. - 0 – Needs additional training.   Comments: |

**SECTION 2 – COUNSELOR SKILLS**

**Instructions:** *Observers will rate the NC on the following parameter at the end of the evaluation. The ratings for the following must be based on the NC’s overall performance across the sections.*

| **1. Therapist neutrality.** | The NC emphasizes couple goals and appears to actively work toward enhancing the quality of their relationship and interactions. The NC does not take sides and does not pit one partner against the other. The NC does not appear unduly biased toward one partner. Attempts to engage both partners as equally as possible. | - 0 – No. - 1 – Yes.   Comments: |
| --- | --- | --- |
| **2. Examples.** | NC uses relevant examples wherever appropriate to illustrate a concept to the couple effectively. | - 0 – No. - 1 – Yes.   Comments: |
| **3. Poise.** | NC appears confident. Does not hesitate in addressing concerns. Well organized, not nervous | - 0 – No. - 1 – Yes.   Comments: |
| **4. Rapport.** | NC gets participants talking; friendly and warm; uses participants’ name when appropriate; seems to understand the participants’ needs and care about them. | - 0 – No. - 1 – Yes.   Comments: |
| **5. Pace.** | NC has a steady flow. Does not rush participants; does not speak too fast but is able to cover all the components; appears relaxed. | - 0 – No. - 1 – Yes.   Comments: |
| **6. Appropriate use of silence and elicitation techniques** | The NC displays the deliberate use of silence to encourage the participant's self-exploration and autonomy, to elicit information and foster participant engagement in the session. Uses verbal ("Hmm", "and...", "Tell me more...", "I'd like to know more...") and non-verbal (steady eye contact, nodding, facial expressions, open body language, etc.) communication to allow and encourage participant to speak and elaborate on important matters. | - 0 – No. - 1 – Yes.   Comments: |
| **7. Handling “Difficult” Situations.** | During challenging or difficult situations during the session, the NC maintains composure and responds appropriately. | - 0 – No. - 1 – Yes. - Comments: |

**Appendix Y**

**MI Fidelity Proforma**

| The purpose of the observation form is to measure the fidelity and quality of implementation of counseling delivery of Motivational Interviewing sessions for *Harmony.* **Prior to administering this form, please ensure the participant/couple has given permission for the counselling session to be observed.** Use your best judgment based on your observations, and *do not* change the scoring provided. **You should complete the observation form *after viewing the entire session*, but you should read through the questions prior to the observation.** | | | | | | | | |
| --- | --- | --- | --- | --- | --- | --- | --- | --- |
| **PART I. CONTENT COVERAGE** | | | | | | | | |
| *Instructions:* For each of the activities in the session, indicate whether the nurse-counselor completed it, modified it, or did not complete it. Modifications might include adding new content or activities, or changing the way you talk about something compared to the manual’s instructions. | | | | | | | | |
| **Consent:** Did the couple provide permission to be observed? | | | ☐_1_  Yes ☐_2_ No | | | | | |
|  | Counselor name: | |  | | | | | |
|  | Observer name: | |  | | | | | |
| **Session One.** Did the nurse-counselor complete each activity below? | | | | | | | | |
| **1. Introduces participant to MI sessions.** | | Explains the number, duration and frequency of sessions. Explains in brief about the content of the sessions: Assessment, feedback, deciding on treatment goals, helping the patient achieve drinking goals and helping the patient to maintain any changes that he makes to his drinking. | | **0**  Not Done | **1**  Poor | **2**  Adequate | **3**  Good | **4**  Excellent |
| **2. Provides psychoeducation**  **and personalized feedback.** | | Helps patient to better understand drinking based on AUDIT assessment; summarizes patient drinking; gives patient personalized feedback. | | **0**  Not Done | **1**  Poor | **2**  Adequate | **3**  Good | **4**  Excellent |
| **3. Goal Setting and Action Plan** | | Helps to set drinking and other-related goals; helps to set change plan and action plan. | | **0**  Not Done | **1**  Poor | **2**  Adequate | **3**  Good | **4**  Excellent |
| **4. Commitment** | | Asks for commitment from patient about agreed- upon goals | | **0**  Not Done | **1**  Poor | **2**  Adequate | **3**  Good | **4**  Excellent |
| **Session Two and Three. Did the nurse-counselor complete each activity below?** | | | | | | | | |
| **5. Drink Refusal Skills** | | Works with patient to develop skills that the patient can use to say 'NO' when offered or tempted by a drink. | | **0**  Not Done | **1**  Poor | **2**  Adequate | **3**  Good | **4**  Excellent |
| **6. Handling Urges Skills** | | Generates strategies to handle drinking urges in collaboration with the patient. | | **0**  Not Done | **1**  Poor | **2**  Adequate | **3**  Good | **4**  Excellent |
| **7.Handling Emotions Skills** | | Helps the patient to develop the skills for recognising their emotions accurately, identifying the thoughts behind the emotions, identifying the consequences of the thoughts and finally challenging the negative thoughts and replacing them with positive thoughts. | | **0**  Not Done | **1**  Poor | **2**  Adequate | **3**  Good | **4**  Excellent |
| **8. Problem-Solving Skills** | | Helps the patient to learn the skills of identifying problems that are contributing to their drinking problem, generate multiple solutions, apply the most appropriate one and review the solution for its effectiveness. | | **0**  Not Done | **1**  Poor | **2**  Adequate | **3**  Good | **4**  Excellent |
| **9. Homework** | | Reviews planned action plan and/or specific homework. | | **0**  Not Done | **1**  Poor | **2**  Adequate | **3**  Good | **4**  Excellent |
| **Session Four. Did the nurse-counselor complete each activity below?** | | | | | | | | |
| **10. Reviewed Skills** | | Reviewed problem-solving skills, drink-refusal skills, handling urges and emotions. | | **0**  Not Done | **1**  Poor | **2**  Adequate | **3**  Good | **4**  Excellent |
| **11. Lapse/Relapse Management** | | Helps patient to identify triggers for lapse, develop strategies to prevent exposure to triggers and cope with triggers differently. If lapse has already occurred, then help the patient to prevent it turning into a relapse. If relapse has already occurred, then help patient to develop skills to prevent recurrence. | | **0**  Not Done | **1**  Poor | **2**  Adequate | **3**  Good | **4**  Excellent |
| **12. Reviewed all Sessions** | | Asks patient to summarise all of the information and ideas and skills and techniques covered in all the sessions, and then adding in any extra ones that they have forgotten. | | **0**  Not Done | **1**  Poor | **2**  Adequate | **3**  Good | **4**  Excellent |
| **PART 2. MI-SPECIFIC SKILLS** | | | | | | | | |
| **1. Evoke and Encourages Change Talk** | | Elicits discussion of change (self-motivational statements of change) through questions/comments deigned to promote ↑ awareness/concern for the problem, increase intent/optimism to change, or encourage elaboration on a topic related to change. | | **0**  Not Done | **1**  Poor | **2**  Adequate | **3**  Good | **4**  Excellent |
| **2. Independence** | | Seeks to enhance patient’s sense of control and freedom of choice. | | **0**  Not Done | **1**  Poor | **2**  Adequate | **3**  Good | **4**  Excellent |
| **3. Navigation** | | Manages the conversation that discussion of the drinking behaviour change remains the focus without causing resistant behaviour. | | **0**  Not Done | **1**  Poor | **2**  Adequate | **3**  Good | **4**  Excellent |
| **4. Information and advice** | | Gives accurate and relevant information or advice with skilfulness. | | **0**  Not Done | **1**  Poor | **2**  Adequate | **3**  Good | **4**  Excellent |
| **5. Affirmation** | | Verbally reinforces client’s strengths, abilities or efforts to change his/her behavior. | | **0**  Not Done | **1**  Poor | **2**  Adequate | **3**  Good | **4**  Excellent |
| **6. Roll with resistance** | | Provide low-key feedback, roll with resistance (e.g., avoiding arguments, shifting focus) and use of a supportive, warm, non-judgemental approach. | | **0**  Not Done | **1**  Poor | **2**  Adequate | **3**  Good | **4**  Excellent |
| **7. Discourages sustain talk & encourages discrepancies** | | Creates or heightens the internal conflicts of the client relative to his substance abuse. | | **0**  Not Done | **1**  Poor | **2**  Adequate | **3**  Good | **4**  Excellent |
| **8. Agenda setting** | | Creates a plan with the patient at the beginning of the session which includes a list of topics to discuss or tasks to complete in the session. | | **0**  Not Done | **1**  Poor | **2**  Adequate | **3**  Good | **4**  Excellent |
| **9. Involves wife** | | Involves or enquires about involving wife in treatment. | | **0**  Not Done | **1**  Poor | **2**  Adequate | **3**  Good | **4**  Excellent |
| **10. Sets/reviews homework** | | Planned/reminds about Action Plan and any specific homework. | | **0**  Not Done | **1**  Poor | **2**  Adequate | **3**  Good | **4**  Excellent |
| **11. Deals with other barriers arising in session** | | Deals with other challenges that arise in the session (e.g., patient comes in drunk, wants medication, expecting directive advice). | | **0**  Not Done | **1**  Poor | **2**  Adequate | **3**  Good | **4**  Excellent |
| **PART 3. GENERAL SKILLS** | | | | | | | | |
| **1. Non-Judgmental** | | Is non-judgemental and matter of fact in communication with patient. | | **0**  Not Done | **1**  Poor | **2**  Adequate | **3**  Good | **4**  Excellent |
| **2. Encouraging** | | Encourages the patient’s progress even in fact of obstacles. | | **0**  Not Done | **1**  Poor | **2**  Adequate | **3**  Good | **4**  Excellent |
| **3. Expresses Warmth** | | Displays warmth during the session and appears natural and genuine in interactions with patient. | | **0**  Not Done | **1**  Poor | **2**  Adequate | **3**  Good | **4**  Excellent |
| **4. Acknowledges participant’s experience** | | Shows that he/she understood patient’s experience. | | **0**  Not Done | **1**  Poor | **2**  Adequate | **3**  Good | **4**  Excellent |
| **5. Empathy** | | Attempts to demonstrate accurate understand. | | **0**  Not Done | **1**  Poor | **2**  Adequate | **3**  Good | **4**  Excellent |
| **6. Collaboration** | | Conveys words/actions that therapy is collaborative (vs. counsellor being in charge). | | **0**  Not Done | **1**  Poor | **2**  Adequate | **3**  Good | **4**  Excellent |
| **7. Summarize** | | Reinforces what has been said, shows that he/she has been listening carefully and prepares participant to move on. | | **0**  Not Done | **1**  Poor | **2**  Adequate | **3**  Good | **4**  Excellent |
| **8. Open-ended**  **Questions** | | Appropriate use of open-ended questions (beyond yes/no responses). | | **0**  Not Done | **1**  Poor | **2**  Adequate | **3**  Good | **4**  Excellent |
| **9. Active Listening** | | Demonstrates effective listening through non- verbal behaviour (e.g., maintaining eye contact)  and verbal behaviour (e.g., reflection). | | **0**  Not Done | **1**  Poor | **2**  Adequate | **3**  Good | **4**  Excellent |
| **10. Selective Reflection** | | Selectively reflects content and feelings which promote change talk and reduce sustain talk. | | **0**  Not Done | **1**  Poor | **2**  Adequate | **3**  Good | **4**  Excellent |

**Appendix Z**

**BCT Fidelity Proforma**

| The purpose of the observation form is to measure the fidelity and quality of implementation of counseling delivery of Behavioral Couples Therapy for *Harmony.* **Prior to administering this form, please ensure the couple has given permission for the counselling session to be observed.** Use your best judgment based on your observations, and *do not* change the scoring provided. **You should complete the observation form *after viewing the entire session*, but you should read through the questions prior to the observation.** | | | |
| --- | --- | --- | --- |
| **PART I. CONTENT COVERAGE** | | | |
| *Instructions:* For each of the activities in the session, indicate whether the nurse-counselor completed it, modified it, or did not complete it. Modifications might include adding new content or activities, or changing the way you talk about something compared to the manual’s instructions. | | | |
| **Consent:** Did the couple provide permission to be observed? | | | ☐_1_  Yes ☐_2_ No |
|  | Counselor name: | |  |
|  | Observer name: | |  |
| **Session One.** Did the nurse-counselor complete each activity below? | | | |
|  | Review MI strategies. (Check one) | ☐_1_ Yes completely  ☐_2_ No  ☐_3_ Yes with changes (Describe: ___________________________________________  ________________________________________________________________________)  ☐_4_ N/A, different session | |
|  | Discuss alcohol use and relationship conflict.  (Check one) | ☐_1_ Yes completely  ☐_2_ No  ☐_3_ Yes with changes (Describe: ___________________________________________  ________________________________________________________________________)  ☐_4_ N/A, different session | |
|  | Introduce couple sessions.  (Check one) | ☐_1_ Yes completely  ☐_2_ No  ☐_3_ Yes with changes (Describe: ___________________________________________  ________________________________________________________________________)  ☐_4_ N/A, different session | |
|  | Introduce caring activity: *Catch Your Partner Doing Something Nice!*  (Check one) | ☐_1_ Yes completely  ☐_2_ No  ☐_3_ Yes with changes (Describe: ___________________________________________  ________________________________________________________________________)  ☐_4_ N/A, different session | |
|  | Introduce Daily Trust Contract. (Check one) | ☐_1_ Yes completely  ☐_2_ No  ☐_3_ Yes with changes (Describe: ___________________________________________  ________________________________________________________________________)  ☐_4_ N/A, different session | |
|  | Practice Daily Trust Discussion. (Check one) | ☐_1_ Yes completely  ☐_2_ No  ☐_3_ Yes with changes (Describe: ___________________________________________  ________________________________________________________________________)  ☐_4_ N/A, different session | |
| **Session Two.** Did the nurse-counselor complete each activity below? | | | |
|  | Discuss and address concerns regarding couple sessions [if any].  (Check one) | ☐_1_ Yes completely  ☐_2_ No  ☐_3_ Yes with changes (Describe: ___________________________________________  ________________________________________________________________________)  ☐_4_ N/A, different session | |
|  | Revisit couple session guidelines. (Check one) | ☐_1_ Yes completely  ☐_2_ No  ☐_3_ Yes with changes (Describe: ___________________________________________  ________________________________________________________________________)  ☐_4_ N/A, different session | |
|  | Review caring activity: *Catch Your Partner Doing Something Nice!*  (Check one) | ☐_1_ Yes completely  ☐_2_ No  ☐_3_ Yes with changes (Describe: ___________________________________________  ________________________________________________________________________)  ☐_4_ N/A, different session | |
|  | Daily Trust Contract calendar review.  (Check one) | ☐_1_ Yes completely  ☐_2_ No  ☐_3_ Yes with changes (Describe: ___________________________________________  ________________________________________________________________________)  ☐_4_ N/A, different session | |
|  | Practice Daily Trust Discussion. (Check one) | ☐_1_ Yes completely  ☐_2_ No  ☐_3_ Yes with changes (Describe: ___________________________________________  ________________________________________________________________________)  ☐_4_ N/A, different session | |
|  | Introduce *Catch Yourself!*  (Check one) | ☐_1_ Yes completely  ☐_2_ No  ☐_3_ Yes with changes (Describe: ___________________________________________  ________________________________________________________________________)  ☐_4_ N/A, different session | |
|  | Introduce *Time-outs/Breaks.* (Check one) | ☐_1_ Yes completely  ☐_2_ No  ☐_3_ Yes with changes (Describe: ___________________________________________  ________________________________________________________________________)  ☐_4_ N/A, different session | |
|  | Introduce breathing exercise. (Check one) | ☐_1_ Yes completely  ☐_2_ No  ☐_3_ Yes with changes (Describe: ___________________________________________  ________________________________________________________________________)  ☐_4_ N/A, different session | |
|  | Practice breathing exercise. (Check one) | ☐_1_ Yes completely  ☐_2_ No  ☐_3_ Yes with changes (Describe: ___________________________________________  ________________________________________________________________________)  ☐_4_ N/A, different session | |
|  | Introduce positive self-talk.  (Check one) | ☐_1_ Yes completely  ☐_2_ No  ☐_3_ Yes with changes (Describe: ___________________________________________  ________________________________________________________________________)  ☐_4_ N/A, different session | |
| **Session Three.** Did the nurse-counselor complete each activity below? | | | |
|  | Daily Trust Contract Calendar review.  (Check one) | ☐_1_ Yes completely  ☐_2_ No  ☐_3_ Yes with changes (Describe: ___________________________________________  ________________________________________________________________________)  ☐_4_ N/A, different session | |
|  | Practice Daily Trust Discussion. (Check one) | ☐_1_ Yes completely  ☐_2_ No  ☐_3_ Yes with changes (Describe: ___________________________________________  ________________________________________________________________________)  ☐_4_ N/A, different session | |
|  | Review breathing exercise. (Check one) | ☐_1_ Yes completely  ☐_2_ No  ☐_3_ Yes with changes (Describe: ___________________________________________  ________________________________________________________________________)  ☐_4_ N/A, different session | |
|  | Introduce and define good communication. (Check one) | ☐_1_ Yes completely  ☐_2_ No  ☐_3_ Yes with changes (Describe: ___________________________________________  ________________________________________________________________________)  ☐_4_ N/A, different session | |
|  | Introduce nonverbal communication, taking turns and not interrupting the other person. (Check one) | ☐_1_ Yes completely  ☐_2_ No  ☐_3_ Yes with changes (Describe: ___________________________________________  ________________________________________________________________________)  ☐_4_ N/A, different session | |
|  | Model communication session with one partner. (Check one) | ☐_1_ Yes completely  ☐_2_ No  ☐_3_ Yes with changes (Describe: ___________________________________________  ________________________________________________________________________)  ☐_4_ N/A, different session | |
|  | Practice communication session.  (Check one) | ☐_1_ Yes completely  ☐_2_ No  ☐_3_ Yes with changes (Describe: ___________________________________________  ________________________________________________________________________)  ☐_4_ N/A, different session | |
|  | Introduce S.O.L.V.E. joint problem-solving strategy. (Check one) | ☐_1_ Yes completely  ☐_2_ No  ☐_3_ Yes with changes (Describe: ___________________________________________  ________________________________________________________________________)  ☐_4_ N/A, different session | |
|  | Practice S.O.L.V.E. join problem-solving strategy. (Check one) | ☐_1_ Yes completely  ☐_2_ No  ☐_3_ Yes with changes (Describe: ___________________________________________  ________________________________________________________________________)  ☐_4_ N/A, different session | |
| **Session Four.** Did the nurse-counselor complete each activity below? | | | |
|  | Daily Trust Contract Calendar review.  (Check one) | ☐_1_ Yes completely  ☐_2_ No  ☐_3_ Yes with changes (Describe: ___________________________________________  ________________________________________________________________________)  ☐_4_ N/A, different session | |
|  | Practice Daily Trust Discussion.  (Check one) | ☐_1_ Yes completely  ☐_2_ No  ☐_3_ Yes with changes (Describe: ___________________________________________  ________________________________________________________________________)  ☐_4_ N/A, different session | |
|  | Review communication session.  (Check one) | ☐_1_ Yes completely  ☐_2_ No  ☐_3_ Yes with changes (Describe: ___________________________________________  ________________________________________________________________________)  ☐_4_ N/A, different session | |
|  | Review joint problem-solving.  (Check one) | ☐_1_ Yes completely  ☐_2_ No  ☐_3_ Yes with changes (Describe: ___________________________________________  ________________________________________________________________________)  ☐_4_ N/A, different session | |
|  | Review caring activity: *Catch Your Partner Doing Something Nice!*  (Check one) | ☐_1_ Yes completely  ☐_2_ No  ☐_3_ Yes with changes (Describe: ___________________________________________  ________________________________________________________________________)  ☐_4_ N/A, different session | |
|  | Introduce *Catch and Tell* caring activity.  (Check one) | ☐_1_ Yes completely  ☐_2_ No  ☐_3_ Yes with changes (Describe: ___________________________________________  ________________________________________________________________________)  ☐_4_ N/A, different session | |
|  | Model *Catch and Tell* caring activity.  (Check one) | ☐_1_ Yes completely  ☐_2_ No  ☐_3_ Yes with changes (Describe: ___________________________________________  ________________________________________________________________________)  ☐_4_ N/A, different session | |
|  | Practice *Catch and Tell* caring activity.  (Check one) | ☐_1_ Yes completely  ☐_2_ No  ☐_3_ Yes with changes (Describe: ___________________________________________  ________________________________________________________________________)  ☐_4_ N/A, different session | |
| **Session Five.** Did the nurse-counselor complete each activity below? | | | |
|  | Daily Trust Contract Calendar review.  (Check one) | ☐_1_ Yes completely  ☐_2_ No  ☐_3_ Yes with changes (Describe: ___________________________________________  ________________________________________________________________________)  ☐_4_ N/A, different session | |
|  | Practice daily trust discussion.  (Check one) | ☐_1_ Yes completely  ☐_2_ No  ☐_3_ Yes with changes (Describe: ___________________________________________  ________________________________________________________________________)  ☐_4_ N/A, different session | |
|  | Review *Catch and Tell* caring activity.  (Check one) | ☐_1_ Yes completely  ☐_2_ No  ☐_3_ Yes with changes (Describe: ___________________________________________  ________________________________________________________________________)  ☐_4_ N/A, different session | |
|  | Introduce Listening and Understanding.  (Check one) | ☐_1_ Yes completely  ☐_2_ No  ☐_3_ Yes with changes (Describe: ___________________________________________  ________________________________________________________________________)  ☐_4_ N/A, different session | |
|  | Model Listening and Understanding. (Check one) | ☐_1_ Yes completely  ☐_2_ No  ☐_3_ Yes with changes (Describe: ___________________________________________  ________________________________________________________________________)  ☐_4_ N/A, different session | |
|  | Practice Listening and Understanding. (Check one) | ☐_1_ Yes completely  ☐_2_ No  ☐_3_ Yes with changes (Describe: ___________________________________________  ________________________________________________________________________)  ☐_4_ N/A, different session | |
|  | Introduce Expressing Feelings Using I-Statements. (Check one) | ☐_1_ Yes completely  ☐_2_ No  ☐_3_ Yes with changes (Describe: ___________________________________________  ________________________________________________________________________)  ☐_4_ N/A, different session | |
|  | Practice Expressing Feelings Using I-Statements. (Check one) | ☐_1_ Yes completely  ☐_2_ No  ☐_3_ Yes with changes (Describe: ___________________________________________  ________________________________________________________________________)  ☐_4_ N/A, different session | |
| **Session Six.** Did the nurse-counselor complete each activity below? | | | |
|  | Daily Trust Contract Calendar review.  (Check one) | ☐_1_ Yes completely  ☐_2_ No  ☐_3_ Yes with changes (Describe: ___________________________________________  ________________________________________________________________________)  ☐_4_ N/A, different session | |
|  | Practice daily trust discussion.  (Check one) | ☐_1_ Yes completely  ☐_2_ No  ☐_3_ Yes with changes (Describe: ___________________________________________  ________________________________________________________________________)  ☐_4_ N/A, different session | |
|  | Review breathing exercise and other coping activities. (Check one) | ☐_1_ Yes completely  ☐_2_ No  ☐_3_ Yes with changes (Describe: ___________________________________________  ________________________________________________________________________)  ☐_4_ N/A, different session | |
|  | Review definition of good communication, nonverbal communication, taking turns and not interrupting the other person. (Check one) | ☐_1_ Yes completely  ☐_2_ No  ☐_3_ Yes with changes (Describe: ___________________________________________  ________________________________________________________________________)  ☐_4_ N/A, different session | |
|  | Review listening and understanding. (Check one) | ☐_1_ Yes completely  ☐_2_ No  ☐_3_ Yes with changes (Describe: ___________________________________________  ________________________________________________________________________)  ☐_4_ N/A, different session | |
|  | Review Expressing Feelings Using I-Statements. (Check one) | ☐_1_ Yes completely  ☐_2_ No  ☐_3_ Yes with changes (Describe: ___________________________________________  ________________________________________________________________________)  ☐_4_ N/A, different session | |
|  | Review joint problem-solving.  (Check one) | ☐_1_ Yes completely  ☐_2_ No  ☐_3_ Yes with changes (Describe: ___________________________________________  ________________________________________________________________________)  ☐_4_ N/A, different session | |
|  | Review caring activities. (Check one) | ☐_1_ Yes completely  ☐_2_ No  ☐_3_ Yes with changes (Describe: ___________________________________________  ________________________________________________________________________)  ☐_4_ N/A, different session | |
|  | Discuss sustaining improvement.  (Check one) | ☐_1_ Yes completely  ☐_2_ No  ☐_3_ Yes with changes (Describe: ___________________________________________  ________________________________________________________________________)  ☐_4_ N/A, different session | |
|  | Discuss relapse prevention.  (Check one) | ☐_1_ Yes completely  ☐_2_ No  ☐_3_ Yes with changes (Describe: ___________________________________________  ________________________________________________________________________)  ☐_4_ N/A, different session | |

**PART 2. QUALITY ASSESSMENT**

| **Part 2. Quality Assessment.** The following questions assess the overall quality of the counseling session and delivery of the information. Use your best judgment and do not circle more than one response. Guidelines for the interpretation of responses follow each scale. | | | |
| --- | --- | --- | --- |
|  | Rate whether the nurse-counselor solicited participants’ ideas/opinions (rather than just educating participants or giving opinions about what to do). Check one:  **☐ 1 ☐ 2 ☐ 3 ☐ 4 ☐ 5 Never Sometimes Often** | | |
| Ex. | 1 - The nurse-counselor might focus on just giving information to the participants, tell them what to do, or convey cynicism about the participants’ opinions/plan.  3 – The nurse-counselor attempts to solicit the participants’ idea and opinions a couple of times but may still adopt a more informational/instructional approach most of the time.  5 - Elicits the participants’ opinions and thoughts about need for relationship dynamic change, if applicable. | | |
|  | How often did the nurse-counselor use relevant examples to illustrate concepts? Check one:  **☐ 1 ☐ 2 ☐ 3 ☐ 4 ☐ 5**  **Never Sometimes Often** | | |
| Ex. | 1 - Counselor covers material straight from the manual without any further explanation, even when needed.  3 - Sometimes gives examples, but not when it would have been helpful.  5 - Gives examples that are personally relevant to the participants at appropriate/helpful points in time during session. | | |
|  | To what extent did the participants appear to understand the material? Check one:  **☐ 1 ☐ 2 ☐ 3 ☐ 4 ☐ 5**  **Never Sometimes Often** | | |
| Ex. | Use your best judgment based on participants’ conversations and feedback. Roughly:  1 - Less than 25% of content understood by participants.  3 - About 50% understood.  5 - 75-100% understood. | | |
|  | How actively did the participant join in counseling discussions? (Note: this isn’t necessarily a reflection of the counseling quality.) Check one:  **☐ 1 ☐ 2 ☐ 3 ☐ 4 ☐ 5**  **Never Sometimes Often** | | |
| Ex. | Use your best judgment based on listening to the discussions and feedback. | | |
|  | To what extent did the nurse-counselor manage the pace and flow of the session? Check one:  **☐ 1 ☐ 2 ☐ 3 ☐ 4 ☐ 5**  **Managed poorly Managed somewhat well Managed well** | | |
| Ex. | 1 - Counselor is rushed (doesn’t allow time for discussion; doesn’t have time for examples; tells participants they are in a hurry; or body language suggests stress/hurry), or regularly allows discussions to drag on.  3 – Somewhat rushed or sometimes allows discussions to drag on.  5 – Does not rush participants; does not speak too fast but is able to cover all the components; appears relaxed. | | |
| **On the following scale, rate the nurse-counselor on the following qualities. Check one:** | | | |
|  | Knowledge of the counseling manual content. Check one:  **☐ 1 ☐ 2 ☐ 3 ☐ 4 ☐ 5**  **Poor Average Excellent** | | |
| Ex. | 1 - Cannot answer questions, mispronounces words; reads from the manual.  2 – The nurse-counselor reads out from the manual or fumbles on a few words a couple of times but is otherwise able to respond to questions moderately well; is familiar with roughly half of the concepts.  5 – Seems very familiar with the concepts and answers questions with ease. | | |
|  | Level of enthusiasm/engagement. Check one:  **☐ 1 ☐ 2 ☐ 3 ☐ 4 ☐ 5**  **Poor Average Excellent** | | |
| Ex. | 1 - Presents information in a dry and boring way; monotonous; lacks personal connection to material; appears “burnt out.”  3 – Occasionally appears “zoned out” or disengaged; attempts to refocus attention, sometimes appears distracted/bored; fluctuations in level of engagement.  5 - Effectively promotes/communicates about the intervention in a positive way. | | |
|  | Poise and confidence. Check one:  **☐ 1 ☐ 2 ☐ 3 ☐ 4 ☐ 5**  **Poor Average Excellent** | | |
| Ex. | 1 - Appears nervous or hurried; poor eye contact; speaks inaudibly; appears hesitant/shy.  3 – Appears somewhat confident in conducting the session but occasionally appears hesitant/nervous/shy.  5 - Does not hesitate in addressing concerns. Well organized, not nervous. | | |
|  | Rapport and communication with participants. Check one:  **☐ 1 ☐ 2 ☐ 3 ☐ 4 ☐ 5**  **Poor Average Excellent** | | |
| Ex. | 1 – Doesn’t remember names; does not “connect” with participants; acts distant, cold, or unfriendly.  3 – Tries to get to know the participants by asking a few relevant questions but appears rehearsed or forced; is somewhat able to connect with participants  5 - Gets participants talking; friendly and warm; uses participants’ name when appropriate; seems to understand the participants’ needs and cares about these. | | |
|  | Effectively addressed questions/concerns. Check one:  **☐ 1 ☐ 2 ☐ 3 ☐ 4 ☐ 5**  **Poor Average Excellent** | | |
| Ex. | 1 - Responds negatively to comments; dismisses concerns and queries; gives inaccurate information; doesn’t connect participants with referral agency information effectively.  3 – Sometimes provides accurate information; occasionally deflects queries/concerns/questions.  5 - Answers questions with correct information in a non-judgmental way; if doesn’t know the answer, is honest about it and directs them elsewhere. | | |
|  | Rate the overall quality of the counseling session. Check one:  **☐ 1 ☐ 2 ☐ 3 ☐ 4 ☐ 5**  **Poor Average Excellent** | | |
| Ex. | Summary measure of all preceding questions. Assesses both the extent of material covered and the performance of the nurse-counselor. **Check off behaviors exhibited in the session**: | | |
|  | **Poor sessions look like:**   - Lecturing tone. - Read the content straight from the manual, which made the session feel scripted/impersonal. - Stumbled along with the content and failed to make connections to what has been discussed previously or what participants bring up. - Unengaged participants. - Judgmental responses. - Flat affect and boring style. - Unorganized and random. - Loses track of time. | | **Excellent sessions look like:**   - Participants are engaged, feel supported. - Discussion of personal stories or specific examples. - Non-judgmental responses to participant questions. - Answering questions with correct information. - Well organized. - Adequate pacing—not too fast and did not drag. - Used effective checks for participant understanding. |
|  | **[Optional]** Briefly describe any implementation problems you noticed, including any major changes to the content or delivery of the material; time wasted in getting the session started or finished, etc.: | | |
|  | Please note at least one major strength of the session and/or nurse-counselor’s delivery of the material: | | |
|  | **[Optional] Other Comments:** Use the space below for additional comments regarding strengths or weaknesses of the session, particularly if there is anything that affected your ratings above. | | |
|  | Duration of counseling session | ☐ hour(s), ☐☐ minutes | |
| 69. | **Calculate** average score from questions 54-64  *Note: A score below 3.3 requires additional training of the nurse-counselor.* | Calculate Average ___________________ | |

**Appendix AA**

**Control Arm: Brief Alcohol Educational Module**

**Mode:** One-on-one**,** in-person

**No. of sessions:** 01

**Duration:** 45 to 50 minutes

**Steps:**

1. Introduction and rapport building.
2. Beginning the conversation about alcohol.
3. Elicit participant’s attitudes towards and perceptions about drinking.
4. Psychoeducation.
5. Dispel/address myths and misconceptions about consuming alcohol.
6. Refer to NIMHANS.

**Step 1. Introduction and rapport building**.

The practitioner greets the participant and introduces themselves and the project. The practitioner holds a conversation with the participant enquiring about how they are doing, their age, their current employment status, current living arrangement (who they live with and their relationship with them), their routine, and recreational activities. Gently enquire about what kind of problems or stressors they may be facing currently.

Talking points:

*Hello, _____. I am ______, and I represent Project Harmony. As you know, the project that you are participating in looks at helping people find ways of reducing their drinking and thereby improve other important parts of their lives. I understand you have gone through a round of questions by the research staff here, which I may ask you again about. I will take about half an hour to 1 hour of your time today so that we can talk about some of the problems you may be facing and see if we could help you help for this. How does that sound?*

*Could you tell me a little more about yourself? How old are you?...... What do you do for a living? ……Who do you live with currently?..... What does your regular day look like?.... What are your past time activities?....*

**Step 2. Beginning the conversation about alcohol.**

The practitioner normalizes the experience of stress in everyday life and then invites the participant to talk about their stressors and problems. The practitioner validates their experience of stress and introduces the concept of coping. After inviting the participant to share their coping strategies, irrespective of whether they mention alcohol or not, the practitioner links the topic of coping with heavy drinking. Practitioner then gently enquires participant’s drinking behaviors.

Talking points: *Everyone experiences difficulties and stress in their daily lives in different forms. What would you say are matters that are causing stress in your life currently or have caused a lot of stress in the past?*

*I can imagine how stressful that can be! When a person faces challenges or is stressed out, they deal or cope with stress in different ways. How do you deal with stress?*

*When some people are stressed or when some people want to cope with problems or stress, they may resort to drinking a lot of alcohol. You had shared with the team earlier that you consume alcohol, so a few of my questions may seem repetitive. When was the last time…. how often….how much…who with…what makes you want to drink…on what occasions do you drink…*

**Step 3. Elicit attitudes and perceptions about drinking.**

Practitioner gently probes participant’s attitudes towards alcohol, their perceptions about drinking, and what they experience when they drink.

Talking points:

*I would now like to know a little more about what you think about alcohol and consuming it. What kind of effects does it have on you…how do you feel when you drink…how do you feel about your drinking habit/behavior…what do you think about other people drinking…what is your understanding of drinking, tolerance, addiction…what are your thoughts on whether alcohol is a drug or not…have you heard of alcoholism? What are your thoughts on alcoholics…*

**Step 4. Psychoeducation.**

The practitioner provides information regarding the impact of alcohol on one’s physical health, psychological wellbeing (immediately and in the long run), impact on financial stability, social relationships, work and productivity.

Talking points:

*What is alcohol?* Alcohol is a psychoactive substance – produces changes in the chemicals our brain produces and affects our body and mind – it has dependence-producing properties i.e. person who consumes a lot of it regularly can get “addicted” or dependent, have intense craving, tolerance (more of it needs to be consumed to have the same effects), withdrawal (stoppage/reduction of consumptions produces effects in the body and mind such as tremors, sleeplessness, anxiety, headaches, racing heart, breathlessness, irritability, sweating and in severe cases, seizures (fits) or even death; they may give up more and more of their everyday life activities to just procure and consume alcohol or to recover from its effects, lose control over how much they drink, and continue to use it despite knowing how harmful it is.

*What are its effects on the body?* Long term heavy alcohol use can lead to painful and fatal diseases such as liver cirrhosis, heart disease and stroke, and cancer of the liver, mouth, throat, colon, etc. It is a big risk factor for early onset dementia.

*Psychological harm.* Depression, anxiety, exacerbation of already existing mental disorders. Emotional unrest, disinhibited behaviors, anger and rage, etc.

Practitioner may use the WHO handout “Effects of High-Risk Drinking” to demonstrate the topic.

*Violence and injuries.* Road traffic accidents, physical fights and brawls leading to traumatic head injury, self-directed violence, and domestic violence.

*Social harms.* Spousal conflict, family breakups, long lasting negative impact on children, unemployment, bankruptcy, significant financial hardship – poor savings for self and family as money gets diverted into purchase of alcohol.


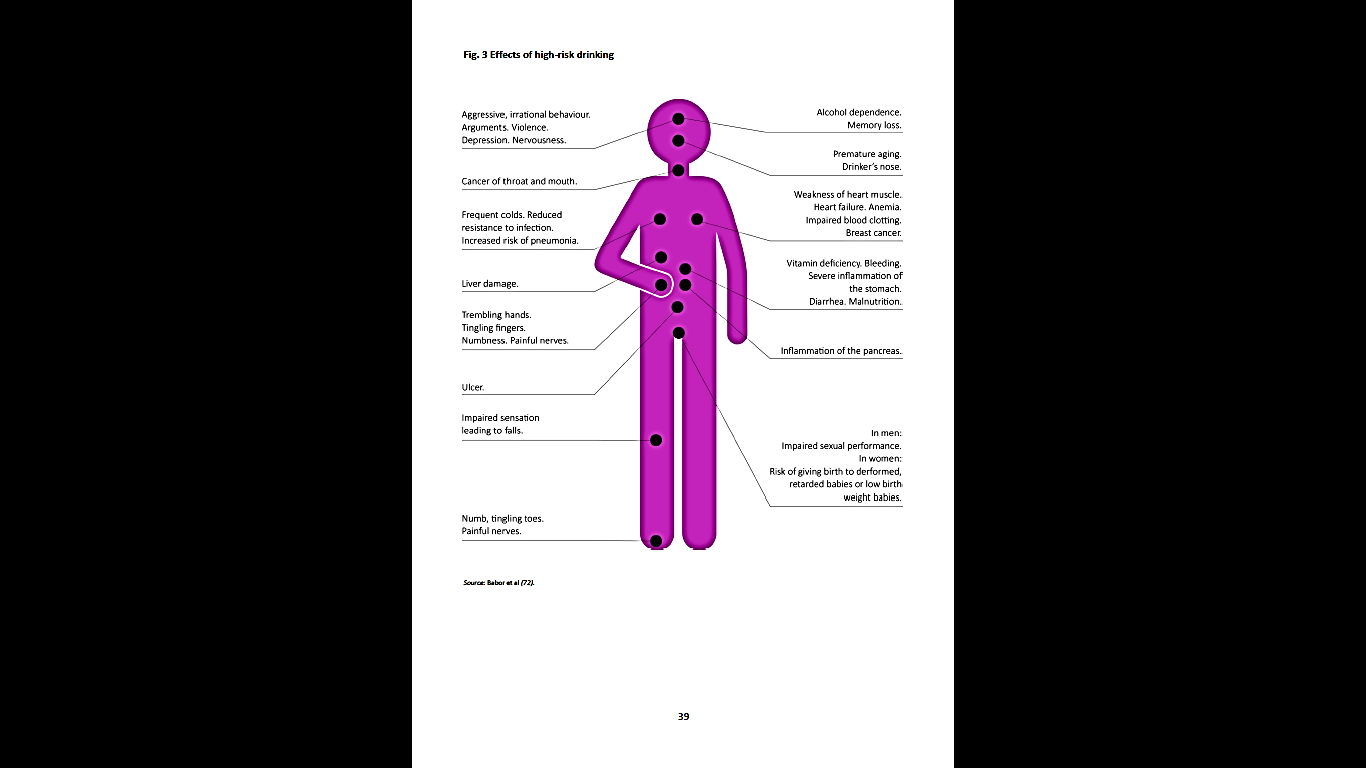


**Step 5. Dispel myths and misconceptions.**

The practitioner may pick up on certain things mentioned by the participant in Step 3, and dispel inaccurate notions and assumptions about alcohol and drinking. The practitioner may also state myths and misconceptions not expressed by the participant himself by saying something along the lines of “several people who drink heavily have told us they think that…” or “people have varied thoughts and ideas about alcohol and drinking such as….”.

- *Alcohol is not a drug...* Alcohol is a drug in the sense that it alters the functioning of the organism, particularly the central nervous system on which thoughts, emotions and behavior depend. It can also cause dependence.
- *Having high alcohol tolerance means that the person will not become an alcoholic...* The truth is exactly the opposite; high tolerance means that the brain is becoming accustomed to the drug.
- *Mixing drinks makes you drunk....* What really gets one drunk is the quantity of alcohol and the speed that one drinks.
- *Beer does not make you drunk...* In the case of beer, the absorption of alcohol through the stomach is a little slower, but depending on the quantity consumed, it does cause drunkenness.
- *Alcohol is sexually stimulating...* Initially alcohol can reduce inhibitions and help people to become more outgoing, but since alcohol has a depressant effect on the nervous system it ends up reducing these sensations and can hamper sexual relations. Alcohol use is one of the most frequent causes of erectile dysfunction (impotence).
- *Alcoholism is an illness that affects older adults...* A majority of alcohol dependent persons are young men of working age.
- *Alcoholics are those that drink daily...* A majority of alcohol-dependent people, in the initial and intermediate stage of the process, drink mainly on the weekend, and continue with their normal school and work activities, but with increasing difficulty.
- *Having a coffee or washing your face with cold water reduces the effects of alcohol...* The only thing that really reduces drunkenness is the gradual elimination of alcohol from the organism, which means forcing the liver to work, which takes time.
- *Alcohol is good for making friends...* Alcohol creates complicity around drinking, but true friendship includes much more than that.
- *Parties are not parties without alcohol...* The media often tries to convince us that parties or celebrations need alcohol, and that alcohol must be at the center of every social gathering. But is this true? What makes a social gathering or a party – the alcohol or the people?

**Step 6 – Refer to NIMHANS.**

The practitioner recommends that the participant seek additional medical support for reducing alcohol consumption by visiting the Centre for Addiction Medicine OPD at NIMHANS. The practitioner may take the participant through the steps of availing psychiatric support at NIMHANS and address concerns, if any, about seeking help at a tertiary care mental healthcare institution.

**Appendix AB**

**NIMHANS Legal Aid/Sakhi/APSA Referral Letter**

**Project Harmony**

To,

ID No:

Date:

Dear ______________,

Project Harmony is a research study at St John's Research Institute, Bengaluru approved by the Government of India. This study implements and evaluates a community level psychosocial intervention that targets alcohol use disorder in men and intimate partner violence in couples residing under the jurisdiction of the ____________________ Primary Health Centre.

Mrs. ______________, is a participant in the study/is ineligible to participate in our study. Ms. _______________ reported to us that she has experienced intimate partner violence on at least one occasion in the past.

We are hereby referring Mrs. ________ to you for facilitating the provision of legal counsel, medico-legal resources, shelters, protection and/or whichever is deemed necessary and caters to the needs of the woman.

Thank you.

Sincerely,

Dr. K. Srinivasan

Main Principal Investigator

Project Harmony

St. John’s Research Institute

St. John’s Medical College and Hospital

Bengaluru, Karnataka - 560034

**Appendix AC**

***Handling Difficult Situations during the intervention***

Counselors may encounter challenging situations during the sessions. Some strategies for handling such challenging situations are recommended below. These are not “hard and fast rules”, counselors must rely on their cultural competence in dealing with different situations and must consult with the supervisor and project concerned staff when in a dilemma.

***1. If a session is rescheduled or cancelled***

The sessions must be conducted in the order provided. If a session is cancelled or rescheduled, continue with the missed session. Do not skip sessions.

***2. Couple altercation escalates to an extreme argument***

**Step i:** Ask the couple to take a time-out/break for 2-3 minutes while you sit in the room together.

**Step ii:** While maintaining your composure, gently state why a break was necessary to the couple. If one person was louder than the other and/or interrupting/yelling at the other, you could say,

*“I noticed that you were raising your voice and you interrupted your partner. You were using threatening words and that does not allow for both of you to communicate and discuss safely in the session. We may continue only after I know that you are feeling more in control right now. If you disagree, or something upsets you, please wait for me or your partner to stop talking and only then tell me what it is that upsets you, without raising your voice. Only then can I help you address your concerns.”*

*“ನಿಮ್ಮ ಸ್ವರ ತುಂಬಾ ಜೋರಾಗಿದ್ದು ಮತ್ತು ನೀವು ನಿಮ್ಮ ಗಂಡ/ಹೆಂಡತಿ ಮಾತನಾಡುತ್ತಿರುವಾಗ ಅಡ್ಡ ಬಂದಿದ್ದು ನನಗೆ ಕಂಡಿತು. ನೀವು ಬೆದರಿಕೆಯ ಮಾತುಗಳನ್ನು ಹೇಳುತ್ತಾ ಇದ್ದೀರಿ, ಅದರ ಕಾರಣ ನಿಮ್ಮಿಬ್ಬರಿಗೂ ಸುರಕ್ಷಿತವಾಗಿ ಮಾತನಾದಕ್ಕೆ ಮತ್ತು ಶಾಂತಿಯಲ್ಲಿ ಚರ್ಚೆ ಮಾಡಕ್ಕೆ ಆಗುತ್ತಿಲ್ಲ. ಈ ಸಭೆ ಮುಂದುವರಿಯಬೇಕೆಂದರೆ ನೀವು ಮೊದಲಾಗಿ ಭಾವನೆಗಳನ್ನು ನಿಯಂತ್ರಣದಲ್ಲಿ ತರಬೇಕು. ನಿಮಗೆ ನಾನು ಅಥವಾ ನಿಮ್ಮ ಗಂಡ/ಹೆಂಡತಿ ಹೇಳುವುದು ಒಪ್ಪಿಕೇ ಇಲ್ಲದಿದ್ದರೆ ಅಥವಾ ಬೇಜಾರಾದರೆ, ನಾನು ಅಥವಾ ನಿಮ ಗಂಡ/ಹೆಂಡತಿ ಮಾತನಾಡಿ ಮುಗಿಸಿದ ಮೇಲೇನೇ ನಿಮಗೆ ಏನು ಅನಿಸುತ್ತಿದೆ ಎಂದು ಮೆದು ಸ್ವರದಲ್ಲಿ ಹೇಳಿ. ಹಾಗಿದ್ದರೇನೇ ನನಗೆ ನಿಮ್ಮ ಅಭಿಪ್ರಾಯಗಳನ್ನು ಅರ್ಥ ಮಾಡಿಕೊಂಡು ಸಹಾಯ ಮಾಡಕ್ಕೆ ಆಗತ್ತೆ.”*

*“Nim'ma svara tumbā jōrāgiddu mattu nīvu nim'ma gaṇḍa/heṇḍati mātanāḍuttiruvāga aḍḍa bandiddu nanage kaṇḍitu. Nīvu bedarikeya mātugaḷannu hēḷuttā iddīri, adara kāraṇa nim'mibbarigū surakṣitavāgi mātanādakke mattu śāntiyalli carce māḍakke āguttilla. Ī sabhe munduvariyabēkendare nīvu modalāgi bhāvanegaḷannu niyantraṇadalli tarabēku. Nimage nānu athavā nim'ma gaṇḍa/heṇḍati hēḷuvudu oppikē illadiddare athavā bējārādare, Nānu athavā nima gaṇḍa/heṇḍati mātanāḍi mugisida mēlēnē nimage ēnu anisuttide endu medu svaradalli hēḷi. Hāgiddarēnē nanage nim'ma abhiprāyagaḷannu artha māḍikoṇḍu sahāya māḍakke āgatte.”*

**Step ii:** If you still feel out of control of the situation after the break or the couple refuses to sit in

silence for 10 minutes, seek help from another staff member if possible and inform the couple that you will have to end the session if they continue arguing in an unproductive way.

**Step iii:** Dissolve the session and clearly state that the couple should try again to take a longer break and must not continue the argument when they return home. If there are circumstances at home either partner thinks may re-open this argument/lead to an altercation, delineate the “Catch Yourself!” and “Time-out/Break” steps to help them regulate their emotions and de-escalate the situation. (Refer to CATCH YOURSELF! and TIME-OUTS/BREAKS from Session 2 of BCT).

**Step iv:** Discuss with the supervisor next steps for the couples’ involvement in the study and identify ways to prevent escalation of arguments in the future.

**Step v:** If conflict or arguments or level of violence or abuse escalates, refer the couple to external agencies for support and guidance. (Refer to the Referral List for participants)

***3. If one participant shows up drunk to the couples’ session***

**Step i:** Talk to the sober partner individually.

Ask: *“What happened?” “Why is your partner drunk?”*

*“ಏನಾಯಿತು?”, “ನಿಮ್ಮ ಗಂಡ ಕುಡಿದುಕೊಂಡು ಯಾಕೆ ಬಂದಿದ್ದಾರೆ?”*

*“Ēnāyitu? Nim'ma gaṇḍa kuḍidukoṇḍu yāke bandiddāre?”*

**Step ii:** Reschedule the session with the sober partner and ask them to attend the next session.

**Step iii:** Make 3 attempts to call the couple before the rescheduled session to:

(a) Remind the participant who was drunk that they must be sober to attend the sessions and showing up drunk is unacceptable.

(b) Remind both participants of their rescheduled session.

**Step iv:** Inform and discuss the situation with your supervisor in the supervision session – the subsequent BCT session can start with a brief revisit and review of MI content on change, avoiding triggers, managing difficult emotions, managing urges, drink refusal skills, lapse management and relapse prevention.

**Step v:** If it happens repeatedly (more than once), contact the supervisor and project manager to inform them and take appropriate action.

***4. If both participants show up to a couples’ session drunk***

**Step i:** Dissolve the session and send the couple home safely.

**Step ii:** Make 3 attempts to call the couple to:

(a) Remind both participants that they must be sober to attend the sessions and showing up drunk is unacceptable.

(b) Reschedule the missed session.

**Step iii:** Call the couple to remind them of their rescheduled session a day prior.

**Step iv:** Inform and discuss the situation with your supervisor in the supervision session.

**Step v:** If it happens repeatedly (more than once), contact the supervisor and project manager to inform them and take appropriate action.

***5. If one partner stops attending the couple sessions***

**Step i:** Ask: *“Where is your partner?” “Why haven’t they shown up?” “How can we help them to attend?”*

*"ನಿಮ್ಮ ಗಂಡ/ಹೆಂಡತಿ ಎಲ್ಲಿದ್ದಾರೆ?", "ಅವರು ಬಂದಿಲ್ಲ ಯಾಕೆ?", "ಅವರು ಈ ಸಭೆಗಳಿಗೆ ಬರುವ ಹಾಗೆ ನಾವು ಏನು ಮಾಡಬಹುದು?"*

*"Nim'ma gaṇḍa/heṇḍati elliddāre?", "Avaru bandilla yāke?", "Avaru ī sabhegaḷige baruva hāge nāvu ēnu māḍabahudu?"*

**Step ii:** Reschedule the session by speaking to both partners over the phone.

**Step iii:** Call the couple to remind them of their rescheduled session a day prior.

**Step iv:** Inform and discuss the situation with your supervisor at the supervision session.

**Step v:** Revisit and review couple session guidelines in the rescheduled session.

**Step vi:** If it happens repeatedly (more than once), contact the supervisor and project manager to inform them and take appropriate action.

***6. If both partners stop showing up to couples’ sessions***

**Step i:** Make 3 attempts to call the couple, preferably speaking to each individually.

1. Ask: *“Why did you miss the couples’ session?” “What might help you attend?”*

*"ದಂಪತಿಗಳ ಸಭೆಗೆ ನೀವು ಯಾಕೆ ಬಂದಿಲ್ಲ?", "ನೀವು ಬರುವ ಹಾಗೆ ನಾವು ಏನು ಮಾಡಬಹುದೆಂದೇ ನನಗೆ ಹೇಳುತ್ತೀರಾ?"*

*"Dampatigaḷa sabhege nīvu yāke bandilla?", "Nīvu baruva hāge nāvu ēnu māḍabahudendē nanage hēḷuttīrā?"*

(b) Reschedule the missed session.

**Step ii:** If the couple does not answer the attempted calls, inform the intervention coordinator, and project manager [to arrange for an ASHA home visit].

**Step iii:** Call the couple to remind them of their rescheduled session a day prior.

**Step iv:** Inform and discuss the situation with your supervisor at the supervision session.

**Step v:** If it happens repeatedly (more than once), contact the supervisor and project manager to inform them and take appropriate action.

***7. Couples don’t understand or misunderstand the intention of the activities***

**Step i:** Re-explain the activity and the rationale for the activity to the couple using different words or examples, if needed.

**Step ii:** Invite each of them to re-state the activity and the rationale in their own words, how they have understood it.

**Step iii:** Ask each partner if they have any questions or doubts. Give them some time (2-3 minutes) to think it over and respond.

**Step iv:** Discuss the situation with your supervisor to find a solution to handle it at the next session.

***8. Individual (during MI #1-3) or couple (rest of Harmony intervention) does not fill out the calendar or does not do home practice activities.***

**Step i:** Ask: “*May I know why you have not been able to _____ [cannot read/write, mark the calendar/practice breathing exercises/other activity]?”.*

*ಈ ಮನೆನಲ್ಲಿ ಮಾಡುವ ಚಟುವಟಿಕೆಯನ್ನು ನೀವು ಯಾಕೆ ಮಾಡಲಿಲ್ಲ ಎಂದು ನನಗೆ ಹೇಳುತ್ತೀರಾ?*

*Ī manenalli māḍuva caṭuvaṭikeyannu nīvu yāke māḍalilla endu nanage hēḷuttīrā?*

**Step ii:** Listen to each partner as they describe their challenges in completing the home practice activity [no time, tired, no privacy, don’t feel like doing it, not useful, slipped my mind, etc.].

**Step iii:** Troubleshoot together to find ways to help them fill out the materials, providing examples of potential problems and solutions.

- *Cannot read/write:* Assess participants’ literacy levels to rule out the inability to complete activities due to low literacy/illiteracy. In case of poor/illiteracy, re-explain concepts with the aid of the pictorial handouts, demonstrate how to tick-mark the calendar (which does not require functional literacy), and encourage participants to recall and retell the tasks at the end of the session.
- *No time*: “Each practice activity takes at most 2-5 minutes of your time. You could do it any time of the day – such as the daily trust discussion. You don’t have to do it for more than 5 minutes on any day. What is the time of the day when both of you might be able to find these 5 minutes?”

"ಪ್ರತಿ ಒಂದು ಚಟುವಟಿಕೆ ಮಾಡಕ್ಕೆ ಎರಡು ಅಥವಾ ಐದು ನಿಮಿಷಕ್ಕಿಂತ ಜಾಸ್ತಿ ಸಮಯ ಬೇಕಾಗಲ್ಲ. ಒಂದು ದಿನದಲ್ಲಿ ನೀವು ಯಾವಾಗ ಬೇಕಾದರೂ ಮಾಡಬಹುದು - ದೈನಂದಿನದ ನಂಬಿಕೆ ಒಪ್ಪಂದ ತರ. ಐದು ನಿಮಿಷ ಕಿಂತ ಜಾಸ್ತಿ ಮಾಡಬೇಕಂತ ಇಲ್ಲ. ಒಂದು ದಿನದಲ್ಲಿ ನೀವಿಬ್ಬರೂ ಯಾವ ಸಮಯಕ್ಕೆ ಈ ಐದು ನಿಮಿಷದ ಚಟುವಟಿಕೆ ಮಾಡಬಹುದೆಂದು ಮಾತನಾಡಿ ನಿರ್ಧಾರಿಸುವನಾ?"

"Prati ondu caṭuvaṭike māḍakke eraḍu athavā aidu nimiṣakkinta jāsti samaya bēkāgalla. Ondu dinadalli nīvu yāvāga bēkādarū māḍabahudu - dainandinada nambike oppanda tara. Aidu nimiṣa kinta jāsti māḍabēkanta illa. Ondu dinadalli nīvibbarū yāva samayakke ī aidu nimiṣada caṭuvaṭike māḍabahudendu mātanāḍi nirdhārisuvanā?"

- *Tired:* “Like we have discussed in the first couple session – you change how you act/behave first and then you will see changes in how you feel about your partner and the relationship, and not the other way around. For that, it is important to take 5 minutes out in the day to do this activity, even if you feel tired. After 5 minutes of doing this, you can both take a break and rest, or get back to your routine. The more regularly you do this, it will become a part of your daily routine, so you might not feel tired to carry it out and it might come more easily/naturally to you.”

"ನಾವು ಮೊದಲನೇ ದಂಪತಿಗಳ ಸಭೆಯಲ್ಲಿ ಹೇಳಿದಹಾಗೆ, ನಿಮ್ಮ ವರ್ತನೆ ಅಥವಾ ನಡವಳಿಕೆಯನ್ನು ಬದಲಾಯಿಸಿದಮೇಲೆನೇ ನಿಮ್ಮ ಭಾವನೆಗಳಲ್ಲಿ ಬದಲಾವಣೆ ಕಂಡು ಬರತ್ತೆ. ಅದ್ಕಕಾಗಿನೀ ದಿನಾ ಐದು ನಿಮಿಷ ಈ ಚಟುವಟಿಕೆಯನ್ನು ಮಾಡಕ್ಕ ಮನಸ್ಸು ಮಾಡಿ, ನಿಮಗೆ ಸುಸ್ತಾಗಿದ್ದರೂ ಸಹ. ಐದು ನಿಮಿಷ ಇದನ್ನ ಮಾಡಿದ ಮೇಲೆ ಬೇಕಾದರೆ ನೀವು ಆರಾಮ ಮಾಡಬಹುದು ಅಥವಾ ನಿಮ್ಮ ದಿನಚರಿಯ ಕೆಲಸಕ್ಕೆ ಮಾಡೋದ್ದು ಶುರು ಮಾಡಬಹುದು, ನಿಮಗೆ ಬಿಟ್ಟಿದ್ದು. ಎಷ್ಟು ನಿಯಮಿತವಾಗಿ ನೀವು ಇವೆಲ್ಲವನ್ನೂ ಮಾಡುತ್ತೀರಿ, ಅಷ್ಟೇ ಅದು ನಿಮ್ಮ ದಿನಚರಿಯ ಒಂದು ಅಂಶ ಆಗಿ ಹೋಗತ್ತೆ, ಮತ್ತು ನಿಮಗೆ ಮಾಡಕ್ಕೆ ಸುಸ್ತಾಗಲ್ಲ. ಅದು ನಿಮಗೆ ಸುಲಭವಾಗಿ ಮಾಡಕ್ಕೆ ಸಾಧ್ಯವಾಗುತ್ತದೆ."

"Nāvu modalanē dampatigaḷa sabheyalli hēḷidahāge, nim'ma vartane athavā naḍavaḷikeyannu badalāyisidamēlenē nim'ma bhāvanegaḷalli badalāvaṇe kaṇḍu baratte. Adkakāginī dinā aidu nimiṣa ī caṭuvaṭikeyannu māḍakka manas'su māḍi, nimage sustāgiddarū saha. Aidu nimiṣa idanna māḍida mēle bēkādare nīvu ārāma māḍabahudu athavā nim'ma dinacariya kelasakke māḍōddu śuru māḍabahudu, nimage biṭṭiddu. Eṣṭu niyamitavāgi nīvu ivellavannū māḍuttīri, aṣṭē adu nim'ma dinacariya ondu anśa āgi hōgatte, mattu nimage māḍakke sustāgalla. Adu nimage sulabhavāgi māḍakke sādhyavāguttade."

- *Not useful*: “Like we had talked about earlier, this activity is a skill, just like riding a bicycle. In the beginning it is difficult and seems to take a lot of effort and time and does not really appear useful. But the more you practice it, the more easily it comes to you, and starts becoming useful too! When you are struggling to ride a bicycle, walking on your feet may seem like a better option. But once you master riding a bicycle, it comes naturally to you, and you can travel to places much faster, saving time and energy. Just like that, you might experience the benefits of doing this activity only after you start doing it regularly.”

“ನಾವು ಈ ಹಿಂದ ಮಾತನಾಡಿಕೊಂಡಹಾಗೆ, ಈ ಚಟುವಟಿಕೆ ಒಂದು ಸ್ಕಿಲ್. ಸೈಕಲ್ ಓಡಿಸಿದ ಹಾಗೆ. ಫಸ್ಟ್-ಫಸ್ಟ್ ಗೆ ಸೈಕಲ್ ಓಡಿಸಕ್ಕೆ ಕಷ್ಟ ಆಗತ್ತೆ, ಬಹಳ ಸಮಯ ಹಿಡಿಯತ್ತೆ, ಮತ್ತು ಅಷ್ಟು ಉಪಯುಕ್ತ ಎಂದು ಅನಿಸಲ್ಲ. ಆದರೆ ಇನ್ನು ಜಾಸ್ತಿ ಅಭ್ಯಾಸ ಮಾಡುತ್ತಾ, ಅದು ಸುಲಭವಾಗತ್ತೆ, ಮತ್ತು ಅಷ್ಟೇ ಉಪಯುಕ್ತ ಸಹ ಆಗತ್ತೆ. ಸೈಕಲ್ ಓಡಿಸಕ್ಕೆ ಕಲಿತಿರುವಾಗ ನಡೆದುಕೊಂಡೇ ಹೋಗುವುದು ವೊಳ್ಳೆಯದು ಅಂತ ಅನಿಸತ್ತ. ಆದರೆ, ಸೈಕಲ್ ಓಡಿಸಕ್ಕೆ ಕಲಿತ ಮೇಲೆ, ಅದು ಸುಲಭ ಅನಿಸುತ್ತದೆ, ಮತ್ತು ದೂರ ಜಾಗಗಳಿಗೆ ಬೇಗ ಹೋಗಕ್ಕಾಗತ್ತೆ. ಸಮಯ ಉಳಿತಾಯ ಮಾಡುತ್ತೇವೆ. ಹೀಗೇನೆ, ಈ ಚಟುವಟಿಕೆ ನಿಯಮಿತವಾಗಿ ಅಭ್ಯಾಸ ಮಾಡುತ್ತಾ ನಿಮಗೆ ಇದರ ಲಾಭಗಳ ಅನುಭವ ಸಿಗತ್ತೆ.”

Nāvu ī hinda mātanāḍikoṇḍahāge, ī caṭuvaṭike ondu skil. Saikal ōḍisida hāge. Phasṭ-phasṭ ge saikal ōḍisakke kaṣṭa āgatte, bahaḷa samaya hiḍiyatte, mattu aṣṭu upayukta endu anisalla. Ādare innu jāsti abhyāsa māḍuttā, adu sulabhavāgatte, mattu aṣṭē upayukta saha āgatte. Saikal ōḍisakke kalitiruvāga naḍedukoṇḍē hōguvudu voḷḷeyadu anta anisatta. Ādare, saikal ōḍisakke kalita mēle, adu sulabha anisuttade, mattu dūra jāgagaḷige bēga hōgakkāgatte. Samaya uḷitāya māḍuttēve. Hīgēne, ī caṭuvaṭike niyamitavāgi abhyāsa māḍuttā nimage idara lābhagaḷa anubhava sigatte.

- *Slipped my mind/forgot:* “I understand that we are discussing a lot of different techniques and activities in the sessions. It is quite difficult to remember all of them all the time. For that reason, I have given you this sheet of paper which has pictures of the activities you need to practice at home regularly. If either one of you forgets, gently remind your partner of the home activity, and help each other to remember in this manner. At the end of every session, it would be helpful to listen carefully when we summarize all the activities to be done at home.”

ನಾವು ಹಲವಾರು ವಿಧಾನಗಳು ಮತ್ತು ಚಟುವಟಿಕೆಗಳನ್ನು ಈ ಸಭೆಗಳಲ್ಲಿ ಚರ್ಚಿಸುತ್ತಿದ್ದೇವೆ ಎಂದು ನನಗೆ ತಿಳಿದಿದೆ. ಎಲ್ಲಾ ವಿಷಯಗಳನ್ನು ನೆನಪಿನಲ್ಲಿ ಇಟ್ಟುಕೊಳ್ಳಕ್ಕೆ ಕಷ್ಟ ಆಗಬಹುದು. ಅದಕ್ಕಾಗಿ ಈ ಚೀಟಿಯಲ್ಲಿ ಎಲ್ಲ ಚಟುವಟಿಕೆಗಳನ್ನು ಪಟ್ಟಿ ಮಾಡಿದ್ದೇವೆ. ನಿಮ್ಮಿಬ್ಬರಲ್ಲಿ ಒಬ್ಬರು ಮರೆತು ಹೋದರೆ, ಚನ್ನಾಗಿ ನಿಮ್ಮ ಗಂಡ/ಹೆಂಡತಿಗೆ ನೆನಪು ಮಾಡಿ. ಈ ರೀತಿಯಲ್ಲಿ ಸಹಾಯ ಮಾಡಿ. ಸಭೆಯ ಕೊನೆಗೆ ನಾನು ಪ್ರತಿಸಲ ಏನೆಲ್ಲಾ ಮಾತನಾಡಿಕೊನಿದ್ದೇವೆ ಮತ್ತು ಮನೆಯಲ್ಲಿ ನೀವು ಏನು ಅಭ್ಯಾಸಮಾಡಬೇಕೆಂದು ಹೇಳುತ್ತಿರುವಾಗ ಗಮನು ಕೊಟ್ಟು ಕೇಳಿಸಿಕೊಂಡರೆ ನೆನಪಿಟ್ಟು ಅಭ್ಯಾಸ ಮಾಡಕ್ಕೆ ಸಹಾಯವಾಗುತ್ತದೆ.

Nāvu halavāru vidhānagaḷu mattu caṭuvaṭikegaḷannu ī sabhegaḷalli carcisuttiddēve endu nanage tiḷidide. Ellā viṣayagaḷannu nenapinalli iṭṭukoḷḷakke kaṣṭa āgabahudu. Adakkāgi ī cīṭiyalli ella caṭuvaṭikegaḷannu paṭṭi māḍiddēve. Nim'mibbaralli obbaru maretu hōdare, cannāgi nim'ma gaṇḍa/heṇḍatige nenapu māḍi. Ī rītiyalli sahāya māḍi. Ī rītiyalli sahāya māḍi. Sabheya konege nānu pratisala ēnellā mātanāḍikoniddēve mattu maneyalli nīvu ēnu abhyāsamāḍabēkendu hēḷuttiruvāga gamanu koṭṭu kēḷisikoṇḍare nenapiṭṭu abhyāsa māḍakke sahāyavāguttade.

**Step iii:** Remind them that all the activities done at home are just as important as the sessions themselves.

***9. One or both partners participate in a perfunctory or superficial manner***

**Step i:** Try to speak to them and attempt to engage them by asking them to respond to questions and asking them about what they think. For example, while introducing taking turns in Good Communication, you may ask them, “What are your thoughts on why taking turns is important?”/ “If someone does not take turn and keeps interrupting and talking over you, how do you think you might feel?” [similar questions can be asked with any of the BCT topics during the session when you notice that one or both partners are not attentive or participating in a superficial manner].

**Step ii:** Gently probe if they are feeling disconnected to the sessions and topics being discussed. Encourage them not to hesitate to bring up matters of disinterest/boredom with you by stating that you value their feedback.

*“Please feel free to tell me if you feel like I’m going too fast, or that you do not understand something. It is important for me to know what you are thinking and feeling, because only then will I know if what I am saying is actually reaching you. And if it isn’t, then we can discuss what it is that is coming in the way or making you lose interest in the sessions.”*

*“ನಾನು ತುಂಬಾ ಬೇಗ ಮಾತನಾಡುತ್ತಿದ್ದರೆ, ಅಥವಾ ನಿಮಗೆ ನಾನು ಏನಾದರೂ ಹೇಳಿದ್ದು ಅರ್ಥಾಗುತ್ತಿಲ್ಲ ಎಂದು ಅನಿಸಿದ್ದರೆ, ದಯವಿಟ್ಟು ನನಗೆ ಹೇಳಿ. ನಿಮಗೆ ಏನು ಅನಿಸುತ್ತಿದೆ ಮತ್ತು ನೀವು ಏನು ಯೋಚಿಸುತ್ತಿದ್ದೀರಿ ಎಂದು ನನಗೆ ನೀವು ಹೇಳುದು ತುಂಬಾ ಮುಖ್ಯ, ಏಕೆಂದರೆ ನೀವು ನನಗೆ ತಿಳಿಸಿದ್ದರೇನೇ ನಾನು ಹೇಳಿದ ಮಾತು ನಿಮ್ಮ ವರೆಗೆ ತಲುಪುತ್ತಿದೆಯಾ ಇಲ್ಲವ ಎಂದು ನನಗೆ ಗೊತ್ತಾಗತ್ತೆ. ನಿಮಗೆ ಅರ್ಥಾಗುತ್ತಿಲ್ಲ ಎಂದರೆ, ನಿಮಗೆ ಅರ್ಥಮಾಡಿಕೊಳ್ಳಕ್ಕೆ ಏನು ಅಡ್ಡ ಬರುತ್ತಿದೆ ಅಥವಾ ಕಷ್ಟ ಆಗುತ್ತಿದೆ ಅಥವಾ ಸಭೆಯಲ್ಲಿ ಮನಸ್ಸಿಲ್ಲದ ಹಾಗೆ ಮಾಡುತ್ತದೆ ಎಂದು ಚರ್ಚಿಸಿ ಸುಧಾರಿಸಕ್ಕಾಗತ್ತೆ.”*

*Nānu tumbā bēga mātanāḍuttiddare, athavā nimage nānu ēnādarū hēḷiddu arthāguttilla endu anisiddare, dayaviṭṭu nanage hēḷi. Nimage ēnu anisuttide mattu nīvu ēnu yōcisuttiddīri endu nanage nīvu hēḷudu tumbā mukhya, ēkendare nīvu nanage tiḷisiddarēnē nānu hēḷida mātu nim'ma varege taluputtideyā illava endu nanage gottāgatte. Nimage arthāguttilla endare, nimage arthamāḍikoḷḷakke ēnu aḍḍa baruttide athavā kaṣṭa āguttide athavā sabheyalli manas'sillada hāge māḍuttade endu carcisi sudhārisakkāgatte.*

**Step iii:** Remind them that active participation is necessary for the session to be productive. Explain to them that although talking about these matters can be uncomfortable, they can try to get out of their comfort zone. Ask why they feel uncomfortable and try to troubleshoot.

**Step iv:** Continue with the session.

***10. Couple blames the counselor for issues in their relationship***

**Step i:** First, recognize and understand that blaming is a normal, although unhealthy, coping mechanism among people. Hence, do *not* react immediately to their comments and concerns.

**Step ii:** Listen carefully to what they are saying. Verbally and nonverbally acknowledge their comments.

*“What I understand from what you’re saying right now is that you feel the counseling sessions are making matters worse for you and your partner by bringing up issues that do not seem to be a problem to you. Thank you for telling me how you feel, because only if you share these feelings with me will I be able to work with you to make it better for you and your partner”.*

*“ನೀವು ಇವಾಗ ಹೇಳುತ್ತಿರುವುದು ಏನೆಂದರೆ ನಿಮ್ಮ ಸಂಬಂಧದಲ್ಲಿಲ್ಲದ ಸಮಸ್ಯೆಗಳನ್ನು ಉಂಟುಮಾಡಿ ಈ ಕೌನ್ಸೆಲಿಂಗ್/ಸಮಾಲೋಚನೆಯ ಸಭೆಗಳು ನಿಮ್ಮ ಸಂಬಂಧವನ್ನು ಇನ್ನೂ ಹಾಳು ಮಾಡುತ್ತಿದೆ. ನಿಮಗೆ ಈ ವಿಷಯಗಳು ಸಮಸ್ಯೆಗಳ ತರ ಅನಿಸುತ್ತಿಲ್ಲ. ನಿಮಗೆ ಏನು ಅನಿಸುತ್ತಿದೆ ಎಂದು ನನಗೆ ನಿಜವಾಗಿ ಹೇಳಿದಕ್ಕೆ ಧನ್ಯವಾದಗಳು. ನೀವು ಈ ಮನಸಿನ ಮಾತುಗಳು ನನಗೆ ಹೇಳಿದ್ದಾರೆ ಮಾತ್ರ ನನಗೆ ನಿಮ್ಮ ಮತ್ತು ನಿಮ್ಮ ಗಂಡ/ಹೆಂಡತಿಯ ಪರಿಸ್ಥಿತಿಯನ್ನು ಉತ್ತಮಗೊಳ್ಳಿಸಕ್ಕೆ ಪ್ರಯತ್ನ ಮಾಡಕ್ಕೆ ಆಗತ್ತೆ.”*

*“Nīvu ivāga hēḷuttiruvudu ēnendare nim'ma sambandhadallillada samasyegaḷannu uṇṭumāḍi ī kaunseliṅg/samālōcaneya sabhegaḷu nim'ma sambandhavannu innū hāḷu māḍuttide. Nimage ī viṣayagaḷu samasyegaḷa tara anisuttilla. Nimage ēnu anisuttide endu nanage nijavāgi hēḷidakke dhan'yavādagaḷu. Nīvu ī manasina mātugaḷu nanage hēḷiddāre mātra nanage nim'ma mattu nim'ma gaṇḍa/heṇḍatiya paristhitiyannu uttamagoḷḷisakke prayatna māḍakke āgatte.”*

**Step ii:** Ask to discuss the specific issues related to the concern.

*“What exactly about the sessions do you feel is a problem/becoming a problem/that you dislike?”*

*“ಈ ಸಭೆಗಳ ಬಗ್ಗೆ ನಿಮಗೆ ಏನು ಇಷ್ಟಾಗಲ್ಲ/ಸಭೆಗಳಲ್ಲಿ ಏನು ಸಮಸ್ಯೆಗಳಿವೆ/ಸಭೆಗಳಿಂದ ಏನು ಸಮಸ್ಯೆ ಉಂಟಾಗುತ್ತಿದೆ ಎಂದು ನಿಮಗೆ ಅನಿಸುತ್ತದೆ?”*

*“Ī sabhegaḷa bagge nimage ēnu iṣṭāgalla/sabhegaḷalli ēnu samasyegaḷive/sabhegaḷinda ēnu samasye uṇṭāguttide endu nimage anisuttade?”*

**Step iii.:** Address specific concerns with the couple, acknowledging that it’s natural for counselling around new communication patterns to raise issues among the couple, but that it is not creating issues. It is rather raising existing issues to the surface and that by addressing these underlying issues, the couple will hopefully be able to improve their relationship in the long-term.

**Step iv:** If you are unable to resolve the couple’s concerns, discuss with your supervisor after the session in supervision.

***11. Couple or one partner wants to withdraw from the study***

**Step i:** Try to talk to them and understand why they wish to withdraw.

*“May I know why you would like to withdraw from the study? I would like to know what your challenges and concerns are, to see if we could help you with them”.*

*"ಈ ಯೋಜನೆಯನ್ನು ಯಾಕೆ ಬಿಡಬೇಕು ಎಂದು ನಿಮಗೆ ಅನಿಸುತ್ತದೆ? ನಿಮಗೆ ಏನು ಕಷ್ಟಗಳು ಇದೆ ಎಂದು ನಾನು ಅರ್ಥಮಾಡಿಕೊಳ್ಳಲು ಬಯಸುತ್ತೇನೆ. ನನಗೆ ಯಾವ ರೀತಿಯಲ್ಲಿ ನಿಮಗೆ ಈ ಕಷ್ಟಗಳನ್ನು ಕಡಿಮೆ ಮಾಡಲು ಸಹಾಯ ಮಾಡಕ್ಕಾಗತ್ತೆ ಎಂದು ತಿಳಿದುಕೊಳ್ಳಕ್ಕೆ."*

*"Ī yōjaneyannu yāke biḍabēku endu nimage anisuttade? Nimage ēnu kaṣṭagaḷu ide endu nānu arthamāḍikoḷḷalu bayasuttēne. Nanage yāva rītiyalli nimage ī kaṣṭagaḷannu kaḍime māḍalu sahāya māḍakkāgatte endu tiḷidukoḷḷakke."*

**Step ii:** Try to engage them in a different way and address their concerns. [no time, costs too much to get here, it’s not helpful]. Revisit couple session guidelines to address all three concerns. Re-emphasize that the sessions are once a week, for 1 hour, for ____ weeks more (depending upon how many sessions are left to go). Reassure them that their travel expenses to and from the counseling and assessment site are covered by the project. Reiterate that change and improvement in relationships and communication patterns take time, effort, and practice. Their continued commitment to attending the sessions must be acknowledged and appreciated, stating that they may reap the benefits of acquiring these new skills over time.

**Step iii.** If you are unable to resolve the couple’s concerns and they still want to withdraw from the study, discuss with your supervisor. Participants may withdraw from the study, as explained to them during the consent procedures. Notify your supervisor (the intervention coordinator) about the couple’s wish to withdraw. Your supervisor will take the couple through the withdrawal process.

***12. Couple or one of the partners expresses concerns regarding the counselor’s age, sex, and expertise (e.g., due to being very young) in helping them.***

**Step i:** Try to talk to them and understand their concerns. Listen and paraphrase their concerns back to them as a means of validating them.

**Step ii** In case of age-related concerns, inform them about your qualifications in the field and extensive training received to conduct the sessions*.*

*“I am a nurse by qualification and have worked a given amount of time in caring for and speaking with patients and their caregivers. I have also undergone training by a team of professionals of varying professional backgrounds and ages, and only after they deemed me ready for carrying out these sessions, I was allowed to start seeing couples like you for counseling. Moreover, I am supervised by a clinical psychologist/counselling professional, with whom I discuss regularly to provide the most suitable counseling services to you possible.”*

*“ನನಗೆ ನರ್ಸಿಂಗ್ ನಲ್ಲಿ ಡಿಗ್ರಿ ಇದೆ; ನಾನು ತುಂಬಾ ಸಮಯ ಪೆಶೆಂಟ್ಸ್ ಮತ್ತು ಅವರನ್ನು ನೋಡಿಕೊಳ್ಳುವವರೊಂದಿಗೆ ಮಾತನಾಡಿ, ಸಹಾಯ ಮಾಡಿ ನನಗೆ ಗೊತ್ತಿದೆ. ತುಂಬ ಕಲಿತ ಡಾಕ್ಟರ್ ಗಳು ಮತ್ತು ಬೇರೆಯವರಿಂದ ತರಬೇತಿ ಪಡೆದುಕೊಂಡು ಮತ್ತು ನಿಮ್ಮ ತರ ದಂಪತಿಗಳನ್ನು ನೋಡಕ್ಕೆ ಮತ್ತು ಕೌನ್ಸೆಲಿಂಗ್ ಕೊಡಕ್ಕೆ ನಾವು ಸರಿಯಾಗಿ ಕಲಿತುಕೊಂಡಿದ್ದೇನೆ ಎಂದು ಅವರೆಲ್ಲ ಒಪ್ಪಿಕೊಂಡಮೇಲೆನೇ ನಾನು ನಿಮ್ಮನ್ನು ಮತ್ತು ಬೇರೆ ಭಾಗವಹಿಸುವವರಿಗೆ ಕೌನ್ಸೆಲಿಂಗ್ ಕೊಡುವುದು ಶುರು ಮಾಡಿದ್ದು. ಅದರೊಟ್ಟಿಗೆ, ನನ್ನ ಎಲ್ಲ ಕೆಲಸವನ್ನು ಹೇಗೆ ಮಾಡಬೇಕೆಂದು ಗಮನಿಸ ಒಬ್ಬರು ಕ್ಲಿನಿಕಲ್ ಮನಶ್ಶಾಸ್ತ್ರಜ್ಞ ಈ ಯೋಜನೆಯಲ್ಲಿದ್ದಾರೆ. ನಿಮಗೆ ಸರಿಯಾದ ಸಹಾಯ ಮತ್ತು ಕೌನ್ಸೆಲಿಂಗ್ಅ ನೀಡಲು ನಾನು ಅವರೊಂದಿಗೆ ನಿಯಮಿತವಾಗಿ ಚರ್ಚಿಸುತ್ತಿರುತ್ತೇನೆ.”*

*“Nanage narsiṅg nalli ḍigri ide; nānu tumbā samaya peśeṇṭs mattu avarannu nōḍikoḷḷuvavarondige mātanāḍi, sahāya māḍi nanage gottide. Tumba kalita ḍākṭar gaḷu mattu bēreyavarinda tarabēti paḍedukoṇḍu mattu nim'ma tara dampatigaḷannu nōḍakke mattu kaunseliṅg koḍakke nāvu sariyāgi kalitukoṇḍiddēne endu avarella oppikoṇḍamēlenē nānu nim'mannu mattu bēre bhāgavahisuvavarige kaunseliṅg koḍuvudu śuru māḍiddu. Adaroṭṭige, nanna ella kelasavannu hēge māḍabēkendu gamanisa obbaru klinikal manaśśāstrajña ī yōjaneyalliddāre. Nimage sariyāda sahāya mattu kaunseliṅga nīḍalu nānu avarondige niyamitavāgi carcisuttiruttēne.”*

**Step iii:** In case of concerns related to the gender of the counselor i.e., the male participant may feel uncomfortable speaking with a female counselor, or a female participant may feel uncomfortable speaking with a male counselor: gently explore what makes them feel uncomfortable – it could be the sensitive nature of the topics discussed, such as alcohol consumption, anger, violence, etc. It may also be that they fear being judged by you. They may additionally have concerns regarding you taking sides based on your gender.

In this case, as stated above, mention your qualification and training. Reassure them of a. confidentiality, b. therapist/counselor neutrality, and c. your non-judgmental stance.

*“As I have told you earlier, everything that you share here will be kept confidential. We will not disclose any details of your participation or of what you say to anybody outside of the study. What is brought up here is discussed primarily with my supervisor, and even then, they would not know of your identity. Do remember that my role here is not to take sides or tell you who is right or wrong – it is not you [refer to one partner] versus you [refer to the other partner], but it is both of you together versus your problems.*

*"ನಾನು ಈ ಹಿಂದೆ ನಿಮಗೆ ಹೇಳಿದಹಾಗೆ, ನೀವು ಇಲ್ಲಿ ಮಾತನಾಡಿದ್ದೆಲ್ಲಾ ವಿಷಯ ನಾವು ಗೌಪ್ಯವಾಗಿ ಇಡುತ್ತೇವೆ. ನಿಮ್ಮ ಭಾಗವಹಿಸುವಿಕೆಯ ಬಗ್ಗೆ ಅಥವಾ ನಿಮ್ಮ ವಿವರಗಳನ್ನು ನಾವು ಈ ಯೋಜನೆಯ ಹೊರಗಿನ ಯಾವುದೇ ಜನರ ಹತ್ತಿರ ಹೇಳಿಕೊಳ್ಳಲ್ಲ. ನಾವು ಇಲ್ಲಿ ಮಾತನಾಡಿದ್ದು ಕೆಲವೊಮ್ಮೆ ನನ್ನ ಸೂಪರ್ವೈಸರ್ ಜೊತೆ ಚರ್ಚೆ ಮಾಡಬಹುದು, ಆದರೂ ಸಹ ಅವರಿಗೆ ನೀವು ಯಾರು ಏನು ಎಂದು ಗೊತ್ತಿರಲ್ಲ. ನೆನಪಿಟ್ಟುಕೊಳ್ಳಿ - ನನ್ನ ಜವಾಬ್ದಾರಿ ಇಲ್ಲಿ ಯಾರು ಸರಿ ಯಾರು ತಪ್ಪು ಎಂದು ಹೇಳಕ್ಕೆ ಅಲ್ಲ. ಇದು ನೀವು [ಹೆಂಡತಿಯನ್ನು ಉಲ್ಲೇಖಿಸಿ] ವಿರುದ್ಧ ನೀವು [ಪತಿಯನ್ನು ಉಲ್ಲೇಖಿಸಿ] ಅಲ್ಲ - ನೀವಿಬ್ಬರೂ ನಿಮ್ಮ ಸಮಸ್ಯೆಗಳ ವಿರುದ್ಧ.”*

*"Nānu ī hinde nimage hēḷidahāge, nīvu illi mātanāḍiddellā viṣaya nāvu gaupyavāgi iḍuttēve. Nim'ma bhāgavahisuvikeya bagge athavā nim'ma vivaragaḷannu nāvu ī yōjaneya horagina yāvudē janara hattira hēḷikoḷḷalla. Nāvu illi mātanāḍiddu kelavom'me nanna sūparvaisar jote carce māḍabahudu, ādarū saha avarige nīvu yāru ēnu endu gottiralla. Nenapiṭṭukoḷḷi - nanna javābdāri illi yāru sari yāru tappu endu hēḷakke alla. Idu nīvu [heṇḍatiyannu ullēkhisi] virud'dha nīvu [patiyannu ullēkhisi] alla - nīvibbarū nim'ma samasyegaḷa virud'dha.*

*My role is to help guide the two of you to communicate better and more positively with each other better, build trust in your relationship, and to help you find ways to show care for each other.*

*ನನ್ನ ಜವಾಬ್ದಾರಿ ಏನೆಂದರೆ ನಿಮ್ಮಿಬರ ಮಧ್ಯೆ ಒಳ್ಳೆಯ ಮಾತು ಕಥೆ ಪ್ರೋತ್ಸಾಹಿಸುವುದು, ಒಬ್ಬರನ್ನೊಬ್ಬರು ಒಳ್ಳೆಯ ರೀತಿಯಲ್ಲಿ ಮಾತನಾಡಿಸಿಕೊಳ್ಳುವುದು, ನಿಮ್ಮ ಸಂಬಂಧದಲ್ಲಿ ವಿಶ್ವಾಸ ಮತ್ತು ನಂಬಿಕೆ ಬೆಳೆಸುವುದು, ಮಾತು ಒಬ್ಬರನ್ನೊಬ್ಬರ ಕಡೆ ಕಾಳಜಿ ಮತ್ತು ಪ್ರೀತಿ ಹೇಗೆ ತೋರಿಸುವುದು ಎಂದು ತೋರಿಸಕ್ಕೆ.*

*Nanna javābdāri ēnendare nim'mibara madhye oḷḷeya mātu kathe prōtsāhisuvudu, obbarannobbaru oḷḷeya rītiyalli mātanāḍisikoḷḷuvudu, nim'ma sambandhadalli viśvāsa mattu nambike beḷesuvudu, mātu obbarannobbara kaḍe kāḷaji mattu prīti hēge tōrisuvudu endu tōrisakke.*

*I understand that these are difficult matters to talk about, especially to a man/woman (based on which partner expresses discomfort). I am here to understand your difficulties better with you, and not to judge you. Irrespective of what you share here, I will not think poorly of you for drinking/ [or anything else shared by the participants]. We are here to work on them so that you can cut down on your drinking/manage your emotions better/reduce your anger outbursts/learn to talk to each other effectively/ [any other concern].***”**

ಇದೆಲ್ಲ ಮಾತನಾದಕ್ಕೆ ಸುಲಭ ವಿಷಯಗಳಲ್ಲ ಎಂದು ನನಗೆ ತಿಳಿದಿದೆ, ವಿಶೇಷವಾಗಿ ಒಂದು ಗಂಡಸಿನೊಂದಿಗೆ/ಹೆಂಗಸಿನೊಂದಿದೆ. ನಿಮ್ಮ ಕಷ್ಟಗಳನ್ನು ಸರಿಯಾಗಿ ಅರ್ಥಮಾಡಿಕೊಳ್ಳಕ್ಕೆ ನಾನು ಇಲ್ಲಿ ಇದ್ದೇನೆ, ನಿಮ್ಮನ್ನು ತೀರಪನ ಮಾಡಕ್ಕೆ ಅಲ್ಲ. ನೀವು ಏನು ಮಾತು ಹೇಳಿದ್ದರೂ, ಕುಡಿಯುವುದಕ್ಕೆ/[ಬೇರೆ ಯಾವುದೇ ವಿಷಯಕ್ಕೆ] ನಾನು ನಿಮ್ಮನ್ನು ಕೀಳಾಗಿ ನೋಡಲ್ಲ. ಈ ವಿಷಯಗಳನ್ನು ಚರ್ಚಿಸಿ ನಿಮ್ಮ ಪರಿಸ್ಥಿತಿಯನ್ನು ಉತ್ತಮಗೊಳ್ಳಿಸಕ್ಕೆ, ನಿಮ್ಮ ಕುಡಿತ ಕಡಿಮೆ ಮಾಡಕ್ಕೆ/ನಿಮ್ಮ ಭಾವನೆಗಳನ್ನು ಒಳ್ಳೆಯ ರೀತಿಯಲ್ಲಿ ನಿಭಾಯಿಸಕ್ಕೆ/ನಿಮ್ಮ ಕೋಪವನ್ನು ಕಡಿಮೆ ಮಾಡಕ್ಕೆ/ ಮಾತುಕಥೆ ಚನ್ನಾಗಿ ನಡೆಸಕ್ಕೆ ನಾವೆಲ್ಲರೂ ಇಲ್ಲಿ ಸಭೆಯಲ್ಲಿದ್ದೇವೆ.

Idella mātanādakke sulabha viṣayagaḷalla endu nanage tiḷidide, viśēṣavāgi ondu gaṇḍasinondige/heṅgasinondide. Nim'ma kaṣṭagaḷannu sariyāgi arthamāḍikoḷḷakke nānu illi iddēne, nim'mannu tīrapana māḍakke alla. Nīvu ēnu mātu hēḷiddarū, kuḍiyuvudakke/[bēre yāvudē viṣayakke] nānu nim'mannu kīḷāgi nōḍalla. Ī viṣayagaḷannu carcisi nim'ma paristhitiyannu uttamagoḷḷisakke, nim'ma kuḍita kaḍime māḍakke/nim'ma bhāvanegaḷannu oḷḷeya rītiyalli nibhāyisakke/ Nim'ma kōpavannu kaḍime māḍakke/ mātukathe cannāgi naḍesakke nāvellarū illi sabheyalliddēve.

**Step iv:** If their concerns persist, discuss these concerns with the supervisor in the subsequent supervision sessions. If needed and deemed non-negotiable by the participant, a different counselor may need to be assigned to the couple based on their age and sex.

***13. Couple needs to bring their young children, or children are in the session.***

Discuss with the couple if they can identify friends, relatives or neighbors, or a nearby anganawadi at which they could drop off their young child/children for care and pick them back up after the session ends.

***14. Couple is in a rush to finish the session.***

**Step i:** Ask the participants the reason for them wanting to finish the session fast.

*“It appears to me that you [one partner]/both of you are in a hurry…could you tell me if something is on your mind/is there something you need to get to right now?”*

*"ನನಗೆ ನೀವು ಅವಸರದಲ್ಲಿದ್ದ ಹಾಗೆ ಅನಿಸುತ್ತದೆ...ನಿಮ್ಮ ಮನಸ್ಸು ಎಲ್ಲಿದೆ ಎಂದು ನನಗೆ ಹೇಳುತ್ತೀರಾ/ನಿಮಗೆ ಎಲ್ಲಿಯೋ ಅರ್ಜೆಂಟ್/ಅವಸರದಲ್ಲಿ ಹೋಗಕ್ಕೆ ಇದೆಯಾ?"*

*"Nanage nīvu avasaradallidda hāge anisuttade...Nim'ma manas'su ellide endu nanage hēḷuttīrā/nimage elliyō arjeṇṭ/avasaradalli hōgakke ideyā?"*

**Step ii:** Listen to what they cite as a reason for rushing through the session. Potential reasons: my child is waiting for me, chores, work matters, boredom/disinterest, sessions are useless.

*Boredom/disinterest:* Refer to Step iii of Situation Number 8.

*Sessions are useless:* Refer to Step iii of Situation Number 8.

*Work/chores:* re-state that each session lasts about an hour/hour-and-a-half, only once a week, for ___ times more (depending on how many sessions are remaining).

*“I understand that you must have a lot of work/chores at home to get back to…it’s only natural for you to feel preoccupied thinking about that right now. It is important that you take a short break away from all of that and be present here in the session. Once the session wraps up, you can return home/to work immediately after and get right to it. If we rush this too much right now, whatever we talk about may not be very useful to you, and there would be no point in you having come all this way to attend the session. We have ____ [time] left to go…could we go through what we have discussed a little slowly, so that I know you have understood? After that we can surely wrap up for the day.”*

*ಮನೆಯಲ್ಲಿ ಬಹಳ ಕೆಲಸ ಇದೆ ಎಂದು ನನಗೆ ತಿಳಿದಿದೆ. ಇಲ್ಲಿ ಇದ್ದಾಗ ಅದರ ಬಗ್ಗೆ ಯೋಚಿಸುವುದು ಸಹಜ...ನನಗೆ ಅರ್ಥಾಗತ್ತೆ. ಆ ಜವಾಬ್ದಾರಿಗಳಿಂದ ಒಂದು ಸ್ವಲ್ಪ ದೂರ ಬಂದು ಈ ಸಭೆಯಲ್ಲಿ ನಿಮ್ಮ ಗಮನ ಹಾಕುವುದು ಮುಖ್ಯ. ಈ ಸಭೆ ಮುಗಿದ ಕೂಡಲೇ ನೀವು ತಕ್ಷಣ ಮನೆಗೆ ಹೋಗಿಬಿಟ್ಟು ನಿಮ್ಮ ಎಲ್ಲಾ ಕೆಲಸ ಮಾಡುವುದು ಶುರು ಮಾಡಬಹುದು. ನಾವು ಅವಸರದಲ್ಲಿ ಇಲ್ಲಿ ಸಭೆಯನ್ನು ಮುಗಿಸಿದ್ದಾರೆ, ನಾವು ಇಲ್ಲಿ ಮಾತನಾಡುವುದಕ್ಕೆ ಏನು ನಿಮಗೆ ಉಪಯೋಗ ಆಗಲ್ಲ. ಇಷ್ಟು ದೂರ ಈ ಸಭೆಯಲ್ಲಿ ಭಾಗವಹಿಸಕ್ಕೆ ಬಂದದ್ದು ವ್ಯರ್ಥವಾಗಿ ಹೋಗಿಬಿಡತ್ತೆ. ನಮಗೆ ____ ಸಮಯ ಉಳಿದಿದ್ದೆ...ನಾವು ಇವತ್ತು ಚರ್ಚಿಸಿದ್ದನ್ನು ಸ್ವಲ್ಪ ನಿಧಾನಕ್ಕೆ ಮಾತನಾಡಿಕೊಳ್ಳುವನಾ? ಅವಾಗ ನಿಮಗೆ ಅರ್ಥಆಗಿದೆ ಎಂದು ನನಗೆ ತಿಳಿಯತ್ತೆ. ಮಾತನಾಡಿ ಮುಗಿದ ಮೇಲೆ ನಮಗೆ ಈ ಸಭೆಯನ್ನು ನಿಲ್ಲಿಸಬಹುದು, ಮತ್ತು ನಿಮಗೆ ನಿಮ್ಮ ಕೆಲಸಕ್ಕೆ ಹಿಂದೆ ಹೋಗಬಹುದು."*

*“Maneyalli bahaḷa kelasa ide endu nanage tiḷidide. Illi iddāga adara bagge yōcisuvudu sahaja...Nanage arthāgatte. Ā javābdārigaḷinda ondu svalpa dūra bandu ī sabheyalli nim'ma gamana hākuvudu mukhya. Ī sabhe mugida kūḍalē nīvu takṣaṇa manege hōgibiṭṭu nim'ma ellā kelasa māḍuvudu śuru māḍabahudu. Nāvu avasaradalli illi sabheyannu mugisiddāre, nāvu illi mātanāḍuvudakke ēnu nimage upayōga āgalla. Iṣṭu dūra ī sabheyalli bhāgavahisakke bandaddu vyarthavāgi hōgibiḍatte. Namage ____ samaya uḷididde...* *Nāvu ivattu carcisiddannu svalpa nidhānakke mātanāḍikoḷḷuvanā? Avāga nimage artha'āgide endu nanage tiḷiyatte. Mātanāḍi mugida mēle namage ī sabheyannu nillisabahudu, mattu nimage nim'ma kelasakke hinde hōgabahudu."*

*Child(ren*): *“I’m glad that you identified someone you trust to take care of your child for you during the time you spend here in counseling. I can imagine how badly you must want to get back to them! As you have informed me, they are in good hands, and I’m certain you have informed the caretakers about how long this would take you. We will be done in ___ [time], after which you can go pick your children up. If we rush this too much right now, whatever we talk about may not be very useful to you, and there would be no point in you having come all this way to attend the session. We have ____ [time] left to go…could we go through what we have discussed a little slowly, so that I know you have understood? After that we can surely wrap up for the day”.*

*“ನೀವು ಈ ಸಭೆಯಲ್ಲಿ ಇದ್ದಾಗ ನಿಮ್ಮ ಮಕ್ಕಳನ್ನು ನೋಡಿಕೊಳ್ಳುವುದಕ್ಕೆ ಯಾರನ್ನೋ ಹುಡಿಕಿತ್ತದ್ದು ಒಳ್ಳೆಯದಾಯಿತು. ನಿಮಗೆ ಈವಾಗಲೇ ನಿಮ್ಮ ಸಣ್ಣ ಮಕ್ಕಳ ಹತ್ತಿರ ಹೋಗಿ ಬಿಡುವ ಎಂದು ಅನಿಸುತ್ತಿರಬಹುದು! ನೀವು ಹೇಳಿದಹಾಗೆ, ಅವರು ವಿಶ್ವಾಸನೀಯ ಜನರೊಂದಿಗೆ ಇದ್ದಾರೆ, ಮತ್ತು ನೋಡಿಕೊಳ್ಳುವವರಿಗೆ ಇಲ್ಲಿ ನಿಮಗೆ ಎಷ್ಟು ಸಮಯ ಹಿಡಿಯತ್ತೆ ಎಂದು ನೀವು ತಿಳಿಸಿರಬಹುದು. ____ಸಮಯದಲ್ಲಿ ನಮ್ಮ ಸಭೆ ಮುಗಿಯುತ್ತೆ. ಅದಾದ ಕೂಡಲೇ ನೀವು ನಿಮ್ಮ ಮಕ್ಕಳ ಹತ್ತಿರ ನೀವು ಹೋಗಬಹುದು. ನಾವು ಅವಸರದಲ್ಲಿ ಇಲ್ಲಿ ಸಭೆಯನ್ನು ಮುಗಿಸಿದ್ದಾರೆ, ನಾವು ಇಲ್ಲಿ ಮಾತನಾಡುವುದಕ್ಕೆ ಏನು ನಿಮಗೆ ಉಪಯೋಗ ಆಗಲ್ಲ. ಇಷ್ಟು ದೂರ ಈ ಸಭೆಯಲ್ಲಿ ಭಾಗವಹಿಸಕ್ಕೆ ಬಂದದ್ದು ವ್ಯರ್ಥವಾಗಿ ಹೋಗಿಬಿಡತ್ತೆ. ನಮಗೆ ____ ಸಮಯ ಉಳಿದಿದ್ದೆ...ನಾವು ಇವತ್ತು ಚರ್ಚಿಸಿದ್ದನ್ನು ಸ್ವಲ್ಪ ನಿಧಾನಕ್ಕೆ ಮಾತನಾಡಿಕೊಳ್ಳುವನಾ? ಅವಾಗ ನಿಮಗೆ ಅರ್ಥಆಗಿದೆ ಎಂದು ನನಗೆ ತಿಳಿಯತ್ತೆ. ಮಾತನಾಡಿ ಮುಗಿದ ಮೇಲೆ ನಮಗೆ ಈ ಸಭೆಯನ್ನು ನಿಲ್ಲಿಸಬಹುದು, ಮತ್ತು ನಿಮಗೆ ನಿಮ್ಮ ಕೆಲಸಕ್ಕೆ ಹಿಂದೆ ಹೋಗಬಹುದು."*

*“Nīvu ī sabheyalli iddāga nim'ma makkaḷannu nōḍikoḷḷuvudakke yārannō huḍikittaddu oḷḷeyadāyitu. Nimage īvāgalē nim'ma saṇṇa makkaḷa hattira hōgi biḍuva endu anisuttirabahudu! Nīvu hēḷidahāge, avaru viśvāsanīya janarondige iddāre, mattu nōḍikoḷḷuvavarige illi nimage eṣṭu samaya hiḍiyatte endu nīvu tiḷisirabahudu. ____Samayadalli nam'ma sabhe mugiyutte. Adāda kūḍalē nīvu nim'ma makkaḷa hattira nīvu hōgabahudu. Nāvu avasaradalli illi sabheyannu mugisiddāre, nāvu illi mātanāḍuvudakke ēnu nimage upayōga āgalla. Iṣṭu dūra ī sabheyalli bhāgavahisakke bandaddu vyarthavāgi hōgibiḍatte. Namage ____ samaya uḷididde...* *Nāvu ivattu carcisiddannu svalpa nidhānakke mātanāḍikoḷḷuvanā? Avāga nimage artha'āgide endu nanage tiḷiyatte. Mātanāḍi mugida mēle namage ī sabheyannu nillisabahudu, mattu nimage nim'ma kelasakke hinde hōgabahudu."*

**Step iii:** If this happens repeatedly (more than one time), bring it up in the supervision session with your supervisor.

***15. Handling situations of varying literacy between the couple, which leads to one understanding and engaging more than the other.***

**Step i:** Identify that differences between the partners in terms of grasping ability may be due to differences in literacy levels. Do *not* overtly bring up this matter in the session, as it may make the partner feel self-conscious and hamper the growth of the therapeutic alliance.

**Step ii:** Slow down the pace of the session. If one partner gets impatient with the speed, state to the couple that it is important for all participants in the session to understand and take away as much valuable information as possible. You may provide a disclaimer at the start of the session that you will be checking in with each partner about whether they have understood what is discussed a few times during the session.

*“Do you have any questions about the joint problem-solving steps we went over just now? Please feel free to ask me.”*

*"ಜೊತೆಯಲ್ಲಿ ಸಮಸ್ಯೆಗಳನ್ನು ಪರಿಹರಿಸುವ ವಿಧಾನದ ಹಂತಗಳ ಬಗ್ಗೆ ನಿಮಗೆ ಏನಾದರೂ ಪ್ರಶ್ನೆಗಳಿವೆಯೇ? ನನಗೆ ಕೇಳಲು ಹಿಂಜರಿಯಬೇಡಿ."*

*"Joteyalli samasyegaḷannu pariharisuva vidhānada hantagaḷa bagge nimage ēnādarū praśnegaḷiveyē? Nanage kēḷalu hin̄jariyabēḍi."*

*“There is no such thing as “big” or “small” question/silly or “stupid” question. Your questions are important for you to take away as much as you can from our sessions, and for me to know that you are paying attention, are curious, and eager to learn!”*

*"ಸಣ್ಣ ಪ್ರಶ್ನೆ ಅಥವಾ ದೊಡ್ಡ ಪ್ರಶ್ನೆ ಎಂದು ಏನೂ ಇಲ್ಲ. ನಿಮಗೆ ಈ ಸಭೆಗಳಿಂದ ಲಾಭ ಆಗಕ್ಕೆ ಎಲ್ಲ ಪ್ರಶ್ನೆಗಳನ್ನು ಕೇಳುವುದು ಮುಖ್ಯ! ನೀವು ಪ್ರಶ್ನೆ ಕೇಳಿದ್ದಾರೆ ನೀವು ಸಭೆಯಲ್ಲಿ ಗಮನಿಸುತ್ತಿದ್ದೀರಿ ಮತ್ತು ಕಲಿತುಕೊಳ್ಳಕ್ಕೆ ಉತ್ಸುಕರಾಗಿದ್ದೀರಿ ಎಂದು ನನಗೆ ತಿಳಿಯತ್ತೆ."*

*"Saṇṇa praśne athavā doḍḍa praśne endu ēnū illa. Nimage ī sabhegaḷinda lābha āgakke ella praśnegaḷannu kēḷuvudu mukhya! Nīvu praśne kēḷiddāre nīvu sabheyalli gamanisuttiddīri mattu kalitukoḷḷakke utsukarāgiddīri endu nanage tiḷiyatte."*

*“It is important for me to know that both of you have understood what is being talked about here, and I would feel glad if you could contribute and participate fully. So, if either one of you or both feel that I’m going too fast, or that you need time to just soak in what is discussed, then I would truly appreciate it if you would let me know. Feel free to stop me and tell me that you would like a minute or two, or if you would like me to explain something to you again or in a different way. If you have understood but your partner has not, then it is a great opportunity to go over what you know again while I re-explain it, and to help me help your partner understand it too!”*

*“ನಿಮ್ಮಿಬ್ಬರಿಗೂ ನಾನು ಹೇಳುವುದೆಲ್ಲ ಅರ್ಥಾಗುತ್ತಿದೆ ಎಂದು ನನಗೆ ತಿಳಿಯಕ್ಕೆ ಬಹಳ ಮುಖ್ಯ. ನೀವಿಬ್ಬರೂ ಪೂರ್ಣವಾಗಿ ಮನಸ್ಸು ಹಾಕಿ ಭಾಗವಹಿಸಿದರೆ ಒಳ್ಳೆಯದು. ಯಾರೊಬ್ಬರಿಗೂ ಅಥವಾ ಇಬ್ಬರಿಗೆ ನಾನು ತುಂಬಾ ಫಾಸ್ಟ್/ಬೇಗ ಮಾತಾಡುತ್ತಿದ್ದೇನೆ ಎಂದು ಅನಿಸಿದ್ದರೆ ಅಥವಾ ನಾನು ಹೇಳಿದ್ದನ್ನೆಲ್ಲ ಕೇಳಿಸಿಕೊಂಡು ಅರ್ಥಮಾಡಿಕೊಳ್ಳಕ್ಕೆ ಸ್ವಲ್ಪ ಸಮಯ ಬೇಕು ಎಂದು ಅನಿಸಿದ್ದರೆ, ನೀವು ನನಗೆ ಸ್ಪಷ್ಟವಾಗಿ, ಹಿಂಜರಿಯದೆ ನನಗೆ ಹೇಳಿದ್ದಾರೆ ನನಗೆ ಬಹಳ ಸಂತೋಷವಾಗುತ್ತದೆ. ಒಂದು-ಎರಡು ನಿಮಿಷ ಬ್ರೇಕ್/ಆರಾಮ ಬೇಕು ಎಂದು ಹೇಳಕ್ಕೆ ಅಥವಾ ನಾನು ಹೇಳಿದ್ದನ್ನು ಬೇರೆ ರೀತಿಯಲ್ಲಿ ವಿವರಿಸಬೇಕೆಂದು ನಿಮಗೆ ಅನಿಸಿದ್ದರೆ ನನಗೆ ತಿಳಿಸಕ್ಕೆ ಹಿಂಜರಿಯಬೇಡಿ. ನಿಮಗೆ ಅರ್ಥಆಗಿದ್ದರೆ ಆದರೆ ನಿಮ್ಮ ಹೆಂಡತಿ/ಗಂಡನಿಗೆ ಇನ್ನು ಸ್ವಲ್ಪ ಸಮಯ ಬೇಕೆಂದು ಅನಿಸಿದ್ದರೆ, ನಾನು ಮತ್ತಿಂದು ಸಲ ವಿವರಿಸುತ್ತಿದ್ದಾಗ ಕೇಳಿಸಿಕೊಂಡು ನಿಮ್ಮ ಗಂಡ/ಹೆಂಡತಿಗೆ ಅರ್ಥಮಾಡಿಕೊಳ್ಳಕ್ಕೆ ಸಹಾಯ ಮಾಡುವುದಕ್ಕೆ ಒಳ್ಳೆಯ ಅವಕಾಶ!”*

*“Nim'mibbarigū nānu hēḷuvudella arthāguttide endu nanage tiḷiyakke bahaḷa mukhya. Nīvibbarū pūrṇavāgi manas'su hāki bhāgavahisidare oḷḷeyadu. Yārobbarigū athavā ibbarige nānu tumbā phāsṭ/bēga mātāḍuttiddēne endu anisiddare athavā nānu hēḷiddannella kēḷisikoṇḍu arthamāḍikoḷḷakke svalpa samaya bēku endu anisiddare, nīvu nanage spaṣṭavāgi, hin̄jariyade nanage hēḷiddāre nanage bahaḷa santōṣavāguttade. Ondu-eraḍu nimiṣa brēk/ārāma bēku endu hēḷakke athavā nānu hēḷiddannu bēre rītiyalli vivarisabēkendu nimage anisiddare nanage tiḷisakke hin̄jariyabēḍi. Nimage artha'āgiddare ādare nim'ma heṇḍati/gaṇḍanige innu svalpa samaya bēkendu anisiddare, nānu mattindu sala vivarisuttiddāga kēḷisikoṇḍu nim'ma gaṇḍa/heṇḍatige arthamāḍikoḷḷakke sahāya māḍuvudakke oḷḷeya avakāśa!”*

**Step iii:** If these concerns persist, discuss them with the supervisor at the subsequent supervision session.

***16. Handling gender-based resistance. For example, a man pushes back about his role in helping out around the house as an act of caring.***

**Step i:** Listen to what the participant has to say about refusing to partake in an activity that they feel is “not right” for them to do, or which outright refuse to do. Your non-verbal gestures and posture need to remain open and receptive; this is to ensure that the participant does not feel that the situation is combative but is more a space to be listened to about their reservations with respect and patience – without you necessarily having to agree with what they are saying. It is important that you do not react or lash out (if/as these may be personally triggering matters to you) but do clearly communicate the following.

**Step ii:** Paraphrase what the participant has said about refusing to do a particular activity. Acknowledge and appreciate the efforts that both partners are putting in to attend the sessions. With reference to the Couple Session Guideline “ACT DIFFERENTLY BEFORE YOU FEEL DIFFERENTLY”, the objectives of Project Harmony, and the overall benefits of partaking in couple sessions, calmly describe how it may help the participant in a different way to merely try out the activity.

*“Let me know if I have understood what you said correctly – you are saying that you do not want to ______ for your wife because you feel that that is not how things work in your family…and that you are already caught up with other important tasks and responsibilities…you feel that it is solely the wife’s responsibility to_____. Did I get that right?*

*ನಿಮಗೆ ನಿಮ್ಮ ಹೆಂಡತಿಗೋಸ್ಕರ ____ ಮಾಡಕ್ಕೆ ಮನಸ್ಸಿಲ್ಲ ಏಕೆಂದರೆ ನಿಮ್ಮ ಕುಟುಂಬದಲ್ಲಿ ಆ ರೀತಿಯಲ್ಲಿ ಎಲ್ಲ ಕೆಲಸ ನದಿಯಲ್ಲ, ಮತ್ತು ನಿಮಗೆ ಇವಾಗಲೀ ಬೇರೆ ಮುಖ್ಯ ಕೆಲಸ ಮತ್ತು ಜವಾಬ್ದಾರಿಗಳಿವೆ ಎಂದು ಅನಿಸತ್ತೆ ಮತ್ತು _____ ಮಾಡುವುದು ನಿಮ್ಮ ಹೆಂಡತಿ ಒಬ್ಬರದೇ ಜವಾಬ್ದಾರಿ ಎಂದು ನೀವು ಹೇಳುತ್ತಿದ್ದೀರಿ...ನಾನು ನೀವು ಹೇಳಿದ್ದನ್ನು ಸರಿಯಾಗಿ ಅರ್ಥ ಮಾಡಿಕೊಂಡೆನಾ?*

*Nimage nim'ma heṇḍatigōskara ____ māḍakke manas'silla ēkendare nim'ma kuṭumbadalli ā rītiyalli ella kelasa nadiyalla, mattu nimage ivāgalī bēre mukhya kelasa mattu javābdārigaḷive endu anisatte mattu _____ māḍuvudu nim'ma heṇḍati obbaradē javābdāri endu nīvu hēḷuttiddīri...Nānu nīvu hēḷiddannu sariyāgi artha māḍikoṇḍenā?*

*I understand that you feel this way. The point of these activities is to help you improve your relationship with your wife – it is not just your wife who would benefit from this, but you would do too. It would be great if the overall environment at home would be more pleasant for you and your wife both. Just like it may make you feel cared for and relaxed when, after a long day’s work, your wife makes a steaming cup of tea for you, it would make your wife feel similarly if you helped her out with _____. Like we had discussed, you may not feel loving or caring or happy from the word-go, because you will have to Act Differently first; the more regularly you do it, you will start to Feel Differently, i.e., more positively towards your wife. The same goes for how your wife will feel about you.*

*ನಿಮಗೆ ಈ ತರ ಅನಿಸುತ್ತಿದೆ ಎಂದು ನನಗೆ ಅರ್ಥಾಗತ್ತೆ. ಈ ಚಟುವಟಿಕೆಗಳ ಉದ್ದೇಶ್ಯ ಇರುವುದು ನೀವು ಮತ್ತು ನಿಮ್ಮ ಹೆಂಡತಿಯ ಸಂಬಂಧವನ್ನು ಉತ್ತಮಗೊಳ್ಳಿಸಕ್ಕೆ. ನಿಮ್ಮ ಹೆಂಡತಿಗೆ ಇದರಲ್ಲಿ ಭಾಗವಹಿಸಿ ಮಾತ್ರ ಲಾಭ ಸಿಗೋದು ಅಲ್ಲ, ನಿಮಗೂ ಸಹ. ಮನೆಯ ವಾತಾವರಣ ನಿಮಗೆ ಮತ್ತು ನಿಮ್ಮ ಹೆಂಡತಿ ಇಬ್ಬರಿಗೂ ಶಾಂತಿ ಆದರೆ ನಿಮ್ಮಿಬ್ಬರಿಗೆ ವೊಳ್ಳೆಯದಾಗತ್ತೆ. ಒಂದು ಪೂರ್ತಿ ದಿನ ಕಷ್ಟದ ಕೆಲಸ ಮಾಡಿ ನೀವು ಮನೆಗೆ ಹಿಂದೆ ಬಂದಾಗ ನಿಮ್ಮ ಹೆಂಡತಿ ಒಂದು ಚಾ ಮಾಡಿ ಕೊಟ್ಟಾಗ ನಿಮಗೆ ಸಂತೋಷ ಅನಿಸತ್ತೆ ಮತ್ತು ನಿಮ್ಮ ಕಡೆ ಕಾಳಜಿ ತೋರುತ್ತಾರೆ, ಹಾಗೇನೇ ನೀವು ನಿಮ್ಮ ಹೆಂಡತಿಗೆ ____ ಒಟ್ಟಿಗೆ ಸಹಾಯ ಮಾಡಿದ್ದಾಗ, ಅವರಿಗೂ ಬಹಳ ಸಂತೋಷವಾಗುತ್ತದೆ. ನಾವು ಈ ಮಾತು ಹೇಳಿದ ಕೂದಲ ನಿಮಗೆ ನಿಮ್ಮ ಹೆಂಡತಿ ಕಡೆ ಸಂತೋಷ ಅಥವಾ ಕಾಳಜಿ ಅಥವಾ ಪ್ರೀತಿ ಹುಟ್ಟಿಬರತ್ತೆ ಎಂದು ಹೇಳುವುದಲ್ಲ...ಮೊದಲಾಗಿ ನೀವು ಅವರ ಕಡೆ ನಿಮ್ಮ ವರ್ತನೆ/ನಡವಳಿಕೆ ಬದಲಾಯಿಸಬೇಕು. ಅದಾದಮೇಲೆನೇ ನಿಮ್ಮ ಹೆಂಡತಿ ಕಡೆ ನಿಮ್ಮ ಭಾವನೆಗಳೂ ಬದಲಾಗುತ್ತವೆ. ನಿಮ್ಮ ಹೆಂಡತಿಗೆ ನಿಮ್ಮ ಬಗ್ಗೆ ಏನು ಅನಿಸುತ್ತದೆ, ಅದೂ ಒಳ್ಳೆಯದಾಗಿ ಬದಲಾಗುತ್ತದೆ.*

*Nimage ī tara anisuttide endu nanage arthāgatte. Ī caṭuvaṭikegaḷa uddēśya iruvudu nīvu mattu nim'ma heṇḍatiya sambandhavannu uttamagoḷḷisakke. Nim'ma heṇḍatige idaralli bhāgavahisi mātra lābha sigōdu alla, nimagū saha. Maneya vātāvaraṇa nimage mattu nim'ma heṇḍati ibbarigū śānti ādare nim'mibbarige voḷḷeyadāgatte. Ondu pūrti dina kaṣṭada kelasa māḍi nīvu manege hinde bandāga nim'ma heṇḍati ondu cā māḍi koṭṭāga nimage santōṣa anisatte mattu nim'ma kaḍe kāḷaji tōruttāre, hāgēnē nīvu nim'ma heṇḍatige ____ . Oṭṭige sahāya māḍiddāga, avarigū bahaḷa santōṣavāguttade. Nāvu ī mātu hēḷida kūdala nimage nim'ma heṇḍati kaḍe santōṣa athavā kāḷaji athavā prīti huṭṭibaratte endu hēḷuvudalla...Modalāgi nīvu avara kaḍe nim'ma vartane/naḍavaḷike badalāyisabēku. Adādamēlenē nim'ma heṇḍati kaḍe nim'ma bhāvanegaḷū badalāguttave. Nim'ma heṇḍatige nim'ma bagge ēnu anisuttade, adū oḷḷeyadāgi badalāguttade.*

*I understand that this is not how things go in your family, and you may be concerned about what people say…is that a concern that you may have?* [encourage the partner to talk about their reservations/concerns about doing the activity, be receptive and open]. *I understand, thank you for telling me. You doing these tasks is for your wellbeing and for your family’s wellbeing, and nobody else’s. You had shared with us earlier how you would like things to be better at home and in your relationship. Just because this is how things have been going on for so long, does not mean it has to continue this way…especially if it is not working/beneficial for you or your loved ones. Both of you can change that for yourself for the years to come. What are your thoughts on that?*

*ನಿಮ್ಮ ಕುಟುಂಬದಲ್ಲಿ ಈ ತರ ಎಲ್ಲ ನಡೆಯಲ್ಲ ಎಂದು ನನಗೆ ಅರ್ಥಾಗತ್ತೆ. ಜನರೆಲ್ಲಾ ನಿಮ್ಮ ಬಗ್ಗೆ ಏನು ತಿಳಿದುಕೊಳ್ಳಬಹುದೆಂದೇ ನಿಮಗೆ ಚಿಂತೆನೂ ಆಗುತ್ತಿರಬಹುದು. ನಿಮ್ಮ ಅನಿಸಿಕೆ ಏನಿದೆ...ಈ ತರದ ಚಿಂತೆ ಇದೆಯಾ? ನನಗೆ ಅರ್ಥಾಗತ್ತೆ. ನನ್ನೊಂದಿಗೆ ಈ ಮಾತುಗಳನ್ನು ಹಂಚಿದಕ್ಕೆ ಧನ್ಯವಾದಗಳು. ನೀವು ಈ ಚಟುವಟಿಕೆಗಳನ್ನು ಮಾಡುವುದು ನಿಮ್ಮ ಒಳ್ಳೆಯದಕ್ಕೆ ಮತ್ತು ನಿಮ್ಮ ಕುಟುಂಬದ ಒಳ್ಳೆಯದಕ್ಕೆ...ಬೇರೆ ಯಾರಿಗೂ ಅಲ್ಲ. ನಮ್ಮೊಂದಿಗೆ ಈ ಹಿಂದೆ ನಿಮ್ಮ ಮನೆಯ ವಾತಾವರಣ ಮತ್ತು ನಿಮ್ಮ ಸಂಬಂಧಗಳನ್ನು ಉತ್ತಮಗೊಲಿಡಬೇಕೆಂದು ನೀವು ಹೇಳಿದ್ದೀರಿ. ಎಷ್ಟೂ ಸಮಯದಿಂದ/ವರ್ಷಗಳಿಂದ ಹೀಗೆಯೇ ನಡೀತಿದ್ದರೂ ಇನ್ನು ಮುಂದೆ ಹೀಗೆಯೇ ನಡಿತಾ ಇರಬೇಕೆಂದು ಅಲ್ಲವೇ ಅಲ್ಲ...ವಿಶೇಷವಾಗಿ ನಿಮಗೆ ಮತ್ತು ನಿಮ್ಮ ಕುಟುಂಬದವರಿಗೆ ಅದರಿಂದ ಏನು ವೊಳ್ಳೆಯದಾಗುತ್ತಿರದಾಗ. ನೀವಿಬ್ಬರೂ ನಿಮ್ಮ ಭವಿಷ್ಯದಲ್ಲಿ ಈ ರೀತಿಯ ಒಳ್ಳೆಯ ಬದಲಾವಣೆ ತರುವುದು ಸಾಧ್ಯ. ನಿಮಗೆ ಇದೆಲ್ಲ ಬಗ್ಗೆ ಏನು ಅನಿಸುತ್ತದೆ?*

*If it helps, you may look at the duration of these sessions as the trial period… instead of outright refusing to try it at all, you may do the activities suggested for the weeks to come and see for yourself. You may then decide what of all of these you would like to continue to keep doing. How does that sound to you?”*

**Step iii:** If these concerns persist, discuss them with the supervisor in the subsequent supervision session.

***17. Female participant expresses mistrust of their husband’s commitment to stop drinking.***

***Woman might say:*** “I won’t believe him because he has told me he would stop drinking in the past but then he didn’t stop.”

***Sample counselor response:*** “It is only natural that you feel this way. Finding it difficult to trust him and each other is normal. Remember that progress may not happen all at once. Your partner may not reduce their drinking all at once. The same thing with trust: you may not immediately trust him if you don’t trust him right now. Even if you don’t trust him completely now, go ahead with the activity and mark that you were worried or doubtful in the discussion box: “Discussion: ☹” (show Daily Trust Contract: “COMPLETE THE TRUST CONTRACT EVEN ON A BAD DAY”). The trust contract is meant to help you improve your relationship. Just doing the contract will help you and your partner build that trust that you might not have right now.”

***Woman might say:*** “I wouldn’t want to do it if my partner is drunk because I will feel like he does not mean it.”

***Sample counselor response:*** “Remember that progress does not happen all at once. Your partner may not reduce his drinking all at once. The same thing with trust: you may not immediately trust him if you don’t trust him right now. The trust discussion is all about the future and developing the skills necessary to reduce drinking and to trust each other. Even if your partner is intoxicated, continue with the trust contract and mark that they were not sober in the discussion box: “Discussion: ☹” (show Daily Trust Contract: “COMPLETE THE TRUST CONTRACT EVEN ON A BAD DAY”). The trust contract is meant to help you improve your relationship and build trust that you might not have right now. Remember to support your partner in following through with the trust contract, even if they are drunk. Difficulty is normal.”

***18. Husband says he is not actually interested in reducing alcohol use***. Although they consented to the intervention, they may later say they are not interested. Telling them about the danger of alcohol use may not work. Instead, try to understand what they DO care about. Is it their friendship with people who drink together? Is it wanting a good future for their child (current or future)? Is it doing well in their job? Only after learning about what is important for him, make the connection with alcohol use.

***Sample counselor response:*** “If you don’t want to talk about drinking, that’s ok. I want to learn what are the things that are important for you in your life. Is it about your job, family, children, friendship, society, anything else?” (After hearing back from the husband): “Do you see a connection between drinking alcohol and keeping your job (replace with what the husband said is important for them)?”

***19. The person has another ongoing addiction that makes it difficult to stop alcohol use.*** Sometimes, people may be addicted to tobacco (smoked tobacco like cigarettes and bidi; and smokeless like khaini, gutkha) and when they use tobacco, they also drink alcohol. It may be difficult for the person to reduce alcohol unless they also cut down tobacco. In such a situation, first, describe the connection between the two addictions and ask them if they can try to reduce tobacco use as well. Some techniques can work for both (stress management, refusal skills, problem solving, etc.) but if they need extra help reducing tobacco, check with your supervisor for additional resources.

***20. If a participant expresses suicidal ideation.***

If a participant states that s/he feels suicidal i.e., may say anything along the lines of the following: “I wish I were dead.”, “I don’t want to live anymore.”, “I am better off dead.”, “I have been having dark thoughts of hurting myself.”, “I have been planning to kill myself.”, etc. [or anything similar], listen to the participant patiently and show compassion with your gestures and words. Do not immediately jump in or start talking. Instead, allow the person to talk about how they have been feeling. Nod when appropriate. Do not pass judgement on their feelings. Validate their feelings by saying something along the lines of the following.

*“I can see that you are going through a lot right now…I can imagine all the strong emotions you may be experiencing when you have these thoughts. Thank you for telling me about this, as I understand that it is very scary to experience all of this. I can see that you need help with these thoughts and feelings, so that you don’t hurt yourself. Your safety is paramount/very important to me. To ensure that you are safe and alright, it is crucial that I bring this to the notice of my supervisor. Rest assured that we will not tell anybody else about this, but we can get you the help you need right now. Would you be okay with me talking to my supervisor about this right now?”*

Note down the participant ID and notify the intervention coordinator and project manager about the participant.

**Appendix AD**

**REDCap User Manual**

# **a. How to get account details?**

- Once your account has been created, you’ll get a mail from REDCAP Admin at SJRI
- Note your username mentioned in the email.
- Click on the link mentioned in the email.
- You are prompted to enter a password of your choice, twice.
- Then you’ll have to enter a response to a secret question.
- Please note your password and secret question answer.
- Login to Redcap as per the details below.

# **b. How to login?**

- Go to Google Chrome or Firefox Application.
- LIVE PATIENT Data entry: <https://redcapcloud.sjri.res.in/>
- For testing / training/ testing cases:

<https://redcapcloud.sjri.res.in/redcap_v12.1.2/index.php?pid=220>

- Please save or book mark this link on your browser.
- Please don’t go to any other link, even though you may see the same screen.
- Enter your authorized username and password.
- Do not log in on behalf of any other individual.
- You will enter the **My Projects Page**


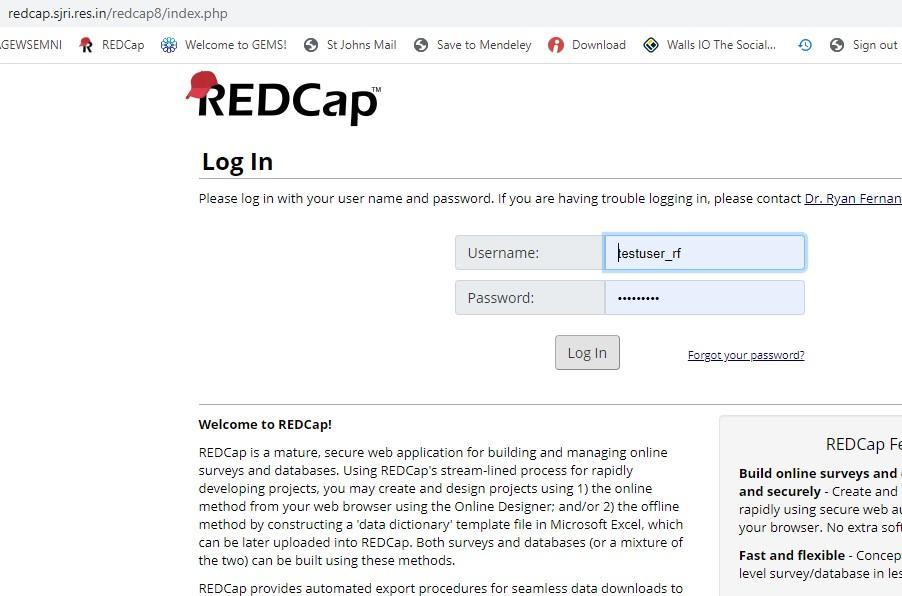


# **c. How to go to your project?**

- You will see a list of projects that you are assigned to.
- Click on the Project HARMONY (LIVE).
- Note for testing or training, Go to the project “HARMONY– UAT/TEST”. For entering LIVE patient data on the field, Go to the project “HARMONY– LIVE”. If you enter Test data in LIVE or LIVE data in test, there will be problems:: HARMONY (DEVELOPMENT).
- You will enter the Project landing page which provides multiple functions.

# **d. How to see your functions?**

- Click on the project you want to access or enter data.
- **You will see on the left-hand column all functions including: -**
  1. **Record Status Dashboard –** TO view and see the tracking board for all the patients with different visits. Again, you can select different views.
  2. **Add / Edit record –** To search a subject filled up forms and enter or edit the data.

# **e. How to recruit a subject?**

- Make sure you are permitted to create a new subject.
- Go to **Add/Edit Record.**


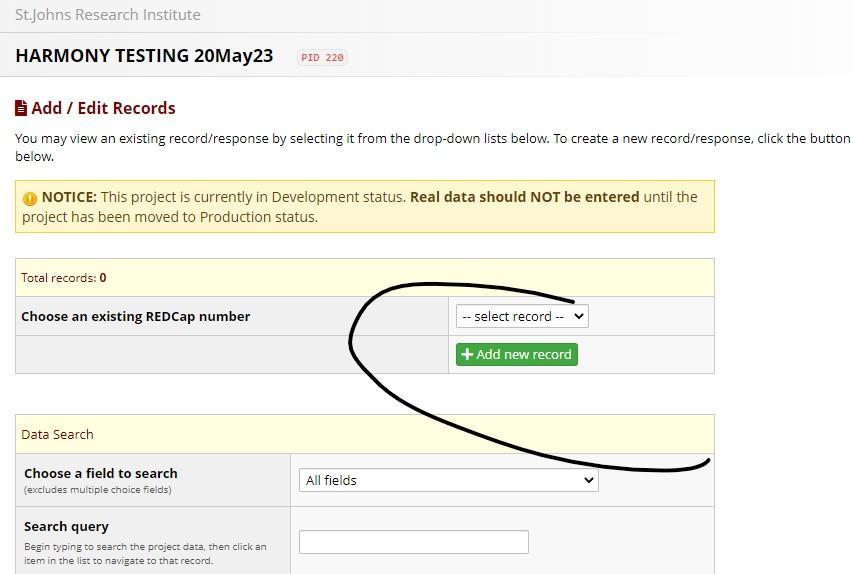


- The patient/Subject Home page will open up.


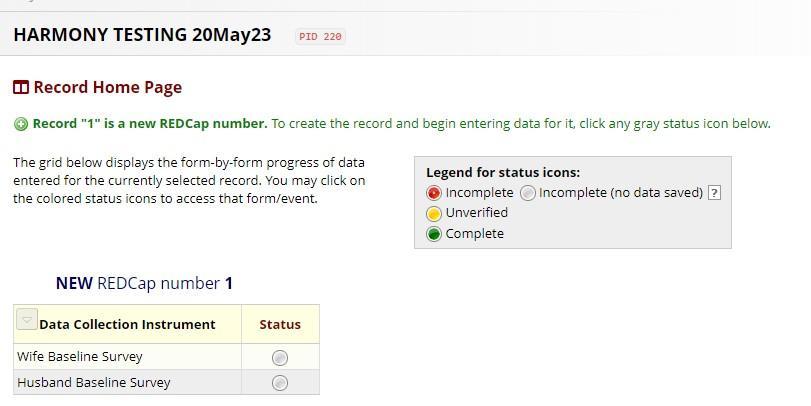


- Click on the Form Status as displayed.
- The respective forms will open (Wife Baseline Survey or Husband Baseline Survey).
- The other forms will not be visible.
- Kindly enter the data in event 1 (Baseline) and Event 2 onwards (Follow-up)
- There may be some fields which have logic and min-max values. These validations will have to be met. For example, the variable and other forms will open only if informed consent is marked as yes.
- Fill the form and then once all data is filled click on complete.

# **f. How to enter data in a form for existing subjects?**

- As explained earlier go to **Add/Edit record.**
- Go to the Data search function.
- Enter a field that you would like to search by (by default it is record id, but you can also any reference or study number).
- Enter the value and select the correct record to be filled.


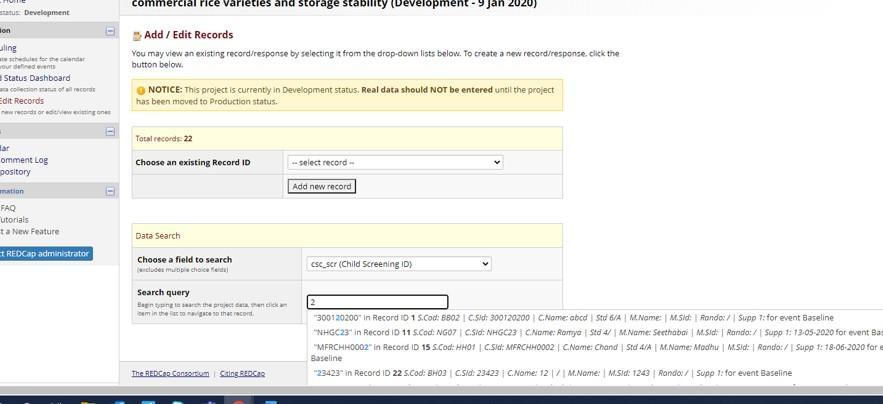


- You will land on the **Subject Record home page –** This page can help you see details of the subject individually, understand which forms are filled up and which are pending.
- Here you can select the correct form and enter the data, as per the correct event.
- Enter the data as recommended.
- Make sure that you select complete before saving the data**.**
- Once all fields are entered, click on **Save and Exit** Form, to save the data and go to the **Subject Home page. Any form, please make sure that you:**


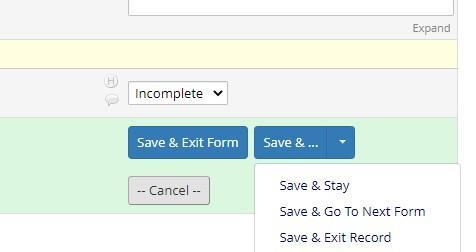


# **g. What is the Dashboard?**

- Click on the **Record Status Dashboard**
- You will see the tracking board to get all the subjects in the list and against each subject, you can view the longitudinal data organized as events**. Each subject with Name and Group**
- The legends mentioned include red dots for incomplete forms, green dots for completed forms and multiple dots for different statuses in multiple forms.
- Click on the **record id** to go to the respective **subject home page.**
- Click on the respective form of a subject to get to a particular form.


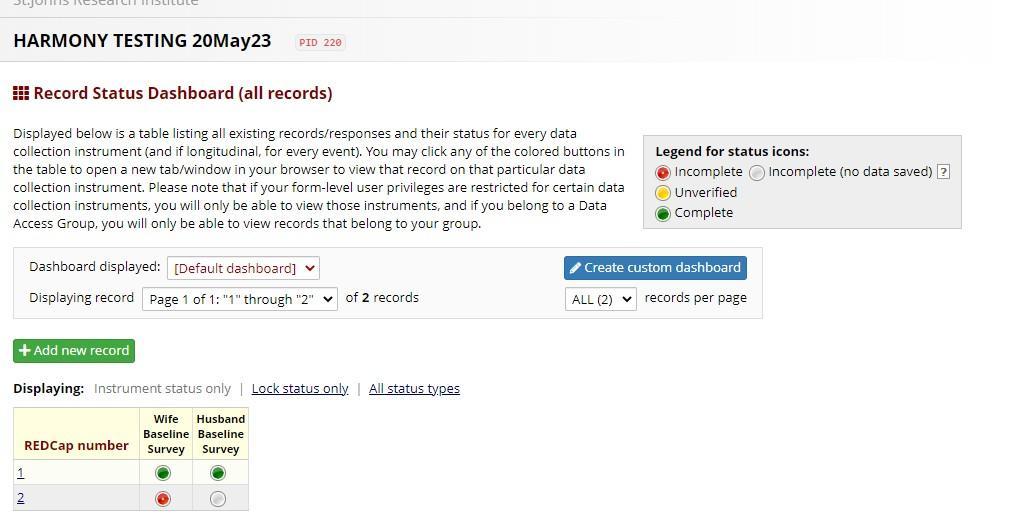


# **h. How to download the data?**

- This function is given only for administrators.
- Click on **data exports and reports.**


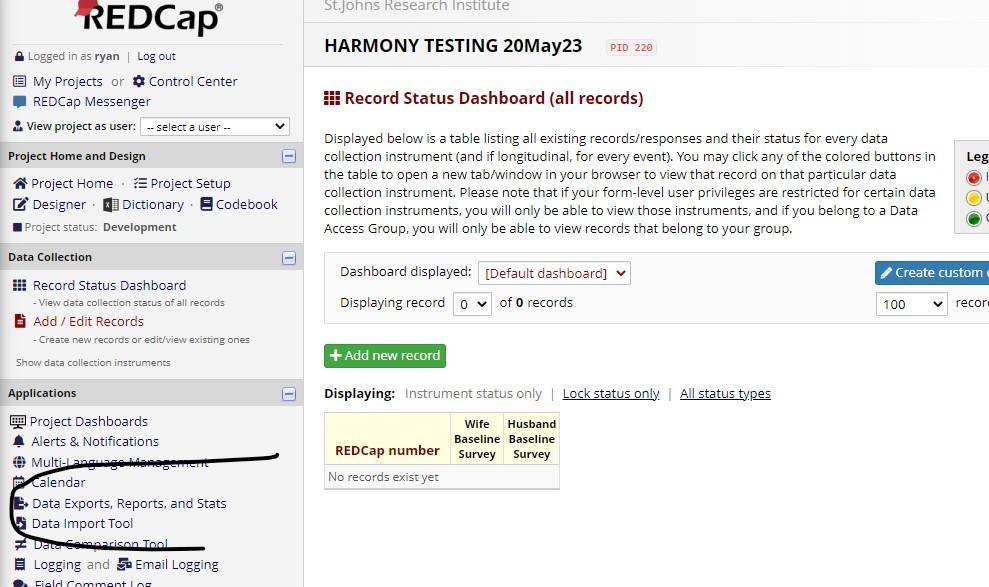


- Select Export data.


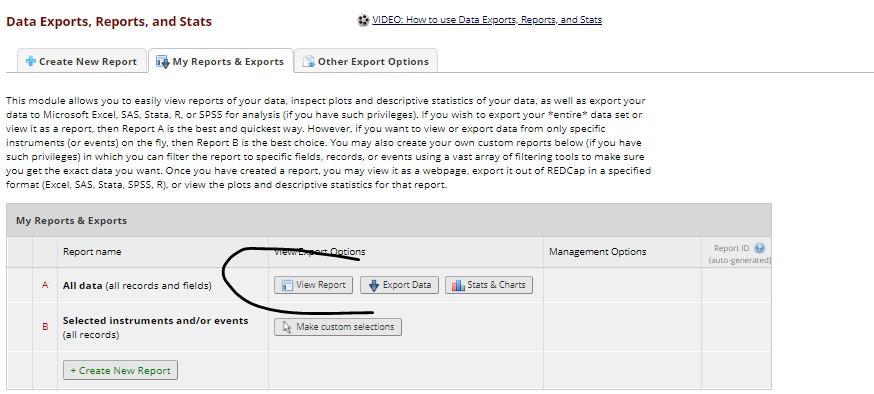


- Select the format you wish for (raw data for analysis or label for viewing) and then click on **Export data.**


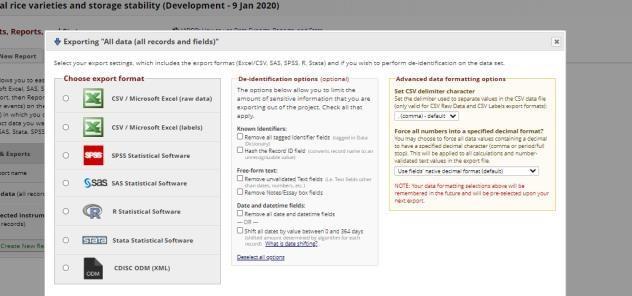


# **i. How to view reports?**

- Created reports are only for administrators.


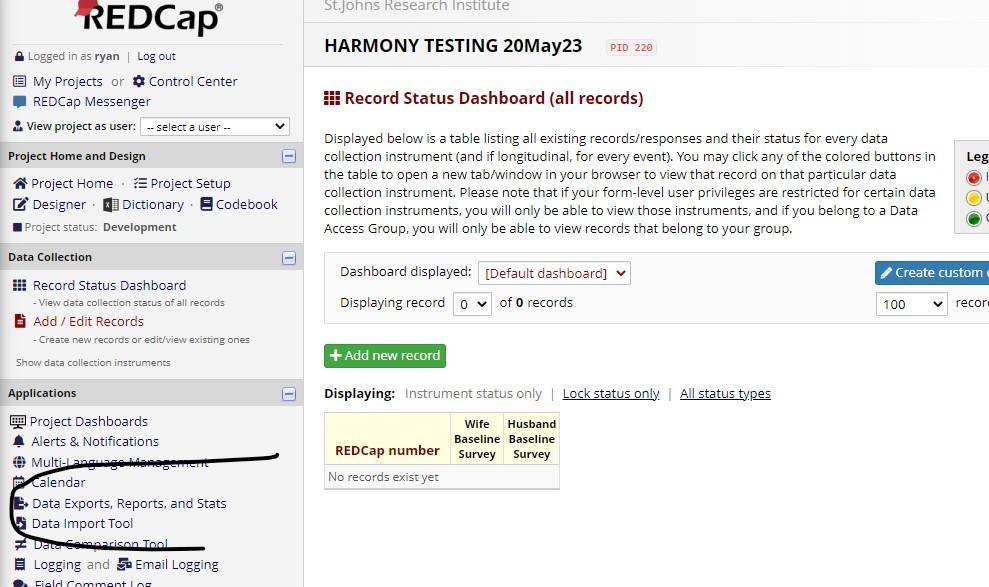


- Click on **Data Exports and Reports.**
- A list of customized reports is available as Export **data.**
- Click on **View report** to view the report or to export the report.


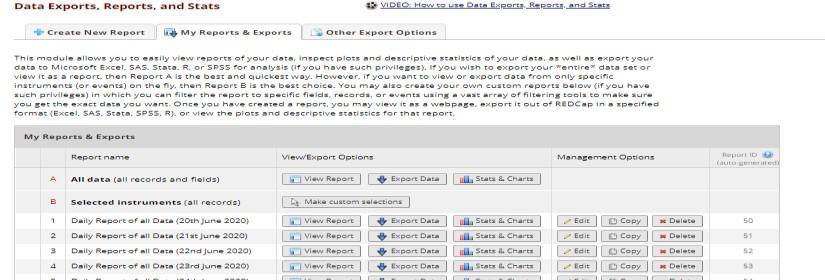


# **How to lock the forms?**

1. It is suggested for each site, a supervisor will be available to lock the forms.
2. Accordingly, rights will be issued.
3. The supervisor can view each form, and ensure that the data is appropriate.
4. Once this check is done, at the bottom following **Complete?** click on lock and **Save and exit** the form.
5. Once forms are locked, to understand the lock status of different forms click on **Displaying; Lock status.**


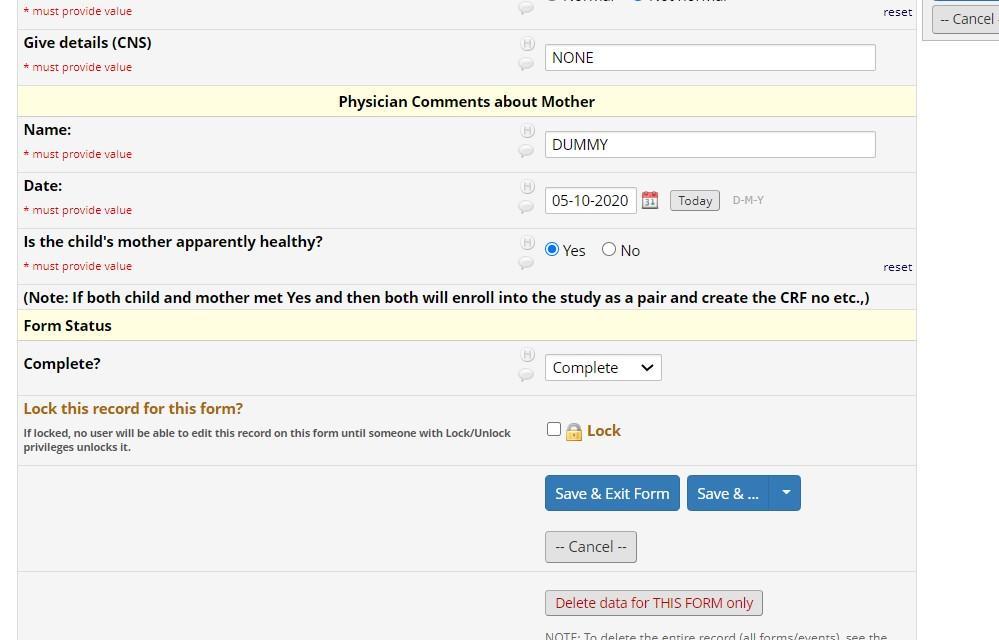


# k. **How to schedule using calendar?**

# The Schedule Generator will allow you to **generate a new schedule** based upon your Events and their Days Offset that have been defined on the [Define My Events](https://redcapcloud.sjri.res.in/redcap_v12.1.2/Design/define_events.php?pid=220) page. You may generate a schedule for a new or existing REDCap number below by selecting a Start Date, which will be used as the starting point for projecting schedule dates using your Days Offset. Once scheduled, you may then view it on the [Calendar](https://redcapcloud.sjri.res.in/redcap_v12.1.2/Calendar/index.php?pid=220), after which, if desired, you may also perform data entry for that calendar event. You may create a new project record here while performing scheduling or you may choose a currently existing one that has not yet been scheduled.
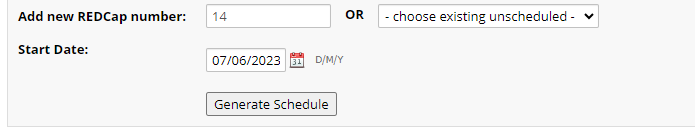


# **L. Important**

1. Always make sure your internet connectivity is there to ensure communication with the SJRI Redcap Server
2. Always go to the correct link and correct project (**Live or UAT** accordingly)
3. Please make sure that you do not leave your account open unsecure, even for a moment. If you need a break, please logout and then rejoin.
4. If you leave a session for more than 15 mins, you will be timed out and you need to login again. Data loss can also happen, and hence it is important to enter the data then and there and save it.
5. Please use only your id to enter your subject data. If you choose a different ID you may not see all your subjects.
6. Each user will have restricted rights on Redcap as per their role. Please check with your supervisor with about this, before contacting the Redcap Team for rights-related issues.
7. Remember you are responsible for entering your data, and hence do not share or ask others for their usernames/passwords.
8. A separate manual is available for the **Redcap Mobile app for offline data entry.**
9. In case of any issues related to redcap, please send a mail to Redcap support calpreg.rch@sjri.res.in.
10. In case you see an error like below, please notify the Database administrators.


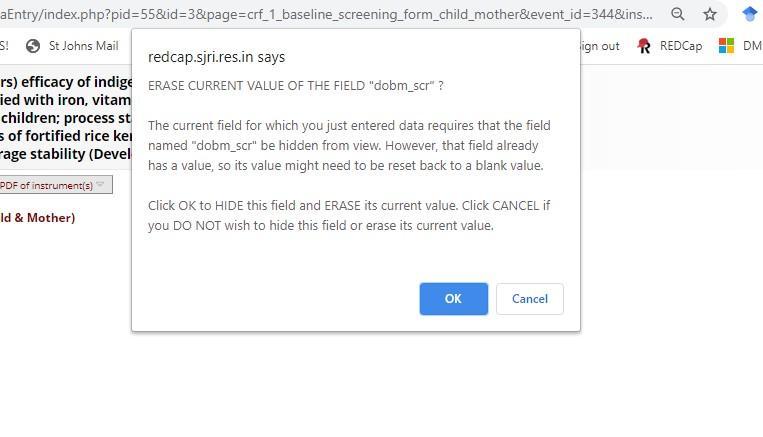


**Checks and Troubleshoot - REDCap**

The potential problems in the field include:

1. Problem with accessing Wi-Fi / Mobile internet
2. Problem with the device
3. Problem with accessing the server

Check access to network (WiFi/ Mobile), If there is no access, interviewer can

- 1. Restart the device
  2. Check if data (mobile data or Wifi) is turned off
  3. Check the Device is in airplane mode
  4. Check if the Device has been recharged (mobile network currency)

Check the device functioning.

- 1. Latest version of browser functioning (Google Chrome/FireFox/Microsoft Edge)
  2. URL is as provided: <https://redcapcloud.sjri.res.in/>
  3. Battery is fully charged
  4. No unnecessary apps installed or functioning (such as Video channels, Photos, media, etc)
  5. No unnecessary data stored
  6. No recent damage to the Tab (fall, Electric, water immersion, accidents etc)

1. Check the Access to the server
   1. Check if the ULR is correct <https://redcapcloud.sjri.res.in/>
   2. Do a Speed test <https://www.speedtest.net/> (at least 2-3 Mbps upload and download speed)
   3. Check if any other website is working: <https://sjri.res.in/>

If the trouble persists, the interviewer will inform the project manager and proceed to conduct the interview using paper questionnaire.

The project manager will escalate the issue to project administrator, who can

1. Contact the mobile service provider for further assistance in case it is a network related issue
2. If still not working, contact the SJRI IT team and the Corporate Device Support
3. If the server is not accessible, contact SJRI REDCap team by sending an email with a screenshot of the error, Speed test report (screenshot). Email ID: [calpreg.rch@sjri.res.in](mailto:calpreg.rch@sjri.res.in)

**Appendix AE – Check list - Health fairs**

Materials required:

- Digital blood pressure machine
- Glucometer and strips
- Cotton swabs
- Spirit
- Disposable gloves
- Measuring tape
- Weighing scale
- Batteries
- Note pads
- Pends
- Tissue paper
- Container to dispose bio-hazard waste like cotton swab and glucometer strips.
- Tabs and chargers
- Electrical spike buster for charging
- Forms, referral letters, reimbursement vouchers to be used by the assessment team.
- Breathalyzers
- Study pamplets
- Register book
- Mask
- Sanitizer
- Cash to cover any emergency expenses.

Instructions to logistics personnel:

- Confirm availability of the hall (1 week in advance and again reconfirm 1 day in advance)
- Place order for additional furniture required if any from a local vendor, two days in advance
- Keep all the materials needed for the session ready as per the above list (two days in advance)
- Reminder to Community health physician (place a call 1day in advance)

# **Appendix AF** **: Leave and Break Policy**

**Attendance policies:**

*Log in at Office*

Official work time of SJRI is 9 to 4:30pm unless permission has been given for a different schedule.

All team members will log-in in the biometric machine during login and log out in the St.John’s Research Institute. The field teams, Assessment and Intervention will report log in and log out on a WhatsApp group as well for the coordinators to be aware of the team’s log in and log out time. Team members logging in at SJRI will log in in the biometrics of SJRI and sign the attendance register in the office. All these measures are to enable calculation attendance and to answer questions from the HR department.

*Late log-in*

If anyone arrives past 9:15 am, their coordinator and the Project Manager need to be informed. The coordinator will keep the Admin team informed on the “Harmony Grant Attendance” whats app group for cross reference.

Team members are allowed to arrive late only 2 times per month as per SJRI rules. The 3^rd^ and subsequent late arrivals will result in deduction of a “casual leave” or leave without pay.

*Log-in in the Field*

Log-in at all other sites will be at 9:30 considering travel time. Any log in beyond these times will be considered as late and standard procedure will apply.

*Log out from field*

At the end of the day, while in the field, the team members will coordinate with their coordinator as to if they can log out directly from site if they are still in the field at 4:00pm. The coordinator will make a plan and then communicate to the manager as to who will be bringing the study materials to SJRI.

All this will be updated on the “Harmony Grant Attendance” WhatsApp group.

*Working late / early (Note: Needs prior approval from the Coordinator / Project Manager)*

If any team members logs out after 5:30pm they can log in by 10:30am. Any log in beyond 10:30am will be considered as "late" and standard procedures will apply.

No compensatory time off will be considered, unless the schedule is continuous.

Team members cannot log in late anticipating a late log out that day.

If anyone logs in before 7:30am they can log out by 3:30pm.

All early log-in and late log-out must be logged into on the biometrics / office register and the coordinators need to be notified.

***Working on Sundays***

***If the team members are scheduled to work on Sundays, which is the official weekly off at SJRI, those particular team members can take an off on Saturday instead.***

**Leave Policy:**

*Earned Leave:*

Earned leave will follow SJRI policy. Staff members will need to give at least 15 days prior notice to take earned leave. This needs to be approved by the coordinator and the project manager. Unless an emergency, no one will take leave when US team members are visiting.

*Casual Leave (CL), Restricted Holiday (RH), and Compensatory time off (CO)*

For staff members taking Casual leave, RH and CO at least one week prior notice to the Project Manager is required. All leaves must be requested via email sent to the Project Manager and copied to the respective coordinators. Once the coordinators approve the leave, the team members can apply on the portal. Leave is only granted once it has been formally approved by Dr Srinivasan (or a designee) through the portal.

For each period of leave, staff members are expected to send the project manager a document with the following information:

1. Staff members who will be available on their respective team (e.g Harmony Grant assessment, tracking etc.).
2. List of tasks the staff member is responsible for that need to be completed during the requested leave period.
3. Staff member(s) who will cover the responsibilities during leave.

*Comp off*

Comp off will be permitted for the team members for working on Public holidays, government declared holidays, Sundays and Saturday after working hours (if the team members work for 6-7 hours).

Working on holidays needs prior approval by the Project Manager and should be justified by the Coordinators.

Comp off should be availed within a month from the date a team member worked. A letter (Template available with the Admin team) needs to be submitted a week in advance, validated by the coordinator and approved by the Project Manager.

Comp off cannot be adjusted with any unplanned or emergency leaves and cannot be permitted to be carried forward to the next month.

*Exceptions to leave policy:*

1. Emergency leave is granted for personal or family emergencies and may include personal or family illness, injury, medical emergency, death, or other urgent needs.
2. All staff members are required to be present when either UCSF PIs or other UCSF team members visit. As these visits are scheduled in advance, staff team members are expected to be available unless there is an exceptional circumstance, such as emergency leave.

*Emergency Leave*

Emergency leave is defined as medical emergency leave (oneself or close family), death of a family member, or if an employee is arrested. When one of the above defined emergencies occur, the emergency needs to be communicated to the project manager as soon as possible. If the emergency happens outside of office hours the staff member will need to send a message (e.g. WhatsApp, SMS etc) and let the coordinator know (1) what the emergency is, (2) how long the staff member will be absent from work, and (3) contact information during the emergency leave. The staff member is then required to notify their coordinator the following morning during office hours to handover any tasks that need to be completed during the extent of the emergency and have those tasks reassigned to another team member.

Exceptions for immediate contact of one’s coordinator will be for emergencies that cause a staff member to be indisposed (e.g. unconscious). In situations where a staff member has been arrested and has lost access to a phone, staff members should ask a family member or friend to contact their coordinator on their behalf as soon as possible.

In most cases emergencies are unpredictable, but if a staff member does foresee an emergency, the staff member should make every attempt to plan as far in advance as far as possible with one’s coordinator to ensure workflow is not interrupted.

*Unapproved leave*

Failure to be present during work hours without prior approval that does not meet the above medical or emergency guidelines, will be subject to leave without pay and will follow SJRI HR rules. Unapproved leave of more than one day that is not defined as emergency leave will result in unpaid leave. Team members taking unapproved leave for more than one day will need to write a letter addressed to the Dean stating the number of days leave has been taken without approval and without pay. This letter will be sent on behalf of the PI to the Dean, and subsequently forwarded to HR. Multiple infractions to this policy may result in termination of employment.

*Break time policy:*

Two 15-minute breaks between 11am to 12:00pm and 3pm to 4:00pm, and (This is more an unwritten rule and is not in the SJRI rule list.) a one hour break for lunch are permitted each working day.

For staff members arriving late into work (beyond 10am, unless logged in late due to a work related late log out) will not be allowed to take breaks until lunch. If team members take breaks longer than permitted, it will be counted as part of the total break time. If there is a circumstance in which the employee requires a break time longer than permitted, employees are required to request permission from their coordinator prior to the extended break.

**Appendix AG: Invoicing procedures:**

The project manager in collaboration with the project administrator, will put together monthly invoices based on the budgeted amounts from the sub-contract. A draft of this invoice is sent to the UCSF project coordinator for review.

The UCSF project coordinator reviews the invoices to ensure that each invoice not only accurately reflects project spending, but is also compliant with UCSF and NIH rules

After review, the project coordinator will send the approved invoice to the project manager.

The Project Manager at SJRI is responsible for following SJRI procedures to obtain the signature of The Director of St John’s National Academy of Health Sciences (SJNAHS) on the invoice in a timely manner, and then submitting the signed invoice to the UCSF Project Coordinator. Upon receipt, the Project Coordinator will submit the invoice promptly for reimbursement.

Once the invoiced funds are transferred to the CBCI bank account in Bangalore, the Project Coordinator at UCSF will provide the tracking info to the SJRI-based project administrator, who will relay the information to the Accounts department at SJRI. The UCSF Project coordinator will send a letter explaining how to deduct costs for indirects and rent. This letter will need to be submitted to the SJRI accounting department with a cover letter signed by the Indian PI. A sample invoice is included in the appendix.

As indicated in the sample invoice in the appendix, rent always will need to be calculated to be 7% of direct costs (personnel+travel+other). The 7% rent will then be summed with direct costs to calculate total direct costs. Indirect costs will be 8% of total direct costs. The total invoice amount will result in total direct + indirect costs.

The UCSF Project Coordinator will prepare invoices to comply with the NIH requirement that all costs and financials are in U.S dollar using an exchange rate applicable at the time the invoice is submitted.

Rebudgeting

Any changes to a budget category (personnel, travel, other, rent) must first be approved by the PI with a justification for the budget change. With the approval of the PI, the budget may be revised to reappropriate funds from one budget category to another budget category. Rebudgeting cannot result in an increase or decrease of the approved budget total.

Budgetary changes of more than 25% to headings will require prior approval from UCSF/NIH. The PI will notify UCSF for the need to modify the budget and submit a budget justification specifying why the modification is necessary.

**Appendix AH: Qualitative IDI Guides**

**M3 In-depth Interview – Husband**

**INSTRUCTIONS for the Interviewer: How to use the IDI Guide**

- Section topics are underlined.
- *Instructions/suggestions to interviewer are in italics and [brackets].*
- Scripts to introduce the IDI, sections and questions appear in normal font prior to the section/questions.
- Primary interview questions: are numbered.
- They address the topics that you as the interviewer must ask and discuss with participants.
- The questions are suggestions for getting the discussion going. You are not required to read them verbatim, but they are written to ensure some consistency across IDIs.
- You may adapt the questions and/or ask them in a different order, depending on how the interview develops. However, you will have to ensure that by the end of the interview, all the topics and key themes have been covered.
- Probing topics: are indicated with a bullet.
- If you find that the participant provides little information in response to the primary question, these probing topics may be used to encourage further discussion.
- You are not required to cover every topic listed. So, depending on what has already been discussed, and the IDI context, you may ask these probes or not.

**Introduction:** Thank you for speaking to me today. As we just discussed in the consent form, the information you provide today will help us to understand your experience in the *Harmony* study and will help us improve the intervention for other families like yours. You do not need to disclose any personal information, if you don’t want to, but we would like you to be our expert today. Please do not hesitate to tell us what you think and feel about the intervention. I assure you that what you tell me in our conversation today will not be shared with anyone outside of the study. All bad/negative and positive feedback and experiences are welcome. Nothing that you say may offend us or make us feel bad, and that you won’t be judged. The information that you provide today will help us improve in the future.

[**Note to interviewer**: start conversation with an appropriate warm-up question like about how the participant is doing, the weather, a holiday, or any other topic that is not controversial.]

**General study participation**

Before we talk about specific parts of the study, I’d first like to ask you about your experience with the *Harmony* study in general.

1. When you first found out about the study, what influenced your decision to join?
   - How did things like a desire to change your alcohol use, a desire to improve family relationship, or pressure play a role? What else influenced your decision?
   - How were other people involved in the decision (e.g. who did you discuss it with, who decided, how that affected you, etc.)?
2. Were you able to attend all the study visits? Why or why not?

**Intervention experience**

3. We’d like to talk more about your participation in the *Harmony* study and what you thought of the intervention. First, what was the most important part of the intervention in your opinion and why? By intervention we mean the counselling sessions that you attended alone and the sessions that you attended with your wife.
   - What do you think of first when you think about the intervention? Why do you think of that part first?
4. What part(s) of the intervention were most challenging for you? Why did you find this part challenging?
   - What was difficult to do, such as in session activities, or homework?
   - Can you tell me what you thought about the timing, frequency, or duration of sessions? What about your relationship with the counsellor? What about the content of counselling?
   - How did other things happening in your life make it challenging (e.g., family illness, work changes, intervention conflicted with cultural beliefs or values, other things at home, etc.)?
5. What part(s) of the intervention were easiest for you? Why did you find this part easy?
   - What about things like the timing, frequency, or duration of sessions? What about your relationship with the counsellor? What about the content of counselling?
   - How did other things in your life make it easier (e.g., family support, other things at home, etc.)?
6. How supportive of your participation were your family/the people you lived with (i.e. children, parents, other relatives, other people in the house)?
   - How did they help you?
   - If they were not supportive, what do you think were their reasons?
   - How did they show they weren’t supportive?
   - In what ways do you think they could’ve been helpful?
7. If you were offered a chance to participate again, would you take it? Why or why not?
8. If you did participate in this study again, how would you suggest we change it for next time? You can tell us as many recommendations as you’d like.

*Alcohol use*

Since the study had a focus on drinking, the next topic we’d like to talk about is your drinking and how the study might have change it, or not.

9. What changed about your drinking after you completed the study?
   - What changed about your drinking behaviors, like how much you drink, when you drink, and where you drink?
   - What about your motivation to change your drinking behaviors? Or how you tried to change?
   - How have these changes affected you/your life positively or negatively (i.e., how you feel about yourself physically and emotionally, your relationship with others, your work life, your home life, etc.)? What positive or negative effects of drinking did you experience before?
10. In your own words, how would you describe how the intervention affected your drinking?
    - Can you tell me about the strategies you used to change drinking behaviors, (i.e., problem solving skills, drink refusal skills, skills for managing drinking urges, and emotional management skills)?
    - What else about the intervention contributed to the change and why, (e.g., counsellor relationship, counselling content, etc.)?
    - What other changes did this create in your life, positive or negative? How did these happen?

*Relationship quality*

Thank you for sharing so far, we have one last topic to discuss. Since the intervention was also designed to improve your relationships, we’d like to talk about how the intervention may have impacted your relationship with your wife. Please remember that we will not share what you say with your wife or with anyone else.

11. How did your relationship(s) with your wife change once you started participating in the study?
    - How would you describe your level of trust now? How does it differ from before the study?
    - What is your communication like with your wife? What is different about it? What was it like before?
    - How do you make decisions together? How does this compare to before?
    - How do you spend your time together? How is this different?
    - What else would you say about how you act with your wife now compared to before the study?
    - What caused these change(s)?
    - How do you feel about the change(s)?
12. All couples have disagreements and sometimes these disagreements can lead to conflicts or fights. Can you tell me about a specific time after the study when you and your wife had a conflict?
    - What was the conflict about?
    - Who started the conflict?
    - How did you handle and express your feelings? What about your wife?
    - How did it resolve?
    - What do you do differently now when you have a conflict compared to before the study? What made you act different?
    - How have the topics of conflict changed since before the study?
13. How would you describe the role of your drinking in your conflicts?
    - What topics do you have conflict on when you drink? How do the conflicts start? How do they escalate?
    - How would you describe how your drinking affected your ability to problem solve or manage your emotions?
    - How is this different after the study?
14. What other changes, if any, would you describe experiencing since you joined the study?
    - How do you feel about yourself now? How do you feel about your mental health? What about your social well-being?
    - How have your other relationships with family (i.e. mother-in-law, siblings, etc.), friends, neighbors changed? How about your relationship with your children?
    - Have other people, such as your family and friends, described any changes in you after participating in the intervention?
    - How do you feel about your household finances? How is it different from before? And why?

**Wrap-up**

We’re almost finished with our discussion today, we just have a couple more questions.

15. Do you have anything else you’d like to share about the study or how it has changed your life?
16. Do you have any questions for me?

**M3 In-depth Interview – Wife**

**INSTRUCTIONS for the Interviewer: How to use the IDI Guide**

- Section topics are underlined.
- *Instructions/suggestions to interviewer are in italics and [brackets].*
- Scripts to introduce the IDI, sections and questions appear in normal font prior to the section/questions.
- Primary interview questions: are numbered.
- They address the topics that you as the interviewer must ask and discuss with participants.
- The questions are suggestions for getting the discussion going. You are not required to read them verbatim, but they are written to ensure some consistency across IDIs.
- You may adapt the questions and/or ask them in a different order, depending on how the interview develops. However, you will have to ensure that by the end of the interview, all the topics and key themes have been covered.
- Probing topics: are indicated with a bullet.
- If you find that the participant provides little information in response to the primary question, these probing topics may be used to encourage further discussion.
- You are not required to cover every topic listed. So, depending on what has already been discussed, and the IDI context, you may ask these probes or not.

**Introduction:** Thank you for speaking to me today. As we just discussed from the consent form, the information you provide today will help us to understand your experience in the *Harmony* study and will help us improve the intervention for other families like yours. You do not need to disclose any personal information, if you don’t want to, but we would like you to be our expert today. Please do not hesitate to tell us what you think and feel about the intervention. I assure you that what you tell me in our conversation today will not be shared with anyone outside of the study. All bad/negative and positive feedback and experiences are welcome. Nothing that you say may offend us or make us feel bad, and that you won’t be judged. The information that you provide today will help us improve in the future.

[**Note to interviewer**: start conversation with an appropriate warm-up question like about how the participant is doing, the weather, a holiday, or any other topic that is not controversial.]

**General study participation**

Before we talk about specific parts of the study, I’d first like to ask you about your experience with the *Harmony* study in general.

17. When you first found out about the study, what influenced your decision to join?
    - How did things like a desire to change your husband’s alcohol use, a desire to improve family relationship, or pressure play a role? What else influenced your decision?
    - How were other people involved in the decision (e.g. who did you discuss it with, who decided, how that affected you, etc.)?
18. Were you able to attend all the study visits? Why or why not?

**Intervention experience**

19. We’d like to talk more about your participation in the *Harmony* study and what you thought of the intervention. First, what was the most important part of the intervention in your opinion and why? By intervention we mean the counselling sessions that you attended with your husband and the sessions that he attended alone.
    - What comes to mind first when you think about the intervention? Why does this come to mind?
20. What part(s) of the intervention were most challenging for you? Why did you find this part challenging?
    - What was difficult to do, such as in session activities, or homework?
    - Can you tell me what you thought about the timing, frequency, or duration of sessions? What about your relationship with the counsellor? What about the content of counselling?
    - How did other things happening in your life make it challenging (e.g., family illness, work changes, intervention conflicted with cultural beliefs or values, other things at home, etc.)?
21. What part(s) of the intervention were easiest for you? Why did you find this part easy?
    - What about things like the timing, frequency, or duration of sessions? What about your relationship with the counsellor? What about the content of counselling?
    - How did other things in your life make it easier (e.g., family support, other things at home, etc.)?
22. How supportive of your participation were your family/the people you lived with (i.e. children, parents, other relatives, other people in the house)?
    - How did they help you?
    - If they were not supportive, what do you think were their reasons?
    - How did they show they weren’t supportive?
    - In what ways do you think they could’ve been helpful?
23. If you were offered a chance to participate again, would you take it? Why or why not?
24. If you did participate in this study again, how would you suggest we change it for next time? You can tell us as many recommendations as you’d like.

*Relationship quality*

Thank you for sharing so far. Since the intervention was also designed to improve your relationships, we’d like to talk about how the intervention may have impacted your relationship with your husband, but first we’ll talk about your relationship before the study. Please remember that we will not share what you say with your husband or with anyone else.

25. Now I’d like to talk about your relationship with your husband. How would you describe your relationship before you joined the study?
    - What would you talk about? How did you talk with one another?
    - How did you make decisions together?
    - How did you spend your time together?
    - How would you describe your level of trust in him?
    - How would you describe your expectations and desires in the relationship?
    - Describe a typical day. How did you treat each other / interact with one another? What did you do together?
26. All couples have disagreements and sometimes these disagreements can lead to conflicts or fights. Can you describe a typical conflict in your relationship before the study?
    - What triggered the conflict? What did you usually have conflict about?
    - Who usually started the conflict?
    - How would you handle and express your feelings? What about your husband?
    - How was it resolved?
27. How would you describe the role of your husband’s drinking in your conflicts?
    - What do you fight about when he’s drinking?
    - How does the conflict start when he’s drinking?
    - What happens? How does it escalate? How does it end?

*Intervention effect*

28. We know that many women experience violence and that are many reasons why men become violent. By violence we mean verbal, physical, or sexual abuse. What are the situations in which your husband is likeliest to become violent?
    - Can you describe a typical situation when your husband might be violent in the past? How is it different now?
    - Did anything about the intervention affect these conditions, either for better or worse? What aspects of the intervention led to these changes? How did they lead to this change?
    - What changed in terms of verbal abuse? What about physical abuse? Sexual?
29. We’d like to talk more about how the intervention may have impacted your relationship in addition to the topic of violence. How did your relationship(s) with your husband change once you started participating in the study?
    - How did your husband’s alcohol use change? How did this change your relationship?
    - How is your trust now? How do you talk to each other now? How do you make decisions? What else is different?
    - What do you think caused the change(s)?
    - How do you feel about the change(s)?
30. Can you tell me about a specific time after the study when you and your husband had a conflict?
    - What was the conflict about?
    - Who started the conflict?
    - How did you handle and express your feelings? What about your husband?
    - How did it resolve?
    - What was different from past conflicts you’ve had? What made it different?
    - How were you different during the conflict?
31. What other changes, if any, would you describe experiencing since you joined the study?
    - How do you feel about yourself now? How do you feel about your mental health? What about your social well-being?
    - How have your other relationships with family (i.e. mother-in-law, siblings), friends, neighbors changed? How about your relationship with your children?
    - Have other people, such as your family and friends, described any changes in you after participating in the intervention?
    - How do you feel about your household finances? How is it different from before? And why?

**Wrap-up**

We’re almost finished with our discussion today, we just have a couple more questions.

32. Do you have anything else you’d like to share about how the study worked or how it has changed your life?
33. Do you have any questions for me?
