# Supplementary material for: Protocol for a randomized controlled trial of a combined motivational interviewing and behavioral couples therapy intervention to reduce intimate partner violence and alcohol use in south India
Source: PLoS One. 2025 Dec 1;20(12):e0335332. doi: 10.1371/journal.pone.0335332 (PMC12668616; doi:10.1371/journal.pone.0335332)
Supplement: Attachment — Submitted filename: Response to Reviewers.docx [file pone.0335332.s002.docx]

August 10, 2025

Emily Chenette

Editor-In-Chief, *PLOS One*

Dear Reviewers,

Our original manuscript (PONE-D-25-17170) was rejected because it lacked an updated ethics review letter from the University of California, San Francisco (UCSF) Institutional Review Board, which we have included in this re-submission.

***Reviewer Comment:***

You have not submitted the requested ethics approval extension documents. It is therefore not clear whether you obtained the necessary ethical approval for the study to take place. Please be aware that we expect all research involving human participants and/or medical data to have been approved by the authors' Institutional Review Board (IRB) or by an equivalent ethics committee(s).

**RESPONSE:** We have provided updated (continuous) UCSF IRB approval as supporting documentation.

We look forward to your review of our manuscript.

Sincerely,

Megan Ramaiya, PhD, MSc

Department of Psychiatry & Behavioral Sciences

University of California, San Francisco

[Megan.Ramaiya@ucsf.edu](mailto:Megan.Ramaiya@ucsf.edu)
